# Deprotective Functionalization: A Direct Conversion of Nms‐Amides to Carboxamides Using Carboxylic Acids

**DOI:** 10.1002/anie.202318304

**Published:** 2024-03-19

**Authors:** Philipp Spieß, Jakub Brześkiewicz, Ricardo Meyrelles, David Just, Nuno Maulide

**Affiliations:** ^1^ Institute of Organic Chemistry University of Vienna Währingerstraße 38 1090 Vienna Austria

**Keywords:** Deprotection, Protecting Group, Sulfonamides, Amides, Carboxylic Acids

## Abstract

The nature of protecting group chemistry necessitates a deprotection step to restore the initially blocked functionality prior to further transformation. As this aspect of protecting group manipulation inevitably adds to the step count of any synthetic sequence, the development of methods enabling simultaneous deprotection and functionalization (“deprotective functionalization”—distinct from “deprotection followed by functionalization”) is appealing, as it has the potential to improve efficiency and streamline synthetic routes. Herein, we report a deprotective functionalization of the newly introduced Nms‐amides guided by density functional theory (DFT) analysis, which exploits the inherent Nms reactivity. Mechanistic studies further substantiate and help rationalize the exquisite reactivity of Nms‐amides, as other commonly used protecting groups are shown not to exhibit the same reactivity patterns. The practicality of this approach was ultimately demonstrated in selected case studies.

## Graphical abstract

We present a concept termed “deprotective functionalization”, which we have applied to the coupling of Nms‐protected amines with carboxylic acids. Notably, this approach circumvents the need for a dedicated amine‐deprotection step and obviates the use of additional reagents for carboxylate activation. The reaction was shown to be, in many cases, superior to deprotection followed by functionalization, both in terms of yield and atom economy.

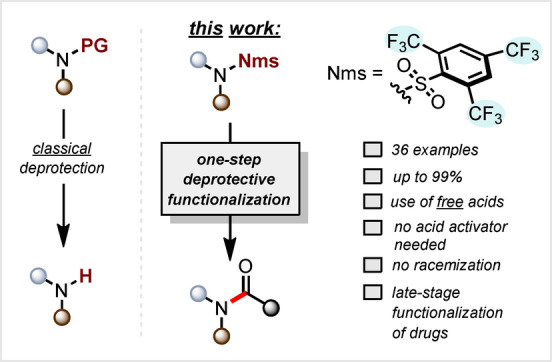

Protecting groups are a key staple of the synthetic repertoire, be it in natural product synthesis^[1]^—despite efforts to circumvent their use[^2^, ^3^, ^4^]—or in the production of pharmaceuticals and fine chemicals.^[5]^ Often installed strategically at a relatively early stage of the synthetic sequence, their high chemical stability is paramount so as to allow their survival throughout a number of functional‐group manipulations. In contrast, deprotection must occur under mild conditions and selectively, ensuring that other sensitive functionality is not adversely affected. Importantly, only after this deprotection has occurred can further functionalization take place. Despite undeniably valuable progress in the field of protecting group chemistry,[^6^, ^7^, ^8^] the inevitable addition to step count remains the major deterrent to this field given the non‐strategic nature of protection and deprotection steps.^[9]^

A hypothetical synthetic transformation that would enable simultaneous deprotection and functionalization (conceptually distinct from “deprotection followed by functionalization”) of a given protecting group in a single operation would, thus, constitute a significant advance. If viable, this approach also holds the promise of substantially reducing the quantity of reagents necessary in a conventional two‐step procedure. Nevertheless, such an approach and its implementation in a practical and general synthetic context have thus far remained elusive (Scheme 1A).

**Scheme 1:**
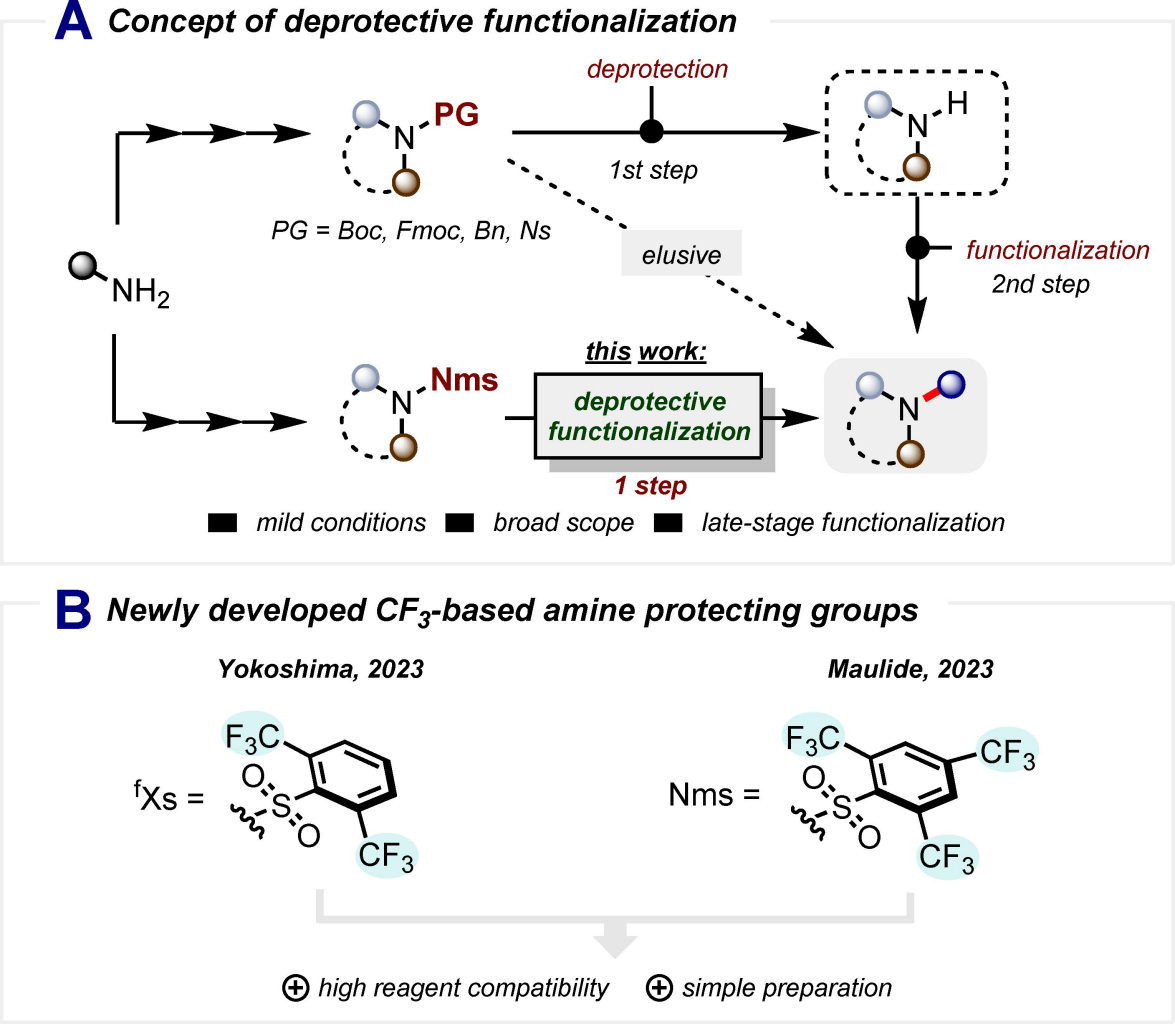
A) Concept of deprotective functionalization. B) New sulfonamide‐based amine protecting groups.

The amino group is a common functionality in organic synthesis which, owing to its basicity and pronounced nucleophilicity, often mandates the installation of a protecting group.^[10]^ Among amine‐protecting groups, sulfonamides have gained widespread popularity.[^11^, ^12^, ^13^] Conventional benchmark sulfonamides such as Ns(nosyl)‐amides and Ts(tosyl)‐amides, however, have historically forced chemists to compromise between chemical stability and a reliable and mild deprotection. To address this issue, our group and Yokoshima's team have recently introduced newly developed sulfonamide protecting groups: Nms‐amides^[14]^ and ^f^Xs‐amides^[15]^ (Scheme 1B), respectively.

Inspired by the serendipitous observation of urea formation in the presence of carbonates (Scheme 2), we speculated whether Nms‐amides could provide unexplored opportunities for alternative deprotection methods, a reactivity we propose to term “deprotective functionalization”. Herein, we present the discovery of a general and simple deprotective functionalization protocol which deploys Nms‐amides for the synthesis of carboxamides. Despite considerable development of alternative approaches for the synthesis for carboxamides,^[16]^ there is still continued high interest in further development.[^17^, ^18^, ^19^, ^20^]

**Scheme 2:**
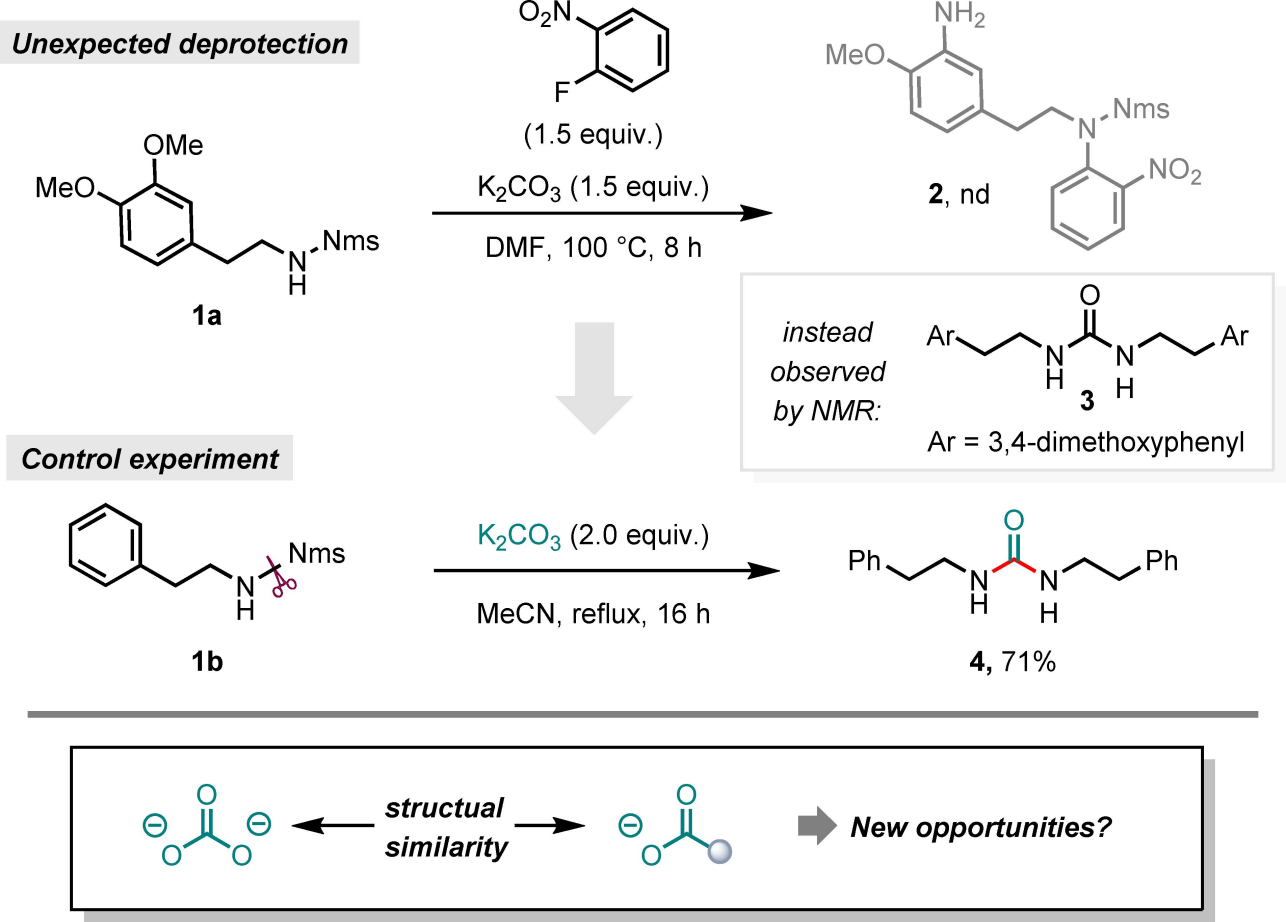
Serendipitous discovery of a deprotective functionalization reaction of Nms‐amides.

While studying the nucleophilic aromatic substitution of 1‐fluoro‐2‐nitrobenzene with a secondary Nms‐amide (**1 a**), we observed the unexpected formation of the symmetrical urea product **3** instead of the anticipated product **2** (Scheme 2). In trying to understand this result, we speculated that the Nms‐amide (**1 a**) could undergo N−S bond cleavage by virtue of an addition‐elimination reaction induced by the carbonate source (in this case potassium carbonate) at elevated temperatures. Pleasingly, in a control experiment with Nms‐amide **1 b** and in the presence of only K_2_CO_3_ in refluxing acetonitrile, the symmetrical urea **4** was formed as the sole product in good yield. This urea formation is mechanistically interesting (with no precedent in the literature),^[21]^ albeit synthetically limited,^[22]^ and led us to hypothesize that alternative modes of deprotection might also be conceivable, particularly given the structural similarity of carbonates and carboxylates (see Scheme 2, bottom).

Particularly, we anticipated that a carboxylate anion could exhibit sufficient nucleophilicity to perform an *ipso*‐substitution on the aryl‐sulfonamide, forming an activated ester (**7**) (Scheme 3A). Consequently, in a second step, the liberated amide **6**, formed through SO_2_ extrusion from **5**, could be acylated by **7** to form an amide,^[23]^ ultimately releasing the phenolate **8** as a byproduct. Prior pioneering studies by Tomkinson showed that 2,4‐dinitrobenzenesulfonamides can be converted to amides in a similar fashion (Scheme 3B),[^24^, ^25^] albeit through a procedure mandating the use of thioacids (RCOSH)—often unstable substances that must themselves be laboriously prepared from carboxylic acids, carbonyl chlorides or thioesters, employing toxic sulfur‐containing reagents (i.e. sodium sulfides) (Scheme 3B).^[26]^ Moreover, the comparatively low chemical stability of dinitrobenzenesulfonamides, restricts their use and renders them an otherwise rare protecting group.[^14^, ^27^]

**Scheme 3:**
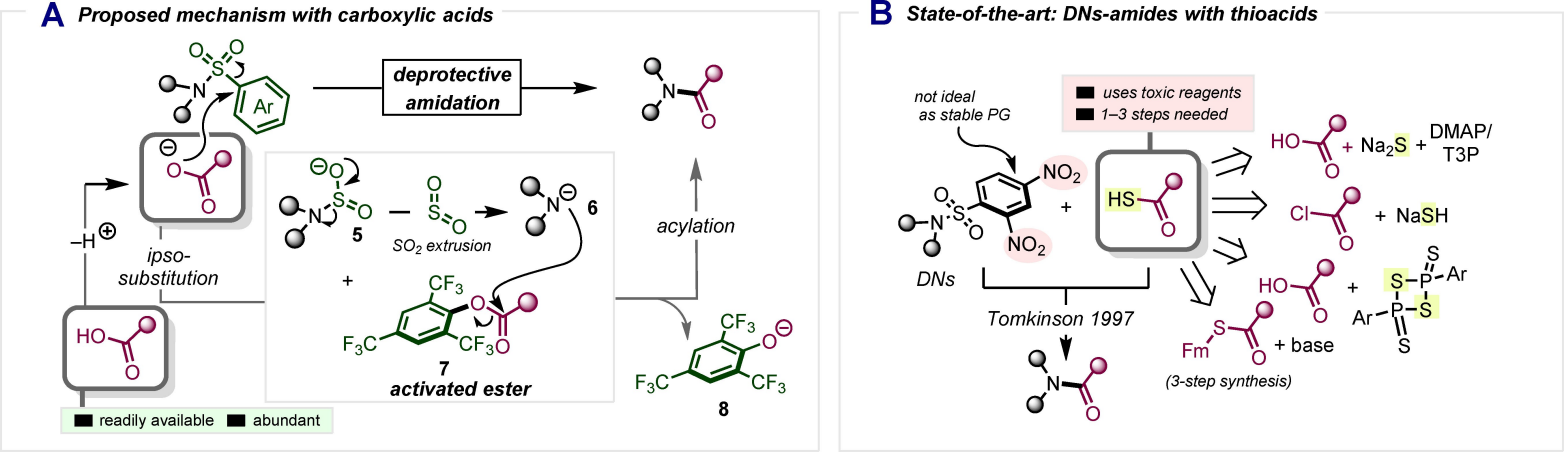
A) Proposed mechanism for deprotective amidation. B) DNs‐amides and thioacids. T3P=propanephosphonic acid anhydride. Fm=9‐fluorenylmethyl.

To verify the plausibility of our assumptions, the *ipso*‐substitution of tertiary Nms‐amides with carbonate and carboxylate was computed at the DFT: ωB97XD/def2‐TZVP,SMD//ωB97XD/def2‐SVP,SMD[^28^, ^29^, ^30^] level of theory (Scheme 4A). Gibbs free‐energy profiles were obtained starting from the separated reagents (Nms‐amide and carboxylate for **IA** and Nms‐amide and potassium carbonate for **IIA**). The interaction of Nms‐amide with phenyl carboxylate or potassium carbonate yields the reactant complex structures **IA′** and **IIA′**, respectively. Notably, the initial aggregations already display a significant energetic difference, as the reaction with carbonate produces a much more unstable complex (Δ*G*(**IIA**→**IIA′**)=22.2 kcal/mol) than that with carboxylate (Δ*G*(**IA**→**IA′**)=8.4 kcal/mol).

**Scheme 4:**
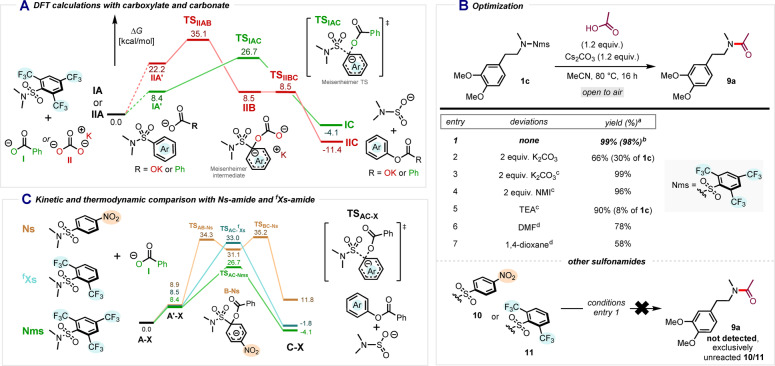
A) Computed reaction profile (ΔG_298_), in kcal/mol for the *ipso*‐substitution with potassium carbonate and phenyl carboxylate.; B) Optimization table of deprotective amidation. (a) NMR yield using CH_2_Br_2_ as internal standard. (b) isolated yield. (c) at 100 °C with 2 equiv. of acetic acid. (d) 2 equiv. Cs_2_CO_3_ and acetic acid.; C) Comparative Gibbs free energy profile in kcal/mol for the *ipso*‐substitution of different protecting groups. The separated reactants (**XA**, for panel A, and **A‐X**, for panel C) were taken as a reference (0.0 kcal/mol). NMI=*N*‐methylimidazole.

The divergence in reaction pathway between **IA′** and **IIA′** becomes even more evident at this point. On the one hand, the former evolves to the complex **IC** via a Meisenheimer transition state undergoing concerted C−O bond formation and C−S bond cleavage. The reaction is exergonic (Δ*G*(**IA**→**IC**)=−4.1 kcal/mol), however, the obtained activation barrier for this step (Δ*G* ^≠^(**IA**→**IC**)=26.7 kcal/mol) suggests a necessity for elevated reaction temperatures.^[31]^ In contrast, for the *ipso*‐substitution with carbonate, the computed energy profile presents a stepwise mechanism, starting with the formation of a Meisenheimer complex intermediate (**IIB**), from which a barrierless cleavage of the C−S bond leads to deprotected intermediate **IIC**. The apparent activation barrier of this mechanism (Δ*G* ^≠^(**IIA**→**IIC**)=35.1 kcal/mol) poses a significant kinetic impediment for the reaction to occur, even though the reaction is computed to be exergonic (Δ*G*(**IIA**→**IIC**)=−11.4 kcal/mol). It is worth noting that mechanistic studies presenting Meisenheimer complexes as either intermediates or transition states have also been previously reported.[^32^, ^33^, ^34^] Given these encouraging computational results, we set out to investigate the performance of acetic acid in the deprotective functionalization of model sulfonamide **1 c** (Scheme 4B). After optimization, amide **9 a** was obtained in an excellent 98 % isolated yield using nearly stoichiometric amounts of acetic acid with Cs_2_CO_3_ as the base (thereby taking advantage of the large kinetic barrier for reaction with carbonate itself), while heating to 80 °C in acetonitrile. Importantly, this reaction required no special handling and could be performed under air. While K_2_CO_3_ also provided **9 a** in good yield (entry 2), a higher reaction temperature was required to ensure complete consumption of the starting material (entry 3).^[35]^ Interestingly, *N*‐methylimidazole and triethylamine also led to formation **9 a** in excellent yields (entries 4 and 5), while acetonitrile proved to be the solvent of choice (entries 6 and 7).

Based on these results, we considered whether other sulfonamides, particularly an Ns‐amide (**10**) and an ^f^Xs‐amide (**11**), might also undergo this deprotective amidation. Strikingly, however, subjecting **10** or **11** to the optimized conditions did not provide even traces of the desired product and the starting materials remained fully unconsumed (see Scheme 4B bottom).

The reactivity of these protecting groups was further studied computationally (Scheme 4C), revealing kinetic control and dissimilar mechanisms at play in this *ipso*‐substitution reaction. While the ^f^Xs group evolves through a concerted step, in similarity to the Nms group (Meisenheimer transition state), the reaction with the Ns group results in the formation of a Meisenheimer complex intermediate (**B‐Ns**). The computed activation barriers for both of these groups reflect their inactivity under experimental conditions (Δ*G* ^≠^(**A‐fXs**→**C‐fXs**)=33.0 kcal/mol and Δ*G* ^≠^(**A‐Ns**→**C‐Ns**)=35.2 kcal/mol). Furthermore, the *ipso*‐subsitution with Ns is endergonic (Δ*G*(**A‐Ns**→**C‐Ns**)=11.8 kcal/mol), imposing an additional handicap for the reactivity of this protecting group. We thus appear to have uncovered an Nms‐selective deprotective amidation under simple and mild conditions.

With optimized conditions in hand, our investigation of the scope of this transformation began by varying the carboxylic acid (Scheme 5). In general, acids bearing alkyl, aryl and alkenyl substituents all furnished the desired amides (**9 a**–**9 e**) in nearly quantitative yields. Due to its lower nucleophilicity, productive reaction of 5‐(trifluoromethyl)picolinic acid with Nms‐amide **1 c** required elevated temperature (100 °C) to smoothly provide amide **9 f**. In addition, a pyrazolo‐pyridine‐based acid provided the desired amide (**9 h**) in excellent yield, and a base‐labile TMS‐alkyne (**9 g**) was also well tolerated. Notably, Boc‐Hyp‐OH was also amenable to our synthetic protocol, delivering proline‐derived amide **9 i** in high yield with complete stereochemical fidelity at the α‐position of the carbonyl.^[36]^ Accordingly, with Boc‐Pro‐OH as acid partner, the product **9 j** was obtained without signs of racemization (>99 % ee). To underscore the utility of our protocol for amide formation on complex carboxylic acids, a series of pharmaceutically relevant compounds (Febuxostat, Probenecid, Ciprofloxacin, Sulindac, Dehydrocholic acid and Indomethacin) were directly subjected to the reaction conditions in the presence of **1 c**, affording the products **9 k**–**9 n, 9 q** and **9 r** in good to excellent yields. In addition, it was shown that dipeptides also constitute suitable substrates (**9 o**, **9 p**), allowing potential application in peptide synthesis. Importantly, we successfully scaled up the synthesis of indomethacin‐derived amide **9 r** to yield over 1 g of product in a single batch.

**Scheme 5:**
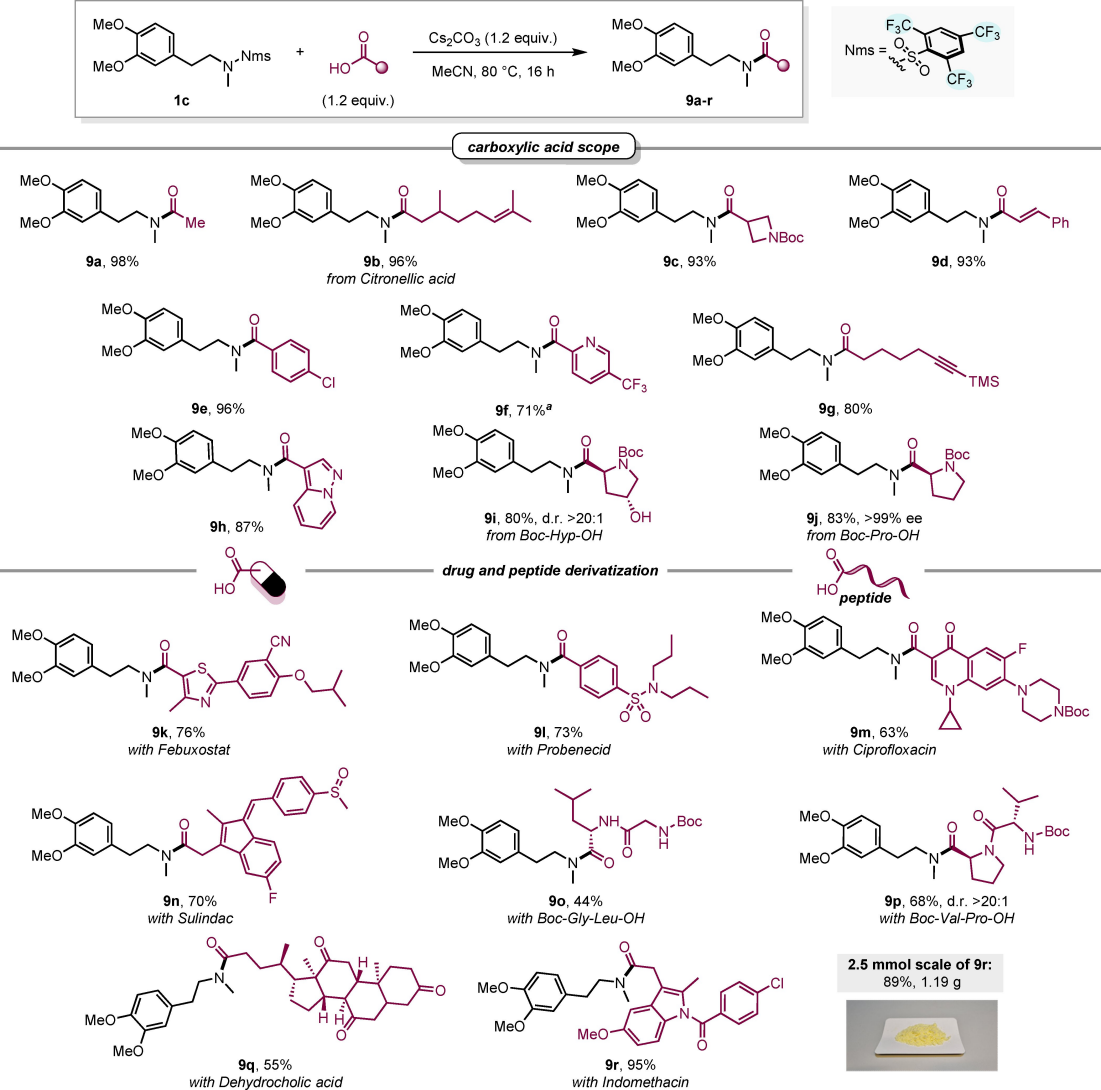
Scope of carboxylic acids. Reaction conditions: sulfonamide (0.1 mmol), carboxylic acid (0.12 mmol), Cs_2_CO_3_ (0.12 mmol), MeCN (1 mL), 80 °C, 16 h, air atmosphere. (a) reaction performed at 100 °C with 2 equiv. of Cs_2_CO_3_ and carboxylic acid.

We next turned our attention to the scope of sulfonamides, starting with secondary sulfonamides (Scheme 6). Interestingly, when Nms‐amide **1 b** (for structure, see Scheme 2) was treated with *p*‐chlorobenzoic acid under reaction conditions employing cesium carbonate, symmetrical urea (**4**, see Scheme 2) was observed as the sole product. Consequently, we opted to change the base and identified *N*‐methylimidazole (NMI) as the ideal substitute.^[37]^ To our delight, under these conditions, the desired amide (**12 a**) was formed in 93 % yield, as the sole product. Moreover, other secondary sulfonamides bearing *tert*‐butyl, benzyl and unprotected indole moieties were well tolerated, delivering amides **12 b**–**12 d** in good yields. Additionally, Nms‐protected l‐isoleucine also delivered the amino‐acid derivative **12 e**, albeit in modest yield.

**Scheme 6:**
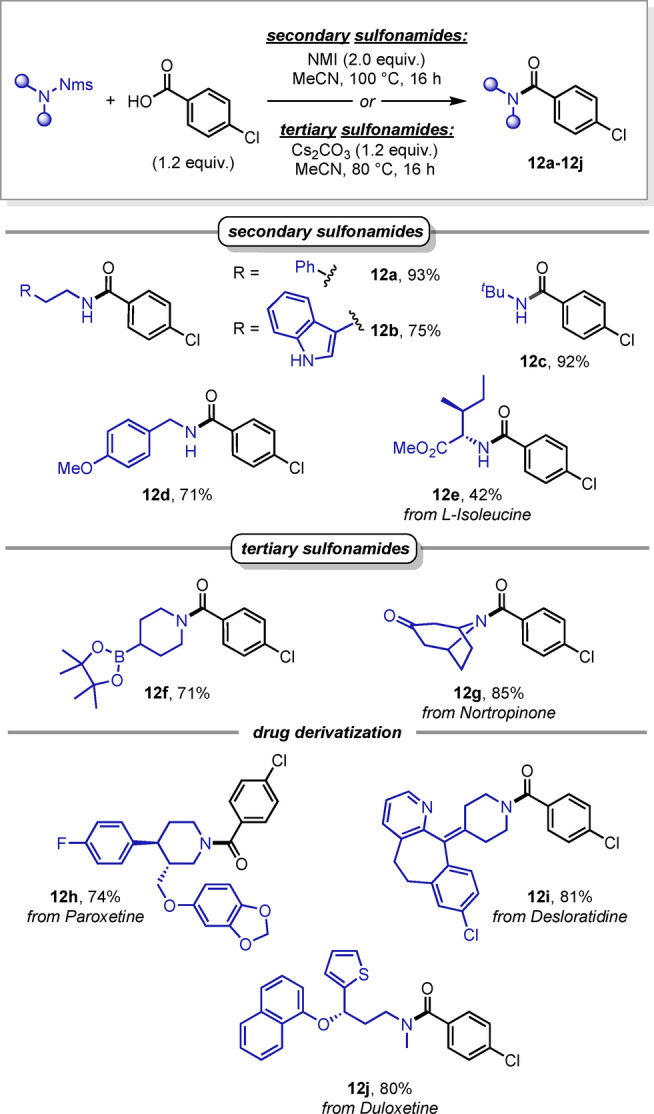
Scope of secondary and tertiary sulfonamides.

Next, tertiary sulfonamides were studied in more detail, and we found that substrates bearing an aliphatic ketone or a Bpin moiety, both potentially reactive functional groups under basic reaction conditions, delivered the corresponding amides **12 f** and **12 g** in good yields. Finally, Nms‐amides derived from the drugs Paroxetine, Desloratidine and Duloxetine could be readily transformed to the desired tertiary amides with high efficiency (**12 h**–**12 j**), underscoring the potential of late‐stage deprotective functionalization for complex protected amines.

Motivated by these findings, we aimed to evaluate the efficacy of our reaction protocol in comparison to both traditional and contemporary approaches for transforming drug amines into their respective amides through coupling with carboxylic acids. Amide coupling stands as a prevalent technique in medicinal chemistry, representing one of the most frequently utilized reactions in this domain,^[5]^ and serves as an attractive late‐stage strategy for generating potentially novel and more potent analogs. Taking a closer look at Desloratadine and Paroxetine, which we compared to previously tested amine‐to‐carboxylic‐acid couplings,[^19^, ^38^, ^39^, ^40^] we found that our deprotecitve amidation performed significantly better with all tested substrates (Scheme 7A, **13 a**–**13 e**).

**Scheme 7:**
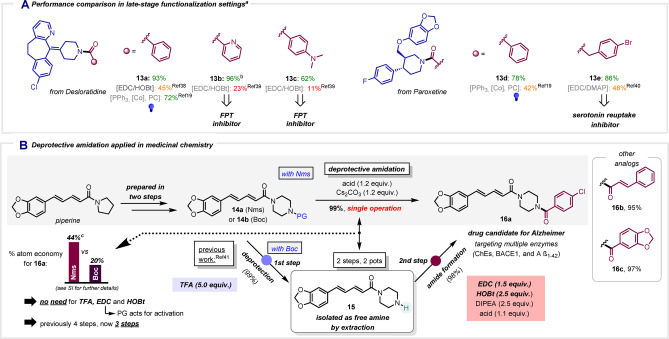
A) Performance comparison of the deprotective amidation compared to other amide couplings in drug derivatization. The reference couplings are between amine and carboxylic acid. B) Synthesis of Alzheimer drug candidates by deprotective amidation. (a) see Scheme 5 for reaction details. (b) reaction conducted at 100 °C. FPT=farnesyl protein transferase. (c) calculation for the reaction performed with K_2_CO_3_ (yield for **16 a**: 92 %). The reaction with Cs_2_CO_3_ has an atom economy of 35%.

Of particular note is the fact that **13 b** and **13 c** were synthesized in good to excellent yields (62 % and 96 %, respectively). This stands in stark contrast to the very low yields (11 % and 23 %, respectively) previously achieved in the synthesis of these potential farnesyl protein transferase inhibitors.^[39]^ Overall, these good performances underline the suitability and, in the examined cases, superiority of our protocol for late‐stage functionalization.

Our final goal was to apply the deprotective functionalization strategy in a multi‐step drug‐discovery sequence. Therefore, we sought to tackle the synthesis of **16 a** and its analogs (**16 b** and **16 c**), all of which have recently shown promising potential as multi‐targeting enzyme candidates for Alzheimer's drug development, with **16 a** being the most active (Scheme 7B).^[41]^ Compound **14 a** could be readily prepared in two steps from commercially available piperine, and, with **14 a** in hand and treated under our standard reaction conditions, we were pleased to obtain an excellent yield of 99 % of the desired Alzheimer's drug **16 a**, but also its analogs (**16 b** and **16 c**). Previous attempts to synthesize **16 a** required a Boc group to prepare **14 b**, which had to be cleaved prior to amide coupling and required isolation of the intermediate amine (by extraction→a total of 2 steps with 2 pots).^[41]^ Our method not only simplifies the synthesis by reducing the number of reaction steps from 4 to 3, but also improves atom economy (more than two‐fold, when using K_2_CO_3_ as base) by utilizing the intrinsic reactivity of the Nms group for carboxylate activation.^[42]^ This eliminates the necessity for a deprotection agent (TFA) and coupling reagents (EDC and HOBt), only requiring acid and base—both reagents essential for amide coupling in any case.

In summary, based on a fortuitous discovery, we have discovered and developed a new deprotection mode that directly converts suitably protected amines (Nms‐amides) into carboxamides in a single step. This protocol is notable for its simplicity, providing numerous amides in a single operation by coupling with free carboxylic acids. Importantly, evaluation of other benchmark sulfonamide protecting groups (e.g. Ns‐ or ^f^Xs‐amides) highlighted the unique proclivity of Nms‐amides to enable “deprotective functionalization” reactivity, in what is an intriguing demonstration of the latent potential of Nms‐amides to serve as useful functional levers for synthesis. This unique reactivity could also be demonstrated in the context of drug discovery, making it an extremely useful method for achieving high reaction performance and facilitating late‐stage functionalization.

## Conflict of interests

The authors declare no conflict of interest.

## Supporting information

As a service to our authors and readers, this journal provides supporting information supplied by the authors. Such materials are peer reviewed and may be re‐organized for online delivery, but are not copy‐edited or typeset. Technical support issues arising from supporting information (other than missing files) should be addressed to the authors.

Supporting Information

## Data Availability

The data that support the findings of this study are available in the supplementary material of this article.
