# Supplementary material for: Deprotective Functionalization: A Direct Conversion of Nms‐Amides to Carboxamides Using Carboxylic Acids
Source: Angew Chem Int Ed Engl. 2024 Mar 19;63(19):e202318304. doi: 10.1002/anie.202318304 (PMC11497274; doi:10.1002/anie.202318304)
Supplement: Supplementary file 1 — Supporting Information [file ANIE-63-e202318304-s001.pdf]

Angewandte Chemie
Eine Zeitschrift der Gesellschaft Deutscher Chemiker

Supporting Information

Deprotective Functionalization: A Direct Conversion of Nms-Amides to Carboxamides Using Carboxylic Acids

*P. Spieß, J. Brześkiewicz, R. Meyrelles, D. Just, N. Maulide**

Deprotective Functionalization: a Direct Conversion of Nms-Amides to Carboxamides Using Carboxylic Acids

Philipp Spieß, Jakub Brześkiewicz, Ricardo Meyrelles, David Just and Nuno Maulide*

Institute of Organic Chemistry, University of Vienna, Währinger Straße 38, 1090 Vienna (Austria)
E-Mail: nuno.maulide@univie.ac.at, Homepage: http://maulide.univie.ac.at

Table of Contents

1. General information 2
2. Urea formation of Nms-amides with carbonates 3
3. Reaction optimization 6
 3.1. Solvent 6
 3.2. Base 6
 3.3. Temperature 7
 3.4. Other deviations 7
 3.5 Testing of Ns-amide and ^{f}Xs-amide 8
4. Experimental 9
4.1. Preparation of starting materials 9
4.2. Deprotective amidation 12
  4.2.1 General procedures 12
  4.2.2 Characterization of amides 13
  4.2.3 Scale-up of 9r 29
  4.2.4 Failed examples 31
  4.2.5 Evidence for proposed deprotective amidation mechanism 32
5. Application 36
 5.1 Performance comparison in late-stage functionalization settings 36
 5.2 Deprotective amidation applied in medicinal chemistry 39
6. DFT calculations 43
 6.1 XYZ structures 43
7. NMR spectra 58
8. References 104

1. General information

Unless otherwise stated, all glassware was flame-dried before use and all reactions were performed under an atmosphere of argon. All solvents were distilled from appropriate drying agents prior to use. All reagents were used as received from commercial suppliers unless otherwise stated. Reaction progress was monitored by thin layer chromatography (TLC) performed on aluminum plates coated with silica gel F254 with 0.2 mm thickness. Chromatograms were visualized by fluorescence quenching with UV light at 254 nm or by staining using potassium permanganate. Flash column chromatography was performed using silica gel 60 (230-400 mesh, Merck and co.). Neat infrared spectra were recorded using a Perkin-Elmer Spectrum 100 FT-IR spectrometer. Wavenumbers (ν_{max}) are reported in cm^{-1}. Mass spectra were obtained using a Finnigan MAT 8200 (70 eV) or an Agilent 5973 (70 eV) spectrometer, using electrospray ionization (ESI). All ^{1}H NMR and ^{13}C NMR spectra were recorded using a Bruker AV-400 or AV-600 spectrometer at 300K. Chemical shifts are given in parts per million (ppm, δ), referenced to the solvent peak of $CDCl_3$ defined at δ = 7.26 ppm (^{1}H-NMR) and δ = 77.2 (^{13}C-NMR), DMSO-d_6 defined at δ = 2.50 ppm (^{1}H-NMR) and δ = 39.5 (^{13}C-NMR), CD_3OD defined at δ = 3.31 ppm (^{1}H-NMR) and δ = 49.0 (^{13}C-NMR) or Acetone-d_6 defined at δ = 2.05 ppm (^{1}H-NMR) and δ = 206.3, 29.8 (^{13}C-NMR). Coupling constants are quoted in Hz (J). ^{1}H NMR splitting patterns are designated as singlet (s), doublet (d), triplet (t), quartet (q), heptet (hept), as they appeared in the spectrum. If the appearance of a signal differs from the expected splitting pattern, the observed pattern is designated as apparent (app). Splitting patterns that could not be interpreted or easily visualized are designated as multiplet (m) or broad (br).

2,4,6-Tris(trifluoromethyl)sulfonyl chloride as well as most Nms-amides were synthesized according to the literature procedure.[1]

2. Urea formation of Nms-amides with carbonates

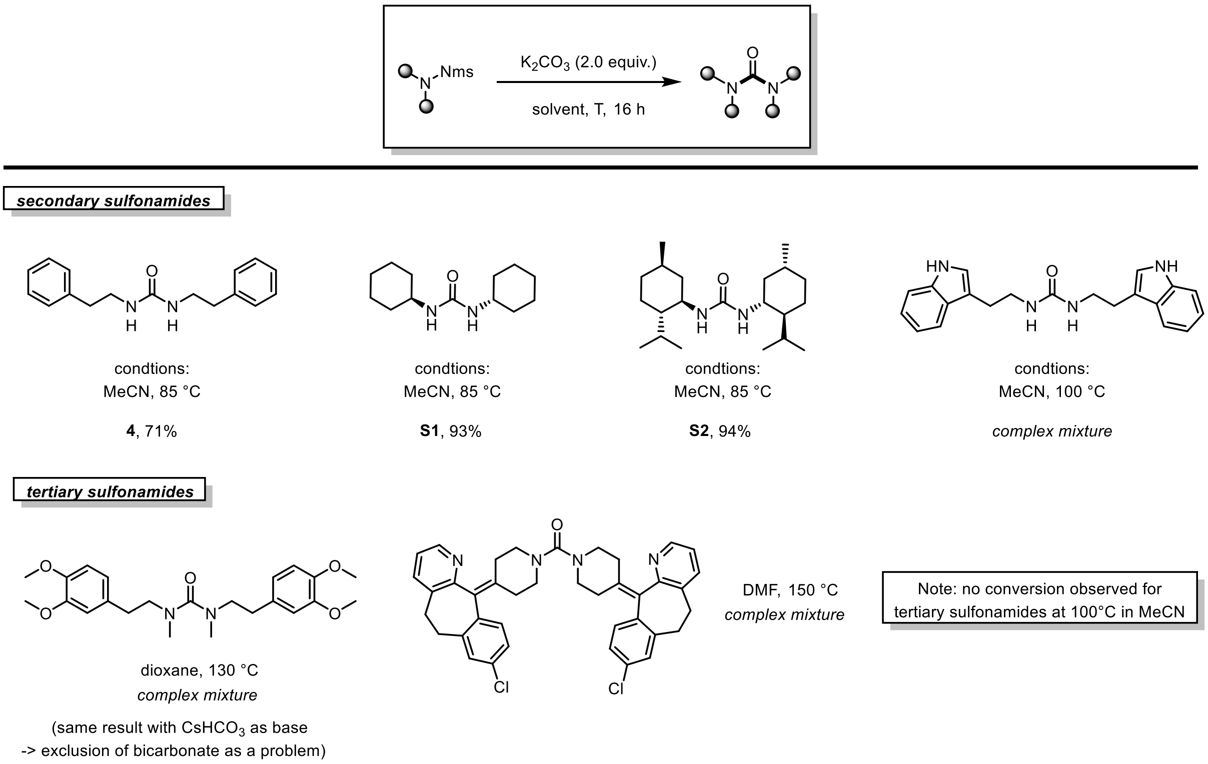

Proposed mechanism:

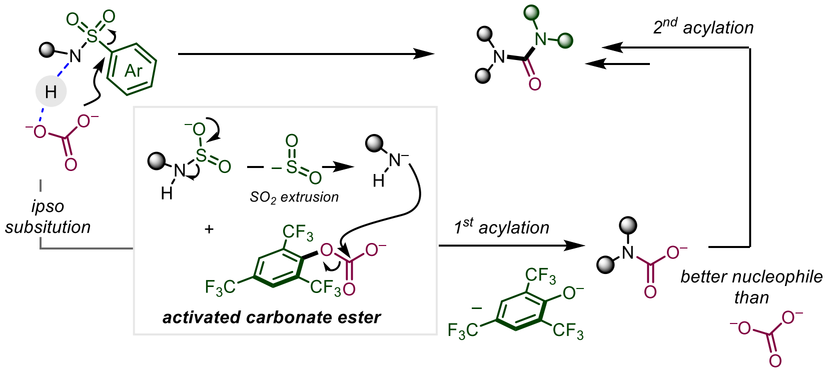

Considering that this urea formation occurs only with secondary sulfonamides (even when tertiary sulfonamides are treated with bicarbonate sources, see above result), hydrogen-bond interaction between the secondary sulfonamide and the carbonate during ipso-substitution seems necessary.

General procedure:

A vial was loaded with the corresponding sulfonamide (0.1 mmol) and K_2CO_3 (2.0 equiv.). Solvent was added (0.1 M) and the mixture was heated to the corresponding temperature. After 16 h, the crude material was passed through a Celite plug using EtOAc as eluent, the solvent was removed in vacuo and the crude material analyzed by ^{1}H NMR. Successful reactions were further purified by column chromatography.

1,3-Diphenethylurea (4)

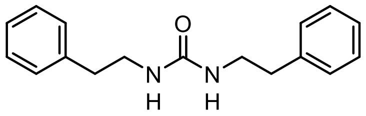

Following the general procedure in MeCN at 85 °C using *N*-phenethyl-2,4,6-tris(trifluoromethyl)benzenesulfonamide (46.5 mg, 0.1 mmol), the titled compound was obtained after column chromatography (heptane/EtOAc) as a colourless solid (9.5 mg, 35 µmol, 71%).

^{1}H NMR (400 MHz, Acetone): δ 7.30 – 7.23 (m, 4H), 7.22 – 7.14 (m, 5H), 5.85 – 5.47 (m, 1H), 3.37 (t, *J* = 7.3 Hz, 4H), 2.74 (t, *J* = 7.3 Hz, 4H) ppm.

All NMR data was in good accordance to the literature.[2]

1,3-Dicyclohexylurea (S1)

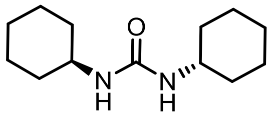

Following the general procedure in MeCN at 85 °C using *N*-cyclohexyl-2,4,6-tris(trifluoromethyl)benzenesulfonamide (88.6 mg, 0.2 mmol), the titled compound was obtained after column chromatography (heptane/EtOAc) as a colourless solid (21 mg, 93 µmol, 93%).

^{1}H NMR (400 MHz, $CDCl_3$): δ 4.20 (s, 2H), 3.46 – 3.35 (m, 2H), 1.97 – 1.82 (m, 4H), 1.72 – 1.55 (m, 4H), 1.38 – 1.24 (m, 2H), 1.20 – 0.99 (m, 6H) ppm.

All NMR data was in good accordance to the literature.[3]

1,3-Bis((1*R*,2*S*,5*R*)-2-isopropyl-5-methylcyclohexyl)urea (S2)

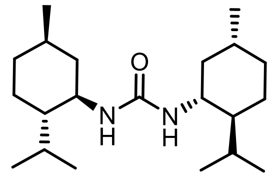

Following the general procedure in MeCN at 85 °C (76 µmol scale) using *N*-((1*R*,2*S*,5*R*)-2-isopropyl-5-methylcyclohexyl)-2,4,6-tris(trifluoromethyl)benzenesulfonamide (38.3 mg, 77 µmol), the titled compound was obtained after column chromatography (heptane/EtOAc) as a colourless solid (12 mg, 36 µmol, 93%).

^{1}H NMR (600 MHz, $CDCl_3$): δ 4.05 – 3.86 (m, 2H), 3.49 – 3.40 (m, 2H), 2.02 – 1.94 (m, 4H), 1.70 – 1.61 (m, 4H), 1.49 – 1.40 (m, 2H), 1.11 – 1.02 (m, 2H), 0.99 – 0.93 (m, 2H), 0.89 – 0.76 (m, 20H) ppm.

^{13}C NMR (151 MHz, $CDCl_3$): δ 157.43, 157.41*, 51.04, 50.96*, 49.16, 49.11*, 44.36, 44.30*, 34.77, 34.73*, 32.16, 32.15*, 26.84, 26.72*, 24.14, 24.08*, 22.34*, 21.42, 21.38*, 16.42, 16.35*. * for signals of other rotamer.

IR (neat): ν_{max} 3351, 3321, 2974, 2922, 2866, 2844, 2444, 1646, 1622, 1553, 1520, 1474, 1456, 1446, 1385, 1378, 1367, 1340, 1298, 1173, 1156, 997 cm^{-1}.

HRMS (ESI$^+$): exact mass calculated for $[M+H]^+$ $(C_{21}H_{41}ON_2)^+$ requires *m/z* 337.3219, found *m/z* 337.3212.

3. Reaction optimization

General procedure: A vial was filled with sulfonamide **1c** (53.9 mg, 0.1 mmol, 1.0 equiv.) and acetic acid. Then, the corresponding base was added, followed by addition of solvent (0.1 M) and the sealed vial was placed into preheated sand bath with the desired reaction temperature. The mixture was stirred for 16 h. After cooling down to room temperature, the crude material was passed through a pad of Celite using EtOAc as eluent. The yield was determined by ^{1}H NMR analysis using dibromomethane (7.0 µL, 17.4 mg, 0.1 mmol) as internal standard.

3.1. Solvent

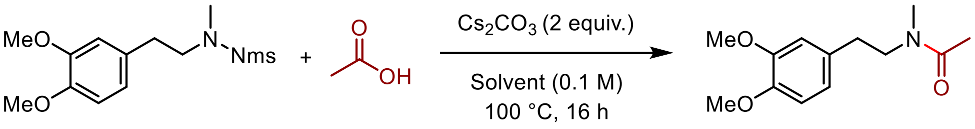

Entry	Solvent	Yield (%)[a]
1	DMF	**78**
2	MeCN	**98**
3	1,4-Dioxane	**58**

3.2. Base

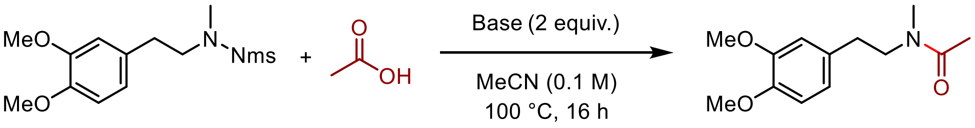

Entry	Base	Yield (%)[a, b]
1	$CsHCO_3$	**92** (6)
2	K_2CO_3	**99**
3	N-Methylimidazole	**96**
4	TEA	**90** (8)

3.3. Temperature

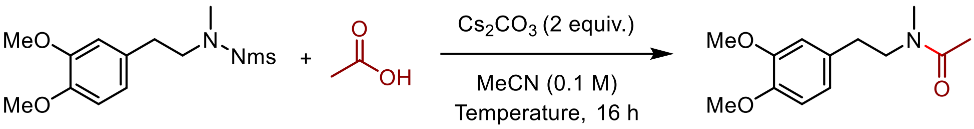

1c 9a

Entry	Temperature	Yield (%)[a, b]
1	60	**66** (31)
2	80	**99**

3.4. Other deviations

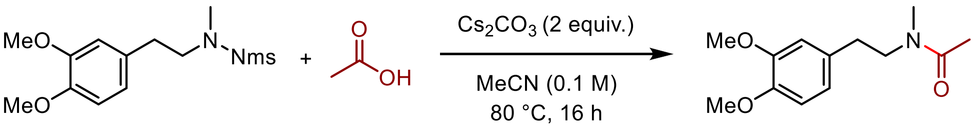

1c 9a

Entry	Other deviations	Yield (%)a
1	2 equiv. K_2CO_3	**65%** (32)[b]
2	2 equiv. K_2CO_3 and 1.2 equiv. carboxylic acid	**66%** (30)[b]
3	1.2 equiv. carboxylic acid	**99%**
**4**	**1.2 equiv. Cs_2CO_3 and 1.2 equiv. carboxylic acid**	**99% (98%)**[c]

3.5 Testing of Ns-amide and ^{f}Xs-amide

General procedure:

A vial was filled with corresponding sulfonamide (0.1 mmol, 1.0 equiv.) and acetic acid (6.9 µL, 0.12 mmol, 1.2 equiv.). Cs_2CO_3 (39.1 mg, 1.2 mmol, 1.2 equiv.) was added, followed by MeCN (1 mL). The vial was sealed and the mixture was stirred for 16 h at 80 °C. The crude material was allowed to cool down to room temperature and was then passed through a pad of Celite using EtOAc as eluent. The yield was determined by ^{1}H NMR analysis using dibromomethane (7.0 µL, 17.4 mg, 0.1 mmol) as internal standard.

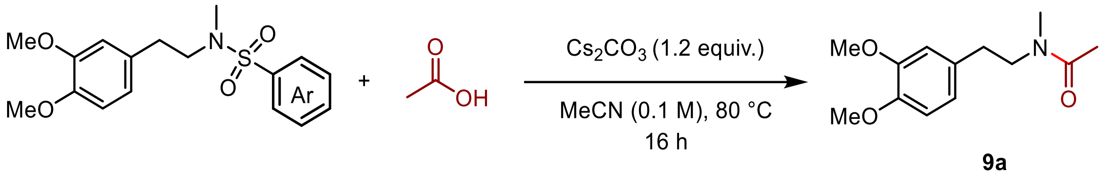

9a

**Ar**	**4-NO$_2$ (10)**	**2,6-CF$_3$ (11)**	**2,4,6-CF$_3$ (1c)**
**Yield (%)**	0 (99% SM)	0 (99% SM)	98[a]

4. Experimental

4.1. Preparation of starting materials

2,6-bis(trifluoromethyl)benzenesulfonyl chloride (S3)

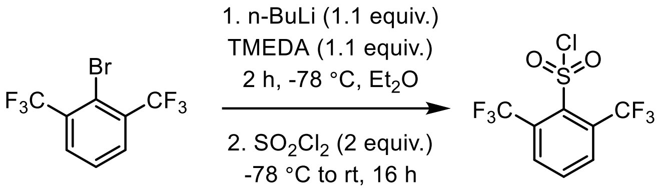

To a stirred solution of *N,N,N′,N′*-tetramethylethylenediamine (3.32 mL, 20.0 mmol, 1.1 equiv.) in diethyl ether (20 mL) was added *n*-butyllithium (1.60 M in hexanes, 13.80 mL, 22.0 mmol, 1.1 equiv.) at −78 °C. The resulting mixture was added to a stirred solution of 2-bromo-1,3-bis(trifluoromethyl)benzene (5.860 g, 20.0 mmol, 1 equiv.) in diethyl ether (80 mL) at −78 °C. After stirring for 2 h, freshly distilled SO_2Cl_2 (3.24 mL, 40 mmol, 2.0 equiv.) was added and the reaction was stirred while warming to room temperature (20-25 °C) for 16 h. Et_2O (80 mL) was added to the crude mixture. This organic phase was washed with 1M HCl (50 mL) and then with a sat. aq. solution of Na_2CO_3 (2 x 50 mL). The organic phase was dried over $mgSO_4$ and the solvent was removed under vacuum. Then, the resulting yellow solid was washed with cold pentane (0 °C, 3 x 4 mL) and the solid was dried under vacuum. 2,6-Bis(trifluoromethyl)benzenesulfonyl chloride (30% yield, 1.892 g, 6.04 mmol) was obtained as a colourless solid without need for further purification.

^{1}H NMR (400 MHz, $CDCl_3$): δ 8.22 (d, *J* = 8.1 Hz, 2H), 8.01 (t, *J* = 8.1 Hz, 1H) ppm.

^{13}C NMR (101 MHz, $CDCl_3$): δ 142.6, 135.4, 133.0 (q, *J* = 6.4 Hz), 132.1 (q, *J* = 34.0 Hz), 122.2 (q, *J* = 274.6 Hz) ppm.

^{19}F NMR (376 MHz, $CDCl_3$): δ -54.2 ppm.

All NMR data were in accordance with the literature.[4]

General procedure for preparation of sulfonamides:

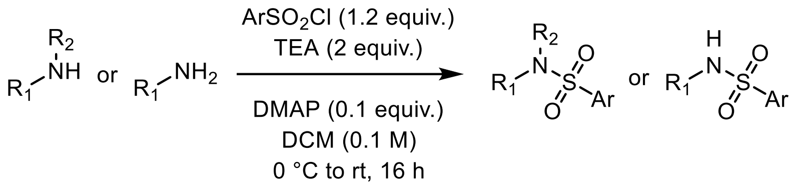

The corresponding amine (1.0 equiv.) was dissolved in DCM (0.1 M). DMAP (4-dimethylaminopyridine, 10 mol%) and Et_3N (2.0 equiv. for free amines, 3.0 equiv. for hydrochloride salts of amines) were added at room temperature. Then, the mixture was cooled to 0 °C and the arenesulfonyl chloride (1.2 equiv.) was added in one portion. The reaction was allowed to slowly warm to room temperature over 16 h. Then, the solvent was removed under vacuum and the residue was purified by column chromatography (silica gel, heptane/EtOAc).

N-Phenethyl-2,4,6-tris(trifluoromethyl)benzenesulfonamide (S4)

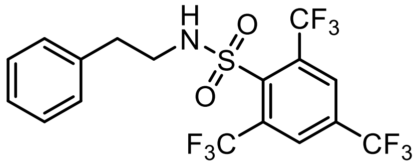

Following the general procedure (3 mmol scale), the titled compound was obtained as a colourless solid (1.379 g, 2.96 mmol, 99%).

^{1}H NMR (400 MHz, $CDCl_3$): δ 8.23 (s, 2H), 7.26 – 7.20 (m, 2H), 7.19 – 7.14 (m, 1H), 7.12 – 7.08 (m, 2H), 4.91 (t, *J* = 5.9 Hz, 1H), 3.47 (q, *J* = 6.6 Hz, 2H), 2.84 (t, *J* = 6.9 Hz, 2H) ppm.

^{13}C NMR (101 MHz, $CDCl_3$): δ 145.5, 137.2, 134.1 (q, *J* = 35.2 Hz), 132.5 (q, *J* = 33.5 Hz), 129.4 (m), 129.0, 128.9, 127.2, 122.2 (q, *J* = 275.4 Hz), 122.0 (q, *J* = 273.5 Hz), 45.1, 36.6 ppm.

^{19}F NMR (376 MHz, $CDCl_3$): δ -54.74 (6F), -63.61 (3F) ppm.

IR (neat): ν_{max} 3359, 1271, 1171, 1132, 1079, 706, 686, 465 cm^{-1}.

HRMS (ESI$^+$): exact mass calculated for $[M+Na]^+$ $(C_{17}H_{12}F_9NO_2SNa)^+$ requires *m/z* 488.0337, found *m/z* 488.0337.

N-(3,4-Dimethoxyphenethyl)-N-methyl-2,6-bis(trifluoromethyl)benzenesulfonamide (S5)

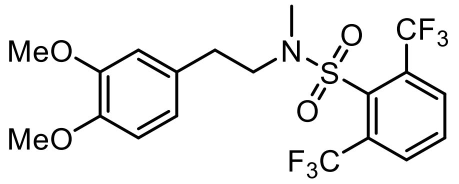

Following the general procedure (1 mmol scale), the titled compound was obtained as a colourless oil (466 mg, 0.99 mmol, 99%).

^{1}H NMR (400 MHz, $CDCl_3$): δ 8.05 (d, *J* = 8.0 Hz, 2H), 7.77 (t, *J* = 8.0 Hz, 1H), 6.79 (d, *J* = 8.6 Hz, 1H), 6.77 – 6.72 (m, 2H), 3.87 (s, 3H), 3.85 (s, 3H), 3.43 (t, *J* = 8.1 Hz, 2H), 2.94 (t, *J* = 8.1 Hz, 2H), 2.80 (s, 3H) ppm.

^{13}C NMR (101 MHz, $CDCl_3$): δ 149.2, 147.9, 140.9, 132.4 (q, *J* = 6.4 Hz), 132.1, 131.9 (q, *J* = 33.0 Hz), 130.9, 122.8 (q, *J* = 275.0 Hz), 120.9, 112.2, 111.5, 56.0 (2C), 52.9, 35.0, 34.3 ppm.

^{19}F NMR (376 MHz, $CDCl_3$): δ -55.45 ppm.

IR (neat): ν_{max} 2940, 1515, 1351, 1283, 1140, 1106, 1027, 723, 589 cm^{-1}.

HRMS (ESI$^+$): exact mass calculated for $[M+Na]^+$ $(C_{19}H_{19}F_6NO_4SNa)^+$ requires *m/z* 494.0831, found *m/z* 494.0841.

***N*-(3,4-Dimethoxyphenethyl)-*N*-methyl-4-nitrobenzenesulfonamide (S6)**

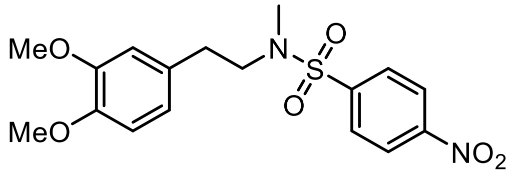

Following the general procedure (3 mmol scale), the titled compound was obtained as a yellow solid (669 mg, 1.76 mmol, 59%).

^{1}H NMR (400 MHz, CDCl$_3$): δ 8.33 (d, *J* = 8.9 Hz, 2H), 7.90 (d, *J* = 8.9 Hz, 2H), 6.78 (d, *J* = 8.7 Hz, 1H), 6.72 – 6.68 (m, 2H), 3.87 (s, 3H), 3.86 (s, 3H), 3.33 (t, *J* = 7.2 Hz, 2H), 2.83 (t, *J* = 7.7 Hz, 2H), 2.81 (s, 3H) ppm.

^{13}C NMR (101 MHz, CDCl$_3$): δ 150.1, 149.2, 148.1, 144.2, 130.4, 128.5, 124.4, 120.9, 112.1, 111.5, 56.0 (2C), 52.1, 35.2, 34.6 ppm.

IR (neat): ν_{max} 2928, 2839, 1527, 1346, 1233, 1146, 1022, 819, 464 cm^{-1}.

HRMS (ESI$^+$): exact mass calculated for $[M+Na]^+$ $(C_{17}H_{20}N_2O_6SNa)^+$ requires *m/z* 403.0934, found *m/z* 403.0936.

Synthesis of *N*-Boc protected Ciprofloxacin:

7-(4-(Tert-butoxycarbonyl)piperazin-1-yl)-1-cyclopropyl-6-fluoro-4-oxo-1,4-dihydroquinoline-3-carboxylic acid (S7)

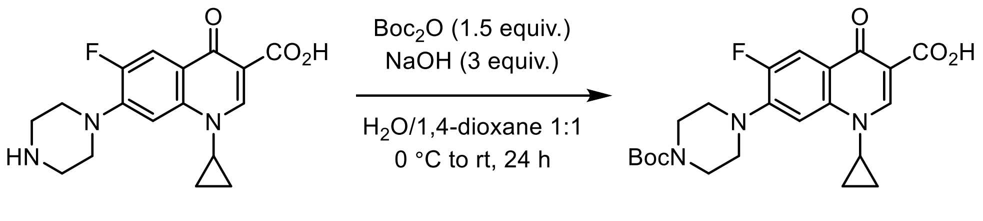

A 100 mL round-bottom flask was loaded with Ciprofloxacin (1.014, 3 mmol, 1 equiv.), water (20 mL) and 1,4-dioxane (20 mL). A solution of NaOH (360 mg, 9 mmol, 3 equiv.) in water (5 mL) was added to the flask and the reaction mixture was cooled down to 0 °C. Subsequently, Boc$_2$O (982 mg, 4.5 mmol, 1.5 equiv.) was added slowly and the reaction was continued for 16 h at room temperature (25 °C). Upon completion, the solution was acidified with 1M HCl to pH 7, concentrated, washed with cold diethyl ether (2 x 30 mL) and the solid was dried under vacuum. N-Boc protected Ciprofloxacin (84% yield, 1.080 g, 2.50 mmol) was obtained as a colourless solid without need for further purification.

^{1}H NMR (400 MHz, CDCl$_3$): δ 14.95 (s, 1H), 8.78 (s, 1H), 8.05 (d, *J* = 12.9 Hz, 1H), 7.37 (d, *J* = 7.1 Hz, 1H), 3.73 − 3.62 (m, 4H), 3.53 (tt, *J* = 7.3, 4.0 Hz, 1H), 3.34 − 3.25 (m, 4H), 1.50 (s, 9H), 1.43 − 1.37 (m, 2H), 1.24 − 1.17 (m, 2H) ppm.

^{13}C NMR (101 MHz, CDCl$_3$): δ 167.1, 154.7, 153.1, 147.7, 113.0, 112.7, 108.5, 105.1,80.5, 35.4, 28.6, 8.4. ppm.

All NMR data were in accordance with the literature.[5]

4.2. Deprotective amidation

4.2.1 General procedures

General procedure (GP1): tertiary sulfonamides

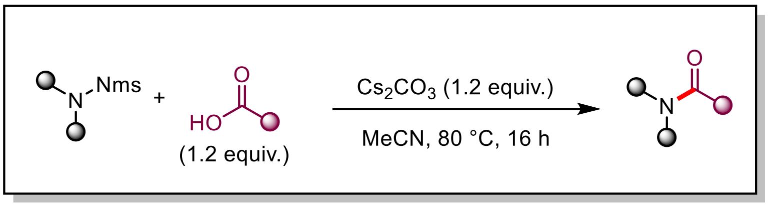

A vial was filled with sulfonamide (1.0 equiv.) and carboxylic acid (1.2 equiv.). Cs_2CO_3 (1.2 equiv.) was added, followed by MeCN (0.1 M). The vial was sealed and placed into a sand bath, and the reaction mixture was stirred for 16 h at 80 °C. The crude reaction mixture was subsequently passed through a pad of Celite using EtOAc as eluent. After evaporation of the solvent, the crude material was subjected to column chromatography (silica gel, heptane/EtOAc) to obtain the desired amide products.

Note: The reaction was carried out in an air atmosphere.

General procedure (GP2): secondary sulfonamides

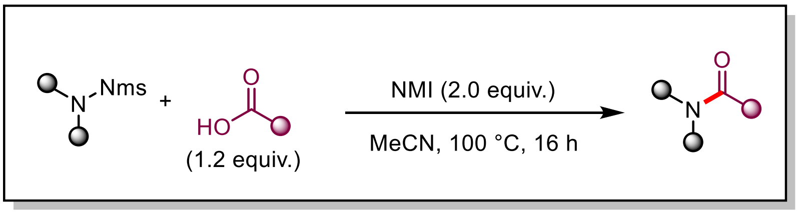

A vial was filled with sulfonamide (1.0 equiv.) and carboxylic acid (1.2 equiv.). *N*-Methylimidazole (1.2 equiv.) was added, followed by MeCN (0.1 M). The vial was sealed and placed into a sand bath, and the reaction mixture was stirred for 16 h at 100 °C. The crude material was evaporated and subjected to column chromatography (silica gel, heptane/EtOAc) to obtain the desired amide products.

Note: The reaction was carried out in an air atmosphere.

4.2.2 Characterization of amides

***N*-(3,4-Dimethoxyphenethyl)-*N*-methylacetamide (9a)**

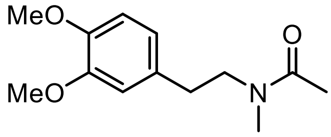

Following the GP1 (0.1 mmol scale), the titled compound was obtained as a colourless oil (23.2 mg, 98 µmol, 98%). The desired amide was obtained as a mixture of two rotamers in a ratio of around 1.1:1. NMR peaks in ^{1}H NMR corresponding exclusively to the minor rotamer are indicated with an asterisk.

^{1}H NMR (400 MHz, $CDCl_3$): δ 6.82 – 6.72 (m, 2H), 6.71 – 6.63 (m, 1H), 3.90 – 3.82 (m, 6H), 3.55 (t, *J* = 7.4 Hz, 1H), 3.48 (t, *J* = 7.1 Hz, 1H*), 2.93 (s, 1.5H*), 2.88 (s, 1.5H), 2.78 (t, *J* = 7.3 Hz, 2H), 2.06 (s, 1.5H), 1.85 (s, 1.5H*) ppm.

^{13}C NMR (101 MHz, $CDCl_3$): δ 170.7, 170.5, 149.2, 149.0, 148.0, 147.7, 131.9, 130.9, 120.9, 120.8, 112.2, 112.0, 111.6, 111.4, 56.1, 56.0 (3C), 52.8, 49.9, 37.0, 34.5, 33.5 (2C), 22.1, 21.2 ppm.

IR (neat): ν_{max} 2936, 1624, 1514, 1463, 1400, 1261, 1235, 1141, 1025 cm^{-1}.

HRMS (ESI$^+$): exact mass calculated for $[M+Na]^+$ $(C_{13}H_{19}NO_3Na)^+$ requires *m/z* 260.1257, found *m/z* 260.1254.

***N*-(3,4-Dimethoxyphenethyl)-*N*,3,7-trimethyloct-6-enamide (9b)**

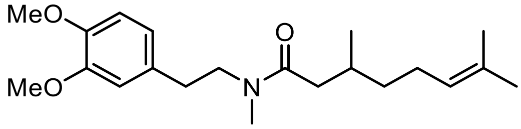

Following the GP1 (0.1 mmol scale), the titled compound was obtained as a colourless oil (33.3 mg, 96 µmol, 96%). The desired amide was obtained as a mixture of two rotamers in a ratio of around 1.4:1. NMR peaks in ^{1}H NMR corresponding exclusively to the minor rotamer are indicated with an asterisk.

^{1}H NMR (400 MHz, $CDCl_3$): δ 6.82 – 6.63 (m, 3H), 5.08 (app q, *J* = 7.1 Hz, 1H), 3.88 – 3.83 (m, 6H), 3.57 (t, *J* = 7.4 Hz, 1.2H), 3.49 (t, *J* = 7.4 Hz, 0.8H*), 2.94 (s, 1.3H*), 2.90 (s, 1.7H), 2.77 (t, *J* = 7.7 Hz, 2H), 2.28 (dd, *J* = 14.4, 5.3 Hz, 0.5H), 2.13 – 1.87 (m, 4.5H), 1.69 – 1.65 (m, 3H), 1.62 – 1.57 (m, 3H), 1.44 – 1.06 (m, 2H), 0.93 (d, *J* = 6.4 Hz, 1.7H), 0.86 (d, *J* = 6.3 Hz, 1.3H*) ppm.

^{13}C NMR (101 MHz, $CDCl_3$): δ 172.7, 172.5, 149.3, 149.0, 148.1, 147.6, 132.0, 131.5, 131.4, 131.0, 124.6 (2C), 120.9, 120.8, 112.2, 112.1, 111.7, 111.37, 56.1, 56.0 (3C), 52.0, 50.2, 41.0, 40.30, 37.3 (2C), 36.4, 34.7, 33.7, 33.5, 30.1, 30.1, 25.8, 25.7, 25.7, 19.9 (2C), 17.8 (2C) ppm.

IR (neat): ν_{max} 2924, 2554, 1638, 1515, 1452, 1262, 1235, 1156, 1028 cm^{-1}.

HRMS (ESI$^+$): exact mass calculated for $[M+Na]^+$ $(C_{21}H_{33}NO_3Na)^+$ requires *m/z* 370.2353, found *m/z* 370.2355.

tert-Butyl 3-((3,4-dimethoxyphenethyl)(methyl)carbamoyl)azetidine-1-carboxylate (9c)

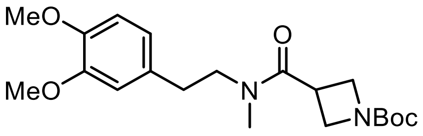

Following the GP1 (0.1 mmol scale), the titled compound was obtained as a colourless oil (35.2 mg, 93 µmol, 93%). The desired amide was obtained as a mixture of two rotamers in a ratio of around 1.7:1. NMR peaks in ^{1}H NMR corresponding exclusively to the minor rotamer are indicated with an asterisk.

^{1}H NMR (400 MHz, CDCl$_3$): δ 6.82 – 6.60 (m, 3H), 4.23 – 4.01 (m, 3H), 3.93 – 3.81 (m, 7H), 3.58 (t, *J* = 6.6 Hz, 1.2H), 3.48 – 3.39 (m, 0.6H), 3.33 (t, *J* = 7.1 Hz, 0.8H*), 3.17 – 3.09 (m, 0.4H*), 2.99 (s, 1.1H*), 2.83 – 2.70 (m, 3.9H), 1.43 (s, 5.2H), 1.41 (s, 3.8H*) ppm.

^{13}C NMR (101 MHz, CDCl$_3$): δ 171.4, 171.0, 156.3 (2C), 149.3, 149.1, 148.2, 147.8, 131.5, 130.5, 120.8 (2C), 112.1, 112.0, 111.7, 111.4, 79.8, 79.7, 56.1 (2C), 56.0 (2C), 51.6, 51.4 (br, 2C), 50.3, 35.5, 34.5, 33.7, 33.4, 31.5, 30.5, 28.5 (2C) ppm.

IR (neat): ν_{max} 2971, 2836, 1695, 1639, 1515, 1390, 1261, 1131, 764 cm^{-1}.

HRMS (ESI$^+$): exact mass calculated for [M+Na]$^+$ ($C_{20}H_{30}N_2O_5Na$)$^+$ requires *m/z* 401.2047, found *m/z* 401.2047.

***N*-(3,4-Dimethoxyphenethyl)-*N*-methylcinnamamide (9d)**

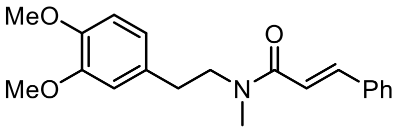

Following the GP1 (0.1 mmol scale), the titled compound was obtained as a colourless oil (30.2 mg, 93 µmol, 93%). The desired amide was obtained as a mixture of two rotamers in a ratio of around 1.2:1. NMR peaks in ^{1}H NMR corresponding exclusively to the minor rotamer are indicated with an asterisk.

^{1}H NMR (400 MHz, CDCl$_3$): δ 7.70 (d, *J* = 15.4 Hz, 0.5H*), 7.56 – 7.47 (m, 1.5H), 7.41 – 7.31 (m, 4H), 6.89 – 6.70 (m, 3H), 6.65 (s, 0.5H), 6.54 (d, *J* = 15.4 Hz, 0.5H), 3.91 – 3.81 (m, 4.5H), 3.75 (s, 1.5H), 3.67 (app q, *J* = 7.8, 6.9 Hz, 2H), 3.05 (s, 3H), 2.86 (app q, *J* = 6.9, 6.5 Hz, 2H) ppm.

^{13}C NMR (101 MHz, CDCl$_3$): δ 167.0, 166.4, 149.3, 149.1, 148.1, 147.7, 142.7, 141.8, 135.5 (2C), 131.9, 130.9, 129.7, 129.5, 128.9, 128.8, 127.9, 127.7, 121.0, 120.8, 117.7 (2C), 112.2 (2C), 111.7, 111.4, 56.0 (4C), 52.2, 50.9, 36.5, 35.1, 34.5, 33.5 ppm.

IR (neat): ν_{max} 2933, 1647, 1600, 1513, 1451, 1260, 1233, 1026, 762 cm^{-1}.

HRMS (ESI$^+$): exact mass calculated for [M+Na]$^+$ ($C_{20}H_{23}NO_3Na$)$^+$ requires *m/z* 348.1570, found *m/z* 348.1570.

4-Chloro-*N*-(3,4-dimethoxyphenethyl)-*N*-methylbenzamide (9e)

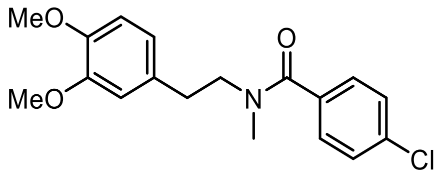

Following the GP1 (0.1 mmol scale), the titled compound was obtained as a colourless oil (32.1 mg, 96 µmol, 96%). The desired amide was obtained as a mixture of two rotamers in a ratio of around 1.6:1. NMR peaks in ^{1}H NMR corresponding exclusively to the minor rotamer are indicated with an asterisk.

^{1}H NMR (400 MHz, CD_3OD): δ 7.44 (d, *J* = 8.2 Hz, 0.8H*), 7.33 – 7.27 (m, 2H), 6.95 – 6.87 (m, 2H), 6.86 – 6.80 (m, 1H), 6.54 (d, *J* = 9.1 Hz, 0.6H), 6.45 (s, 0.6H), 3.85 – 3.79 (m, 4.2H), 3.75 (t, *J* = 7.2 Hz, 0.8H*), 3.68 (s, 1.8H), 3.54 (t, *J* = 6.4 Hz, 1.2H), 3.13 (s, 1.8H), 2.93 (t, *J* = 7.3 Hz, 0.8H*), 2.86 (s, 1.2H*), 2.74 (t, *J* = 6.4 Hz, 1.2H) ppm.

^{13}C NMR (101 MHz, CD_3OD): δ 173.2, 172.4, 150.6, 150.6, 149.4, 149.3, 136.8, 136.3, 136.2, 135.9, 133.0, 132.3, 129.8, 129.6, 129.4 (2C), 122.4, 114.0, 113.6, 113.1 (2C), 56.6 (2C), 56.5, 56.2, 54.4, 50.4, 38.6, 34.6, 33.6, 33.5 ppm.

IR (neat): ν_{max} 2920, 1626, 1514, 1400, 1260, 1145, 1026, 838, 756 cm^{-1}.

HRMS (ESI$^+$): exact mass calculated for $[M+Na]^+$ $(C_{18}H_{20}ClNO_3Na)^+$ requires *m/z* 356.1024, found *m/z* 356.1025.

***N*-(3,4-Dimethoxyphenethyl)-*N*-methyl-5-(trifluoromethyl)picolinamide (9f)**

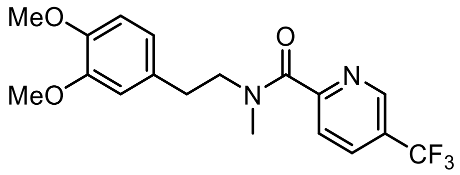

Following the slightly modified GP1 (0.1 mmol scale). The reaction was performed with 2 equiv. of Cs_2CO_3 and 2 equiv. of carboxylic acid at 100 °C. The titled compound was obtained as a colourless oil (26.2 mg, 71 µmol, 71%). The desired amide was obtained as a mixture of two rotamers in a ratio of around 1.8:1. NMR peaks corresponding exclusively to the minor rotamer are indicated with an asterisk.

^{1}H NMR (400 MHz, $CDCl_3$): δ 8.84 (app s, 0.4H*), 8.78 (app s, 0.6H), 8.03 (dd, *J* = 8.2, 1.8 Hz, 0.4H*), 7.86 (dd, *J* = 8.2, 1.8 Hz, 0.6H), 7.72 (d, *J* = 8.2 Hz, 0.4H*), 7.22 (d, *J* = 8.2 Hz, 0.6H), 6.85 – 6.81 (m, 1H), 6.70 (d, *J* = 8.1 Hz, 0.7H), 6.52 (dd, *J* = 8.1, 2.0 Hz, 0.7H), 6.35 (d, *J* = 1.9 Hz, 0.6H), 3.90 (s, 1H), 3.88 – 3.84 (m, 3H), 3.79 – 3.69 (m, 4H), 3.18 (s, 1.9H), 2.99 – 2.93 (m, 1.8H), 2.82 (t, *J* = 6.8 Hz, 1.3H) ppm.

^{13}C NMR (101 MHz, $CDCl_3$): δ 167.9, 167.4, 158.0, 157.6, 149.1 (2C), 148.0, 147.8, 145.4 (q, *J* = 4.0 Hz), 144.6 (q, *J* = 4.0 Hz), 134.4 (q, *J* = 3.4 Hz), 134.0 (q, *J* = 3.5 Hz), 131.5, 130.7, 126.9 (q, *J* = 33.1 Hz, 2C), 123.8, 123.5, 123.3 (q, *J* = 272.8 Hz, 2C), 121.1, 120.9, 112.3, 111.8, 111.5, 111.4, 56.0 (3C), 55.7, 52.8, 50.6, 37.9, 34.3, 34.1, 33.1 ppm.

^{19}F NMR (376 MHz, $CDCl_3$): δ -62.57 (s, 3F), -62.59 (s, 1.6F) ppm.

IR (neat): ν_{max} 2937, 1633, 1514, 1324, 1261, 1124, 1079, 1015, 854 cm^{-1}.

HRMS (ESI⁺): exact mass calculated for $[M+Na]^+$ $(C_{18}H_{19}F_3N_2O_3Na)^+$ requires *m/z* 391.1240, found *m/z* 391.1241.

***N*-(3,4-Dimethoxyphenethyl)-N-methyl-7-(trimethylsilyl)hept-6-ynamide (9g)**

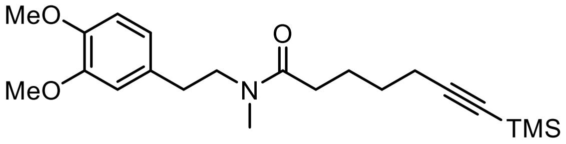

Following the GP1 (0.1 mmol scale), the titled compound was obtained as a yellow oil (30.0 mg, 80 µmol, 80%). The desired amide was obtained as a mixture of two rotamers in a ratio of around 1.2:1.

^{1}H NMR (400 MHz, CDCl$_3$): δ 6.82 – 6.74 (m, 2H), 6.73 – 6.63 (m, 1H), 3.89 – 3.83 (m, 6H), 3.61 – 3.45 (m, 2H), 2.96 – 2.85 (m, 3H), 2.84 – 2.75 (m, 2H), 2.32 – 2.06 (m, 4H), 1.77 – 1.41 (m, 4H), 0.19 – 0.07 (9H) ppm.
^{13}C NMR (101 MHz, CDCl$_3$): δ 172.9, 172.7, 149.2, 149.0, 148.1, 147.6, 131.9, 130.9, 120.9, 120.8, 112.2, 112.1, 111.6, 111.3, 107.2, 84.7, 84.7, 56.0, 56.0, 56.0, 51.9, 50.2, 36.2, 34.6, 33.7, 33.5, 33.3, 32.3, 28.5, 28.5, 24.6, 24.3, 19.8, 19.7, 0.3 ppm.
IR (neat): ν_{max} 2172, 1640, 1515, 1463, 1261, 1247, 1236, 1155, 1141, 1028, 839, 806, 760 cm^{-1}.
HRMS (ESI⁺): exact mass calculated for $[M+Na]^+$ $(C_{21}H_{33}NO_3SiNa)^+$ requires *m/z* 398.2122, found *m/z* 398.2121.

***N*-(3,4-Dimethoxyphenethyl)-*N*-methylpyrazolo[1,5-*a*]pyridine-3-carboxamide (9h)**

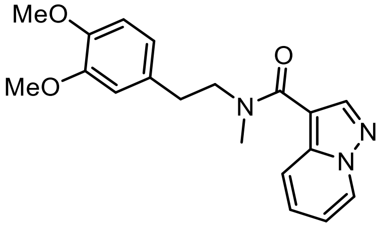

Following the GP1 (0.1 mmol scale), the titled compound was obtained as a colourless oil (29.6 mg, 87 µmol, 87%).

^{1}H NMR (400 MHz, DMSO-*d*$_6$): δ 8.74 (d, *J* = 6.9 Hz, 1H), 8.19 (br s, 1H), 7.84 (br s, 1H), 7.37 (t, *J* = 7.6 Hz, 1H), 7.02 (t, *J* = 6.5 Hz, 1H), 6.91 – 6.61 (m, 3H), 3.76 – 3.62 (m, 8H), 3.12 (s, 3H), 2.81 (t, *J* = 7.3 Hz, 2H) ppm.

^{13}C NMR (101 MHz, DMSO-*d*$_6$): δ 163.8 (br), 148.6, 147.2, 141.4 (br), 140.2, 131.3 (br), 129.0, 126.0, 120.6, 118.7, 113.6, 112.5, 111.8, 106.2, 55.4, 55.3, 50.4 (br), 32.9 (br) ppm. One alkyl carbon could not be clearly identified due to a strong broadening.

IR (neat): ν_{max} 2931, 1602, 1513, 1399, 1260, 1155, 1024, 766 cm^{-1}.

HRMS (ESI⁺): exact mass calculated for $[M+Na]^+$ $(C_{19}H_{21}N_3O_3Na)^+$ requires *m/z* 362.1475, found *m/z* 362.1476.

***tert*-Butyl (2*S*,4*R*)-2-((3,4-dimethoxyphenethyl)(methyl)carbamoyl)-4-hydroxypyrrolidine-1-carboxylate (9i)**

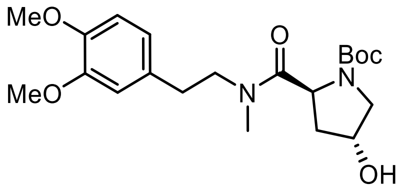

Following the GP1 (0.1 mmol scale), the titled compound was obtained as pale-yellow oil (32.8 mg, 80 µmol, 80%). The desired amide was obtained as four rotamers in a ratio of around 1.5:1.5:1:1. NMR peaks corresponding exclusively to one of the minor rotamers are indicated with an asterisk.

^{1}H NMR (400 MHz, Acetone-*d*$_6$): δ 7.00 – 6.73 (m, 3H), 4.81 – 4.68 (m, 1H), 4.48 – 4.36 (m, 1H), 4.13 – 4.08 (m, 0.9H), 3.83 – 3.62 (m, 7.1H), 3.61 – 3.46 (m, 1.5H), 3.45 – 3.34 (m, 1.5H), 3.07 (s, 0.9H), 2.99 (s, 0.6H*), 2.94 (s, 0.9H), 2.89 (t, *J* = 7.8 Hz, 0.7H), 2.81 – 2.72 (m, 1.9H), 1.90 – 1.81 (m, 1H), 1.43 (s, 3.6H*), 1.37 (s, 5.4H) ppm.

^{13}C NMR (101 MHz, Acetone-*d*$_6$): δ 173.3 (2C), 173.1, 172.7, 155.2, 155.1, 154.5 (2C), 150.7, 150.6, 150.5 (2C), 149.4, 149.2, 149.1, 149.0, 133.3, 132.9, 132.7, 132.2, 121.9 (2C), 121.8 (2C), 114.2, 114.0, 113.9, 113.8, 113.3 (2C), 113.1, 113.1, 79.5, 79.4, 79.3 (2C), 70.7, 70.6, 69.8 (2C), 56.4, 56.3 (4C), 56.2 (3C), 56.0 (4C), 55.8, 52.4 (2C), 51.1, 50.8, 40.5, 39.7, 39.7, 39.0, 36.2, 35.7 (2C), 35.5, 34.7, 34.3, 34.1, 34.0, 28.8 (2C), 28.7 (2C) ppm.

IR (neat): ν_{max} 3401, 2935, 1637, 1515, 1400, 1261, 1156, 1026, 730 cm^{-1}.

HRMS (ESI$^+$): exact mass calculated for $[M+Na]^+$ $(C_{21}H_{32}N_2O_6Na)^+$ requires *m/z* 431.2153, found *m/z* 431.2158.

Confirmation of four rotamers:

To confirm that no epimerization occurred for the chiral α-carbon center during deprotective amidation and that the four isomers were indeed the rotamers visible in NMR, the same amide was synthesized using a method for peptide couplings known not to allow epimerization.[6]

tert-Butyl (2_S_,4_R_)-2-((3,4-dimethoxyphenethyl)(methyl)carbamoyl)-4-hydroxypyrrolidine-1-carboxylate (9i)

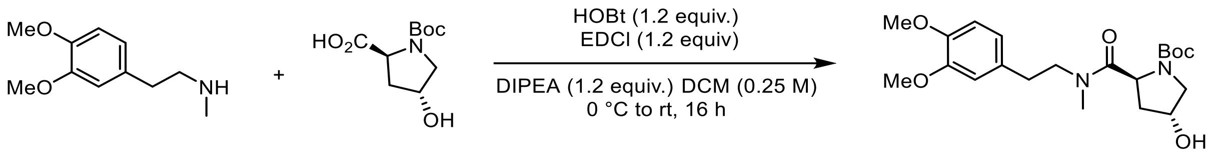

To a stirred solution of Boc-Hyp-OH (232 mg, 1 mmol, 1 equiv.) in DCM (4 mL) at 0 °C were added HOBt (162 mg, 1.2 mmol, 1.2 equiv.), EDCl (230 mg, 1.2 mmol, 1.2 equiv.) and DIPEA (194 mg, 1.5 mmol, 1.5 equiv.). After stirring for 20 minutes, 2-(3,4-dimethoxyphenyl)-*N*-methylethylamine (213 µL, 1.1 mmol, 1.1 equiv.) was added and the resulting mixture was allowed to stir at room temperature (25 °C) for 16 h. After completion of the reaction, the crude mixture was diluted with DCM (20 mL), washed with water (20mL), 1M HCl (20 mL) and brine (20 mL). The organic phase was dried over anhydrous Na_2SO_4, filtrated and concentrated *in vacuo*. The crude material was purified by column chromatography (silica gel, EtOAc). The title compound was obtained as a pale-yellow oil (340 mg, 0.83 mmol, 83% yield).

All NMR data was in complete accordance with the data obtained for amide **9i** obtained from deprotective amidation (see NMR attachment for stacked spectra).

tert-Butyl (_S_)-2-((3,4-dimethoxyphenethyl)(methyl)carbamoyl)pyrrolidine-1-carboxylate (9j)

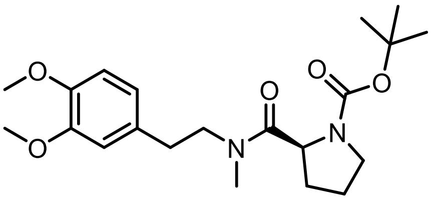

Following the GP1 (0.1 mmol scale), the titled compound was obtained as a yellow oil (32.6 mg, 83 µmol, 83%). The desired amide was obtained as four rotamers.

^{1}H NMR (400 MHz, CDCl$_3$): δ 6.85 – 6.71 (m, 3H), 4.64 – 4.46 (m, 1H), 3.90 – 3.80 (m, 6H), 3.81 – 3.28 (m, 4H), 3.03 – 2.73 (m, 5H), 2.16 – 1.65 (m, 4H), 1.47 – 1.37 (m, 9H) ppm.

^{13}C NMR (101 MHz, CDCl$_3$): δ 172.9, 172.7, 172.6, 172.2, 154.5, 153.9, 149.2, 149.1, 149.0, 148.9, 148.0, 147.8, 147.5, 131.8, 131.4, 120.8, 112.2, 112.0, 111.2, 79.4, 79.4, 79.4, 56.6, 56.5, 56.0, 55.9, 51.7, 51.7, 50.4, 47.0, 46.8, 46.8, 46.6, 35.8, 35.5, 35.0, 34.8, 34.5, 34.2, 33.3, 33.3, 31.1, 30.3, 30.1, 29.7, 29.4, 28.5, 28.5, 28.4, 24.3, 24.1, 23.6, 23.5 ppm.

IR (neat): ν_{max} 1690, 1650, 1515, 1453, 1394, 1364, 1262, 1236, 1157, 1139, 1121, 1927, 916, 764, 729 cm^{-1}.

HRMS (ESI$^+$): exact mass calculated for $[M+Na]^+$ $(C_{21}H_{32}N_2O_5Na)^+$ requires *m/z* 415.2203, found *m/z* 415.2202.

Enantiomeric excess: > 99% determined by chiral HPLC analysis: Chiralcel OD-H, *n*-heptane + 0.1%IPA/EtOH 95:5, 1 mL/min, 25 °C, detection at 210 nm, retention time (min): 20.1.

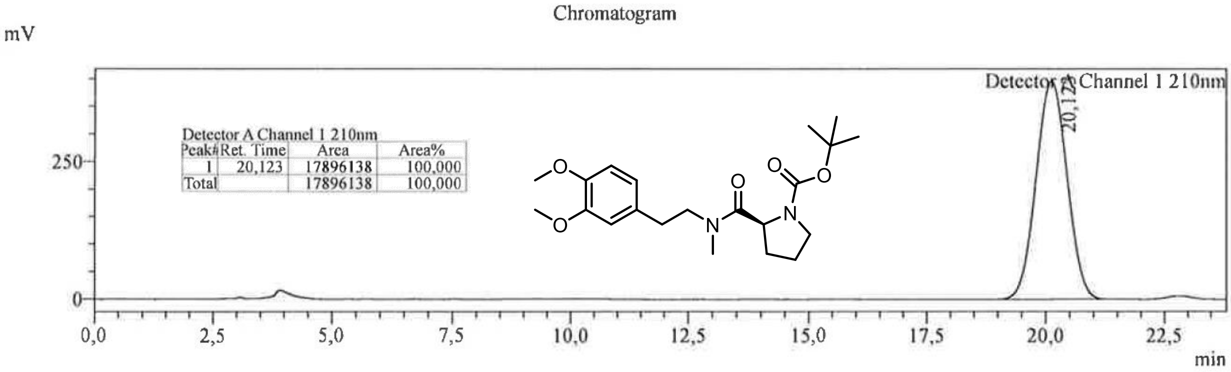

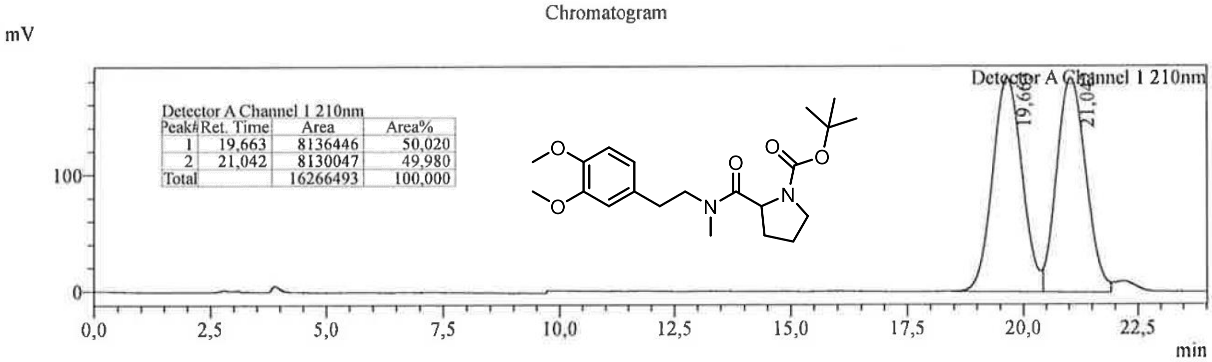

2-(3-Cyano-4-isobutoxyphenyl)-*N*-(3,4-dimethoxyphenethyl)-*N*,4-dimethylthiazole-5-carboxamide (9k)

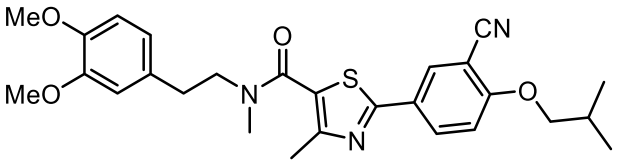

Following the GP1 (0.1 mmol scale), the titled compound was obtained as a colourless oil (37.5 mg, 76 µmol, 76%).

^{1}H NMR (400 MHz, CDCl$_3$): δ 8.08 (d, *J* = 2.2 Hz, 1H), 7.96 (d, *J* = 8.5 Hz, 1H), 6.99 (d, *J* = 8.9 Hz, 1H), 6.89 – 6.46 (m, 3H), 3.95 – 3.65 (m, 10H), 3.03 (br s, 3H), 2.87 (br s, 2H), 2.36 (s, 3H), 2.19 (hept, *J* = 6.6 Hz, 1H), 1.08 (d, *J* = 6.7 Hz, 6H) ppm.

^{13}C NMR (101 MHz, CDCl$_3$): δ 164.8, 163.7, 162.2, 152.7, 149.3, 148.1, 132.4, 131.8, 126.2, 125.0, 120.9, 115.6, 112.7, 112.0, 111.5, 103.0, 75.8, 56.1, 56.0, 53.2, 49.7, 33.6, 28.3, 19.2, 16.5 ppm. One carbon could not be clearly identified due to overlap with another signal.

IR (neat): ν_{max} 2959, 2874, 2228, 1625, 1513, 1262, 1156, 1026, 729 cm^{-1}.

HRMS (ESI$^+$): exact mass calculated for [M+Na]$^+$ ($C_{27}H_{31}N_3O_4SNa$)$^+$ requires *m/z* 516.1927, found *m/z* 516.1934.

***N*-(3,4-Dimethoxyphenethyl)-4-(*N*,*N*-dipropylsulfamoyl)-*N*-methylbenzamide (9l)**

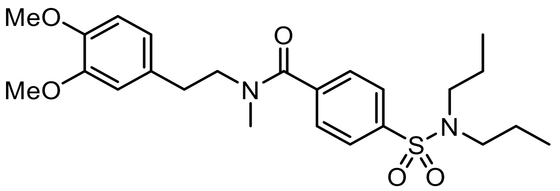

Following the GP1 (0.1 mmol scale), the titled compound was obtained as a colourless oil (33.6 mg, 73 µmol, 73%). The desired amide was obtained as a mixture of two rotamers in a ratio of around 1.3:1. NMR peaks corresponding exclusively to the minor rotamers are indicated with an asterisk.

^{1}H NMR (400 MHz, CDCl$_3$): δ 7.81 (d, *J* = 8.0 Hz, 0.9H*), 7.71 (d, *J* = 8.0 Hz, 1.1H), 7.40 (d, *J* = 8.0 Hz, 0.9H*), 7.08 (d, *J* = 8.0 Hz, 1.1H), 6.82 (app s, 1.2H), 6.74 (d, *J* = 8.1 Hz, 0.6H), 6.47 (d, *J* = 7.7 Hz, 0.6H), 6.34 (s, 0.6H), 3.90 – 3.84 (m, 4.4H), 3.80 – 3.73 (m, 2.5H), 3.41 (t, *J* = 6.6 Hz, 1.1H), 3.14 (s, 1.6H), 3.10 – 2.99 (m, 4H), 2.94 (t, *J* = 7.2 Hz, 0.9H*), 2.79 (s, 1.4H*), 2.71 (t, *J* = 6.6 Hz, 1.1H), 1.61 – 1.47 (m, 4H), 0.90 – 0.81 (m, 6H) ppm.

^{13}C NMR (101 MHz, CDCl$_3$): δ 170.7, 169.9, 149.2, 149.1, 148.1, 147.9, 141.2, 140.8, 140.5, 140.3, 131.2, 130.0, 127.5, 127.4, 127.3, 127.1, 121.0 (2C), 112.2, 111.9, 111.6, 111.4, 56.1 (4C), 53.1, 50.2 (4C), 49.3, 38.1, 34.1, 33.0 (2C), 22.2 (4C), 11.3 (4C) ppm.

IR (neat): ν_{max} 2965, 2876, 1630, 1515, 1336, 1262, 1155, 1027, 730 cm^{-1}.

HRMS (ESI$^+$): exact mass calculated for [M+Na]$^+$ ($C_{24}H_{34}N_2O_5SNa$)$^+$ requires *m/z* 485.2081, found *m/z* 485.2084.

***tert*-Butyl 4-(1-cyclopropyl-3-((3,4-dimethoxyphenethyl)(methyl)carbamoyl)-6-fluoro-4-oxo-1,4-dihydroquinolin-7-yl)piperazine-1-carboxylate (9m)**

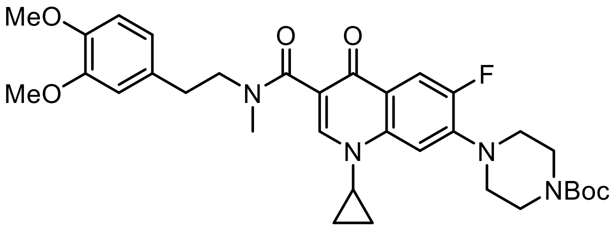

Following the GP1 (0.1 mmol scale), the titled compound was obtained as a pale-yellow oil (38.2 mg, 63 µmol, 63%). The desired amide was obtained as a mixture of two rotamers in a ratio of around 2.6:1. Both rotamers:

^{1}H NMR (400 MHz, CDCl$_3$): 8.03 – 7.95 (m, 1.4H), 7.21 (d, *J* = 7.1 Hz, 0.8H), 7.05 (s, 0.8H), 6.88 – 6.79 (m, 0.9H), 6.74 (d, *J* = 8.1 Hz, 0.7H), 6.56 (dd, *J* = 8.0, 1.6 Hz, 0.7H), 6.27 (d, *J* = 1.6 Hz, 0.7H), 3.90 (s, 0.7H), 3.87 – 3.83 (m, 3H), 3.74 – 3.59 (m, 6.2H), 3.39 – 3.26 (m, 3.3H), 3.23 – 3.13 (m, 6.1H), 2.98 – 2.89 (m, 1.4H), 2.72 (t, *J* = 5.9 Hz, 1.3H), 1.49 (s, 9H), 1.30 – 1.24 (m, 0.8H), 1.13 (d, *J* = 6.6 Hz, 1.9H), 0.91 (s, 1.3H).

^{13}C NMR (101 MHz, CDCl$_3$): δ 172.3, 171.9, 167.6, 167.3, 154.7 (2C), 153.3 (d, *J* = 248.5 Hz), 153.2 (d, *J* = 248.1 Hz), 149.0, 148.6, 147.6, 147.5, 144.6 (d, *J* = 10.9 Hz), 144.5 (d, *J* = 10.7 Hz), 144.1, 143.7, 138.5, 138.1, 132.1, 131.9, 122.1 (d, *J* = 6.8 Hz), 121.9 (d, *J* = 6.9 Hz), 121.6, 120.9, 118.8, 118.7, 113.0 (d, *J* = 23.5 Hz), 112.9 (d, *J* = 22.8 Hz), 112.4, 111.7, 111.4, 110.8, 104.8 (2C), 80.3 (2C), 56.0 (2C), 55.9, 55.3, 53.0, 50.3, 50.3, 50.2, 43.6 (br s, 2C), 37.3, 34.3, 34.0 (2C), 33.6, 33.4, 28.6 (2C), 8.2, 7.9 ppm.

^{19}F NMR (376 MHz, CDCl$_3$): δ -124.61 (dd, *J* = 13.0, 7.0 Hz, 0.4F), -124.80 (dd, *J* = 13.1, 7.1 Hz, 1F) ppm.

IR (neat): ν_{max} 2931, 2860, 1623, 1478, 1243, 1158, 1024, 723 cm^{-1}.

HRMS (ESI$^+$): exact mass calculated for $[M+Na]^+$ $(C_{33}H_{41}FN_4O_6Na)^+$ requires *m/z* 631.2902, found *m/z* 631.2914.

(*Z*)-*N*-(3,4-dimethoxyphenethyl)-2-(5-fluoro-2-methyl-1-(4-(methylsulfinyl)benzylidene)-1*H*-inden-3-yl)-*N*-methylacetamide (9n)

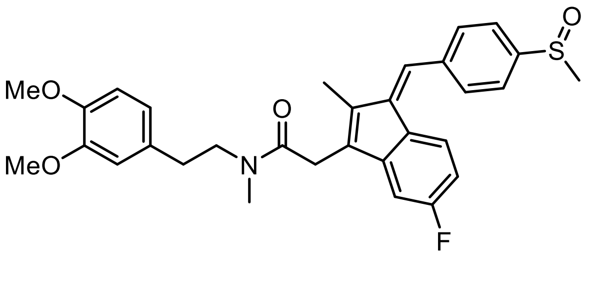

Following the GP1 (0.1 mmol scale), the titled compound was obtained as a yellow solid (37.4 mg, 70 µmol, 70%). The desired amide was obtained as a mixture of two rotamers in a ratio of around 1.1:1. NMR peaks corresponding exclusively to the minor rotamers are indicated with an asterisk.

^{1}H NMR (700 MHz, CDCl$_3$): δ 7.71 – 7.68 (m, 2H), 7.65 (d, *J* = 8.0 Hz, 1H), 7.63 (d, *J* = 8.0 Hz, 1H*), 7.14 – 7.08 (m, 1.5H), 7.07 (s, 0.5H*), 6.89 (dd, *J* = 9.0, 2.4 Hz, 0.5H), 6.83 (d, *J* = 8.1 Hz, 0.5H*), 6.76 (d, *J* = 7.9 Hz, 0.5H), 6.72 – 6.67 (m, 2H), 6.65 (d, *J* = 1.9 Hz, 0.5H*), 6.56 – 6.49 (m, 1H), 3.87 – 3.80 (m,

6H), 3.62 – 3.55 (m, 3H), 3.25 (s, 1H), 3.01 (s, 1.5H*), 2.98 (s, 1.5H), 2.81 – 2.77 (m, 5H), 2.15 (s, 1.5H), 2.03 (s, 1.5H*) ppm.

^{13}C NMR (176 MHz, CDCl$_3$): δ 169.7, 169.3, 163.5 (d, *J* = 246.0 Hz), 163.4 (d, *J* = 246.4 Hz), 149.3, 149.0, 148.2, 147.7, 147.2, 147.2, 145.5, 145.4, 141.9, 139.9, 137.4, 137.2, 133.3, 133.1 (d, *J* = 2.0 Hz), 131.6, 130.7, 130.4, 129.7 (d, *J* = 2.2 Hz), 129.6 (d, *J* = 2.2 Hz), 123.9, 123.9, 123.6 (d, *J* = 8.2 Hz), 123.6 (d, *J* = 7.7 Hz), 121.1, 120.8, 112.1, 112.1, 111.7, 111.4, 110.8 (d, *J* = 22.4 Hz), 110.7 (d, *J* = 22.6 Hz), 106.5 (d, *J* = 23.9 Hz), 106.4 (d, *J* = 24.1 Hz), 56.1, 56.0, 56.0, 56.0, 51.9, 50.6, 44.0, 36.5, 34.3, 33.7, 33.4, 32.1, 31.5, 10.7, 10.6 ppm.

^{19}F NMR (659 MHz, CDCl$_3$): δ -112.79, -112.84 ppm.

IR (neat): ν_{max} 1709, 1638, 1514, 1465, 1263, 1236, 1028, 812 cm^{-1}.

HRMS (ESI$^+$): exact mass calculated for [M+Na]$^+$ ($C_{31}H_{32}FNO_4SNa$)$^+$ requires *m/z* 556.1928, found *m/z* 556.1923.

***tert*-Butyl (*S*)-(2-((1-((3,4-dimethoxyphenethyl)(methyl)amino)-4-methyl-1-oxopentan-2-yl)amino)-2-oxoethyl)carbamate (9o)**

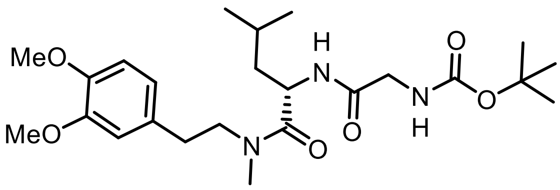

Following the GP1 (0.1 mmol scale), the titled compound was obtained as a colourless oil (20.6 mg, 44 µmol, 44%). The desired amide was obtained as a mixture of two rotamers in a ratio of around 1.3:1. NMR peaks in ^{1}H NMR corresponding exclusively to the minor rotamer are indicated with an asterisk.

^{1}H NMR (400 MHz, CDCl$_3$): δ 6.83 – 6.61 (m, 4H), 5.14 (s, 1H), 4.98 – 4.81 (m, 1H), 3.89 – 3.84 (m, 6H), 3.78 – 3.46 (m, 3H), 2.97 (s, 1.7H), 2.96 (s, 1.3H), 2.88 – 2.75 (m, 2H), 1.79 – 1.52 (m, 2H), 1.47 – 1.43 (m, 9H), 1.33 – 1.18 (m, 1H), 0.99 – 0.91 (m, 4H), 0.87 (t, *J* = 6.5 Hz, 3H) ppm.

^{13}C NMR (101 MHz, CDCl$_3$): 172.6, 172.2, 169.0, 168.9, 155.9, 149.2, 149.0, 148.0, 147.8, 131.3, 130.4, 120.9, 120.9, 112.2, 112.0, 111.7, 111.4, 80.3, 56.0, 56.0, 52.0, 50.1, 47.5, 47.2, 45.4, 44.3, 42.9, 42.5, 35.8, 34.8, 34.2, 33.2, 28.4, 24.7, 24.6, 23.5, 22.6, 21.9, 21.8 ppm.

IR (neat): ν_{max} 1712, 1669, 1630, 1514, 1464, 1453, 1260, 1235, 1156, 1027, 914, 728 cm^{-1}.

HRMS (ESI$^+$): exact mass calculated for [M+Na]$^+$ ($C_{24}H_{39}N_3O_6Na$)$^+$ requires *m/z* 488.2731, found *m/z* 488.2724.

tert-Butyl ((_S_)-1-((_S_)-2-((3,4-dimethoxyphenethyl)(methyl)carbamoyl)pyrrolidin-1-yl)-3-methyl-1-oxobutan-2-yl)carbamate (9p)

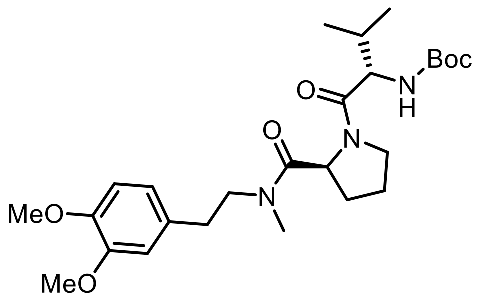

Following the GP1 (0.1 mmol scale), the titled compound was obtained as a colorless oil (33.6 mg, 68 µmol, 68%). The desired amide was obtained as a mixture of two rotamers in a ratio of around 1.5:1. NMR peaks in ^{1}H NMR corresponding exclusively to the minor rotamer are indicated with an asterisk.

^{1}H NMR (400 MHz, CDCl$_3$): δ 6.82 – 6.70 (m, 3H), 5.21 (d, *J* = 9.3 Hz, 1H), 4.84 – 4.73 (m, 1H), 4.34 – 4.25 (m, 1H), 3.90 – 3.84 (m, 6H), 3.78 – 3.37 (m, 4H), 2.98 (s, 1.8H), 2.97 – 2.89 (m, 1H), 2.85 (s, 1.2H), 2.76 (t, 1H), 2.19 – 1.72 (m, 6H), 1.41 (s, 9H), 1.06 – 1.00 (m, 3H), 0.94 – 0.88 (m, 3H) ppm.
^{13}C NMR (101 MHz, CDCl$_3$): δ 171.9, 171.5, 171.0, 170.9, 156.0, 149.2, 149.0, 147.9, 147.7, 131.9, 131.1, 120.9, 120.8, 112.4, 112.3, 111.6, 111.4, 79.5, 57.0, 56.9, 56.7, 56.5, 56.1, 56.0, 51.9, 50.6, 47.6, 47.5, 36.1, 34.8, 34.7, 33.3, 31.5, 31.4, 29.3, 28.7, 28.5, 25.2, 25.1, 19.6, 19.5, 17.6, 17.5.
IR (neat): ν_{max} 1706, 1634, 1514, 1439, 1366, 1261, 1235, 1157, 1027, 911, 726 cm^{-1}.
HRMS (ESI$^+$): exact mass calculated for $[M+Na]^+$ $(C_{26}H_{41}N_3O_6Na)^+$ requires *m/z* 514.2888, found *m/z* 514.2881.

(4*R*)-*N*-(3,4-Dimethoxyphenethyl)-4-((8*R*,9*S*,10*S*,13*R*,14*S*,17*R*)-10,13-dimethyl-3,7,12-trioxohexadecahydro-1*H*-cyclopenta[*a*]phenanthren-17-yl)-*N*-methylpentanamide (9q)

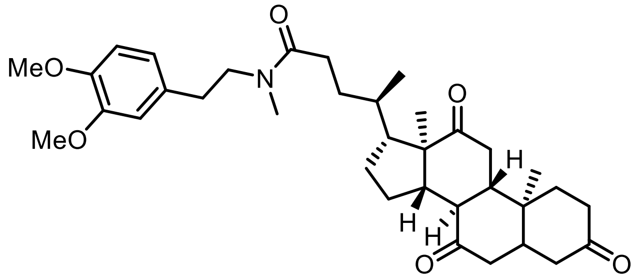

Following the GP1 (0.1 mmol scale), the titled compound was obtained as colourless solid (32.0 mg, 55 µmol, 55%). The desired amide was obtained as a mixture of two rotamers in a ratio of around 1.1:1. NMR peaks corresponding exclusively to the minor rotamers are indicated with an asterisk.

^{1}H NMR (600 MHz, CDCl$_3$): δ 6.79 (d, *J* = 8.1 Hz, 0.5H*), 6.77 (d, *J* = 8.0 Hz, 0.5H), 6.74 – 6.72 (m, 1H), 6.67 (dd, *J* = 8.1, 1.9 Hz, 0.5H*), 6.63 (d, *J* = 1.9 Hz, 0.5H*), 3.86 – 3.83 (m, 6H), 3.59 – 3.44 (m, 2H), 2.94 – 2.74 (m, 8H), 2.36 – 1.65 (m, 16H), 1.63 – 1.56 (m, 1H), 1.40 – 1.37 (m, 3H), 1.36 – 1.19 (m, 4H), 1.06 (s, 1.6H), 1.03 (s, 1.4H*), 0.84 (d, *J* = 6.4 Hz, 1.6H), 0.74 (d, *J* = 6.4 Hz, 1.4H*) ppm.

^{13}C NMR (151 MHz, CDCl$_3$): δ 212.2 (2C), 209.2, 208.8 (2C), 173.5, 173.2, 149.2, 149.0, 148.0, 147.6, 131.9, 130.9, 120.9, 120.8, 112.1, 112.0, 111.6, 111.3, 57.0, 57.0, 56.0, 55.9, 51.9 (2C), 51.8.50.1, 49.1, 46.9, 45.8, 45.7, 45.7, 45.6, 45.1, 42.9, 38.8, 38.7, 36.6, 36.2, 36.1, 35.6, 35.6, 35.4, 34.5, 33.6, 33.4, 30.8, 30.6 (2C), 29.8, 27.7, 27.5, 25.3, 25.2, 22.0, 18.9 (2C), 12.0, 11.9 ppm.

IR (neat): ν_{max} 1707, 1629, 1515, 1464, 1264, 1236, 1028 cm^{-1}.

HRMS (ESI⁺): exact mass calculated for $[M+Na]^+$ $(C_{35}H_{49}NO_6Na)^+$ requires *m/z* 602.3542, found *m/z* 602.3542.

2-(1-(4-Chlorobenzoyl)-5-methoxy-2-methyl-1*H*-indol-3-yl)-*N*-(3,4-dimethoxyphenethyl)-*N*-methylacetamide (9r)

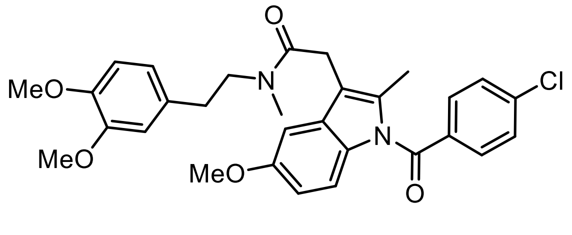

Following the GP1 (0.1 mmol scale), the titled compound was obtained as yellow solid (51.0 mg, 95 µmol, 95%). The desired amide was obtained as a mixture of two rotamers in a ratio of around 1.2:1. NMR peaks corresponding exclusively to the minor rotamers are indicated with an asterisk.

^{1}H NMR (400 MHz, CDCl$_3$): δ 7.64 (dd, *J* = 13.2, 8.5 Hz, 2H), 7.45 (dd, *J* = 9.9, 8.6 Hz, 2H), 7.03 (d, *J* = 2.4 Hz, 0.5H), 6.92 (d, *J* = 2.4 Hz, 0.5H), 6.86 – 6.78 (m, 1.5H), 6.76 – 6.59 (m, 3.5H), 3.89 – 3.76 (m, 9H), 3.68 (s, 1.1H), 3.63 – 3.53 (m, 2H), 3.41 (s, 0.9H*), 3.00 (s, 1.4H*), 2.97 (s, 1.6H), 2.83 – 2.71 (m, 2H), 2.35 (s, 1.6H), 2.25 (s, 1.4H*) ppm.

^{13}C NMR (101 MHz, CDCl$_3$): δ 170.3, 169.9, 168.4 (2C), 156.1 (2C), 149.3, 149.0, 148.1, 147.7, 139.3 (2C), 135.4, 135.2, 134.1 (2C), 131.7, 131.3 (2C), 131.0 (3C), 130.9, 130.7, 129.2 (2C), 120.9, 120.8, 115.0, 114.9, 113.6, 113.5, 112.1 (2C), 111.6 (2C), 111.5, 111.4, 101.9, 101.8, 56.1, 56.0 (3C) , 55.8 (2C), 52.0, 50.7, 36.6, 34.3, 33.9, 33.5, 30.8, 30.2, 13.6, 13.5 ppm.

IR (neat): ν_{max} 2932, 1639, 1514, 1453, 1315, 1233, 1143, 1028, 726 cm^{-1}.

HRMS (ESI⁺): exact mass calculated for $[M+Na]^+$ $(C_{30}H_{31}ClN_2O_5Na)^+$ requires *m/z* 557.1814, found *m/z* 557.1817.

4-Chloro-*N*-phenethylbenzamide (12a)

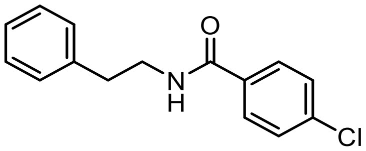

Following the GP2 (0.1 mmol scale), the titled compound was obtained as a colourless solid (24.2 mg, 93 µmol, 93%).

^{1}H NMR (400 MHz, CDCl$_3$): δ 7.62 (d, *J* = 8.6 Hz, 2H), 7.41 – 7.30 (m, 4H), 7.30 – 7.19 (m, 3H), 6.11 (br s, 1H), 3.71 (q, *J* = 6.8 Hz, 2H), 2.93 (t, *J* = 6.9 Hz, 2H) ppm.

^{13}C NMR (101 MHz, CDCl$_3$): δ 166.5, 138.9, 137.8, 133.1, 129.0, 128.9 (2C), 128.4, 126.8, 41.3, 35.8 ppm.

IR (neat): ν_{max} 3344, 2924, 1637, 1539, 1478, 1091, 1012, 843 cm^{-1}.

HRMS (ESI$^+$): exact mass calculated for [M+Na]$^+$ ($C_{15}H_{14}ClNONa$)$^+$ requires *m/z* 282.0656, found *m/z* 282.0656.

***N*-(2-(1*H*-Indol-3-yl)ethyl)-4-chlorobenzamide (12b)**

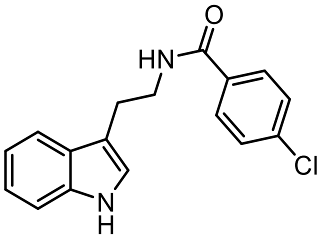

Following the GP2 (0.1 mmol scale), the titled compound was obtained as a beige solid (22.3 mg, 75 µmol, 75%).

^{1}H NMR (400 MHz, CDCl$_3$): δ 8.18 (br s, 1H), 7.68 – 7.56 (m, 3H), 7.44 – 7.31 (m, 3H), 7.23 (t, *J* = 7.6 Hz, 1H), 7.13 (t, *J* = 7.5 Hz, 1H), 7.06 (d, *J* = 2.3 Hz, 1H), 6.21 (br s, 1H), 3.79 (q, *J* = 6.6 Hz, 2H), 3.10 (t, *J* = 6.6 Hz, 2H) ppm.

^{13}C NMR (101 MHz, CDCl$_3$): δ 166.5, 137.7, 136.6, 133.2, 128.9, 128.4, 127.4, 122.5, 122.2, 119.8, 118.8, 113.1, 111.5, 40.5, 25.4 ppm.

IR (neat): ν_{max} 3329, 3254, 2938, 1610, 1561, 1320, 1091, 850 cm^{-1}.

HRMS (ESI$^+$): exact mass calculated for [M+Na]$^+$ ($C_{17}H_{15}ClN_2ONa$)$^+$ requires *m/z* 321.0765, found *m/z* 321.0761.

N-(tert-Butyl)-4-chlorobenzamide (12c)

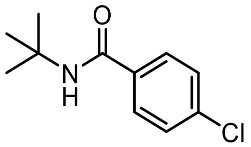

Following the GP2 (0.1 mmol scale), the titled compound was obtained as a colourless solid (19.5 mg, 92 µmol, 92%).

^{1}H NMR (400 MHz, CDCl$_3$): δ 7.66 (d, *J* = 8.5 Hz, 2H), 7.38 (d, *J* = 8.5 Hz, 2H), 5.88 (s, 1H), 1.47 (s, 9H) ppm.

^{13}C NMR (101 MHz, CDCl$_3$): δ 166.0, 137.4, 134.4, 128.8, 128.3, 51.9, 29.0 ppm.

IR (neat): ν_{max} 3319, 2975, 1634, 1537, 1486, 1317, 1218, 1090, 761 cm^{-1}.

HRMS (ESI$^+$): exact mass calculated for $[M+H]^+$ $(C_{11}H_{15}ClNO)^+$ requires *m/z* 212.0837, found *m/z* 212.0833.

4-Chloro-N-(4-methoxybenzyl)benzamide (12d)

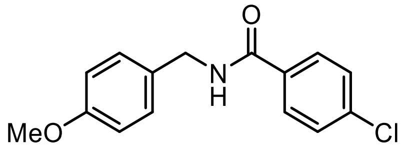

Following the GP2 (0.1 mmol scale), the titled compound was obtained as a colourless solid (19.5 mg, 71 µmol, 71%).

^{1}H NMR (400 MHz, CDCl$_3$): δ 7.71 (d, *J* = 8.6 Hz, 2H), 7.38 (d, *J* = 8.5 Hz, 2H), 7.26 (d, *J* = 8.6 Hz, 2H), 6.88 (d, *J* = 8.7 Hz, 2H), 6.39 (s, 1H), 4.55 (d, *J* = 5.5 Hz, 2H), 3.80 (s, 3H) ppm.

^{13}C NMR (101 MHz, CDCl$_3$): δ 166.3, 159.3, 137.9, 132.9, 130.1, 129.5, 129.0, 128.5, 114.3, 55.5, 43.9 ppm.

IR (neat): ν_{max} 3329, 2930, 1637, 1542, 1510, 1245, 1171, 1034, 848 cm^{-1}.

HRMS (ESI$^+$): exact mass calculated for $[M+Na]^+$ $(C_{15}H_{14}ClNO_2Na)^+$ requires *m/z* 298.0605, found *m/z* 298.0599.

Methyl (4-chlorobenzoyl)-L-isoleucinate (12e)

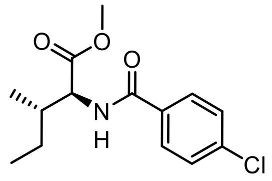

Following the GP2 (0.1 mmol scale), the titled compound was obtained as a colourless solid (12.0 mg, 42 µmol, 42%). In addition, unreacted starting material was recovered (7.5 mg, 15 µmol, 15%).

^{1}H NMR (400 MHz, CDCl$_3$): δ 7.78 – 7.67 (m, 2H), 7.46 – 7.34 (m, 2H), 6.61 (d, *J* = 8.1 Hz, 1H), 4.80 (dd, *J* = 8.4, 4.9 Hz, 1H), 3.78 (s, 3H), 2.06 – 1.96 (m, 1H), 1.57 – 1.46 (m, 1H), 1.32 – 1.20 (m, 1H), 0.99 – 0.94 (m, 6H) ppm.

^{13}C NMR (101 MHz, CDCl$_3$): δ 172.7, 166.2, 138.2, 132.7, 129.0, 128.6, 57.0, 52.4, 38.4, 25.5, 15.6, 11.8 ppm.

IR (neat) ν_{max}: 3318, 2964, 1741, 1639, 1596, 1528, 1484, 1273, 1200, 1179, 1152, 1091, 1014, 845, 758.

HRMS (ESI$^+$): exact mass calculated for [M+Na]$^+$ ($C_{14}H_{18}ClNO_3Na^+$) requires *m/z* 306.0867, found *m/z* 306.0865.

(4-Chlorophenyl)(4-(4,4,5,5-tetramethyl-1,3,2-dioxaborolan-2-yl)piperidin-1-yl)methanone (12f)

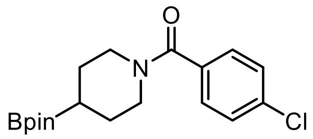

Following the GP1 (0.081 mmol scale), the titled compound was obtained as a colourless solid (19.9 mg, 57 µmol, 71%).

^{1}H NMR (400 MHz, CDCl$_3$): δ 7.38 – 7.31 (m, 4H), 4.26 (br s, 1H), 3.54 (br s, 1H), 3.15 – 3.02 (m, 2H), 1.89 – 1.43 (m, 5H), 1.24 (s, 12H) ppm.

^{13}C NMR (101 MHz, CDCl$_3$): δ 169.4, 135.5, 135.0, 128.8, 128.6, 83.5, 49.2, 43.6, 27.9, 27.0, 24.9, 20.2 ppm.

IR (neat): ν_{max} 2980, 2970, 1633, 1386, 1332, 1276, 1135, 997, 848 cm^{-1}.

HRMS (ESI$^+$): exact mass calculated for [M+H]$^+$ ($C_{18}H_{26}BClNO_3$)$^+$ requires *m/z* 350.1689, found *m/z* 350.1694.

8-(4-Chlorobenzoyl)-8-azabicyclo[3.2.1]octan-3-one (12g)

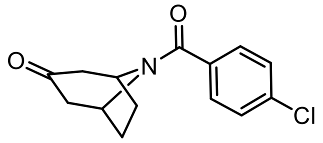

Following the GP1 (0.1 mmol scale), the titled compound was obtained as a colourless solid (22.0 mg, 85 µmol, 85%).

^{1}H NMR (400 MHz, CDCl$_3$): δ 7.51 – 7.45 (m, 2H), 7.44 – 7.40 (m, 2H), 5.03 (br s, 1H), 4.38 (br s, 1H), 2.92 (br s, 1H), 2.47 (br s, 3H), 2.23 – 2.09 (m, 2H), 1.77 (app d, *J* = 8.3 Hz, 2H) ppm.

^{13}C NMR (101 MHz, CDCl$_3$): δ 207.2, 168.0, 136.9, 133.9, 129.1, 128.8, 56.3, 51.9, 49.7, 48.8, 29.8, 28.2 ppm.

IR (neat) ν_{max}: 1710, 1633, 1412, 1359, 1220, 1089, 732. 1092

HRMS (ESI⁺): exact mass calculated for $[M+Na]^+$ ($C_{14}H_{14}ClNO_2Na^+$) requires *m/z* 286.0605, found *m/z* 286.0603.

((3*S*,4*R*)-3-((Benzo[*d*][1,3]dioxol-5-yloxy)methyl)-4-(4-fluorophenyl)piperidin-1-yl)(4-chlorophenyl)methanone (12h)

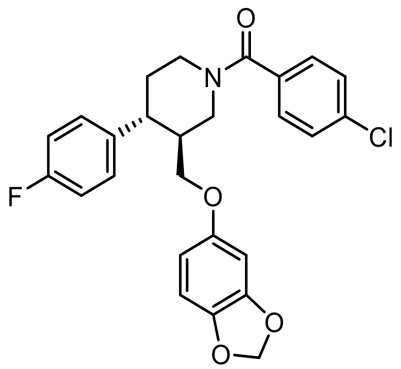

Following the GP1 (0.1mmol scale), the titled compound was obtained as a colourless oil (34.5 mg, 74 µmol, 74%).

^{1}H NMR (400 MHz, CDCl$_3$): δ 7.40 (s, 4H), 7.16 (dd, *J* = 8.6, 5.4 Hz, 2H), 7.00 (t, *J* = 8.6 Hz, 2H), 6.62 (d, *J* = 7.8 Hz, 1H), 6.43 – 5.96 (m, 2H), 5.89 (s, 2H), 5.10 – 4.77 (m, 1H), 4.22 – 3.82 (m, 1H), 3.74 – 3.37 (m, 2H), 3.22 – 2.75 (m, 3H), 2.19 – 1.68 (m, 3H) ppm.

^{13}C NMR (101 MHz, CDCl$_3$): δ 169.6, 161.9 (d, *J* = 245.2 Hz), 148.4, 142.0, 138.5 (d, *J* = 3.2 Hz), 136.0, 134.4, 128.9 (2C), 128.8, 115.8 (d, *J* = 21.2 Hz), 108.0, 105.7, 101.3, 98.1, 68.5, 51.3, 48.4, 44.2, 43.0, 34.8, 33.8 ppm.

^{19}F NMR (376 MHz, CDCl$_3$): δ -115.66 (d, *J* = 40.6 Hz, 1F) ppm.

IR (neat): ν_{max} 2919, 1627, 1486, 1181, 1089, 1015, 832, 729 cm^{-1}.

HRMS (ESI⁺): exact mass calculated for $[M+Na]^+$ $(C_{26}H_{23}ClFNO_4Na)^+$ requires *m/z* 490.1192, found *m/z* 490.1193.

(4-(8-Chloro-5,6-dihydro-11*H*-benzo[5,6]cyclohepta[1,2-*b*]pyridin-11-ylidene)piperidin-1-yl)(4-chlorophenyl)methanone (12i)

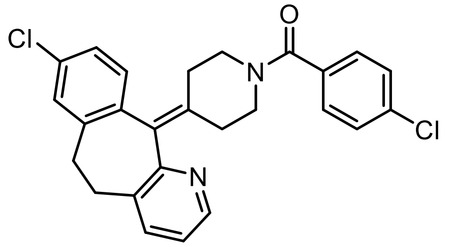

Following the GP1 (0.1 mmol scale), the titled compound was obtained as a colourless oil (36.5 mg, 81 µmol, 81%).

^{1}H NMR (400 MHz, CDCl$_3$): δ 8.38 (br s, 1H), 7.44 (d, *J* = 7.2 Hz, 1H), 7.35 (app s, 4H), 7.22 – 7.04 (m, 4H), 4.15 (br s, 1H), 3.59 (br s, 1H), 3.46 – 3.16 (m, 4H), 2.93 – 2.74 (m, 2H), 2.66 – 2.24 (m, 4H) ppm.

^{13}C NMR (101 MHz, CDCl$_3$): δ 169.5, 156.8, 146.8, 139.7, 137.8, 137.6, 136.7, 135.8, 135.1, 134.5, 133.5, 133.2, 130.5, 129.2, 128.9, 128.6, 126.4, 122.5, 48.7, 43.5, 31.8, 31.7, 31.4, 30.5 ppm.

IR (neat): ν_{max} 2907, 1625, 1434, 1279, 1088, 991, 829, 726 cm^{-1}.

HRMS (ESI$^+$): exact mass calculated for $[M+H]^+$ $(C_{26}H_{23}Cl_2N_2O)^+$ requires *m/z* 449.1182, found *m/z* 449.1178.

(*S*)-4-Chloro-*N*-methyl-*N*-(3-(naphthalen-1-yloxy)-3-(thiophen-2-yl)propyl)benzamide (12j)

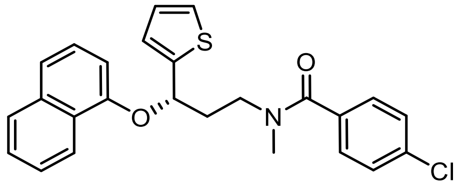

Following the GP1 (0.1 mmol scale), the titled compound was obtained as a colourless solid (35.0 mg, 80 µmol, 80%). The desired amide was obtained as a mixture of two rotamers in a ratio of around 1.3:1. NMR peaks corresponding exclusively to the minor rotamer are indicated with an asterisk.

^{1}H NMR (600 MHz, CDCl$_3$): δ 8.37 (s, 0.4H*), 7.92 – 7.87 (m, 0.6H), 7.81 – 7.73 (m, 1H), 7.51 – 7.38 (m, 3H), 7.29 – 6.66 (m, 9H), 5.81 (s, 0.4H*), 5.51 (s, 0.6H*), 3.92 (s, 0.4*H), 3.72 – 3.51 (m, 1.6H), 3.13 – 2.90 (m, 3H), 2.66 – 2.29 (m, 2H) ppm.

^{13}C NMR (151 MHz, CDCl$_3$): δ 171.2, 170.6, 153.2, 152.8, 144.8, 144.0, 135.7, 135.5, 134.7 (2C), 134.4, 128.7, 128.1, 127.7, 126.9, 126.7, 126.5, 125.9, 125.6, 125.5, 125.1, 124.7, 122.1, 121.6, 121.0, 107.2, 106.5, 74.8, 73.1, 48.0, 45.3, 38.2, 37.7, 36.3, 33.2 ppm.

IR (neat) ν_{max}: 1709, 1628, 1396, 1264, 1235, 1220, 1091, 1065, 771.

HRMS (ESI$^+$): exact mass calculated for $[M+Na]^+$ $(C_{25}H_{22}ClNO_2SNa^+)$ requires *m/z* 458.0952, found *m/z* 458.0952.

4.2.3 Scale-up of 9r

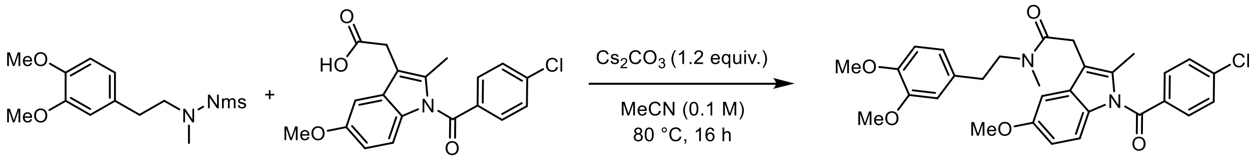

1c

9p, 89%

A 100 mL round-bottom flask was charged with sulfonamide **1c** (1.35 g, 2.5 mmol, 1.0 equiv.), Indomethacin (1.07 g, 3 mmol, 1.2 equiv.) and cesium carbonate (0.98 g, 3 mmol, 1.2 equiv.), before MeCN (25 mL) was added as solvent. The flask was closed with a plastic stopper and placed in an oil bath, and the mixture was heated for 16 h at 80 °C. After cooling to room temperature, the crude material was passed through a pad of Celite using EtOAc as eluent. After evaporation of the solvent, the crude material was subjected to column chromatography (silica gel, heptane/EtOAc) to afford the amide **9r** as a pale-yellow solid (1.193 g, 2.23 mmol, 89%)

Note: The reaction was performed under an atmosphere of air.

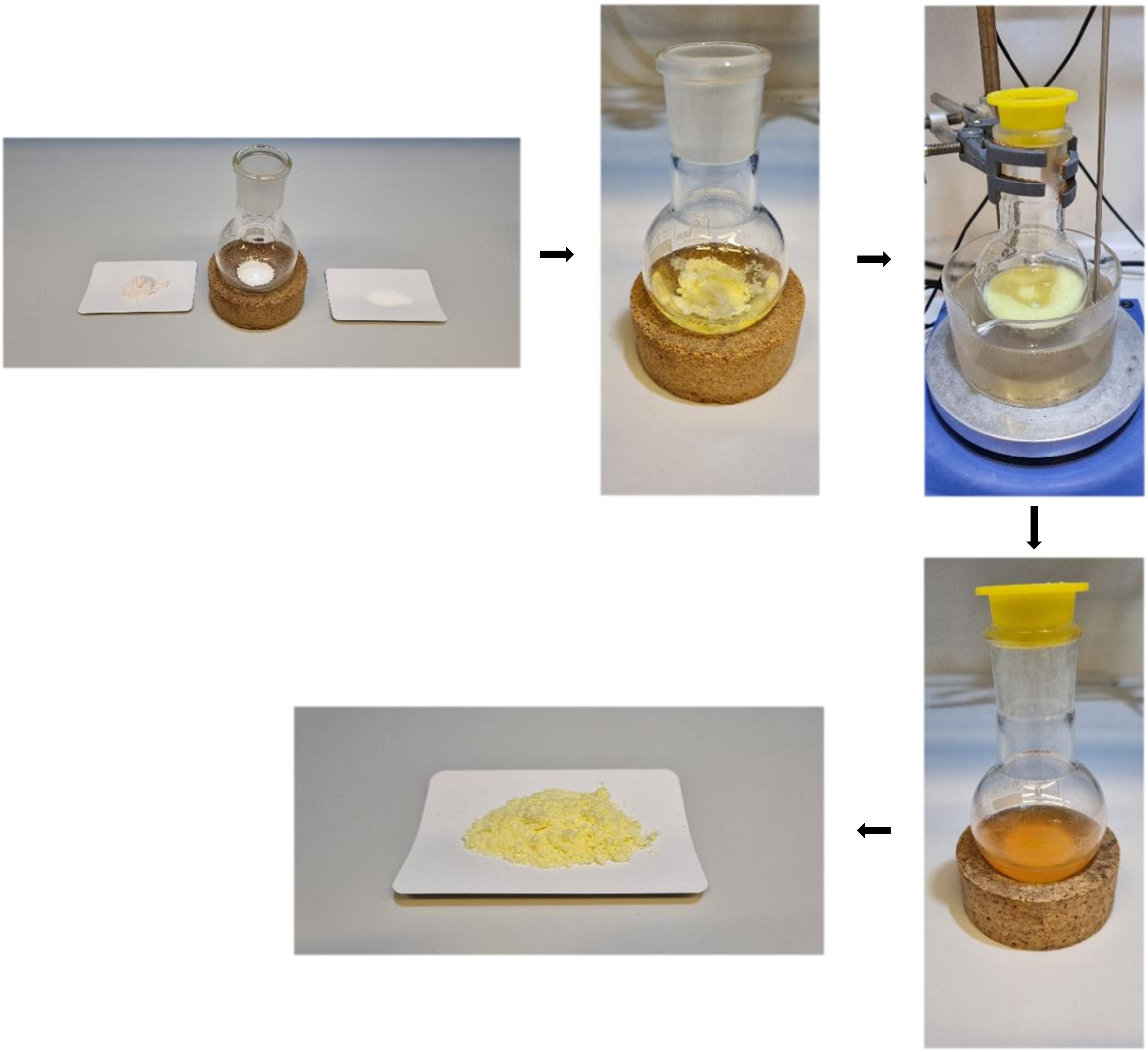

4.2.4 Failed examples

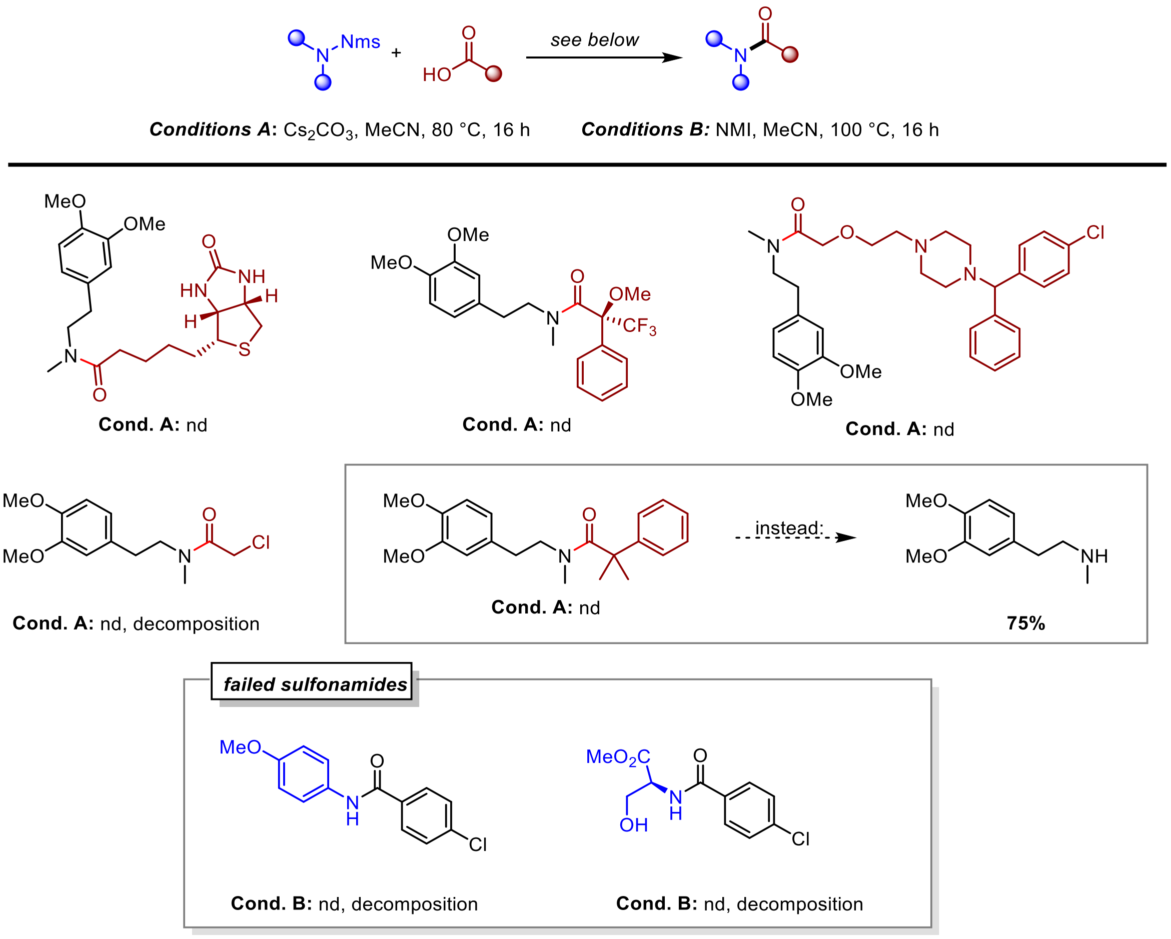

4.2.5 Evidence for proposed deprotective amidation mechanism

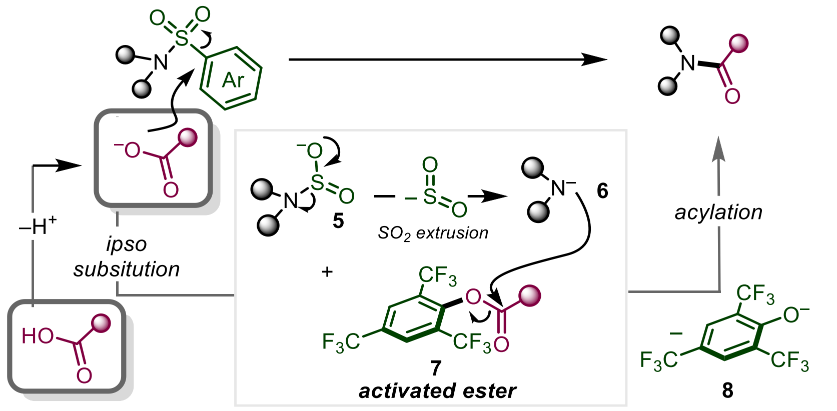

When examining the acid scope of this reaction, we only observed traces of the desired amide for ferrocene monocarboxylic acid. Instead, the major product was the ester **S8**. This result clearly shows that the activated ester is formed in the course of deprotection. The observation of **S8**, rather than the desired amide product, likely results from inefficient reaction of the amine with the sterically encumbered environment of the carbonyl of **S8** (see failed examples for related cases where free amine was isolated instead of amide).

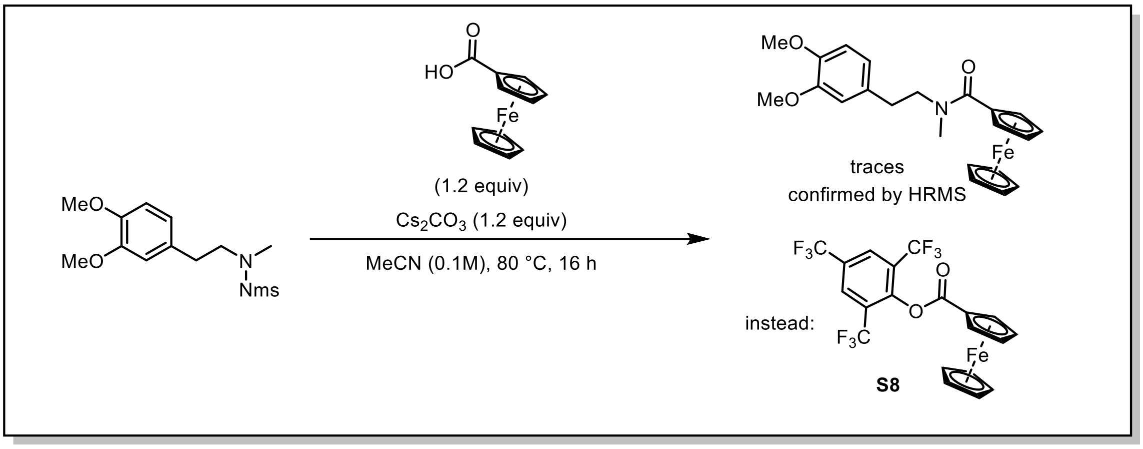

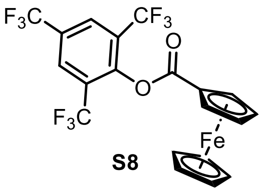

Following the GP1 (0.1 mmol scale), **S8** was obtained as an orange solid (37.9 mg, 74 µmol, 74%).

^{1}H NMR (400 MHz, CDCl$_3$): δ 8.17 (s, 2H), 4.97 – 4.91 (m, 2H), 4.59 – 4.55 (m, 2H), 4.37 (s, 5H).

^{13}C NMR (100 MHz, CDCl$_3$): δ 169.4, 150.2, 129.3 (q, *J* = 34.8 Hz), 128.5, 127.5 (q, *J* = 33.0 Hz), 122.6 (q, *J* = 272.9 Hz), 121.9 (q, *J* = 274.1 Hz), 72.4, 71.1, 70.2, 68.8 ppm.

^{19}F NMR (376 MHz, CDCl$_3$): δ -61.41 (6F), -62.77 (3F) ppm.

HRMS (ESI$^+$): exact mass calculated for $[M+Na]^+$ ($C_{20}H_{11}F_9O_2Fe^+$) requires *m/z* 509.9959, found *m/z* 509.9953.

HRMS detection for the formation of the desired ferrocene amide in trace amounts:

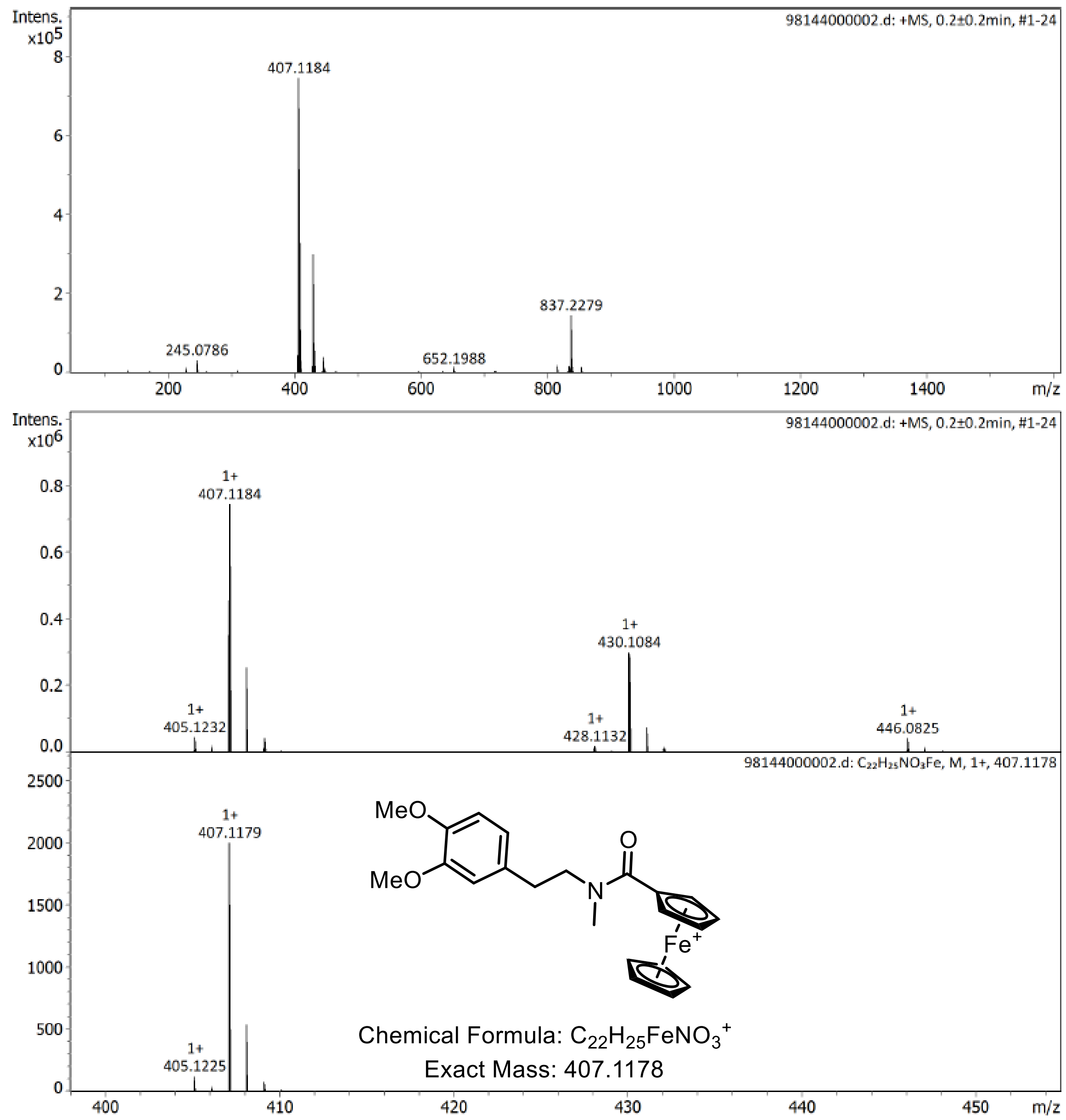

8 was also consistently observed as a by-product by crude NMR analysis. However, it could not be readily isolated cleanly by column chromatography (tailing on column chromatography) without impurities. The spectra shown below are ^{1}H and ^{19}F only (clean ^{13}C could not be obtained). When attempting to remove all solvent under high vacuum, the material also began to decompose. Its presence was additionally detected by HRMS analysis (see below).

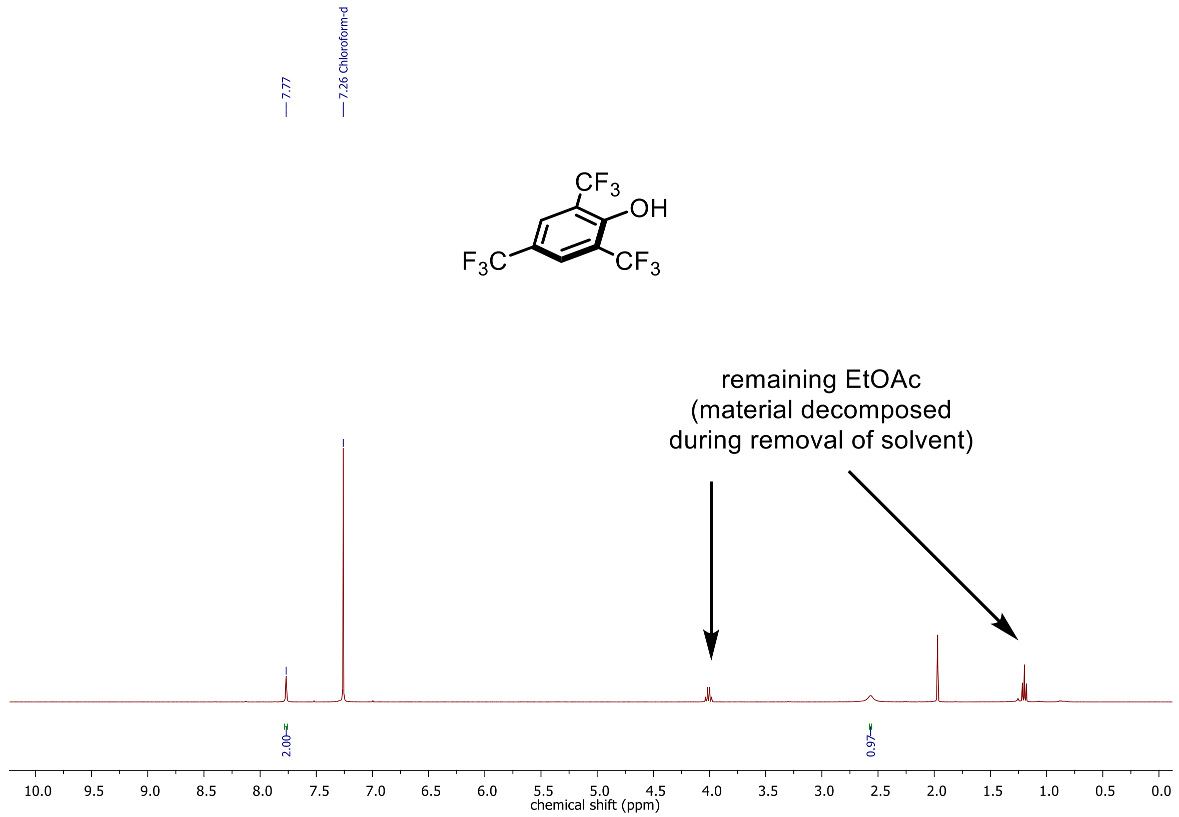
7.77
7.26 Chloroform-d
CF3
OH
F3C
CF3
remaining EtOAc
(material decomposed
during removal of solvent)
2.00
0.97
10.0 9.5 9.0 8.5 8.0 7.5 7.0 6.5 6.0 5.5 5.0 4.5 4.0 3.5 3.0 2.5 2.0 1.5 1.0 0.5 0.0
chemical shift (ppm)

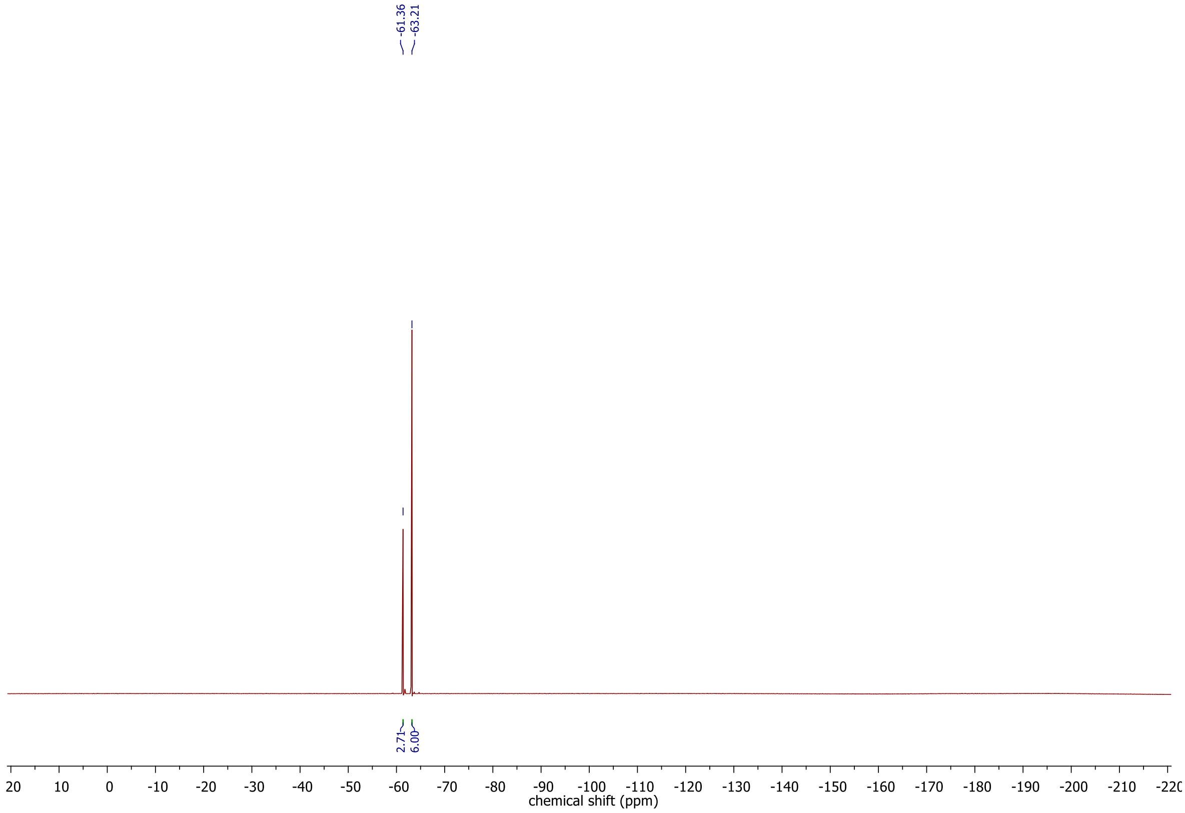
-61.36
-63.21
2.71
6.00
20 10 0 -10 -20 -30 -40 -50 -60 -70 -80 -90 -100 -110 -120 -130 -140 -150 -160 -170 -180 -190 -200 -210 -220
chemical shift (ppm)

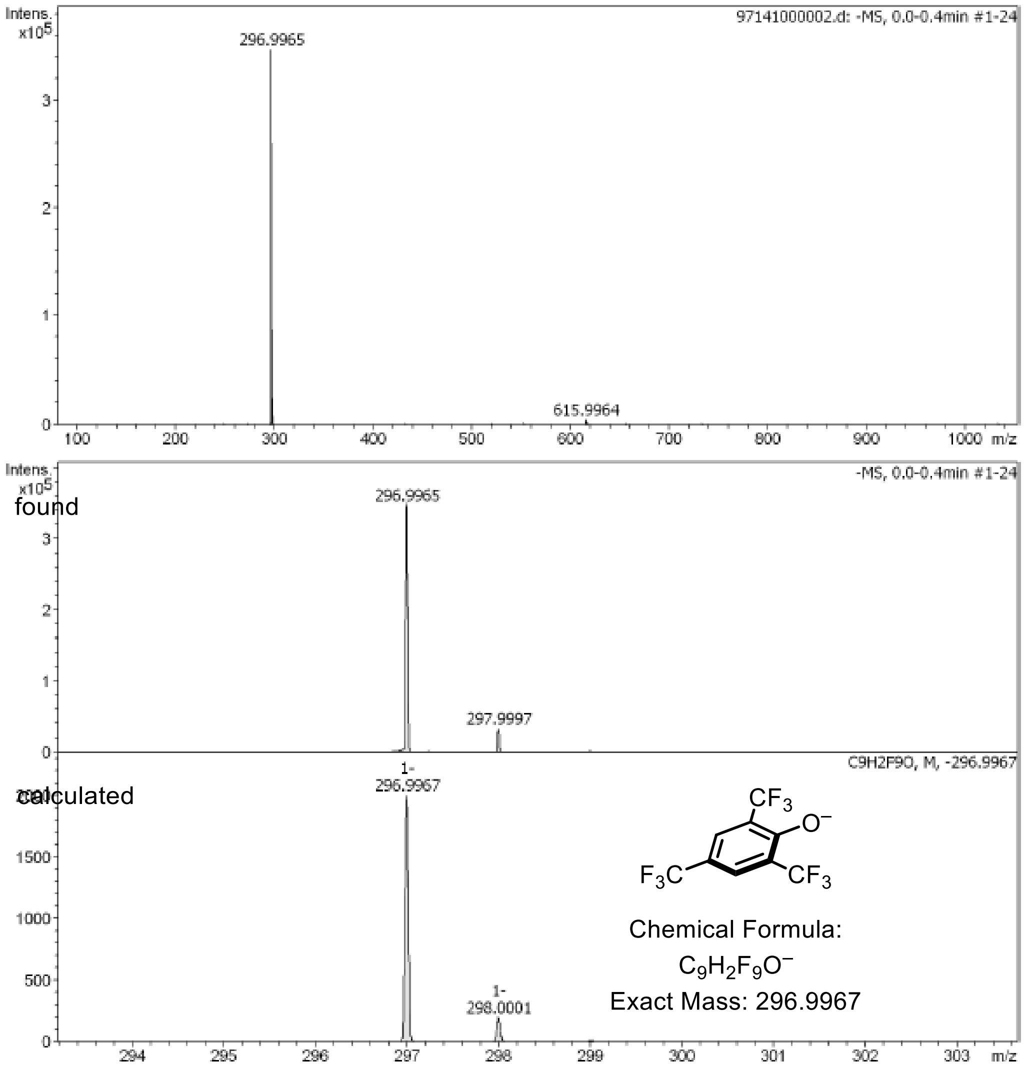

Intens.
x10^5
97141000002.d: -MS, 0.0-0.4min #1-24
296.9965
615.9964
m/z
-MS, 0.0-0.4min #1-24
found
297.9997
C9H2F9O, M, -296.9967
calculated
1-
296.9967
1-
298.0001
CF3
O−
F3C
CF3
Chemical Formula:
C9H2F9O−
Exact Mass: 296.9967

5. Application

5.1 Performance comparison in late-stage functionalization settings

(4-(8-Chloro-5,6-dihydro-11H-benzo[5,6]cyclohepta[1,2-b]pyridin-11-ylidene)piperidin-1-yl)(phenyl)methanone (13a)

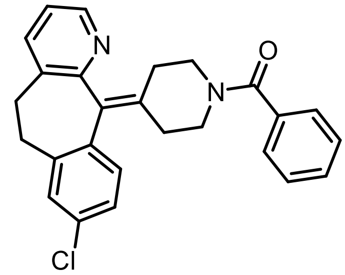

Following the GP1 (60 µmol scale), the titled compound was obtained as a yellow oil (23.2 mg, 56 µmol, 93%).

^{1}H NMR (400 MHz, CDCl$_3$): δ 8.49 – 8.25 (m, 1H), 7.47 – 7.31 (m, 6H), 7.20 – 7.02 (m, 4H), 4.26 – 4.04 (m, 1H), 3.69 – 3.50 (m, 1H), 3.45 – 3.10 (m, 4H), 2.92 – 2.77 (m, 2H), 2.62 – 2.20 (m, 4H) ppm.
^{13}C NMR (101 MHz, CDCl$_3$): δ 170.6, 156.9, 146.8, 139.7, 137.8, 136.9, 136.0, 134.9, 133.5, 133.2, 130.6, 129.8, 129.1, 128.5, 126.9, 126.3, 122.5, 48.7, 43.4, 31.8, 31.6, 31.4, 30.6 ppm.

All NMR data were in good accordance with the literature.[7]

(4-(8-Chloro-5,6-dihydro-11H-benzo[5,6]cyclohepta[1,2-b]pyridin-11-ylidene)piperidin-1-yl)(pyridin-2-yl)methanone (13b)

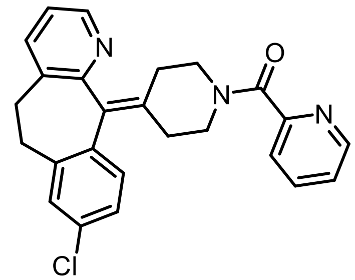

Following the GP1 (60 µmol scale) but with a reaction temperature of 100 °C, the titled compound was obtained as a white solid (24.0 mg, 58 µmol, 96%).

^{1}H NMR (400 MHz, CDCl$_3$): δ 8.54 (d, *J* = 4.7 Hz, 1H), 8.38 (dd, *J* = 30.6, 3.6 Hz, 1H), 7.77 (td, *J* = 7.7, 1.6 Hz, 1H), 7.62 (dd, *J* = 7.7, 2.7 Hz, 1H), 7.48 – 7.42 (m, 1H), 7.33 – 7.26 (m, 1H), 7.20 – 7.04 (m, 4H), 4.28 – 4.13 (m, 1H), 3.83 – 3.67 (m, 1H), 3.47 – 3.24 (m, 4H), 2.94 – 2.77 (m, 2H), 2.63 – 2.29 (m, 4H) ppm.
^{13}C NMR (101 MHz, CDCl$_3$): δ 167.8, 167.7, 157.1, 156.9, 154.4, 148.5, 148.5, 146.8, 146.6, 139.7, 139.6, 137.9, 137.8, 137.5, 137.4, 137.3, 137.2, 134.5, 133.7, 133.6, 133.2, 133.1, 130.7, 130.6, 129.2, 129.1, 126.4, 126.3, 124.5, 124.5, 123.8, 123.7, 122.5, 122.5, 48.2, 48.1, 43.7, 31.8, 31.8, 31.6, 31.6, 31.4, 30.5, 30.3 ppm. (due to romateric effects, more carbon peaks have been observed)

All NMR data were in good accordance with the literature.[8]

(4-(8-Chloro-5,6-dihydro-11H-benzo[5,6]cyclohepta[1,2-b]pyridin-11-ylidene)piperidin-1-yl)(4-(dimethylamino)phenyl)methanone (13c)

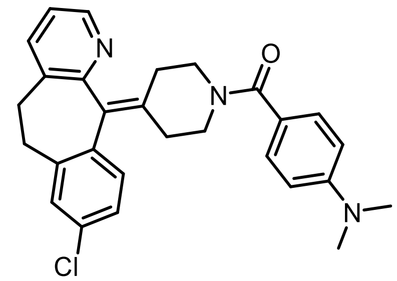

Following the GP1 (60 µmol scale), the titled compound was obtained as a white solid (17.0 mg, 37 µmol, 62%).

^{1}H NMR (400 MHz, CDCl$_3$): δ 8.40 – 8.36 (m, 1H), 7.43 (dd, *J* = 7.7, 1.5 Hz, 1H), 7.35 – 7.30 (m, 2H), 7.18 – 7.05 (m, 4H), 6.65 – 6.59 (m, 2H), 4.03 – 3.85 (m, 2H), 3.44 – 3.20 (m, 4H), 2.96 (s, 5H), 2.87 – 2.76 (m, 2H), 2.58 – 2.48 (m, 1H), 2.45 – 2.29 (m, 3H) ppm.
^{13}C NMR (101 MHz, CDCl$_3$): δ 171. 1, 157.1, 151.6, 146.8, 139.7, 137.7, 137.7, 137.6, 134.5, 133.5, 133.1, 130.7, 129.2, 129.1, 126.3, 122.7, 122.4, 111.3, 53.9, 53.6, 40.3, 31.8, 31.6, 31.2, 29.4 ppm.

All NMR data were in good accordance with the literature.[8]

((3R,4S)-3-((benzo[d][1,3]dioxol-5-yloxy)methyl)-4-(4-fluorophenyl)piperidin-1-yl)(phenyl)methanone (13d)

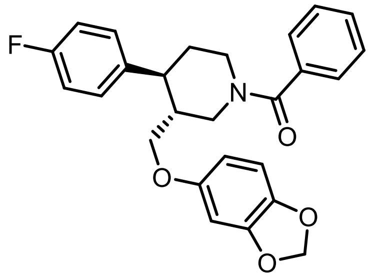

Following the GP1 (0.1 mmol scale), the titled compound was obtained as a colorless amorphous solid (33.3 mg, 77 µmol, 77%). The desired amide was obtained as a mixture of two rotamers in a ratio of around 1.2:1.

^{1}H NMR (700 MHz, CDCl$_3$): δ 7.48 – 7.36 (m, 5H), 7.19 – 7.07 (m, 2H), 7.03 – 6.94 (m,, 2H), 6.70 – 6.55 (m, 1H), 6.43 – 5.94 (m, 2H), 5.88 (s, 2H), 5.06 – 4.76 (m, 1H), 4.17 – 3.84 (m, 1H), 3.73 – 3.29 (m, 2H), 3.22 – 2.66 (m, 3H), 2.18 – 1.62 (m, 3H) ppm.
^{13}C NMR (176 MHz, CDCl$_3$): δ 170.7, 161.8 (d, *J* = 245.2 Hz), 154.4, 153.8, 148.3, 141.9, 138.6, 135.9, 129.9, 128.9, 128.6, 127.1, 115.8 (d, *J* = 21.1 Hz), 108.0, 105.7, 105.6, 101.3, 98.2, 98.0, 68.6, 68.4, 51.3, 48.4, 45.6, 44.4, 43.9, 43.0, 42.1, 34.7, 33.7 ppm.
^{19}F NMR (659 MHz, CDCl$_3$): δ -115.70, -115.86 ppm.

All NMR data were in good accordance with the literature.[9]

1-((3R,4S)-3-((benzo[d][1,3]dioxol-5-yloxy)methyl)-4-(4-fluorophenyl)piperidin-1-yl)-2-(4-bromophenyl)ethan-1-one (13e)

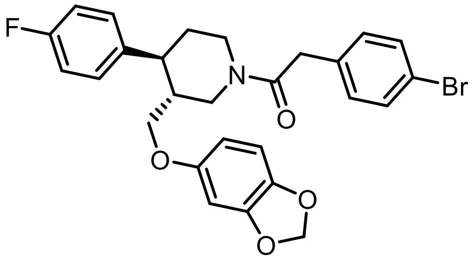

Following the GP1 (0.1 mmol scale), the titled compound was obtained as a colorless amorphous solid (45.2 mg, 86 µmol, 86%). The desired amide was obtained as a mixture of two rotamers in a ratio of around 1.4:1. NMR peaks in ^{1}H and ^{13}C NMR corresponding exclusively to the minor rotamer are indicated with an asterisk.

^{1}H NMR (700 MHz, CDCl$_3$): δ 7.49 – 7.40 (m, 2H), 7.20 – 7.14 (m, 2H), 7.07 – 7.02 (m, 2H), 7.00 – 6.94 (m, 2H), 6.64 (d, *J* = 8.5 Hz, 0.6H), 6.61 (d, *J* = 8.5 Hz, 0.4H*), 6.34 (d, *J* = 2.4 Hz, 0.4H*), 6.31 (d, *J* = 2.4 Hz, 0.6H), 6.12 (dd, *J* = 8.5, 2.4 Hz, 0.4H*), 6.09 (dd, *J* = 8.5, 2.4 Hz, 0.6H), 5.90 (s, 1.1H), 5.87 (s, 0.9H*), 4.92 (dd, *J* = 13.3, 2.0 Hz, 0.4H*), 4.80 – 4.75 (m, 0.6H), 4.22 (dd, *J* = 13.6, 1.5 Hz, 0.6H), 3.95 (d, *J* = 13.6 Hz, 0.4H*), 3.77 – 3.70 (m, 2H), 3.59 (dd, *J* = 9.3, 2.1 Hz, 0.4H*), 3.50 (dd, *J* = 9.6, 2.9 Hz, 0.6H), 3.46 – 3.35 (m, 1H), 3.14 – 2.98 (m, 1H), 2.84 – 2.57 (m, 2H), 1.95 – 1.71 (m, 2H), 1.66 – 1.57 (m, 0.6H), 1.48 – 1.41 (m, 0.4H*) ppm.
^{13}C NMR (176 MHz, CDCl$_3$): δ 169.3, 169.2*, 161.84 (d, *J* = 245.2 Hz), 161.78* (d, *J* = 245.0 Hz), 154.3*, 154.0, 148.4, 148.3*, 142.0, 141.9*, 138.5, 138.4*, 134.2*, 134.2, 131.9, 130.61, 130.56*, 128.9 (d, *J* = 7.7 Hz), 128.7 (d, *J* = 7.8 Hz), 121.0, 115.9, 115.8, 115.7, 108.1, 108.0*, 105.7*, 105.5, 101.4, 101.2*, 98.1*, 97.9, 68.7, 68.5*, 49.8, 46.8, 45.4, 44.3, 43.7, 42.8, 42.5, 41.9, 40.6, 40.5, 34.3, 33.6 ppm.
^{19}F NMR (659 MHz, CDCl$_3$): δ -115.59, -115.77* ppm.

All NMR data were in good accordance with the literature.[10]

5.2 Deprotective amidation applied in medicinal chemistry

Synthesis route of piperine-derived Alzheimer drugs:

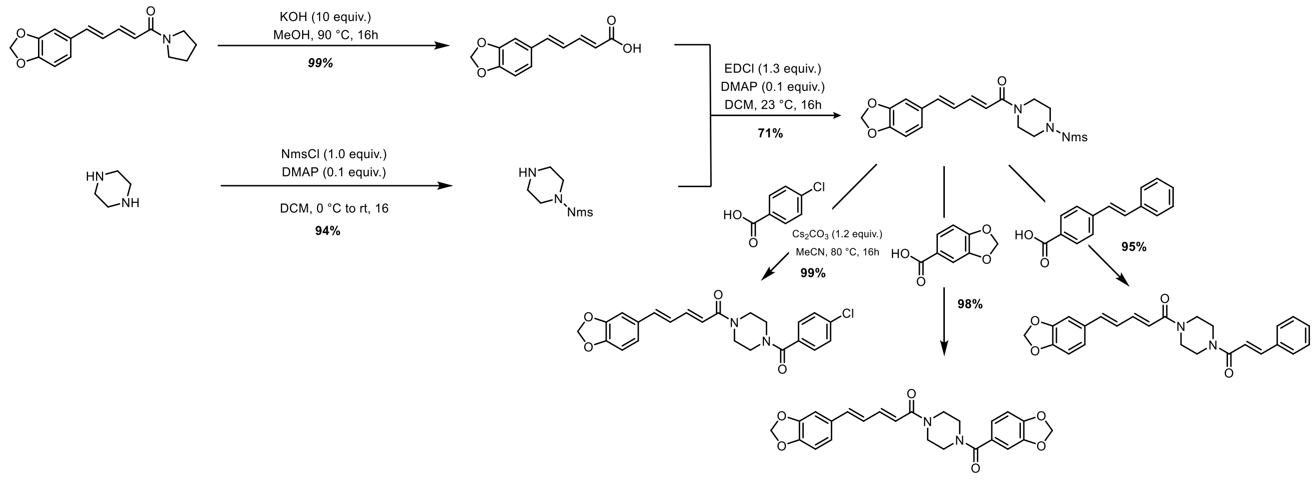

Piperic acid

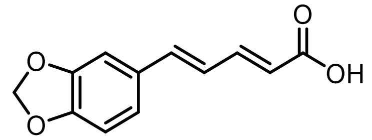

Was prepared in 99% yield according to literature.[11]

^{1}H NMR (400 MHz, d$_6$-DMSO): δ 7.35 – 7.25 (m, 1H), 7.22 (d, *J* = 1.2 Hz, 1H), 7.03 – 6.89 (m, 4H), 6.03 (s, 2H), 5.93 (d, *J* = 15.2 Hz, 1H) ppm.
^{13}C NMR (101 MHz, d$_6$-DMSO): δ 167.9, 148.3, 148.1, 144.8, 140.0, 130.7, 125.0, 123.3, 121.3, 108.7, 105.9, 101.5 ppm.

1-((2,4,6-Tris(trifluoromethyl)phenyl)sulfonyl)piperazine (S9)

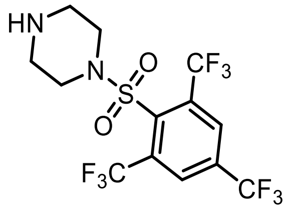

Piperazine (396 mg, 4.59 mmol, 5.0 equiv.) was dissolved in DCM (0.1M) at room temperature. DMAP (9.4 mg, 77 µmol, 10 mol%), was added at this temperature. Then, the mixture was cooled to 0 °C and the NmsCl (291 mg, 0.77 mmol, 1.0 equiv.) was added in one portion. The reaction was allowed to slowly warm up to room temperature over 15 h (without removing the cooling bath). Then, the solvents were removed under vacuum and the residue was purified by column chromatography (silica gel, DCM/MeOH, 9:1) to provide the titled compound as a white solid (310 mg, 0.72 mmol, 94%).

^{1}H NMR (700 MHz, CDCl$_3$): δ 8.26 (s, 2H), 3.41 – 3.30 (m, *J* = 13.6, 8.7 Hz, 4H), 2.99 – 2.91 (m, 3H), 2.60 (app s, 1H), 1.66 (app s, 1H) ppm.

^{13}C NMR (176 MHz, CDCl$_3$): δ 145.3, 145.2, 134.2 (q, *J* = 35.2 Hz), 133.2 (q, *J* = 33.9 Hz), 133.1 (q, *J* = 33.9 Hz), 129.4 (s), 122.19 (q, *J* = 275.6 Hz), 122.0 (q, *J* = 273.8 Hz), 51.3, 47.9, 47.0, 46.1 ppm.
^{19}F NMR (659 MHz, CDCl$_3$): δ -55.39, -55.40, -63.58 ppm.
IR (neat): $ν_{max}$ 1281, 1187, 1167, 1145, 1134, 1119, 950, 726 cm^{-1}.
HRMS (ESI$^+$): exact mass calculated for $[M+H]^+$ $(C_{13}H_{12}F_9N_2O_2S)^+$ requires *m/z* 431.0470, found *m/z* 431.0468.

(2*E*,4*E*)-5-(Benzo[d][1,3]dioxol-5-yl)-1-(4-((2,4,6-tris(trifluoromethyl)phenyl)sulfonyl)piperazin-1-yl)penta-2,4-dien-1-one (14)

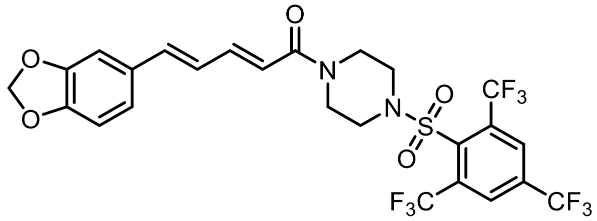

A mixture of piperic acid (144 mg, 0.66 mmol, 1.1 equiv.) and **S9** (0.6 mmol, 258 mg, 1.0 equiv.) dissolved in DCM (3 mL) was cooled to 0 °C. Then 1-(3-dimethylaminopropyl)-3-ethylcarbodiimide hydrochloride (150 mg, 0.78 mmol, 1.3 equiv.) was added, followed by 4-dimethylaminopyridine (14.7 mg, 0.12 mmol, 0.2 equiv.). The mixture was allowed to warm slowly to room temperature for 15 hours. 1M HCl was added and the suspension was extracted with EtOAc. The organic phase was extracted again with brine. The organic phase was dried with $MgSO_4$ and the solvent was removed under reduced pressure. The crude material was purified by column chromatography to give **14** as a white solid (270 mg, 0.43 mmol, 71 %).

^{1}H NMR (600 MHz, CDCl$_3$): δ 8.29 (s, 2H), 7.46 (dd, *J* = 14.5, 10.9 Hz, 1H), 6.98 (d, *J* = 1.5 Hz, 1H), 6.91 (dd, *J* = 8.1, 1.5 Hz, 1H), 6.80 (dd, *J* = 14.9, 11.7 Hz, 2H), 6.73 (dd, *J* = 15.4, 10.9 Hz, 1H), 6.34 (d, *J* = 14.6 Hz, 1H), 5.98 (s, 2H), 3.94 – 3.59 (m, 4H), 3.40 (s, 4H) ppm.
^{13}C NMR (151 MHz, CDCl$_3$): δ 165.9, 148.6, 148.4, 144.7, 144.4, 139.9, 134.6 (q, *J* = 35.3 Hz), 133.1 (q, *J* = 34.0 Hz), 130.8, 129.5, 124.9, 123.0, 122.1 (q, *J* = 274.0 Hz), 121.9 (q, *J* = 273.8 Hz)118.3, 108.7, 105.9, 47.0, 46.1, 42.1 ppm.
^{19}F NMR (565 MHz, CDCl$_3$): δ -55.43 (6F), -63.58 (3F) ppm.
IR (neat): $ν_{max}$ 1644, 1595, 1442, 1358, 1286, 1188, 1166 1146, 1121, 913, 725 cm^{-1}.
HRMS (ESI$^+$): exact mass calculated for $[M+Na]^+$ $(C_{25}H_{19}F_9N_2O_5SNa)^+$ requires *m/z* 653.0763, found *m/z* 653.0765.

(2*E*,4*E*)-5-(Benzo[d][1,3]dioxol-5-yl)-1-(4-(4-chlorobenzoyl)piperazin-1-yl)penta-2,4-dien-1-one (16a)

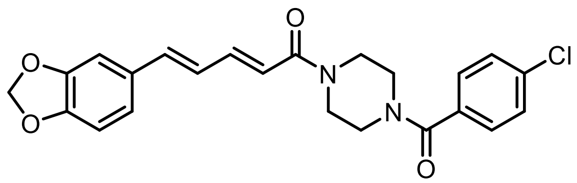

Following the GP1 (0.1 mmol scale), the titled compound was obtained as a white solid (42.0 mg, 99 µmol, 99%). The same reaction carried out with K_2CO_3 at 100 °C instead of Cs_2CO_3 resulted in a yield of 92 %.

^{1}H NMR (400 MHz, CDCl$_3$): δ 7.44 (dd, *J* = 14.6, 10.2 Hz, 1H), 7.40 – 7.31 (m, 4H), 6.95 (d, *J* = 1.5 Hz, 1H), 6.86 (dd, *J* = 8.1, 1.6 Hz, 1H), 6.80 – 6.68 (m, 3H), 6.33 (app d, *J* = 14.3 Hz, 1H), 5.97 (s, 2H), 3.87 – 3.31 (m, *J* = 72.1 Hz, 8H) ppm.
^{13}C NMR (101 MHz, CDCl$_3$): δ 169.7, 166.0, 148.6, 148.4, 144.4, 139.8, 136.4, 133.4, 130.7, 129.0, 128.8, 124.8, 123.0, 118.2, 108.6, 105.8, 101.5, 47.6, 45.6, 42.4 ppm.

All NMR data were in good agreement with the literature.[11]

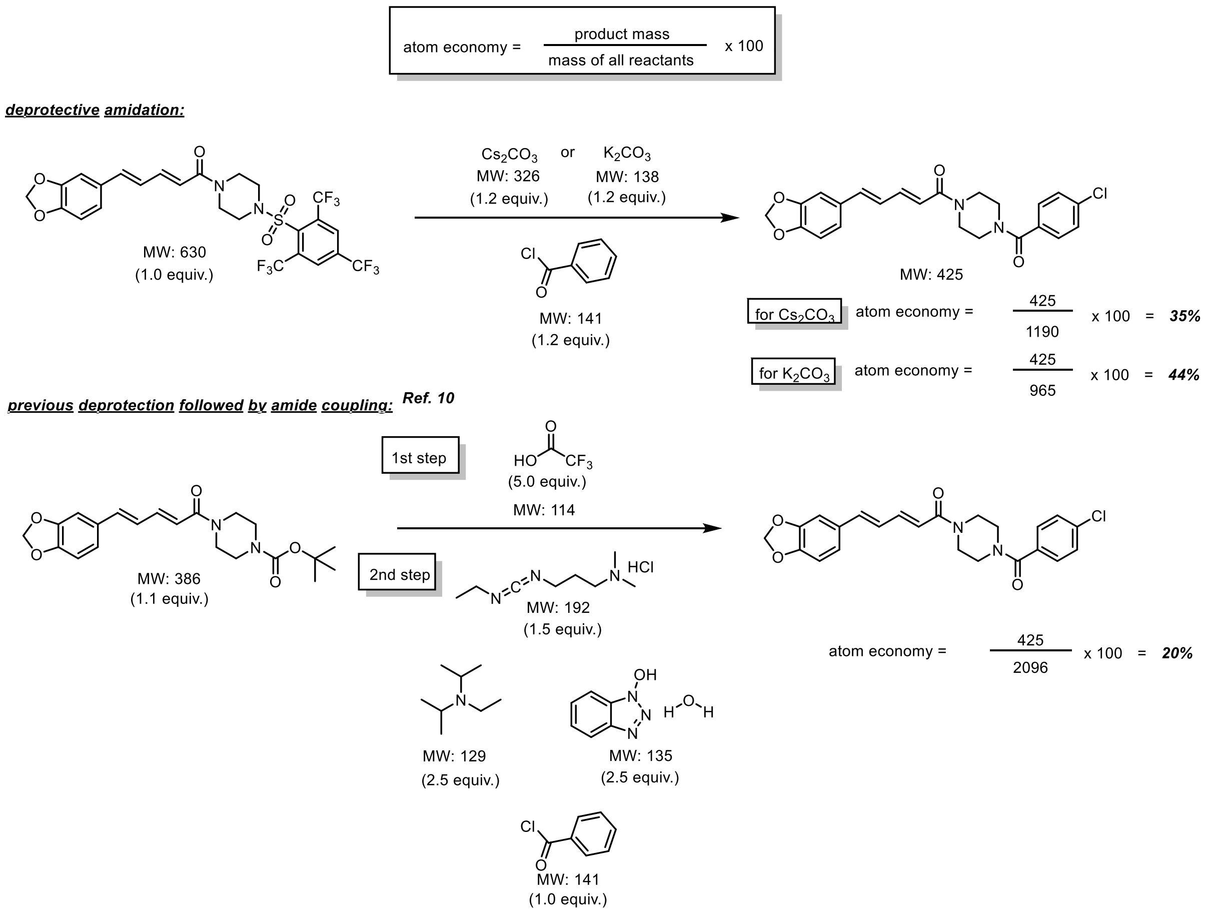

Figure S1: Atom economy calculation for the synthesis of **16**.

(2*E*,4*E*)-5-(Benzo[d][1,3]dioxol-5-yl)-1-(4-cinnamoylpiperazin-1-yl)penta-2,4-dien-1-one (16b)

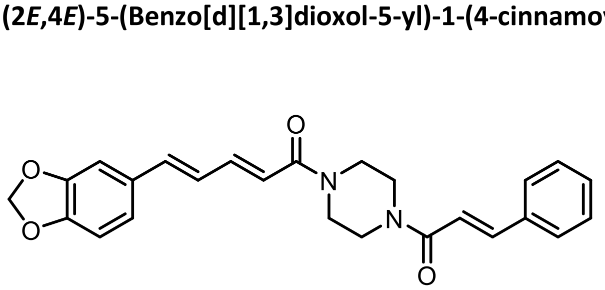

Following the GP1 (60 µmol scale), the titled compound was obtained as a white solid (23.8 mg, 57 µmol, 95%).

^{1}H NMR (400 MHz, CDCl$_3$): δ 7.71 (s, 1H), 7.51 – 7.42 (m, 3H), 7.41 – 7.34 (m, 3H), 6.96 (d, *J* = 1.5 Hz, 1H), 6.87 (dd, *J* = 8.1, 1.6 Hz, 1H), 6.84 – 6.70 (m, 4H), 6.34 (d, *J* = 14.6 Hz, 1H), 5.97 (s, 2H), 3.83 – 3.53 (m, 8H) ppm.

^{13}C NMR (101 MHz, CDCl$_3$): δ 166.0, 165.9, 148.6, 148.4, 144.3, 143.9, 139.8, 135.1, 130.8, 130.1, 129.0, 128.0, 124.9, 123.0, 118.4, 116.4, 108.7, 105.8, 101.5, 45.6, 42.2 ppm.

All NMR data were in good agreement with the literature.[11]

(2*E*,4*E*)-5-(Benzo[d][1,3]dioxol-5-yl)-1-(4-(benzo[d][1,3]dioxole-5-carbonyl)piperazin-1-yl)penta-2,4-dien-1-one (16c)

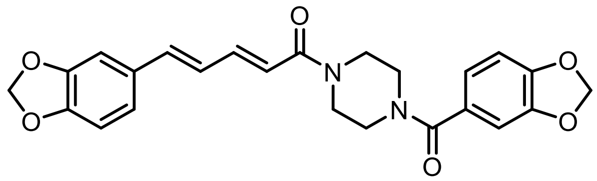

Following the GP1 (60 µmol scale), the titled compound was obtained as a white solid (25.3 mg, 58 µmol, 97%).

^{1}H NMR (400 MHz, CDCl$_3$): δ 7.44 (dd, *J* = 14.6, 10.2 Hz, 1H), 7.00 – 6.74 (m, 8H), 6.36 (d, *J* = 14.6 Hz, 1H), 6.01 (s, 2H), 5.98 (s, 2H), 3.64 (app s, 8H) ppm.
^{13}C NMR (101 MHz, CDCl$_3$): δ 170.3, 166.0, 149.3, 148.5, 148.4, 147.9, 144.1, 139.6, 130.9, 128.8, 124.9, 122.9, 121.9, 118.6, 108.7, 108.4, 108.2, 105.8, 101.7, 101.5, 45.7, 42.3 ppm.

All NMR data were in good agreement with the literature.[11]

6. DFT calculations

The conformational space of all molecules has been initially searched using meta-dynamics simulations based on semiempirical tight-binding quantum chemical calculations as implemented in CREST.[12,13]

Structures located with CREST have then been subjected to wB97XD/def2-SVP[14–16] single point calculation. Structures within a window of 6 kcal mol^{-1} to the structure with the lowest energy have been subjected to geometry optimization at the same level of theory. The nature of all stationary points (minima and transition states) was verified through the computation of the vibrational frequencies. The thermal corrections to the Gibbs free energies were combined with the single point energies calculated at the wB97XD /def2-TZVP level of theory to yield Gibbs free energies (“G_{298}”) at 298.15 K. All energies are reported in kcal mol^{-1}. The energy profiles were constructed using the most stable conformation (the global minimum) of each intermediate and transition state.

The DFT calculations have been performed with the Gaussian 16 program package.[17] The polarizable continuum model (PCM) with SMD parameters[18,19] of acetonitrile was applied to consider solvent effects for both geometries and energies.

Free energies in solution have been corrected to a reference state of 1 mol l^{-1} at 298.15 K through the addition of RTln(24.46) = +7.925 kJ mol^{-1} to the gas phase (1 atm) free energies.

6.1 XYZ structures

Cartesian coordinates of the most stable (ΔG_{298}) conformations, computed at the wB97XD/def2-TZVP-SMD//wB97XD/def2-SVP-SMD level of theory.

```
32
NMs
C        0.917147000      0.246027000     -0.029237000
S       -0.609783000      1.227874000      0.240631000
C       -0.571694000      3.891432000      0.437692000
H        0.216557000      4.650888000      0.539166000
H       -1.450169000      4.201840000      1.028053000
N       -0.044206000      2.616149000      0.903105000
O       -1.351430000      0.487221000      1.250104000
O       -1.220041000      1.522813000     -1.043315000
H       -0.855866000      3.818855000     -0.617125000
C        0.862982000     -1.154456000      0.133153000
C        2.156921000      0.842012000     -0.326808000
C        2.040819000     -1.897175000      0.158415000
C        3.324167000      0.076963000     -0.289653000
C        3.271066000     -1.276831000     -0.006558000
H        4.283864000      0.551016000     -0.492043000
C        2.370091000      2.289101000     -0.762119000
F        3.448541000      2.367455000     -1.552989000
F        1.353444000      2.765363000     -1.475750000
F        2.600052000      3.116057000      0.259387000
C        4.531066000     -2.107969000      0.038299000
F        4.514298000     -2.961067000      1.066249000
F        5.623600000     -1.353390000      0.159050000
F        4.671161000     -2.838077000     -1.073739000
H        1.992897000     -2.976842000      0.302421000
C       -0.416457000     -1.986795000      0.212368000
F       -0.866892000     -2.143386000      1.453477000
F       -1.392485000     -1.488714000     -0.544428000
F       -0.178528000     -3.220866000     -0.257174000
C        0.361434000      2.545952000      2.299684000
H       -0.499955000      2.612923000      2.984469000
H        0.903496000      1.610998000      2.498652000
H        1.046259000      3.381305000      2.497960000


14
I
O       -0.447983000      0.101223000     -2.191679000
C       -1.618173000      0.496321000     -2.016370000
O       -2.067589000      1.654609000     -2.128501000
C       -2.643099000     -0.588919000     -1.607652000
C       -2.235358000     -1.908477000     -1.382717000
C       -3.997406000     -0.271623000     -1.455125000
C       -3.155749000     -2.889658000     -1.011338000
C       -4.925479000     -1.247673000     -1.089396000
C       -4.505677000     -2.561095000     -0.864914000
H       -1.175457000     -2.142578000     -1.506677000
H       -4.301696000      0.762549000     -1.632490000
H       -2.822053000     -3.916111000     -0.835249000
H       -5.981383000     -0.985929000     -0.977497000
H       -5.229682000     -3.327701000     -0.576207000


12
II-1
O        3.168183000      0.713402000     -0.036847000
C        2.831661000     -0.544149000      0.040098000
O        1.579765000     -0.843657000      0.111023000
O        3.731614000     -1.445548000      0.042582000
O       -3.167171000     -0.717976000     -0.001815000
C       -2.830673000      0.539531000     -0.076691000
O       -1.578863000      0.841143000     -0.140603000
O       -3.730756000      1.441180000     -0.084606000
K       -0.756372000     -1.681875000     -0.049524000
K        0.758157000      1.677996000      0.013949000
K       -5.650063000     -0.187536000      0.069183000
K        5.648636000      0.191484000     -0.120080000


7
II-2
O        1.410844000     -0.297617000     -1.549309000
C        1.826627000     -1.406753000     -1.040209000
O        1.494716000     -1.728319000      0.163455000
O        2.574092000     -2.194699000     -1.735054000
K        0.116502000      0.397414000      0.552536000
K        2.778613000     -3.948045000      0.122518000
K        2.574927000     -0.677000000     -3.801372000


46
IA
C       -0.425489000      1.068828000      0.592818000
O        1.554995000      0.214654000     -1.866633000
S        0.305071000      2.626160000      1.219830000
C        0.070523000      4.958803000     -0.050213000
H       -0.273300000      5.263644000     -1.049143000
H        0.906144000      5.611115000      0.256112000
N        0.482737000      3.564298000     -0.109433000
O        1.621538000      2.245062000      1.713248000
O       -0.649393000      3.256484000      2.117694000
H       -0.755202000      5.080493000      0.658110000
C       -0.102031000     -0.137291000      1.247851000
C       -1.273926000      1.020664000     -0.528583000
C       -0.494134000     -1.353958000      0.693211000
C       -1.652775000     -0.210235000     -1.062140000
C       -1.239884000     -1.391697000     -0.474106000
H       -2.285242000     -0.241827000     -1.947567000
C       -1.914037000      2.225220000     -1.209300000
F       -2.238794000      3.185688000     -0.343674000
F       -1.162888000      2.758855000     -2.173506000
F       -3.062449000      1.859902000     -1.798242000
C       -1.694559000     -2.720160000     -1.023383000
F       -0.745259000     -3.652562000     -0.939609000
F       -2.090987000     -2.643770000     -2.290818000
F       -2.743479000     -3.181572000     -0.316927000
H       -0.225875000     -2.283748000      1.194213000
C        0.580553000     -0.249208000      2.610284000
F        1.908784000     -0.272282000      2.554952000
F        0.206836000      0.724801000      3.441226000
F        0.216720000     -1.397431000      3.204030000
C        1.598887000      3.242764000     -0.990178000
H        2.555528000      3.620147000     -0.590626000
H        1.660792000      2.157504000     -1.167133000
H        1.405251000      3.724881000     -1.958412000
C        1.478527000     -1.017991000     -2.060044000
O        0.747207000     -1.615966000     -2.872835000
C        2.384011000     -1.897877000     -1.178701000
C        3.129155000     -1.335220000     -0.138100000
C        2.467575000     -3.277152000     -1.397425000
C        3.934771000     -2.132308000      0.675926000
C        3.277478000     -4.079557000     -0.593672000
```

C	4.012368000	-3.507940000	0.448222000
H	3.048010000	-0.259211000	0.027611000
H	1.875480000	-3.703087000	-2.209862000
H	4.501937000	-1.680997000	1.494386000
H	3.335658000	-5.156224000	-0.775795000
H	4.643687000	-4.135004000	1.083339000

37

IIA

C	-0.135722000	1.047848000	0.631187000
O	5.040880000	1.371392000	5.145430000
S	1.509550000	1.838513000	0.530360000
C	2.055068000	3.870558000	-1.106143000
H	1.540895000	4.342969000	-1.954444000
H	3.139223000	3.848987000	-1.303950000
N	1.519720000	2.522491000	-0.947957000
O	2.461663000	0.729493000	0.567465000
O	1.630289000	2.871239000	1.551705000
H	1.862988000	4.463015000	-0.205894000
C	-0.267875000	-0.175516000	1.322939000
C	-1.262405000	1.584162000	-0.018830000
C	-1.447999000	-0.906048000	1.213904000
C	-2.430606000	0.826875000	-0.120725000
C	-2.507121000	-0.429513000	0.454308000
H	-3.290280000	1.235069000	-0.651344000
C	-1.371976000	2.994496000	-0.593348000
F	-2.641267000	3.411855000	-0.522604000
F	-0.649893000	3.879160000	0.090148000
F	-1.027703000	3.064788000	-1.879697000
C	-3.766090000	-1.256613000	0.341974000
F	-3.482867000	-2.537534000	0.092362000
F	-4.563557000	-0.822319000	-0.633788000
F	-4.469970000	-1.223708000	1.478224000
H	-1.540142000	-1.859012000	1.735215000
C	0.757635000	-0.766938000	2.289838000
F	1.667835000	-1.535409000	1.706389000
F	1.390799000	0.190088000	2.987200000
F	0.139660000	-1.528551000	3.196503000
C	1.674996000	1.614179000	-2.078606000
H	2.730790000	1.349167000	-2.248590000
H	1.094720000	0.694603000	-1.919842000
H	1.277272000	2.114764000	-2.971099000
C	6.178858000	0.921385000	4.690732000
O	7.130655000	0.647432000	5.477481000
O	6.307428000	0.761204000	3.401334000
K	3.968632000	1.452750000	2.893268000

46

TSIAC

C	0.479304000	0.628099000	-0.154781000
O	-1.322929000	-0.044517000	0.060914000
S	-0.200990000	2.273147000	-0.733824000
C	-0.517137000	4.355573000	0.900821000
H	-0.781963000	4.516454000	1.955591000
H	-0.996889000	5.141832000	0.290909000
N	-0.968618000	3.027467000	0.513815000
O	-1.161750000	2.058011000	-1.806715000
O	1.022794000	3.023240000	-1.009133000
H	0.568712000	4.437523000	0.797175000
C	1.089941000	-0.144831000	-1.210806000
C	1.122144000	0.554544000	1.129721000
C	2.170577000	-0.970785000	-0.950641000
C	2.210450000	-0.283479000	1.331980000
C	2.758634000	-1.038892000	0.307404000
H	2.647560000	-0.346410000	2.326874000
C	0.603357000	1.221703000	2.384233000
F	0.953539000	2.516257000	2.489123000
F	-0.721513000	1.150274000	2.527885000
F	1.111183000	0.638006000	3.487455000
C	3.948680000	-1.915763000	0.521952000
F	3.714180000	-3.190014000	0.161604000
F	4.344219000	-1.940831000	1.800620000
F	5.012523000	-1.522362000	-0.203357000
H	2.581317000	-1.573556000	-1.760261000
C	0.542661000	-0.222296000	-2.622373000
F	-0.760637000	-0.506440000	-2.675231000
F	0.746023000	0.893997000	-3.338077000
F	1.150372000	-1.200898000	-3.321840000
C	-2.390974000	2.773066000	0.682266000
H	-3.002141000	3.411866000	0.020939000
H	-2.604839000	1.717486000	0.483566000
H	-2.656037000	2.990397000	1.726918000
C	-1.454784000	-1.295546000	0.309997000
O	-0.569331000	-2.077693000	0.639763000
C	-2.882550000	-1.795264000	0.159733000
C	-3.851014000	-1.030188000	-0.499613000
C	-3.229942000	-3.050544000	0.668986000
C	-5.151933000	-1.512463000	-0.642324000
C	-4.532431000	-3.530058000	0.536794000
C	-5.495657000	-2.761161000	-0.120251000
H	-3.565270000	-0.057729000	-0.905562000
H	-2.460987000	-3.642547000	1.170148000
H	-5.901990000	-0.912494000	-1.163754000
H	-4.798604000	-4.508526000	0.944559000
H	-6.516080000	-3.137483000	-0.227981000

46

IC

C	-0.214579000	-0.624263000	-0.177103000
O	1.120849000	-0.868239000	-0.090955000
S	1.273314000	2.123597000	1.629585000
C	-0.879646000	3.001673000	0.334964000
H	-1.428834000	3.090396000	-0.617780000
H	-0.985691000	3.963652000	0.880550000
N	0.493021000	2.628410000	0.096342000
O	2.688660000	1.877830000	1.155210000
O	1.209209000	3.359606000	2.487602000
H	-1.381671000	2.221376000	0.931698000
C	-1.010633000	-1.036552000	0.895844000
C	-0.782158000	0.067866000	-1.250255000
C	-2.369099000	-0.742047000	0.912076000
C	-2.146291000	0.343427000	-1.231004000
C	-2.933437000	-0.051551000	-0.154560000
H	-2.594598000	0.891499000	-2.061494000
C	-0.004042000	0.529056000	-2.462757000
F	-0.434460000	1.725165000	-2.878961000
F	1.308705000	0.625843000	-2.269096000
F	-0.183982000	-0.316895000	-3.488634000
C	-4.382139000	0.355562000	-0.121709000
F	-5.093853000	-0.371485000	0.742567000

F	-4.961240000	0.233202000	-1.320589000
F	-4.514822000	1.639789000	0.242394000
H	-2.982762000	-1.054976000	1.756482000
C	-0.383591000	-1.813681000	2.031167000
F	0.220562000	-2.925866000	1.585858000
F	0.534012000	-1.108953000	2.691179000
F	-1.300256000	-2.200298000	2.922277000
C	1.242808000	3.637545000	-0.612215000
H	1.184616000	4.641246000	-0.139079000
H	2.299260000	3.331936000	-0.637001000
H	0.878013000	3.731033000	-1.648721000
C	1.698674000	-1.816358000	-0.878410000
O	1.056831000	-2.510680000	-1.618085000
C	3.164807000	-1.858734000	-0.673016000
C	3.827883000	-0.850076000	0.039737000
C	3.877377000	-2.929057000	-1.228692000
C	5.211417000	-0.930974000	0.193039000
C	5.256679000	-3.001923000	-1.062041000
C	5.923439000	-2.001806000	-0.349913000
H	3.275377000	0.005187000	0.454573000
H	3.341049000	-3.700546000	-1.785123000
H	5.738294000	-0.146952000	0.742158000
H	5.814712000	-3.838430000	-1.488262000
H	7.007149000	-2.057543000	-0.220553000

37
TSIIAB

C	0.202061000	-0.038809000	0.541219000
O	-0.829068000	-0.573333000	2.447826000
S	-1.279324000	0.813139000	-0.235437000
C	-2.992941000	1.465811000	1.713116000
H	-2.607998000	0.923020000	2.589057000
H	-4.082218000	1.599538000	1.786470000
N	-2.731927000	0.703309000	0.501404000
O	-1.406553000	0.153702000	-1.530240000
O	-0.894180000	2.221206000	-0.221188000
H	-2.531171000	2.455573000	1.641761000
C	0.664952000	-1.251407000	-0.032608000
C	1.190351000	0.814839000	1.114576000
C	2.016948000	-1.586503000	-0.007650000
C	2.535910000	0.474484000	1.087797000
C	2.963821000	-0.724857000	0.522545000
H	3.267730000	1.142138000	1.547628000
C	0.917052000	2.048966000	1.956651000
F	1.320324000	3.178082000	1.365865000
F	-0.327592000	2.215584000	2.361058000
F	1.651257000	1.955672000	3.101212000
C	4.405164000	-1.122590000	0.620802000
F	4.691994000	-1.592934000	1.854940000
F	5.233050000	-0.094174000	0.410187000
F	4.732638000	-2.086806000	-0.240940000
H	2.334561000	-2.542727000	-0.423594000
C	-0.200674000	-2.317489000	-0.676220000
F	-1.468237000	-2.300325000	-0.289087000
F	-0.181948000	-2.241402000	-2.017433000
F	0.269784000	-3.543774000	-0.391324000
C	-3.549631000	-0.495690000	0.346227000
H	-4.602197000	-0.174797000	0.389207000
H	-3.372966000	-0.942260000	-0.636960000
H	-3.336735000	-1.217750000	1.154722000
C	-1.172539000	-1.849704000	2.593739000
O	-2.374460000	-2.159731000	2.787077000
O	-0.219282000	-2.712477000	2.556594000
K	1.506841000	-1.234360000	3.544690000

37
IIB

C	-0.548165000	0.043497000	0.004330000
O	-1.605949000	-0.028440000	0.900285000
S	-1.670818000	0.197515000	-1.709661000
C	-3.947536000	1.331874000	-0.833610000
H	-3.882432000	1.342410000	0.268471000
H	-5.010354000	1.345804000	-1.123994000
N	-3.329897000	0.152948000	-1.409770000
O	-1.391086000	-1.009627000	-2.504256000
O	-1.407113000	1.503444000	-2.331861000
H	-3.471021000	2.233885000	-1.233199000
C	0.302775000	-1.154380000	-0.213583000
C	0.268871000	1.278391000	0.102018000
C	1.587273000	-1.042204000	-0.688948000
C	1.561688000	1.343175000	-0.360512000
C	2.235549000	0.200069000	-0.826142000
H	2.092621000	2.296171000	-0.317024000
C	-0.326417000	2.491581000	0.739558000
F	0.390858000	3.600026000	0.488559000
F	-1.580547000	2.760652000	0.357162000
F	-0.375003000	2.392410000	2.091685000
C	3.677221000	0.255545000	-1.155113000
F	4.472375000	0.052261000	-0.058206000
F	4.059729000	1.441165000	-1.649395000
F	4.054120000	-0.685107000	-2.034164000
H	2.144191000	-1.956385000	-0.914603000
C	-0.185403000	-2.550391000	0.014980000
F	-1.485671000	-2.645111000	0.301152000
F	0.036482000	-3.357693000	-1.038556000
F	0.469423000	-3.148108000	1.045378000
C	-3.889429000	-1.104813000	-0.949431000
H	-4.949335000	-1.146358000	-1.247699000
H	-3.367408000	-1.941778000	-1.426902000
H	-3.822432000	-1.212361000	0.147136000
C	-1.448006000	-0.402251000	2.269959000
O	-2.509911000	-0.423336000	2.871171000
O	-0.285830000	-0.649583000	2.641160000
K	2.134691000	-0.374328000	2.294034000

37
TSIIBC

C	-0.460814000	0.032727000	0.146345000
O	-1.552076000	-0.012476000	0.975275000
S	-1.667907000	0.143463000	-1.748714000
C	-3.971328000	1.306412000	-0.981356000
H	-3.893687000	1.342289000	0.121367000
H	-5.039616000	1.338626000	-1.252846000
N	-3.378908000	0.111462000	-1.548765000
O	-1.378589000	-1.098918000	-2.506310000
O	-1.402026000	1.419491000	-2.453818000
H	-3.484365000	2.193845000	-1.402926000
C	0.337507000	-1.159598000	-0.105943000
C	0.307048000	1.270026000	0.146325000
C	1.612833000	-1.063429000	-0.623830000

```
C        1.587747000      1.333180000     -0.359691000
C        2.251847000      0.174528000     -0.789986000
H        2.106806000      2.292662000     -0.384685000
C       -0.312893000      2.506846000      0.720863000
F        0.404146000      3.606027000      0.439585000
F       -1.556118000      2.744570000      0.293145000
F       -0.389642000      2.453568000      2.070772000
C        3.685720000      0.227593000     -1.180298000
F        4.510321000      0.059302000     -0.108489000
F        4.035193000      1.399937000     -1.723170000
F        4.026367000     -0.734268000     -2.047763000
H        2.156528000     -1.980867000     -0.864335000
C       -0.169795000     -2.548114000      0.147160000
F       -1.477514000     -2.625370000      0.390291000
F        0.087539000     -3.380956000     -0.873807000
F        0.447724000     -3.108625000      1.218739000
C       -3.922390000     -1.121337000     -1.011381000
H       -4.990005000     -1.186860000     -1.279389000
H       -3.405820000     -1.978483000     -1.459759000
H       -3.834895000     -1.183804000      0.089220000
C       -1.427773000     -0.349642000      2.367842000
O       -2.501511000     -0.352831000      2.942491000
O       -0.271224000     -0.583195000      2.763225000
K        2.157814000     -0.360510000      2.407285000


37
IIC
C       -0.126493000     -0.214838000      1.291897000
O       -0.905916000     -0.443077000      2.352506000
S       -1.476413000     -0.005986000     -2.020304000
C       -3.622204000      1.573416000     -2.265003000
H       -3.636409000      1.851547000     -1.185325000
H       -4.640548000      1.736601000     -2.657467000
N       -3.202545000      0.209170000     -2.502906000
O       -1.248985000     -1.437171000     -2.431706000
O       -0.799035000      0.970476000     -2.944003000
H       -2.938602000      2.261475000     -2.781720000
C        0.342717000     -1.277325000      0.502829000
C        0.235019000      1.098471000      0.939114000
C        1.188410000     -1.038842000     -0.574858000
C        1.082249000      1.334569000     -0.136778000
C        1.579067000      0.263317000     -0.874943000
H        1.352024000      2.355991000     -0.404361000
C       -0.351717000      2.255990000      1.707688000
F        0.087186000      3.430011000      1.242728000
F       -1.686053000      2.277208000      1.625009000
F       -0.039325000      2.217034000      3.010118000
C        2.554845000      0.502360000     -1.992355000
F        3.820829000      0.318554000     -1.558396000
F        2.493398000      1.743816000     -2.471681000
F        2.379726000     -0.338241000     -3.011791000
H        1.536024000     -1.870908000     -1.185827000
C       -0.140824000     -2.677681000      0.783515000
F       -1.469668000     -2.771629000      0.719860000
F        0.360509000     -3.558775000     -0.085925000
F        0.221538000     -3.099573000      2.006876000
C       -4.044336000     -0.746246000     -1.815271000
H       -5.076813000     -0.696664000     -2.201932000
H       -3.659084000     -1.761088000     -1.985015000
H       -4.097729000     -0.581228000     -0.713892000
C       -0.224742000     -0.660481000      3.658196000
O       -1.008984000     -0.858485000      4.554944000
O        1.009919000     -0.597979000      3.582862000
K        3.066103000     -0.310050000      2.217859000


43
A-fXs
C       -1.067796000      0.319142000     -0.289086000
O        1.570327000     -2.094248000     -0.373369000
S       -1.889402000     -0.217757000     -1.832366000
C       -3.707037000     -2.167616000     -1.907232000
H       -4.055956000     -2.936313000     -1.202359000
H       -3.712666000     -2.589896000     -2.927111000
N       -2.370265000     -1.752741000     -1.511578000
O       -0.849103000     -0.246512000     -2.850304000
O       -3.067818000      0.610736000     -2.039260000
H       -4.396087000     -1.317832000     -1.868804000
C        0.029527000      1.203916000     -0.367890000
C       -1.488496000     -0.120971000      0.983331000
C        0.754894000      1.513346000      0.784056000
C       -0.731232000      0.198331000      2.113213000
C        0.401140000      0.986541000      2.014429000
H       -1.041251000     -0.173195000      3.088798000
C       -2.763105000     -0.900510000      1.283209000
F       -3.788716000     -0.535169000      0.514426000
F       -2.611927000     -2.224116000      1.187334000
F       -3.156835000     -0.670480000      2.545561000
H        1.614449000      2.178711000      0.714553000
C        0.486268000      1.941995000     -1.622885000
F        1.314127000      1.236168000     -2.391287000
F       -0.537709000      2.360818000     -2.369475000
F        1.163000000      3.051726000     -1.282192000
C       -1.336733000     -2.779010000     -1.536966000
H       -1.134512000     -3.123688000     -2.566192000
H       -0.395115000     -2.425612000     -1.082427000
H       -1.697188000     -3.631891000     -0.944358000
C        2.003502000     -1.791657000      0.761898000
O        1.628745000     -2.225230000      1.867035000
C        3.127729000     -0.737227000      0.780684000
C        3.575178000     -0.155519000     -0.409521000
C        3.683084000     -0.307792000      1.989891000
C        4.543478000      0.848764000     -0.394429000
C        4.654413000      0.693815000      2.014064000
C        5.084013000      1.278804000      0.820019000
H        3.126286000     -0.498223000     -1.343352000
H        3.319880000     -0.768293000      2.911545000
H        4.875068000      1.303913000     -1.331769000
H        5.076810000      1.025264000      2.966709000
H        5.839978000      2.068329000      0.836270000
H        0.995548000      1.212257000      2.900186000


43
TSAC-fXs
C       -0.977997000      0.461886000      0.261743000
O        0.700685000     -0.295053000      0.039263000
S       -1.438607000     -0.018381000     -1.500645000
C       -2.774501000     -2.252646000     -2.087460000
H       -2.836077000     -3.282684000     -1.707657000
H       -2.824738000     -2.280043000     -3.191202000
N       -1.530008000     -1.665677000     -1.617562000
```

O	-0.424691000	0.457504000	-2.435942000
O	-2.803404000	0.494495000	-1.616975000
H	-3.631352000	-1.688924000	-1.706672000
C	-0.823383000	1.890509000	0.428175000
C	-1.657966000	-0.215649000	1.340601000
C	-1.251839000	2.526511000	1.590075000
C	-2.070588000	0.481016000	2.473457000
C	-1.897358000	1.850190000	2.614762000
H	-2.548282000	-0.074562000	3.280825000
C	-1.842654000	-1.711406000	1.414881000
F	-2.939388000	-2.158543000	0.769546000
F	-0.803300000	-2.418219000	0.961012000
F	-2.012824000	-2.117119000	2.690679000
H	-1.082905000	3.598801000	1.689153000
C	-0.108906000	2.771138000	-0.570257000
F	1.061335000	2.279040000	-0.991756000
F	-0.838811000	3.052988000	-1.664725000
F	0.186456000	3.973764000	-0.034239000
C	-0.319828000	-2.380152000	-1.988299000
H	-0.161563000	-2.383809000	-3.081372000
H	0.545001000	-1.929080000	-1.490270000
H	-0.414340000	-3.419897000	-1.642763000
C	1.598485000	-0.086389000	0.938153000
O	1.418474000	0.341467000	2.070303000
C	2.999176000	-0.430975000	0.466001000
C	3.280599000	-0.598876000	-0.894540000
C	4.030033000	-0.558042000	1.402288000
C	4.577609000	-0.895869000	-1.312190000
C	5.324771000	-0.866421000	0.987311000
C	5.600292000	-1.034856000	-0.371517000
H	2.469134000	-0.482838000	-1.615911000
H	3.798893000	-0.411791000	2.459770000
H	4.793008000	-1.019249000	-2.376513000
H	6.124193000	-0.973230000	1.724826000
H	6.615828000	-1.272434000	-0.698240000
H	-2.243243000	2.373777000	3.506548000

43
C-fXs

C	-0.410095000	0.582033000	0.938435000
O	0.823413000	0.211950000	0.481731000
S	-0.657991000	-0.076289000	-2.564049000
C	-2.794394000	-1.459095000	-1.666920000
H	-3.208256000	-2.088256000	-0.861175000
H	-3.157814000	-1.864095000	-2.636215000
N	-1.356279000	-1.401300000	-1.574243000
O	-0.991596000	-0.475863000	-3.978647000
O	-1.507335000	1.082975000	-2.112116000
H	-3.189004000	-0.437938000	-1.554248000
C	-0.771481000	1.920784000	0.771402000
C	-1.315253000	-0.334223000	1.473266000
C	-2.046339000	2.345272000	1.129007000
C	-2.593300000	0.107065000	1.827578000
C	-2.959980000	1.435792000	1.654675000
H	-3.309913000	-0.604988000	2.241555000
C	-0.990581000	-1.784216000	1.738717000
F	-1.996641000	-2.585712000	1.366500000
F	0.108983000	-2.229921000	1.132600000
F	-0.814435000	-1.992595000	3.054977000
H	-2.329454000	3.389345000	0.992549000
C	0.245877000	2.899999000	0.236531000
F	1.274858000	3.031874000	1.098194000
F	0.775861000	2.552578000	-0.931330000
F	-0.281702000	4.119218000	0.081375000
C	-0.715285000	-2.656306000	-1.878760000
H	-0.973424000	-3.049687000	-2.885930000
H	0.380684000	-2.538396000	-1.837570000
H	-0.992895000	-3.421976000	-1.135136000
C	1.839975000	-0.044204000	1.335628000
O	1.723828000	0.024619000	2.529344000
C	3.073438000	-0.403349000	0.590890000
C	3.079053000	-0.499568000	-0.807450000
C	4.241979000	-0.649918000	1.320500000
C	4.256915000	-0.842746000	-1.466780000
C	5.416175000	-0.989632000	0.654655000
C	5.423074000	-1.085974000	-0.738474000
H	2.160818000	-0.309750000	-1.369015000
H	4.219436000	-0.571380000	2.409308000
H	4.265185000	-0.920778000	-2.556118000
H	6.329357000	-1.180208000	1.222545000
H	6.344821000	-1.353456000	-1.260687000
H	-3.962754000	1.765455000	1.930492000

39
A-Ns

C	-0.952025000	1.275322000	0.534925000
O	2.377787000	1.082753000	-0.246102000
S	-1.314452000	3.023350000	0.444696000
C	-1.695524000	4.365269000	-1.817192000
H	-1.462474000	4.271583000	-2.887364000
H	-1.516164000	5.411376000	-1.509817000
N	-0.854863000	3.420455000	-1.094824000
O	-0.491414000	3.692255000	1.449495000
O	-2.764265000	3.177644000	0.518779000
H	-2.753769000	4.122540000	-1.668043000
C	-0.308769000	0.783746000	1.666922000
C	-1.408694000	0.434722000	-0.477468000
C	-0.125573000	-0.587616000	1.799237000
C	-1.217467000	-0.935353000	-0.355869000
C	-0.584346000	-1.418922000	0.783990000
H	-1.547026000	-1.621912000	-1.134252000
H	0.377901000	-1.009266000	2.668060000
C	0.582526000	3.550177000	-1.304981000
H	0.959107000	4.528494000	-0.955662000
H	1.137529000	2.731417000	-0.813957000
H	0.773949000	3.461288000	-2.383699000
C	2.062040000	0.161491000	-1.034184000
O	1.456091000	0.255823000	-2.119357000
C	2.465346000	-1.260029000	-0.595898000
C	3.154879000	-1.469550000	0.603594000
C	2.130805000	-2.367513000	-1.380753000
C	3.493877000	-2.757399000	1.016397000
C	2.466739000	-3.659501000	-0.974199000
C	3.147394000	-3.857644000	0.228301000
H	3.406186000	-0.595446000	1.208384000
H	1.589388000	-2.188879000	-2.312477000
H	4.025391000	-2.908130000	1.960059000
H	2.189150000	-4.517462000	-1.592374000
H	3.405501000	-4.868946000	0.553279000
H	0.052328000	1.468832000	2.434288000
H	-1.891825000	0.845069000	-1.365197000

```
N       -0.383812000     -2.871174000      0.916973000
O       -0.777727000     -3.582601000      0.017223000
O        0.150338000     -3.280110000      1.925427000


39
TSAB-Ns
C       -0.925630000      0.604531000      0.241682000
O        0.795926000      0.137842000     -0.048475000
S       -0.433353000      2.332278000      0.613643000
C       -0.490501000      3.966105000     -1.508197000
H       -0.215886000      3.898766000     -2.571526000
H       -0.243534000      4.980546000     -1.146353000
N        0.230313000      2.933507000     -0.780694000
O        0.560303000      2.340628000      1.683921000
O       -1.700471000      3.028330000      0.834556000
H       -1.571644000      3.815762000     -1.418257000
C       -1.385397000     -0.105041000      1.401754000
C       -1.678375000      0.448539000     -0.969882000
C       -2.338926000     -1.085266000      1.281601000
C       -2.631948000     -0.531466000     -1.073334000
C       -2.953953000     -1.329008000      0.040649000
H       -3.165801000     -0.687904000     -2.011410000
H       -2.647750000     -1.664802000      2.152399000
C        1.678485000      2.955322000     -0.903147000
H        2.122710000      3.848069000     -0.428148000
H        2.106576000      2.049739000     -0.461833000
H        1.934294000      2.965321000     -1.973057000
C        1.094730000     -1.104109000     -0.253223000
O        0.322032000     -2.008440000     -0.528520000
C        2.580180000     -1.380001000     -0.120857000
C        3.432432000     -0.477379000      0.525395000
C        3.102602000     -2.571310000     -0.634914000
C        4.792169000     -0.761119000      0.649963000
C        4.463608000     -2.850486000     -0.521469000
C        5.310405000     -1.945274000      0.122056000
H        3.012201000      0.442216000      0.937670000
H        2.424863000     -3.273679000     -1.125109000
H        5.451244000     -0.055541000      1.161828000
H        4.866806000     -3.778877000     -0.933484000
H        6.376904000     -2.165166000      0.215043000
H       -0.921062000      0.105941000      2.366905000
H       -1.432819000      1.078552000     -1.827152000
N       -3.944286000     -2.349554000     -0.075838000
O       -4.483119000     -2.529230000     -1.162053000
O       -4.224027000     -3.018506000      0.912366000


39
B-Ns
C        0.644352000      0.171889000     -0.186595000
O       -0.791257000      0.013999000     -0.004153000
S        0.620369000      2.018358000     -0.631282000
C        1.037855000      3.673302000      1.446301000
H        0.772945000      3.693431000      2.514243000
H        0.994850000      4.706461000      1.055784000
N        0.115955000      2.786015000      0.757709000
O       -0.359715000      2.238755000     -1.699208000
O        2.012900000      2.396772000     -0.866129000
H        2.064832000      3.304310000      1.350411000
C        1.153589000     -0.574314000     -1.360201000
C        1.434395000     -0.021027000      1.049304000
C        2.270883000     -1.345133000     -1.293352000
C        2.549853000     -0.795594000      1.075955000
C        3.010928000     -1.475584000     -0.086021000
H        3.111199000     -0.914577000      2.004025000
H        2.618478000     -1.880734000     -2.178109000
C       -1.296503000      3.099019000      0.888447000
H       -1.562517000      4.052269000      0.397425000
H       -1.909316000      2.296021000      0.464607000
H       -1.538557000      3.178385000      1.958773000
C       -1.343424000     -1.164184000      0.297024000
O       -0.739210000     -2.153883000      0.624194000
C       -2.834831000     -1.097589000      0.181940000
C       -3.470385000     -0.052801000     -0.500357000
C       -3.595563000     -2.126334000      0.748213000
C       -4.859772000     -0.036533000     -0.606059000
C       -4.984471000     -2.102490000      0.648011000
C       -5.616963000     -1.057539000     -0.028890000
H       -2.867359000      0.734345000     -0.956680000
H       -3.086856000     -2.940461000      1.268678000
H       -5.354035000      0.775924000     -1.143452000
H       -5.576960000     -2.902551000      1.097478000
H       -6.706499000     -1.041094000     -0.110204000
H        0.590476000     -0.477435000     -2.291439000
H        1.078860000      0.484677000      1.949844000
N        4.165294000     -2.263045000     -0.039328000
O        4.789779000     -2.361220000      1.023804000
O        4.543770000     -2.846961000     -1.061831000


39
TSBC-Ns
C       -0.520933000     -0.207510000      0.148057000
O        0.892246000     -0.281192000      0.127993000
S       -0.479111000      1.913516000      0.672982000
C       -1.077380000      3.990119000     -0.929859000
H       -1.193398000      4.251809000     -1.993390000
H       -0.537941000      4.817882000     -0.428168000
N       -0.380108000      2.722004000     -0.831068000
O        0.659595000      2.332070000      1.530586000
O       -1.825920000      2.240693000      1.186948000
H       -2.074163000      3.897297000     -0.481277000
C       -1.171458000     -0.844522000      1.268552000
C       -1.194934000     -0.289259000     -1.126358000
C       -2.460903000     -1.293192000      1.169526000
C       -2.484758000     -0.739133000     -1.207848000
C       -3.151445000     -1.222565000     -0.059787000
H       -3.003143000     -0.755504000     -2.167158000
H       -2.961631000     -1.730473000      2.034080000
C        0.948224000      2.710706000     -1.414179000
H        1.627072000      3.451960000     -0.950357000
H        1.401827000      1.715449000     -1.308276000
H        0.872772000      2.935189000     -2.489602000
C        1.536795000     -1.418879000     -0.170333000
O        1.001487000     -2.448505000     -0.490387000
C        3.013626000     -1.223659000     -0.056795000
C        3.555539000     -0.032722000      0.445195000
C        3.857631000     -2.265905000     -0.455499000
C        4.938184000      0.109552000      0.540762000
C        5.238981000     -2.116937000     -0.361151000
C        5.779057000     -0.929230000      0.136526000
H        2.885699000      0.768805000      0.764342000
```

H	3.419742000	-3.189145000	-0.840564000
H	5.362538000	1.035893000	0.934336000
H	5.897599000	-2.929487000	-0.675828000
H	6.862971000	-0.813161000	0.211607000
H	-0.625856000	-0.905654000	2.212889000
H	-0.667202000	0.060040000	-2.015464000
N	-4.489775000	-1.682908000	-0.153281000
O	-5.064354000	-1.625172000	-1.237803000
O	-5.039375000	-2.123143000	0.853563000

39
C-Ns

C	0.737890000	-1.210076000	-0.145265000
O	-0.593714000	-0.939493000	-0.313259000
S	0.316327000	2.300685000	-0.923199000
C	-0.153651000	4.400353000	0.708500000
H	0.030938000	4.848802000	1.700312000
H	-1.210021000	4.610687000	0.435029000
N	0.162873000	2.991788000	0.720946000
O	-1.074187000	2.453929000	-1.508952000
O	1.271192000	3.277739000	-1.549866000
H	0.496591000	4.896532000	-0.027583000
C	1.485333000	-1.475471000	-1.291252000
C	1.334531000	-1.150652000	1.115475000
C	2.853928000	-1.685036000	-1.182263000
C	2.701551000	-1.363186000	1.229086000
C	3.441678000	-1.627540000	0.078656000
H	3.195304000	-1.318740000	2.199295000
H	3.461358000	-1.891630000	-2.062692000
C	-0.748328000	2.214688000	1.526688000
H	-1.817192000	2.372326000	1.265027000
H	-0.533452000	1.141023000	1.403467000
H	-0.628112000	2.459432000	2.596697000
C	-1.539363000	-1.625340000	0.372647000
O	-1.288376000	-2.593498000	1.040298000
C	-2.892198000	-1.043502000	0.170174000
C	-3.081985000	0.178758000	-0.489520000
C	-3.989179000	-1.750843000	0.680199000
C	-4.375623000	0.680573000	-0.630591000
C	-5.275956000	-1.244172000	0.527428000
C	-5.469227000	-0.025967000	-0.128495000
H	-2.235848000	0.760132000	-0.877921000
H	-3.821507000	-2.699470000	1.194161000
H	-4.526676000	1.637214000	-1.136387000
H	-6.130736000	-1.798038000	0.921714000

```
32
NMs
C        0.917147000      0.246027000     -0.029237000
S       -0.609783000      1.227874000      0.240631000
C       -0.571694000      3.891432000      0.437692000
H        0.216557000      4.650888000      0.539166000
H       -1.450169000      4.201840000      1.028053000
N       -0.044206000      2.616149000      0.903105000
O       -1.351430000      0.487221000      1.250104000
O       -1.220041000      1.522813000     -1.043315000
H       -0.855866000      3.818855000     -0.617125000
C        0.862982000     -1.154456000      0.133153000
C        2.156921000      0.842012000     -0.326808000
C        2.040819000     -1.897175000      0.158415000
C        3.324167000      0.076963000     -0.289653000
C        3.271066000     -1.276831000     -0.006558000
H        4.283864000      0.551016000     -0.492043000
C        2.370091000      2.289101000     -0.762119000
F        3.448541000      2.367455000     -1.552989000
F        1.353444000      2.765363000     -1.475750000
F        2.600052000      3.116057000      0.259387000
C        4.531066000     -2.107969000      0.038299000
F        4.514298000     -2.961067000      1.066249000
F        5.623600000     -1.353390000      0.159050000
F        4.671161000     -2.838077000     -1.073739000
H        1.992897000     -2.976842000      0.302421000
C       -0.416457000     -1.986795000      0.212368000
F       -0.866892000     -2.143386000      1.453477000
F       -1.392485000     -1.488714000     -0.544428000
F       -0.178528000     -3.220866000     -0.257174000
C        0.361434000      2.545952000      2.299684000
H       -0.499955000      2.612923000      2.984469000
H        0.903496000      1.610998000      2.498652000
H        1.046259000      3.381305000      2.497960000
```

```
14
I
O       -0.447983000      0.101223000     -2.191679000
C       -1.618173000      0.496321000     -2.016370000
O       -2.067589000      1.654609000     -2.128501000
C       -2.643099000     -0.588919000     -1.607652000
C       -2.235358000     -1.908477000     -1.382717000
C       -3.997406000     -0.271623000     -1.455125000
C       -3.155749000     -2.889658000     -1.011338000
C       -4.925479000     -1.247673000     -1.089396000
C       -4.505677000     -2.561095000     -0.864914000
H       -1.175457000     -2.142578000     -1.506677000
H       -4.301696000      0.762549000     -1.632490000
H       -2.822053000     -3.916111000     -0.835249000
H       -5.981383000     -0.985929000     -0.977497000
H       -5.229682000     -3.327701000     -0.576207000
```

```
12
II-1
O        3.168183000      0.713402000     -0.036847000
C        2.831661000     -0.544149000      0.040098000
O        1.579765000     -0.843657000      0.111023000
O        3.731614000     -1.445548000      0.042582000
O       -3.167171000     -0.717976000     -0.001815000
C       -2.830673000      0.539531000     -0.076691000
O       -1.578863000      0.841143000     -0.140603000
O       -3.730756000      1.441180000     -0.084606000
K       -0.756372000     -1.681875000     -0.049524000
K        0.758157000      1.677996000      0.013949000
K       -5.650063000     -0.187536000      0.069183000
K        5.648636000      0.191484000     -0.120080000
```

```
7
II-2
O        1.410844000     -0.297617000     -1.549309000
C        1.826627000     -1.406753000     -1.040209000
O        1.494716000     -1.728319000      0.163455000
O        2.574092000     -2.194699000     -1.735054000
K        0.116502000      0.397414000      0.552536000
K        2.778613000     -3.948045000      0.122518000
K        2.574927000     -0.677000000     -3.801372000
```

```
46
IA
C       -0.425489000      1.068828000      0.592818000
O        1.554995000      0.214654000     -1.866633000
S        0.305071000      2.626160000      1.219830000
C        0.070523000      4.958803000     -0.050213000
H       -0.273300000      5.263644000     -1.049143000
H        0.906144000      5.611115000      0.256112000
N        0.482737000      3.564298000     -0.109433000
O        1.621538000      2.245062000      1.713248000
O       -0.649393000      3.256484000      2.117694000
H       -0.755202000      5.080493000      0.658110000
C       -0.102031000     -0.137291000      1.247851000
C       -1.273926000      1.020664000     -0.528583000
C       -0.494134000     -1.353958000      0.693211000
C       -1.652775000     -0.210235000     -1.062140000
C       -1.239884000     -1.391697000     -0.474106000
H       -2.285242000     -0.241827000     -1.947567000
C       -1.914037000      2.225220000     -1.209300000
F       -2.238794000      3.185688000     -0.343674000
F       -1.162888000      2.758855000     -2.173506000
F       -3.062449000      1.859902000     -1.798242000
C       -1.694559000     -2.720160000     -1.023383000
F       -0.745259000     -3.652562000     -0.939609000
F       -2.090987000     -2.643770000     -2.290818000
F       -2.743479000     -3.181572000     -0.316927000
H       -0.225875000     -2.283748000      1.194213000
C        0.580553000     -0.249208000      2.610284000
F        1.908784000     -0.272282000      2.554952000
F        0.206836000      0.724801000      3.441226000
F        0.216720000     -1.397431000      3.204030000
C        1.598887000      3.242764000     -0.990178000
H        2.555528000      3.620147000     -0.590626000
H        1.660792000      2.157504000     -1.167133000
H        1.405251000      3.724881000     -1.958412000
C        1.478527000     -1.017991000     -2.060044000
O        0.747207000     -1.615966000     -2.872835000
C        2.384011000     -1.897877000     -1.178701000
C        3.129155000     -1.335220000     -0.138100000
C        2.467575000     -3.277152000     -1.397425000
C        3.934771000     -2.132308000      0.675926000
```

```
C        3.277478000     -4.079557000     -0.593672000
C        4.012368000     -3.507940000      0.448222000
H        3.048010000     -0.259211000      0.027611000
H        1.875480000     -3.703087000     -2.209862000
H        4.501937000     -1.680997000      1.494386000
H        3.335658000     -5.156224000     -0.775795000
H        4.643687000     -4.135004000      1.083339000


37
IIA
C       -0.135722000      1.047848000      0.631187000
O        5.040880000      1.371392000      5.145430000
S        1.509550000      1.838513000      0.530360000
C        2.055068000      3.870558000     -1.106143000
H        1.540895000      4.342969000     -1.954444000
H        3.139223000      3.848987000     -1.303950000
N        1.519720000      2.522491000     -0.947957000
O        2.461663000      0.729493000      0.567465000
O        1.630289000      2.871239000      1.551705000
H        1.862988000      4.463015000     -0.205894000
C       -0.267875000     -0.175516000      1.322939000
C       -1.262405000      1.584162000     -0.018830000
C       -1.447999000     -0.906048000      1.213904000
C       -2.430606000      0.826875000     -0.120725000
C       -2.507121000     -0.429513000      0.454308000
H       -3.290280000      1.235069000     -0.651344000
C       -1.371976000      2.994496000     -0.593348000
F       -2.641267000      3.411855000     -0.522604000
F       -0.649893000      3.879160000      0.090148000
F       -1.027703000      3.064788000     -1.879697000
C       -3.766090000     -1.256613000      0.341974000
F       -3.482867000     -2.537534000      0.092362000
F       -4.563557000     -0.822319000     -0.633788000
F       -4.469970000     -1.223708000      1.478224000
H       -1.540142000     -1.859012000      1.735215000
C        0.757635000     -0.766938000      2.289838000
F        1.667835000     -1.535409000      1.706389000
F        1.390799000      0.190088000      2.987200000
F        0.139660000     -1.528551000      3.196503000
C        1.674996000      1.614179000     -2.078606000
H        2.730790000      1.349167000     -2.248590000
H        1.094720000      0.694603000     -1.919842000
H        1.277272000      2.114764000     -2.971099000
C        6.178858000      0.921385000      4.690732000
O        7.130655000      0.647432000      5.477481000
O        6.307428000      0.761204000      3.401334000
K        3.968632000      1.452750000      2.893268000


46
TSIAC
C        0.479304000      0.628099000     -0.154781000
O       -1.322929000     -0.044517000      0.060914000
S       -0.200990000      2.273147000     -0.733824000
C       -0.517137000      4.355573000      0.900821000
H       -0.781963000      4.516454000      1.955591000
H       -0.996889000      5.141832000      0.290909000
N       -0.968618000      3.027467000      0.513815000
O       -1.161750000      2.058011000     -1.806715000
O        1.022794000      3.023240000     -1.009133000
H        0.568712000      4.437523000      0.797175000
C        1.089941000     -0.144831000     -1.210806000
C        1.122144000      0.554544000      1.129721000
C        2.170577000     -0.970785000     -0.950641000
C        2.210450000     -0.283479000      1.331980000
C        2.758634000     -1.038892000      0.307404000
H        2.647560000     -0.346410000      2.326874000
C        0.603357000      1.221703000      2.384233000
F        0.953539000      2.516257000      2.489123000
F       -0.721513000      1.150274000      2.527885000
F        1.111183000      0.638006000      3.487455000
C        3.948680000     -1.915763000      0.521952000
F        3.714180000     -3.190014000      0.161604000
F        4.344219000     -1.940831000      1.800620000
F        5.012523000     -1.522362000     -0.203357000
H        2.581317000     -1.573556000     -1.760261000
C        0.542661000     -0.222296000     -2.622373000
F       -0.760637000     -0.506440000     -2.675231000
F        0.746023000      0.893997000     -3.338077000
F        1.150372000     -1.200898000     -3.321840000
C       -2.390974000      2.773066000      0.682266000
H       -3.002141000      3.411866000      0.020939000
H       -2.604839000      1.717486000      0.483566000
H       -2.656037000      2.990397000      1.726918000
C       -1.454784000     -1.295546000      0.309997000
O       -0.569331000     -2.077693000      0.639763000
C       -2.882550000     -1.795264000      0.159733000
C       -3.851014000     -1.030188000     -0.499613000
C       -3.229942000     -3.050544000      0.668986000
C       -5.151933000     -1.512463000     -0.642324000
C       -4.532431000     -3.530058000      0.536794000
C       -5.495657000     -2.761161000     -0.120251000
H       -3.565270000     -0.057729000     -0.905562000
H       -2.460987000     -3.642547000      1.170148000
H       -5.901990000     -0.912494000     -1.163754000
H       -4.798604000     -4.508526000      0.944559000
H       -6.516080000     -3.137483000     -0.227981000


46
IC
C       -0.214579000     -0.624263000     -0.177103000
O        1.120849000     -0.868239000     -0.090955000
S        1.273314000      2.123597000      1.629585000
C       -0.879646000      3.001673000      0.334964000
H       -1.428834000      3.090396000     -0.617780000
H       -0.985691000      3.963652000      0.880550000
N        0.493021000      2.628410000      0.096342000
O        2.688660000      1.877830000      1.155210000
O        1.209209000      3.359606000      2.487602000
H       -1.381671000      2.221376000      0.931698000
C       -1.010633000     -1.036552000      0.895844000
C       -0.782158000      0.067866000     -1.250255000
C       -2.369099000     -0.742047000      0.912076000
C       -2.146291000      0.343427000     -1.231004000
C       -2.933437000     -0.051551000     -0.154560000
H       -2.594598000      0.891499000     -2.061494000
C       -0.004042000      0.529056000     -2.462757000
F       -0.434460000      1.725165000     -2.878961000
F        1.308705000      0.625843000     -2.269096000
F       -0.183982000     -0.316895000     -3.488634000
```

C	-4.382139000	0.355562000	-0.121709000
F	-5.093853000	-0.371485000	0.742567000
F	-4.961240000	0.233202000	-1.320589000
F	-4.514822000	1.639789000	0.242394000
H	-2.982762000	-1.054976000	1.756482000
C	-0.383591000	-1.813681000	2.031167000
F	0.220562000	-2.925866000	1.585858000
F	0.534012000	-1.108953000	2.691179000
F	-1.300256000	-2.200298000	2.922277000
C	1.242808000	3.637545000	-0.612215000
H	1.184616000	4.641246000	-0.139079000
H	2.299260000	3.331936000	-0.637001000
H	0.878013000	3.731033000	-1.648721000
C	1.698674000	-1.816358000	-0.878410000
O	1.056831000	-2.510680000	-1.618085000
C	3.164807000	-1.858734000	-0.673016000
C	3.827883000	-0.850076000	0.039737000
C	3.877377000	-2.929057000	-1.228692000
C	5.211417000	-0.930974000	0.193039000
C	5.256679000	-3.001923000	-1.062041000
C	5.923439000	-2.001806000	-0.349913000
H	3.275377000	0.005187000	0.454573000
H	3.341049000	-3.700546000	-1.785123000
H	5.738294000	-0.146952000	0.742158000
H	5.814712000	-3.838430000	-1.488262000
H	7.007149000	-2.057543000	-0.220553000

37
TSIIAB

C	0.202061000	-0.038809000	0.541219000
O	-0.829068000	-0.573333000	2.447826000
S	-1.279324000	0.813139000	-0.235437000
C	-2.992941000	1.465811000	1.713116000
H	-2.607998000	0.923020000	2.589057000
H	-4.082218000	1.599538000	1.786470000
N	-2.731927000	0.703309000	0.501404000
O	-1.406553000	0.153702000	-1.530240000
O	-0.894180000	2.221206000	-0.221188000
H	-2.531171000	2.455573000	1.641761000
C	0.664952000	-1.251407000	-0.032608000
C	1.190351000	0.814839000	1.114576000
C	2.016948000	-1.586503000	-0.007650000
C	2.535910000	0.474484000	1.087797000
C	2.963821000	-0.724857000	0.522545000
H	3.267730000	1.142138000	1.547628000
C	0.917052000	2.048966000	1.956651000
F	1.320324000	3.178082000	1.365865000
F	-0.327592000	2.215584000	2.361058000
F	1.651257000	1.955672000	3.101212000
C	4.405164000	-1.122590000	0.620802000
F	4.691994000	-1.592934000	1.854940000
F	5.233050000	-0.094174000	0.410187000
F	4.732638000	-2.086806000	-0.240940000
H	2.334561000	-2.542727000	-0.423594000
C	-0.200674000	-2.317489000	-0.676220000
F	-1.468237000	-2.300325000	-0.289087000
F	-0.181948000	-2.241402000	-2.017433000
F	0.269784000	-3.543774000	-0.391324000
C	-3.549631000	-0.495690000	0.346227000
H	-4.602197000	-0.174797000	0.389207000
H	-3.372966000	-0.942260000	-0.636960000
H	-3.336735000	-1.217750000	1.154722000
C	-1.172539000	-1.849704000	2.593739000
O	-2.374460000	-2.159731000	2.787077000
O	-0.219282000	-2.712477000	2.556594000
K	1.506841000	-1.234360000	3.544690000

37
IIB

C	-0.548165000	0.043497000	0.004330000
O	-1.605949000	-0.028440000	0.900285000
S	-1.670818000	0.197515000	-1.709661000
C	-3.947536000	1.331874000	-0.833610000
H	-3.882432000	1.342410000	0.268471000
H	-5.010354000	1.345804000	-1.123994000
N	-3.329897000	0.152948000	-1.409770000
O	-1.391086000	-1.009627000	-2.504256000
O	-1.407113000	1.503444000	-2.331861000
H	-3.471021000	2.233885000	-1.233199000
C	0.302775000	-1.154380000	-0.213583000
C	0.268871000	1.278391000	0.102018000
C	1.587273000	-1.042204000	-0.688948000
C	1.561688000	1.343175000	-0.360512000
C	2.235549000	0.200069000	-0.826142000
H	2.092621000	2.296171000	-0.317024000
C	-0.326417000	2.491581000	0.739558000
F	0.390858000	3.600026000	0.488559000
F	-1.580547000	2.760652000	0.357162000
F	-0.375003000	2.392410000	2.091685000
C	3.677221000	0.255545000	-1.155113000
F	4.472375000	0.052261000	-0.058206000
F	4.059729000	1.441165000	-1.649395000
F	4.054120000	-0.685107000	-2.034164000
H	2.144191000	-1.956385000	-0.914603000
C	-0.185403000	-2.550391000	0.014980000
F	-1.485671000	-2.645111000	0.301152000
F	0.036482000	-3.357693000	-1.038556000
F	0.469423000	-3.148108000	1.045378000
C	-3.889429000	-1.104813000	-0.949431000
H	-4.949335000	-1.146358000	-1.247699000
H	-3.367408000	-1.941778000	-1.426902000
H	-3.822432000	-1.212361000	0.147136000
C	-1.448006000	-0.402251000	2.269959000
O	-2.509911000	-0.423336000	2.871171000
O	-0.285830000	-0.649583000	2.641160000
K	2.134691000	-0.374328000	2.294034000

37
TSIIBC

C	-0.460814000	0.032727000	0.146345000
O	-1.552076000	-0.012476000	0.975275000
S	-1.667907000	0.143463000	-1.748714000
C	-3.971328000	1.306412000	-0.981356000
H	-3.893687000	1.342289000	0.121367000
H	-5.039616000	1.338626000	-1.252846000
N	-3.378908000	0.111462000	-1.548765000
O	-1.378589000	-1.098918000	-2.506310000
O	-1.402026000	1.419491000	-2.453818000
H	-3.484365000	2.193845000	-1.402926000

C	0.337507000	-1.159598000	-0.105943000
C	0.307048000	1.270026000	0.146325000
C	1.612833000	-1.063429000	-0.623830000
C	1.587747000	1.333180000	-0.359691000
C	2.251847000	0.174528000	-0.789986000
H	2.106806000	2.292662000	-0.384685000
C	-0.312893000	2.506846000	0.720863000
F	0.404146000	3.606027000	0.439585000
F	-1.556118000	2.744570000	0.293145000
F	-0.389642000	2.453568000	2.070772000
C	3.685720000	0.227593000	-1.180298000
F	4.510321000	0.059302000	-0.108489000
F	4.035193000	1.399937000	-1.723170000
F	4.026367000	-0.734268000	-2.047763000
H	2.156528000	-1.980867000	-0.864335000
C	-0.169795000	-2.548114000	0.147160000
F	-1.477514000	-2.625370000	0.390291000
F	0.087539000	-3.380956000	-0.873807000
F	0.447724000	-3.108625000	1.218739000
C	-3.922390000	-1.121337000	-1.011381000
H	-4.990005000	-1.186860000	-1.279389000
H	-3.405820000	-1.978483000	-1.459759000
H	-3.834895000	-1.183804000	0.089220000
C	-1.427773000	-0.349642000	2.367842000
O	-2.501511000	-0.352831000	2.942491000
O	-0.271224000	-0.583195000	2.763225000
K	2.157814000	-0.360510000	2.407285000

37
IIC

C	-0.126493000	-0.214838000	1.291897000
O	-0.905916000	-0.443077000	2.352506000
S	-1.476413000	-0.005986000	-2.020304000
C	-3.622204000	1.573416000	-2.265003000
H	-3.636409000	1.851547000	-1.185325000
H	-4.640548000	1.736601000	-2.657467000
N	-3.202545000	0.209170000	-2.502906000
O	-1.248985000	-1.437171000	-2.431706000
O	-0.799035000	0.970476000	-2.944003000
H	-2.938602000	2.261475000	-2.781720000
C	0.342717000	-1.277325000	0.502829000
C	0.235019000	1.098471000	0.939114000
C	1.188410000	-1.038842000	-0.574858000
C	1.082249000	1.334569000	-0.136778000
C	1.579067000	0.263317000	-0.874943000
H	1.352024000	2.355991000	-0.404361000
C	-0.351717000	2.255990000	1.707688000
F	0.087186000	3.430011000	1.242728000
F	-1.686053000	2.277208000	1.625009000
F	-0.039325000	2.217034000	3.010118000
C	2.554845000	0.502360000	-1.992355000
F	3.820829000	0.318554000	-1.558396000
F	2.493398000	1.743816000	-2.471681000
F	2.379726000	-0.338241000	-3.011791000
H	1.536024000	-1.870908000	-1.185827000
C	-0.140824000	-2.677681000	0.783515000
F	-1.469668000	-2.771629000	0.719860000
F	0.360509000	-3.558775000	-0.085925000
F	0.221538000	-3.099573000	2.006876000
C	-4.044336000	-0.746246000	-1.815271000
H	-5.076813000	-0.696664000	-2.201932000
H	-3.659084000	-1.761088000	-1.985015000
H	-4.097729000	-0.581228000	-0.713892000
C	-0.224742000	-0.660481000	3.658196000
O	-1.008984000	-0.858485000	4.554944000
O	1.009919000	-0.597979000	3.582862000
K	3.066103000	-0.310050000	2.217859000

43
A-fXs

C	-1.067796000	0.319142000	-0.289086000
O	1.570327000	-2.094248000	-0.373369000
S	-1.889402000	-0.217757000	-1.832366000
C	-3.707037000	-2.167616000	-1.907232000
H	-4.055956000	-2.936313000	-1.202359000
H	-3.712666000	-2.589896000	-2.927111000
N	-2.370265000	-1.752741000	-1.511578000
O	-0.849103000	-0.246512000	-2.850304000
O	-3.067818000	0.610736000	-2.039260000
H	-4.396087000	-1.317832000	-1.868804000
C	0.029527000	1.203916000	-0.367890000
C	-1.488496000	-0.120971000	0.983331000
C	0.754894000	1.513346000	0.784056000
C	-0.731232000	0.198331000	2.113213000
C	0.401140000	0.986541000	2.014429000
H	-1.041251000	-0.173195000	3.088798000
C	-2.763105000	-0.900510000	1.283209000
F	-3.788716000	-0.535169000	0.514426000
F	-2.611927000	-2.224116000	1.187334000
F	-3.156835000	-0.670480000	2.545561000
H	1.614449000	2.178711000	0.714553000
C	0.486268000	1.941995000	-1.622885000
F	1.314127000	1.236168000	-2.391287000
F	-0.537709000	2.360818000	-2.369475000
F	1.163000000	3.051726000	-1.282192000
C	-1.336733000	-2.779010000	-1.536966000
H	-1.134512000	-3.123688000	-2.566192000
H	-0.395115000	-2.425612000	-1.082427000
H	-1.697188000	-3.631891000	-0.944358000
C	2.003502000	-1.791657000	0.761898000
O	1.628745000	-2.225230000	1.867035000
C	3.127729000	-0.737227000	0.780684000
C	3.575178000	-0.155519000	-0.409521000
C	3.683084000	-0.307792000	1.989891000
C	4.543478000	0.848764000	-0.394429000
C	4.654413000	0.693815000	2.014064000
C	5.084013000	1.278804000	0.820019000
H	3.126286000	-0.498223000	-1.343352000
H	3.319880000	-0.768293000	2.911545000
H	4.875068000	1.303913000	-1.331769000
H	5.076810000	1.025264000	2.966709000
H	5.839978000	2.068329000	0.836270000
H	0.995548000	1.212257000	2.900186000

43
TSAC-fXs

C	-0.977997000	0.461886000	0.261743000
O	0.700685000	-0.295053000	0.039263000
S	-1.438607000	-0.018381000	-1.500645000

```
C       -2.774501000     -2.252646000     -2.087460000
H       -2.836077000     -3.282684000     -1.707657000
H       -2.824738000     -2.280043000     -3.191202000
N       -1.530008000     -1.665677000     -1.617562000
O       -0.424691000      0.457504000     -2.435942000
O       -2.803404000      0.494495000     -1.616975000
H       -3.631352000     -1.688924000     -1.706672000
C       -0.823383000      1.890509000      0.428175000
C       -1.657966000     -0.215649000      1.340601000
C       -1.251839000      2.526511000      1.590075000
C       -2.070588000      0.481016000      2.473457000
C       -1.897358000      1.850190000      2.614762000
H       -2.548282000     -0.074562000      3.280825000
C       -1.842654000     -1.711406000      1.414881000
F       -2.939388000     -2.158543000      0.769546000
F       -0.803300000     -2.418219000      0.961012000
F       -2.012824000     -2.117119000      2.690679000
H       -1.082905000      3.598801000      1.689153000
C       -0.108906000      2.771138000     -0.570257000
F        1.061335000      2.279040000     -0.991756000
F       -0.838811000      3.052988000     -1.664725000
F        0.186456000      3.973764000     -0.034239000
C       -0.319828000     -2.380152000     -1.988299000
H       -0.161563000     -2.383809000     -3.081372000
H        0.545001000     -1.929080000     -1.490270000
H       -0.414340000     -3.419897000     -1.642763000
C        1.598485000     -0.086389000      0.938153000
O        1.418474000      0.341467000      2.070303000
C        2.999176000     -0.430975000      0.466001000
C        3.280599000     -0.598876000     -0.894540000
C        4.030033000     -0.558042000      1.402288000
C        4.577609000     -0.895869000     -1.312190000
C        5.324771000     -0.866421000      0.987311000
C        5.600292000     -1.034856000     -0.371517000
H        2.469134000     -0.482838000     -1.615911000
H        3.798893000     -0.411791000      2.459770000
H        4.793008000     -1.019249000     -2.376513000
H        6.124193000     -0.973230000      1.724826000
H        6.615828000     -1.272434000     -0.698240000
H       -2.243243000      2.373777000      3.506548000


43
C-fXs
C       -0.410095000      0.582033000      0.938435000
O        0.823413000      0.211950000      0.481731000
S       -0.657991000     -0.076289000     -2.564049000
C       -2.794394000     -1.459095000     -1.666920000
H       -3.208256000     -2.088256000     -0.861175000
H       -3.157814000     -1.864095000     -2.636215000
N       -1.356279000     -1.401300000     -1.574243000
O       -0.991596000     -0.475863000     -3.978647000
O       -1.507335000      1.082975000     -2.112116000
H       -3.189004000     -0.437938000     -1.554248000
C       -0.771481000      1.920784000      0.771402000
C       -1.315253000     -0.334223000      1.473266000
C       -2.046339000      2.345272000      1.129007000
C       -2.593300000      0.107065000      1.827578000
C       -2.959980000      1.435792000      1.654675000
H       -3.309913000     -0.604988000      2.241555000
C       -0.990581000     -1.784216000      1.738717000
F       -1.996641000     -2.585712000      1.366500000
F        0.108983000     -2.229921000      1.132600000
F       -0.814435000     -1.992595000      3.054977000
H       -2.329454000      3.389345000      0.992549000
C        0.245877000      2.899999000      0.236531000
F        1.274858000      3.031874000      1.098194000
F        0.775861000      2.552578000     -0.931330000
F       -0.281702000      4.119218000      0.081375000
C       -0.715285000     -2.656306000     -1.878760000
H       -0.973424000     -3.049687000     -2.885930000
H        0.380684000     -2.538396000     -1.837570000
H       -0.992895000     -3.421976000     -1.135136000
C        1.839975000     -0.044204000      1.335628000
O        1.723828000      0.024619000      2.529344000
C        3.073438000     -0.403349000      0.590890000
C        3.079053000     -0.499568000     -0.807450000
C        4.241979000     -0.649918000      1.320500000
C        4.256915000     -0.842746000     -1.466780000
C        5.416175000     -0.989632000      0.654655000
C        5.423074000     -1.085974000     -0.738474000
H        2.160818000     -0.309750000     -1.369015000
H        4.219436000     -0.571380000      2.409308000
H        4.265185000     -0.920778000     -2.556118000
H        6.329357000     -1.180208000      1.222545000
H        6.344821000     -1.353456000     -1.260687000
H       -3.962754000      1.765455000      1.930492000


39
A-Ns
C       -0.952025000      1.275322000      0.534925000
O        2.377787000      1.082753000     -0.246102000
S       -1.314452000      3.023350000      0.444696000
C       -1.695524000      4.365269000     -1.817192000
H       -1.462474000      4.271583000     -2.887364000
H       -1.516164000      5.411376000     -1.509817000
N       -0.854863000      3.420455000     -1.094824000
O       -0.491414000      3.692255000      1.449495000
O       -2.764265000      3.177644000      0.518779000
H       -2.753769000      4.122540000     -1.668043000
C       -0.308769000      0.783746000      1.666922000
C       -1.408694000      0.434722000     -0.477468000
C       -0.125573000     -0.587616000      1.799237000
C       -1.217467000     -0.935353000     -0.355869000
C       -0.584346000     -1.418922000      0.783990000
H       -1.547026000     -1.621912000     -1.134252000
H        0.377901000     -1.009266000      2.668060000
C        0.582526000      3.550177000     -1.304981000
H        0.959107000      4.528494000     -0.955662000
H        1.137529000      2.731417000     -0.813957000
H        0.773949000      3.461288000     -2.383699000
C        2.062040000      0.161491000     -1.034184000
O        1.456091000      0.255823000     -2.119357000
C        2.465346000     -1.260029000     -0.595898000
C        3.154879000     -1.469550000      0.603594000
C        2.130805000     -2.367513000     -1.380753000
C        3.493877000     -2.757399000      1.016397000
C        2.466739000     -3.659501000     -0.974199000
C        3.147394000     -3.857644000      0.228301000
H        3.406186000     -0.595446000      1.208384000
H        1.589388000     -2.188879000     -2.312477000
```

```
H        4.025391000     -2.908130000      1.960059000
H        2.189150000     -4.517462000     -1.592374000
H        3.405501000     -4.868946000      0.553279000
H        0.052328000      1.468832000      2.434288000
H       -1.891825000      0.845069000     -1.365197000
N       -0.383812000     -2.871174000      0.916973000
O       -0.777727000     -3.582601000      0.017223000
O        0.150338000     -3.280110000      1.925427000


39
TSAB-Ns
C       -0.925630000      0.604531000      0.241682000
O        0.795926000      0.137842000     -0.048475000
S       -0.433353000      2.332278000      0.613643000
C       -0.490501000      3.966105000     -1.508197000
H       -0.215886000      3.898766000     -2.571526000
H       -0.243534000      4.980546000     -1.146353000
N        0.230313000      2.933507000     -0.780694000
O        0.560303000      2.340628000      1.683921000
O       -1.700471000      3.028330000      0.834556000
H       -1.571644000      3.815762000     -1.418257000
C       -1.385397000     -0.105041000      1.401754000
C       -1.678375000      0.448539000     -0.969882000
C       -2.338926000     -1.085266000      1.281601000
C       -2.631948000     -0.531466000     -1.073334000
C       -2.953953000     -1.329008000      0.040649000
H       -3.165801000     -0.687904000     -2.011410000
H       -2.647750000     -1.664802000      2.152399000
C        1.678485000      2.955322000     -0.903147000
H        2.122710000      3.848069000     -0.428148000
H        2.106576000      2.049739000     -0.461833000
H        1.934294000      2.965321000     -1.973057000
C        1.094730000     -1.104109000     -0.253223000
O        0.322032000     -2.008440000     -0.528520000
C        2.580180000     -1.380001000     -0.120857000
C        3.432432000     -0.477379000      0.525395000
C        3.102602000     -2.571310000     -0.634914000
C        4.792169000     -0.761119000      0.649963000
C        4.463608000     -2.850486000     -0.521469000
C        5.310405000     -1.945274000      0.122056000
H        3.012201000      0.442216000      0.937670000
H        2.424863000     -3.273679000     -1.125109000
H        5.451244000     -0.055541000      1.161828000
H        4.866806000     -3.778877000     -0.933484000
H        6.376904000     -2.165166000      0.215043000
H       -0.921062000      0.105941000      2.366905000
H       -1.432819000      1.078552000     -1.827152000
N       -3.944286000     -2.349554000     -0.075838000
O       -4.483119000     -2.529230000     -1.162053000
O       -4.224027000     -3.018506000      0.912366000


39
B-Ns
C        0.644352000      0.171889000     -0.186595000
O       -0.791257000      0.013999000     -0.004153000
S        0.620369000      2.018358000     -0.631282000
C        1.037855000      3.673302000      1.446301000
H        0.772945000      3.693431000      2.514243000
H        0.994850000      4.706461000      1.055784000
N        0.115955000      2.786015000      0.757709000
O       -0.359715000      2.238755000     -1.699208000
O        2.012900000      2.396772000     -0.866129000
H        2.064832000      3.304310000      1.350411000
C        1.153589000     -0.574314000     -1.360201000
C        1.434395000     -0.021027000      1.049304000
C        2.270883000     -1.345133000     -1.293352000
C        2.549853000     -0.795594000      1.075955000
C        3.010928000     -1.475584000     -0.086021000
H        3.111199000     -0.914577000      2.004025000
H        2.618478000     -1.880734000     -2.178109000
C       -1.296503000      3.099019000      0.888447000
H       -1.562517000      4.052269000      0.397425000
H       -1.909316000      2.296021000      0.464607000
H       -1.538557000      3.178385000      1.958773000
C       -1.343424000     -1.164184000      0.297024000
O       -0.739210000     -2.153883000      0.624194000
C       -2.834831000     -1.097589000      0.181940000
C       -3.470385000     -0.052801000     -0.500357000
C       -3.595563000     -2.126334000      0.748213000
C       -4.859772000     -0.036533000     -0.606059000
C       -4.984471000     -2.102490000      0.648011000
C       -5.616963000     -1.057539000     -0.028890000
H       -2.867359000      0.734345000     -0.956680000
H       -3.086856000     -2.940461000      1.268678000
H       -5.354035000      0.775924000     -1.143452000
H       -5.576960000     -2.902551000      1.097478000
H       -6.706499000     -1.041094000     -0.110204000
H        0.590476000     -0.477435000     -2.291439000
H        1.078860000      0.484677000      1.949844000
N        4.165294000     -2.263045000     -0.039328000
O        4.789779000     -2.361220000      1.023804000
O        4.543770000     -2.846961000     -1.061831000


39
TSBC-Ns
C       -0.520933000     -0.207510000      0.148057000
O        0.892246000     -0.281192000      0.127993000
S       -0.479111000      1.913516000      0.672982000
C       -1.077380000      3.990119000     -0.929859000
H       -1.193398000      4.251809000     -1.993390000
H       -0.537941000      4.817882000     -0.428168000
N       -0.380108000      2.722004000     -0.831068000
O        0.659595000      2.332070000      1.530586000
O       -1.825920000      2.240693000      1.186948000
H       -2.074163000      3.897297000     -0.481277000
C       -1.171458000     -0.844522000      1.268552000
C       -1.194934000     -0.289259000     -1.126358000
C       -2.460903000     -1.293192000      1.169526000
C       -2.484758000     -0.739133000     -1.207848000
C       -3.151445000     -1.222565000     -0.059787000
H       -3.003143000     -0.755504000     -2.167158000
H       -2.961631000     -1.730473000      2.034080000
C        0.948224000      2.710706000     -1.414179000
H        1.627072000      3.451960000     -0.950357000
H        1.401827000      1.715449000     -1.308276000
H        0.872772000      2.935189000     -2.489602000
C        1.536795000     -1.418879000     -0.170333000
O        1.001487000     -2.448505000     -0.490387000
C        3.013626000     -1.223659000     -0.056795000
```

```
C        3.555539000     -0.032722000      0.445195000
C        3.857631000     -2.265905000     -0.455499000
C        4.938184000      0.109552000      0.540762000
C        5.238981000     -2.116937000     -0.361151000
C        5.779057000     -0.929230000      0.136526000
H        2.885699000      0.768805000      0.764342000
H        3.419742000     -3.189145000     -0.840564000
H        5.362538000      1.035893000      0.934336000
H        5.897599000     -2.929487000     -0.675828000
H        6.862971000     -0.813161000      0.211607000
H       -0.625856000     -0.905654000      2.212889000
H       -0.667202000      0.060040000     -2.015464000
N       -4.489775000     -1.682908000     -0.153281000
O       -5.064354000     -1.625172000     -1.237803000
O       -5.039375000     -2.123143000      0.853563000


39
C-Ns
C        0.737890000     -1.210076000     -0.145265000
O       -0.593714000     -0.939493000     -0.313259000
S        0.316327000      2.300685000     -0.923199000
C       -0.153651000      4.400353000      0.708500000
H        0.030938000      4.848802000      1.700312000
H       -1.210021000      4.610687000      0.435029000
N        0.162873000      2.991788000      0.720946000
O       -1.074187000      2.453929000     -1.508952000
O        1.271192000      3.277739000     -1.549866000
H        0.496591000      4.896532000     -0.027583000
C        1.485333000     -1.475471000     -1.291252000
C        1.334531000     -1.150652000      1.115475000
C        2.853928000     -1.685036000     -1.182263000
C        2.701551000     -1.363186000      1.229086000
C        3.441678000     -1.627540000      0.078656000
H        3.195304000     -1.318740000      2.199295000
H        3.461358000     -1.891630000     -2.062692000
C       -0.748328000      2.214688000      1.526688000
H       -1.817192000      2.372326000      1.265027000
H       -0.533452000      1.141023000      1.403467000
H       -0.628112000      2.459432000      2.596697000
C       -1.539363000     -1.625340000      0.372647000
O       -1.288376000     -2.593498000      1.040298000
C       -2.892198000     -1.043502000      0.170174000
C       -3.081985000      0.178758000     -0.489520000
C       -3.989179000     -1.750843000      0.680199000
C       -4.375623000      0.680573000     -0.630591000
C       -5.275956000     -1.244172000      0.527428000
C       -5.469227000     -0.025967000     -0.128495000
H       -2.235848000      0.760132000     -0.877921000
H       -3.821507000     -2.699470000      1.194161000
H       -4.526676000      1.637214000     -1.136387000
H       -6.130736000     -1.798038000      0.921714000
```

7. NMR spectra

1,3-Bis((1*R*,2*S*,5*R*)-2-isopropyl-5-methylcyclohexyl)urea (S2)

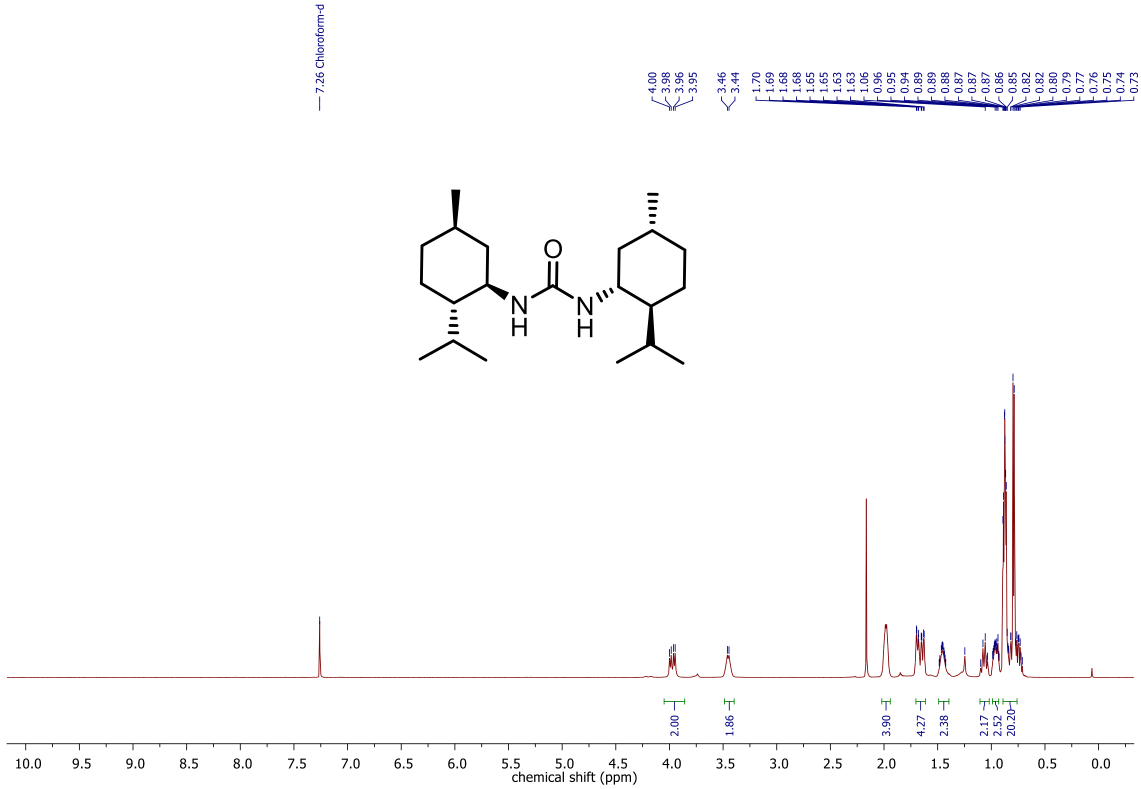

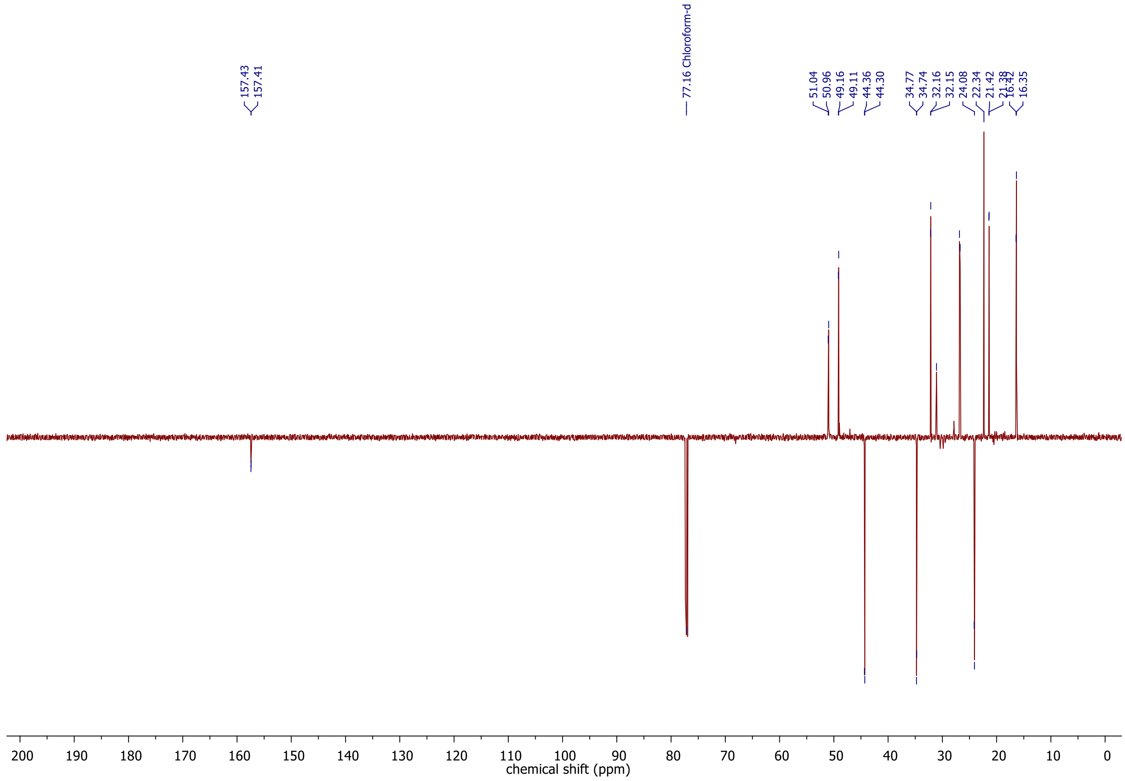

***N*-Phenethyl-2,4,6-tris(trifluoromethyl)benzenesulfonamide (S4)**

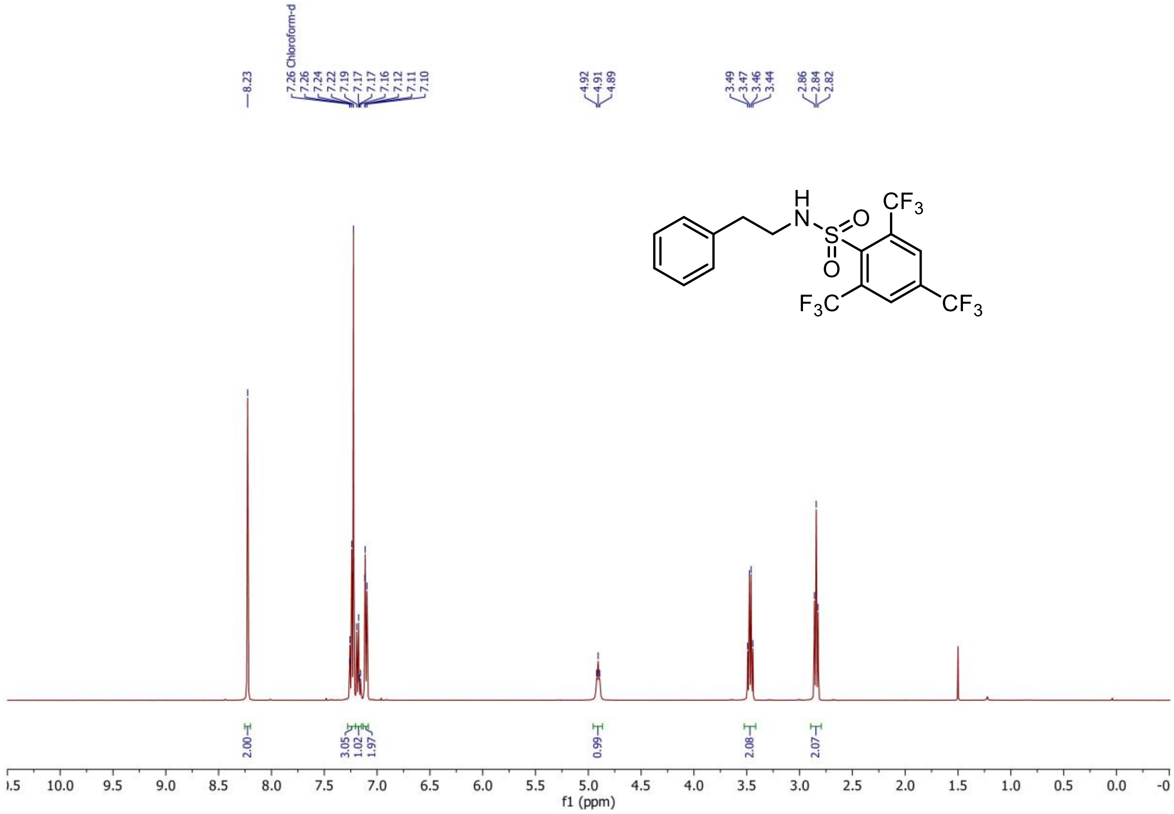

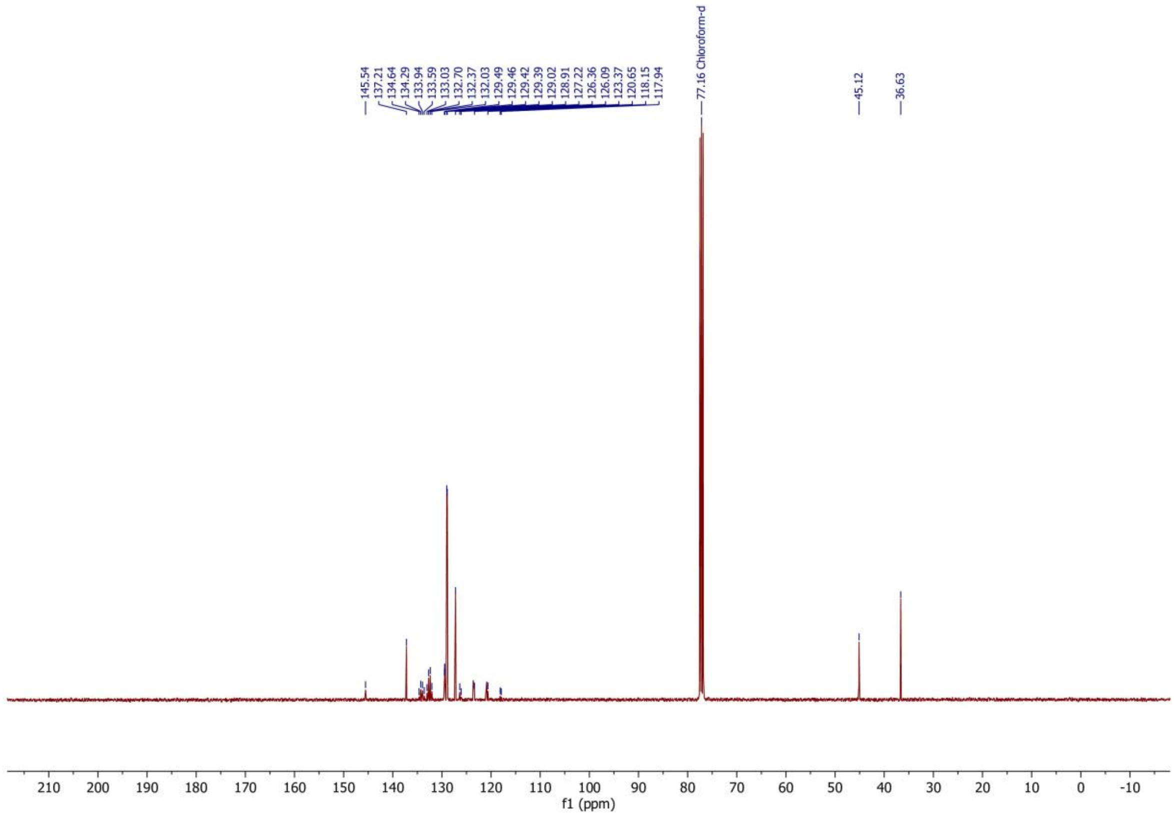

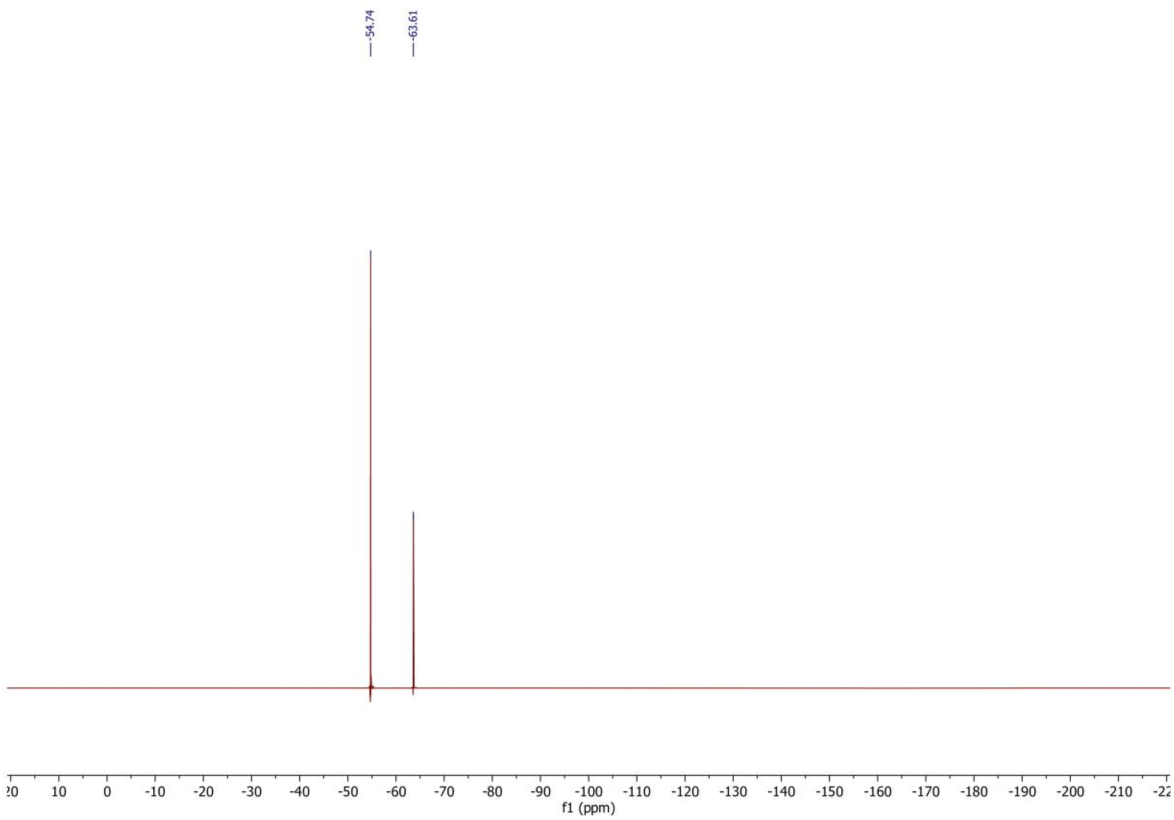

-54.74
-63.61
20 10 0 -10 -20 -30 -40 -50 -60 -70 -80 -90 -100 -110 -120 -130 -140 -150 -160 -170 -180 -190 -200 -210 -22
f1 (ppm)

***N*-(3,4-Dimethoxyphenethyl)-*N*-methyl-2,6-bis(trifluoromethyl)benzenesulfonamide (S5)**

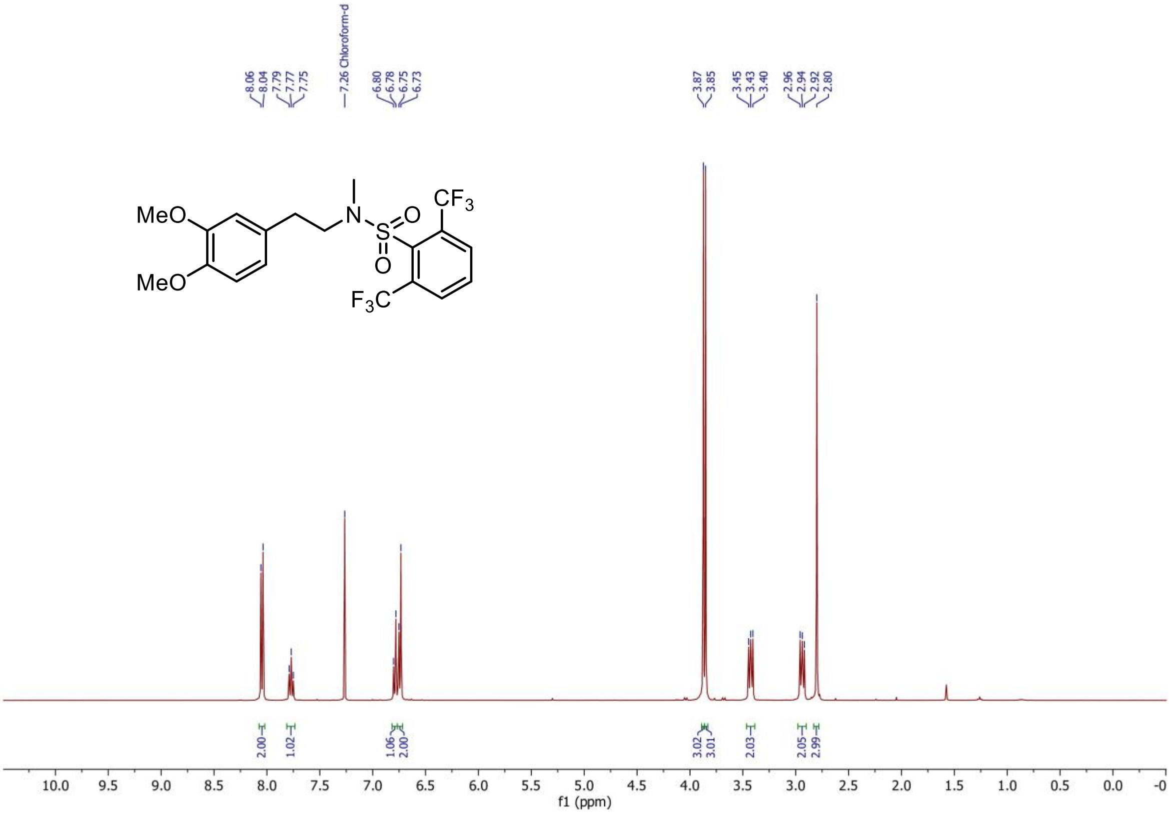

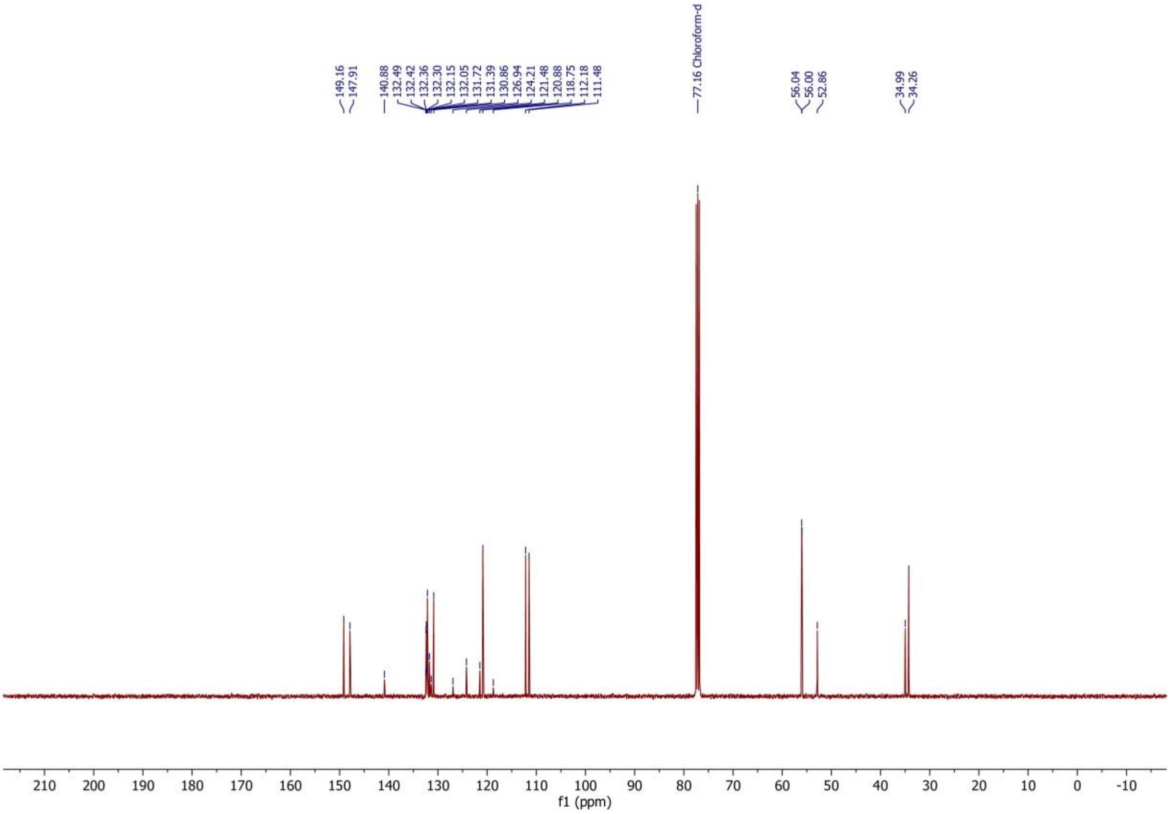

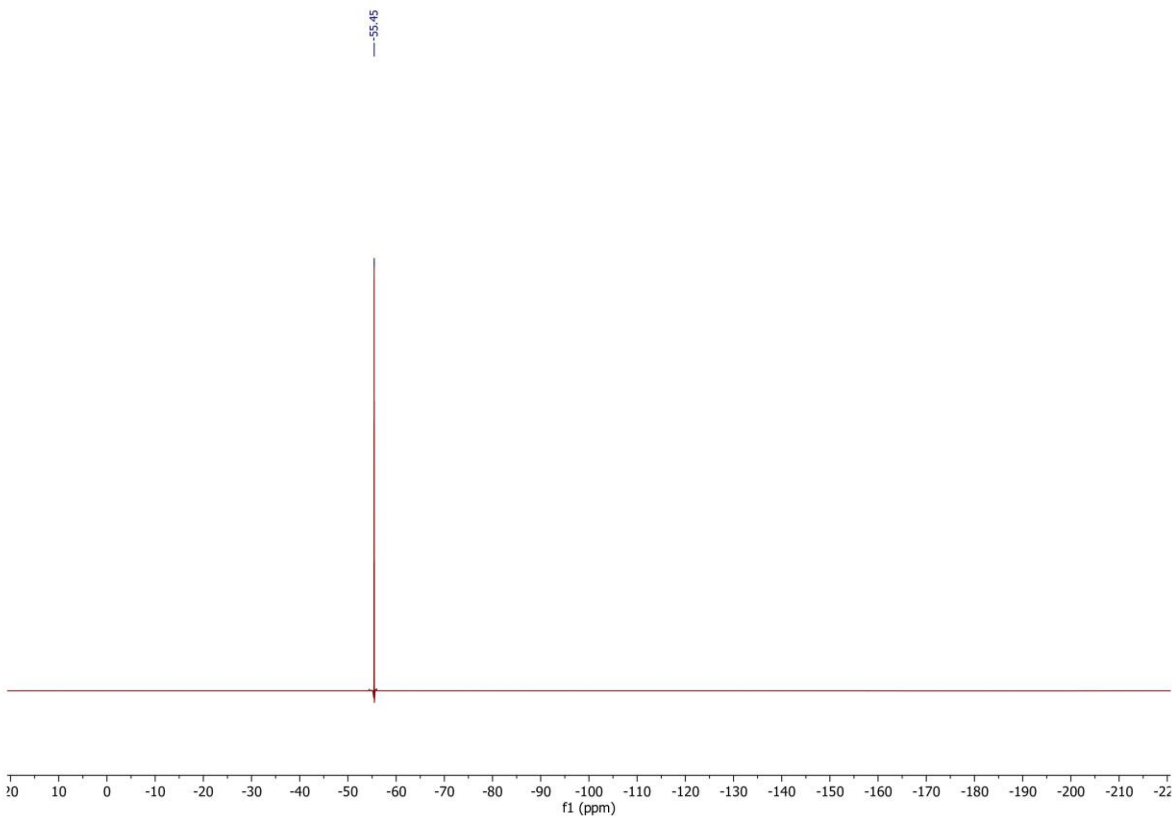
-55.45
20 10 0 -10 -20 -30 -40 -50 -60 -70 -80 -90 -100 -110 -120 -130 -140 -150 -160 -170 -180 -190 -200 -210 -220
f1 (ppm)

_N_-(3,4-Dimethoxyphenethyl)-_N_-methyl-4-nitrobenzenesulfonamide (S6)

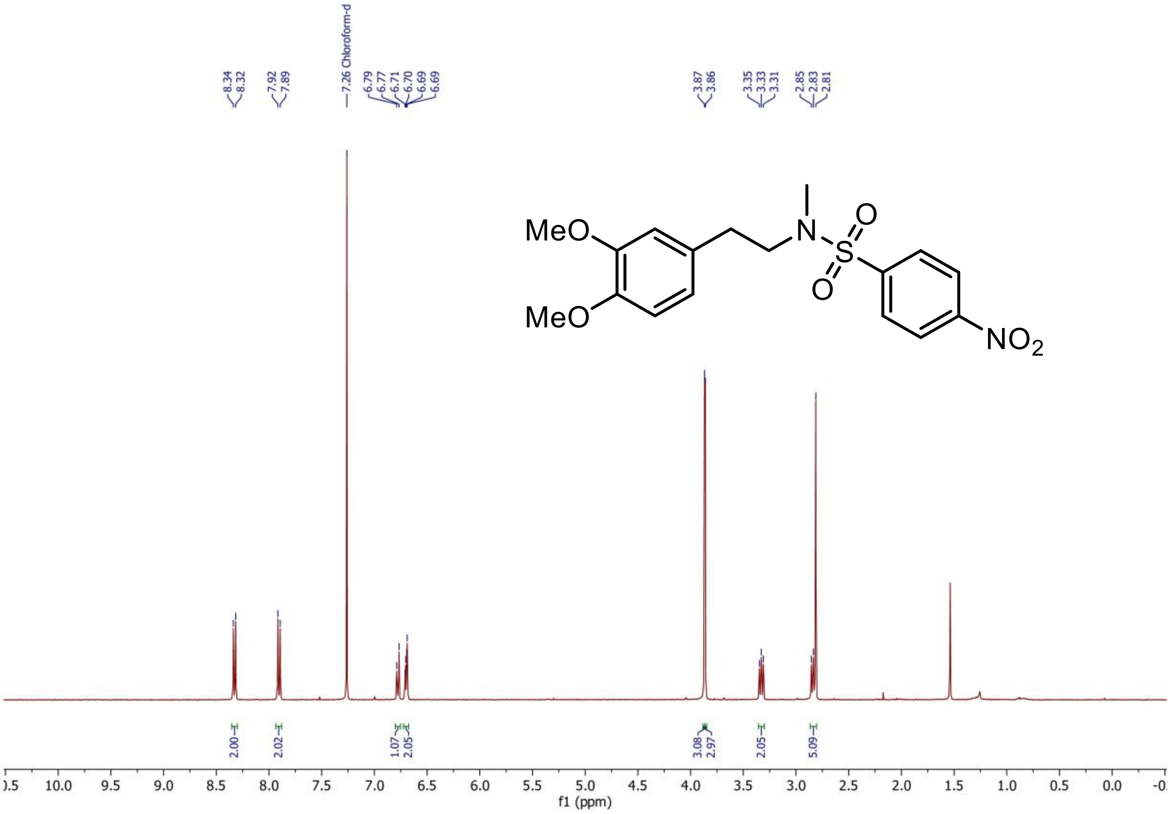

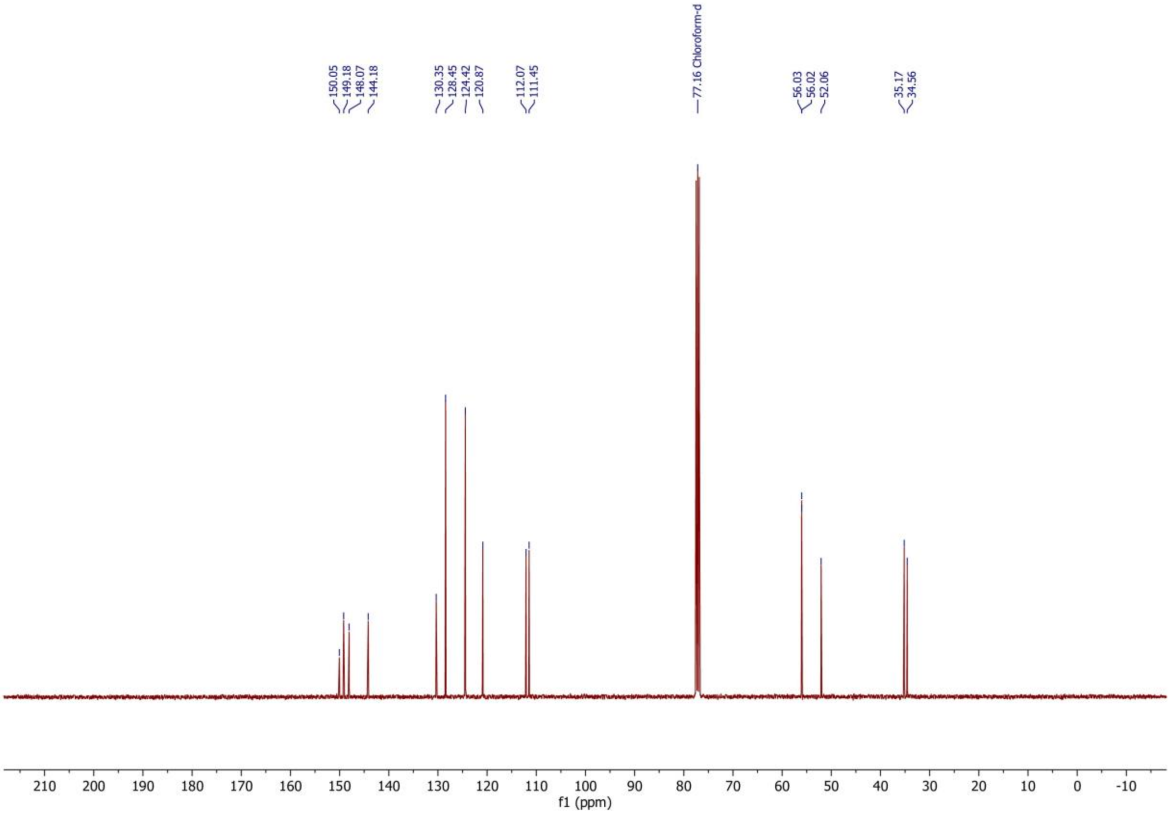

N-(3,4-Dimethoxyphenethyl)-N-methylacetamide (9a)

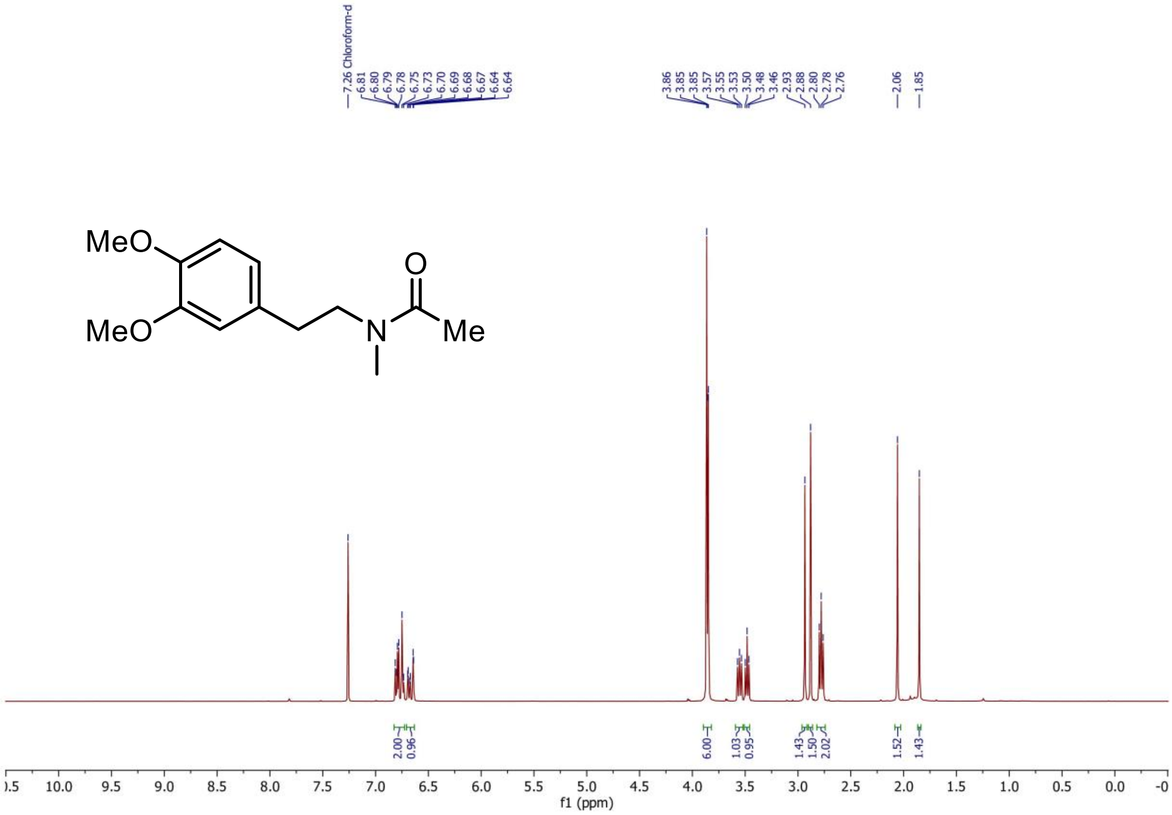

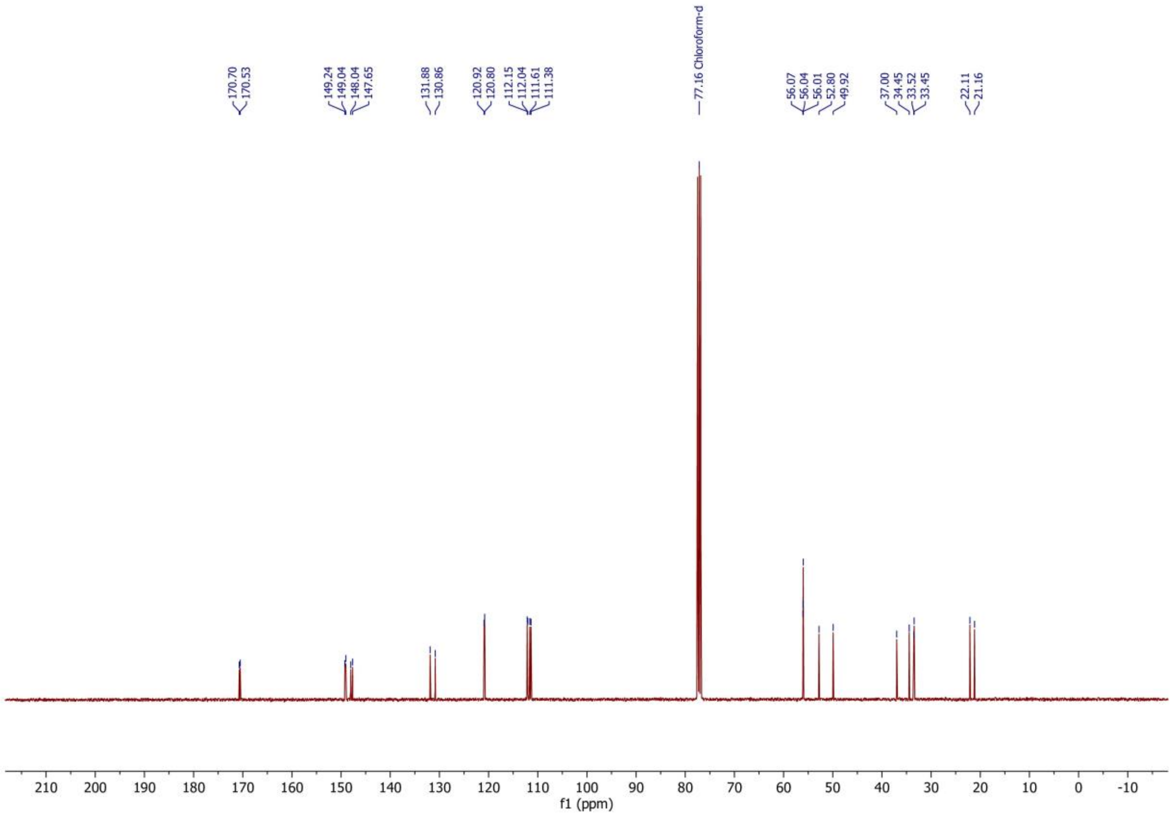

_N_-(3,4-Dimethoxyphenethyl)-_N_,3,7-trimethyloct-6-enamide (9b)

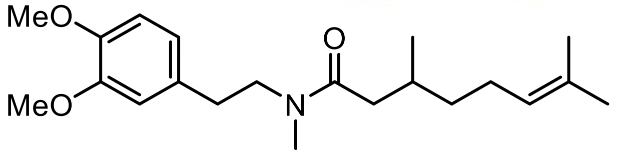

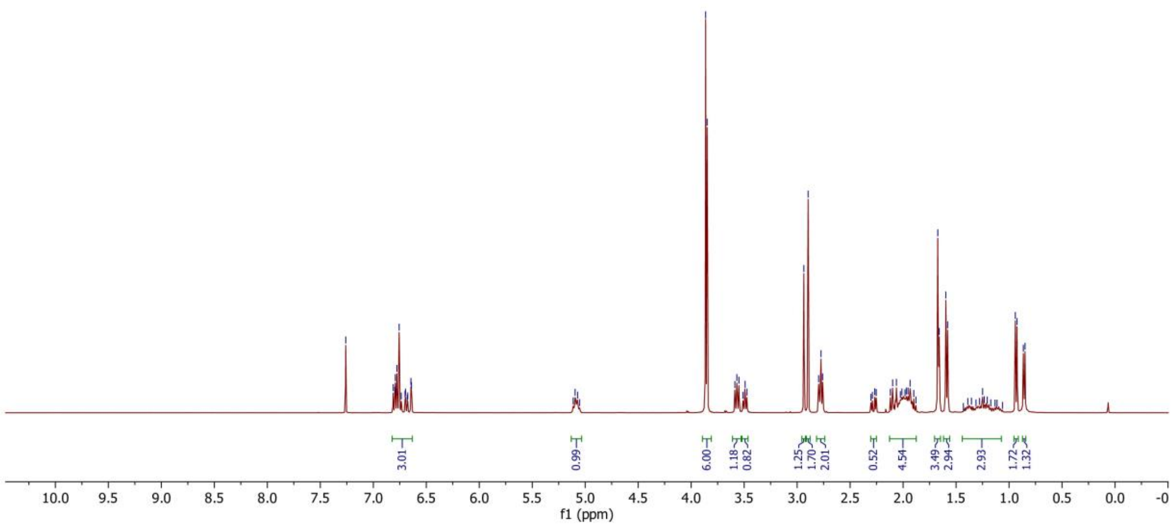

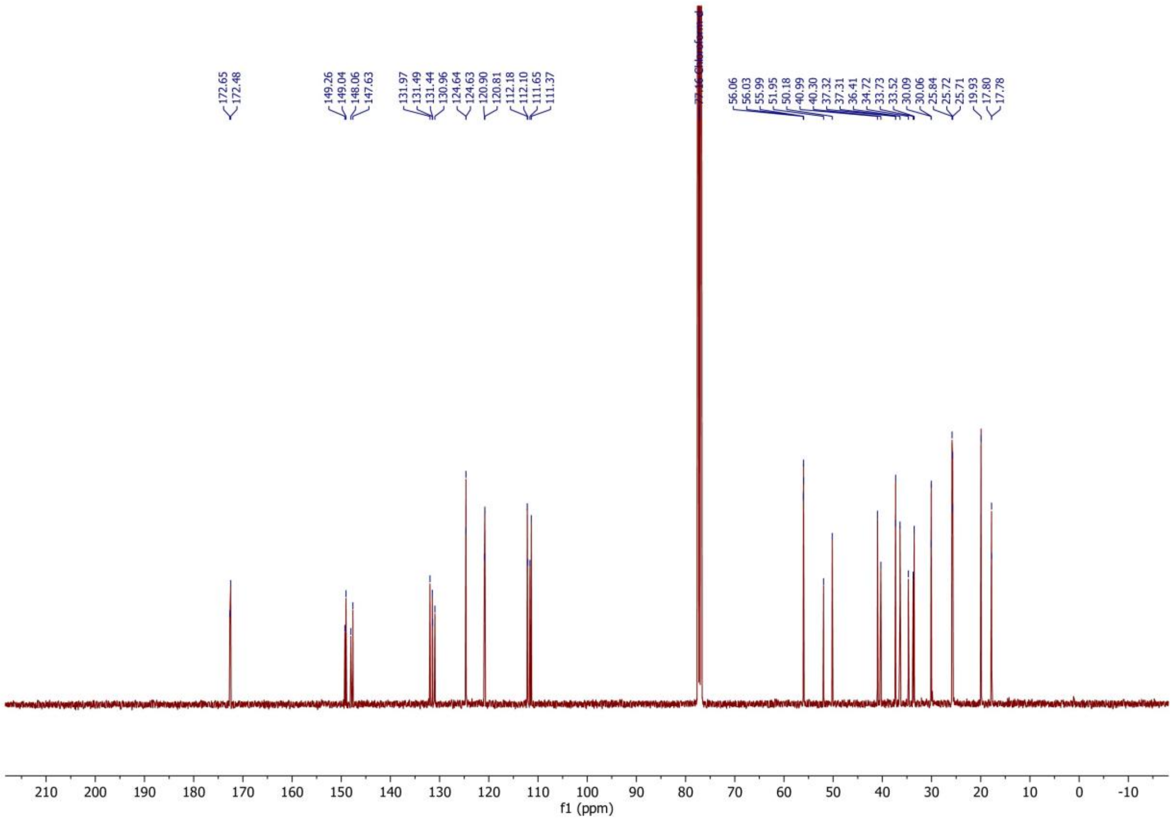

tert-Butyl 3-((3,4-dimethoxyphenethyl)(methyl)carbamoyl)azetidine-1-carboxylate (9c)

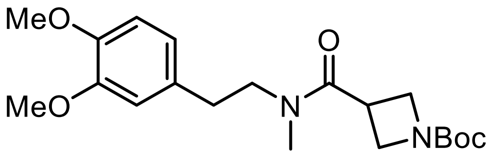

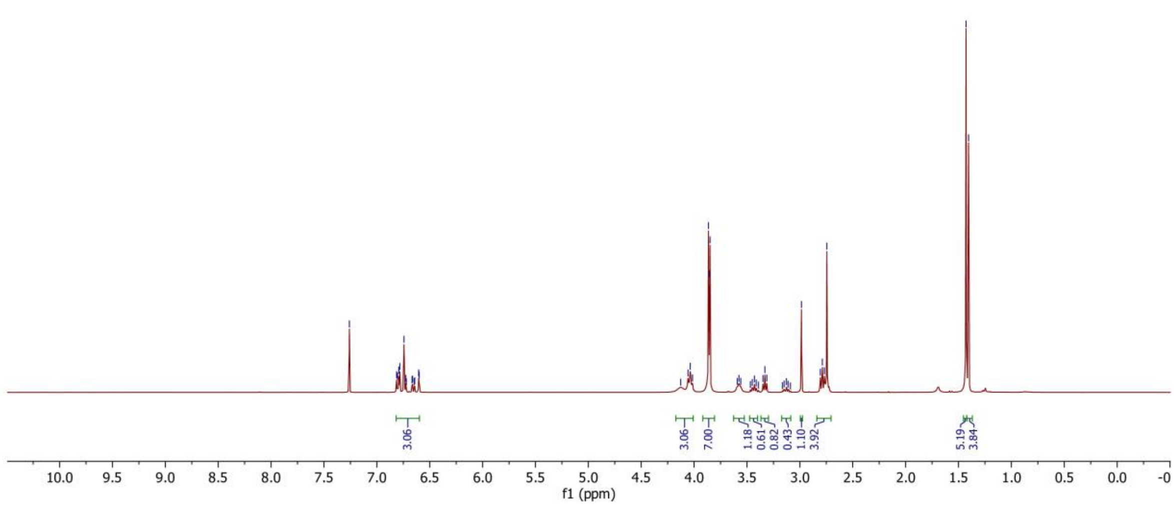

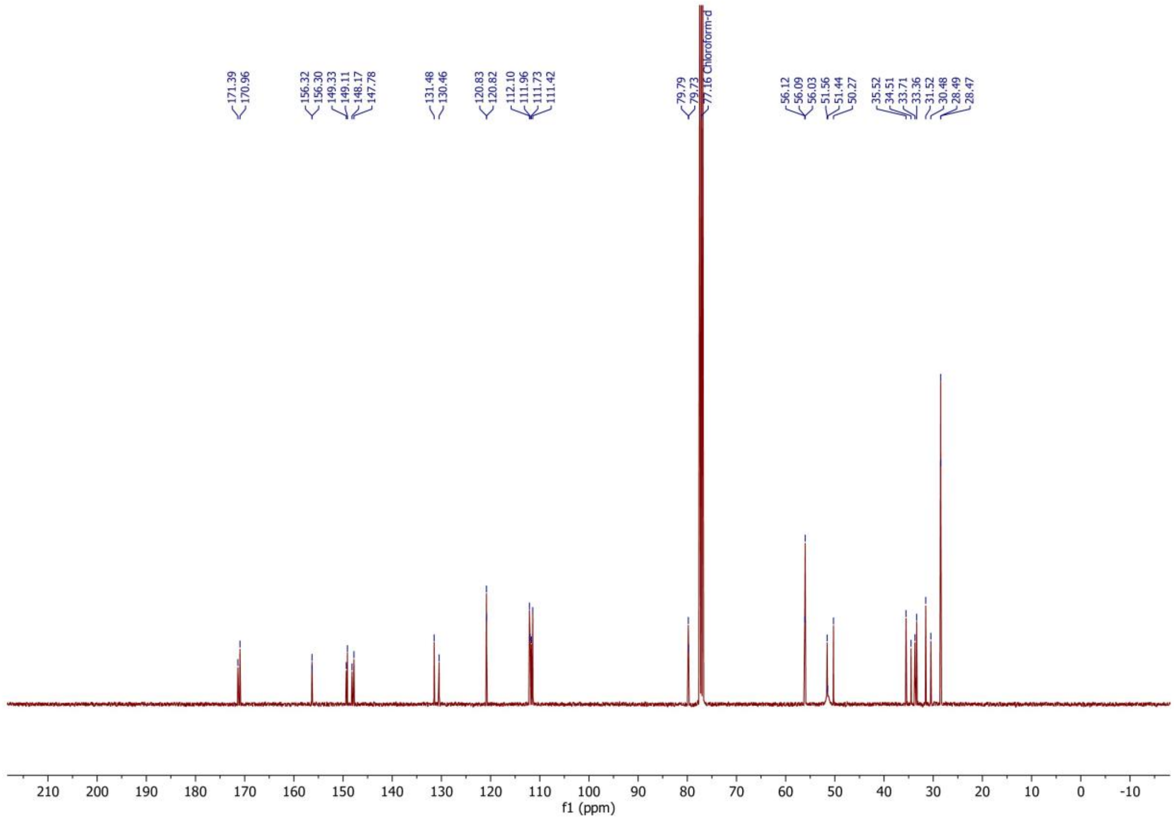

***N*-(3,4-Dimethoxyphenethyl)-*N*-methylcinnamamide (9d)**

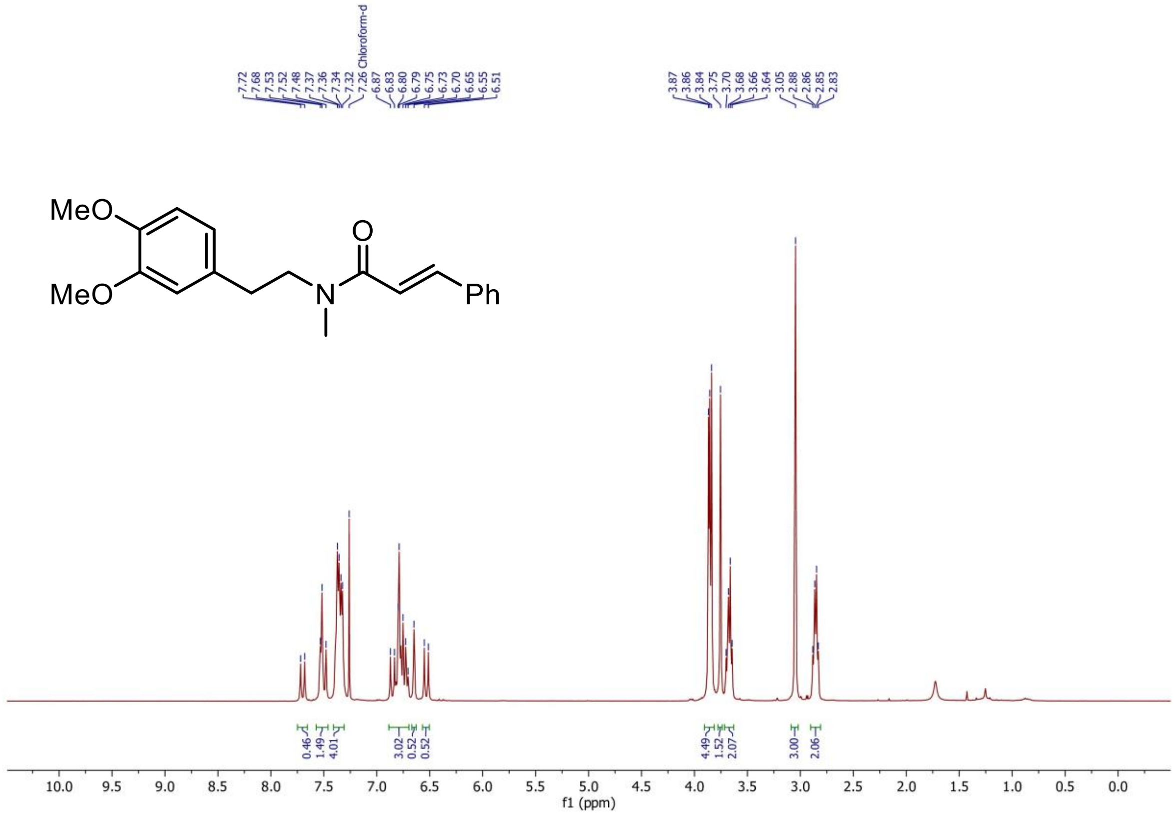

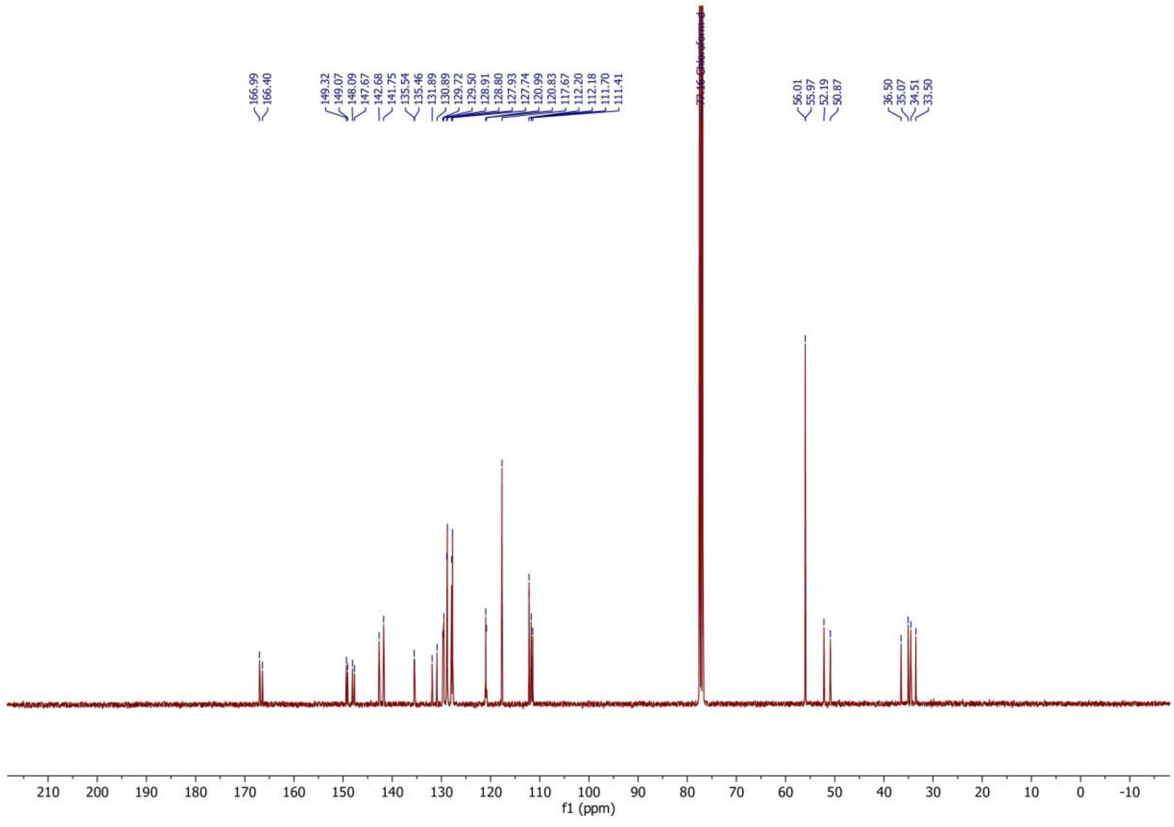

4-Chloro-*N*-(3,4-dimethoxyphenethyl)-*N*-methylbenzamide (9e)

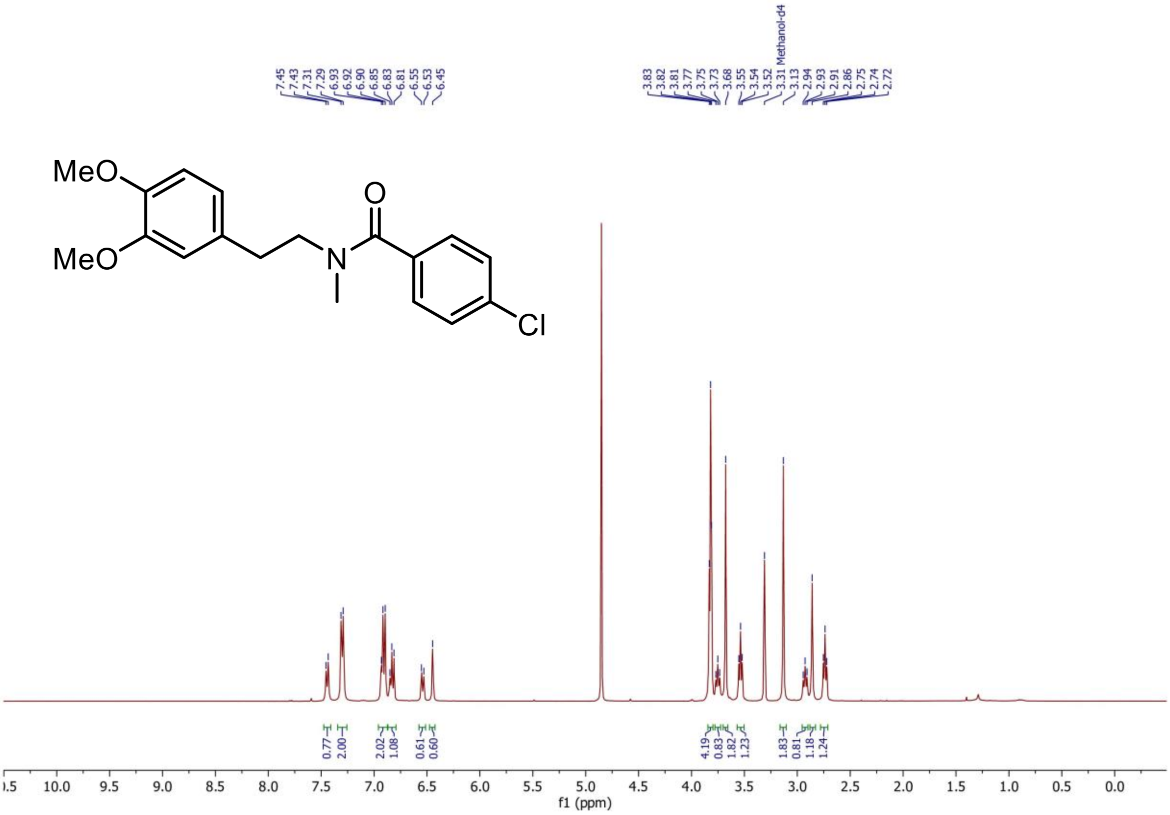

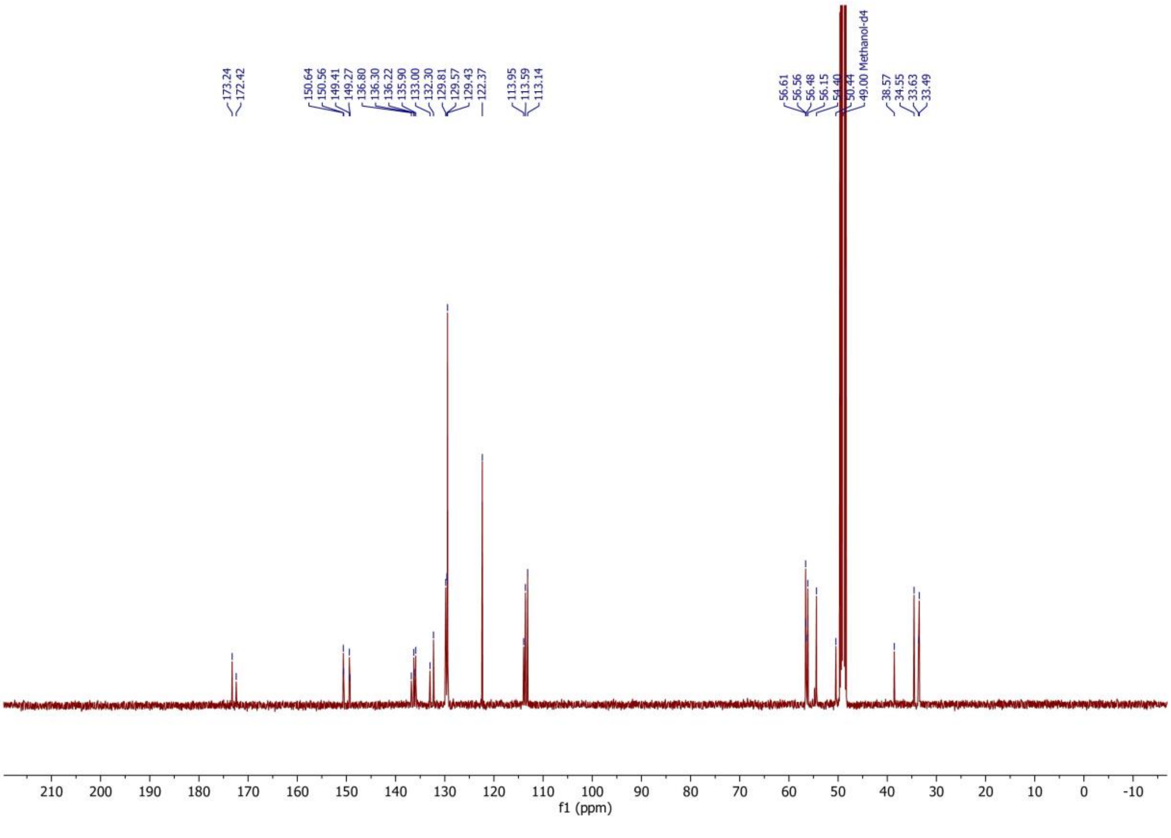

_N_-(3,4-Dimethoxyphenethyl)-_N_-methyl-5-(trifluoromethyl)picolinamide (9f)

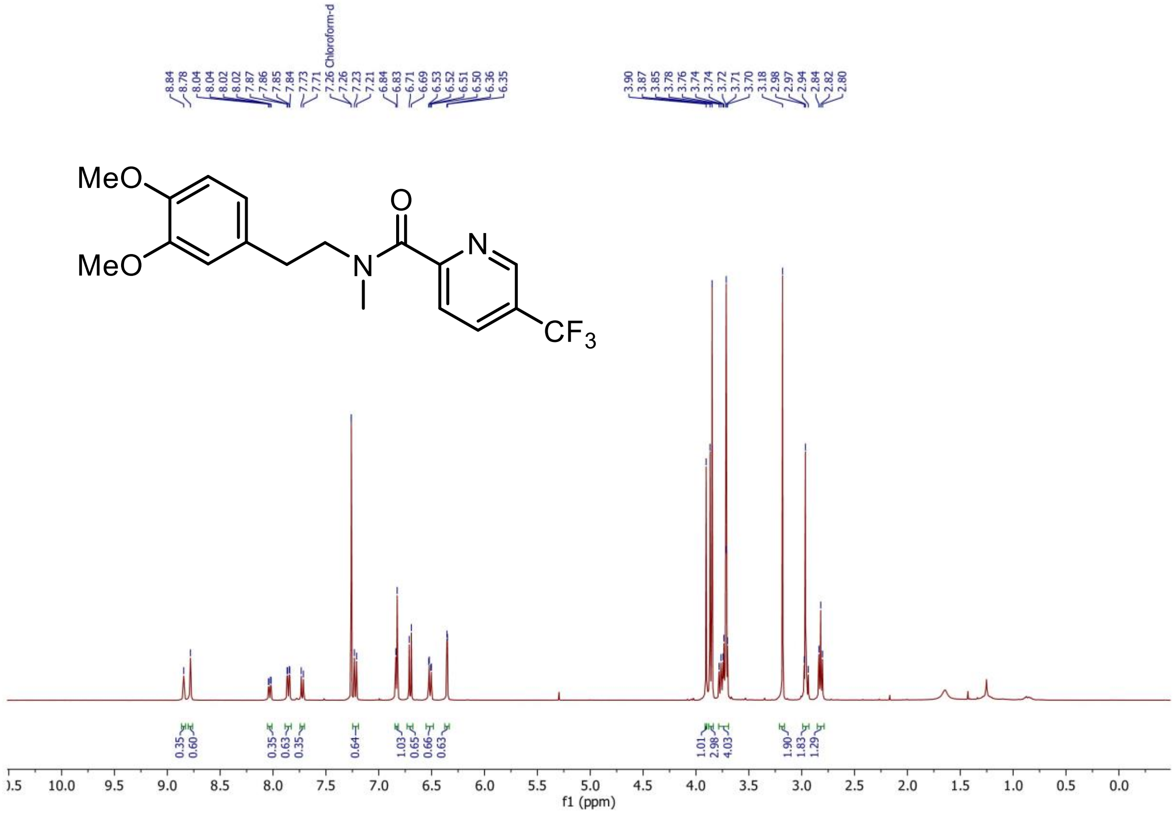

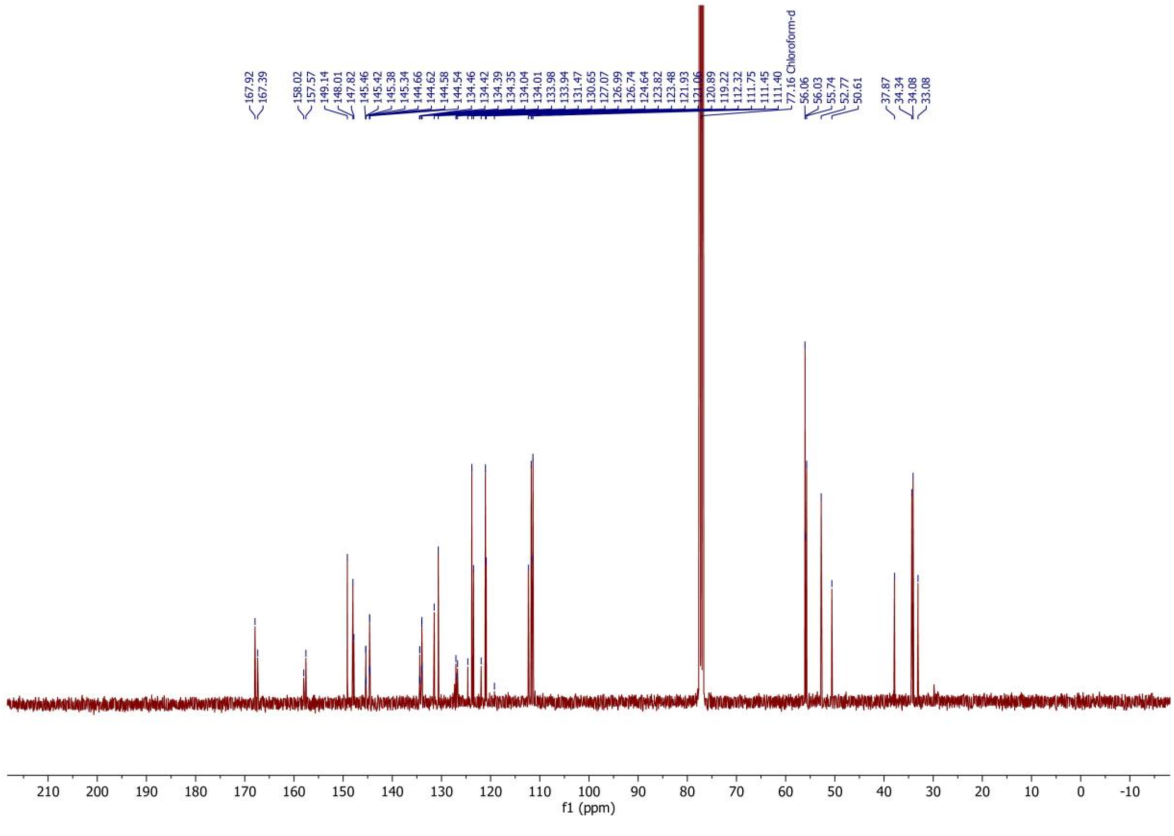

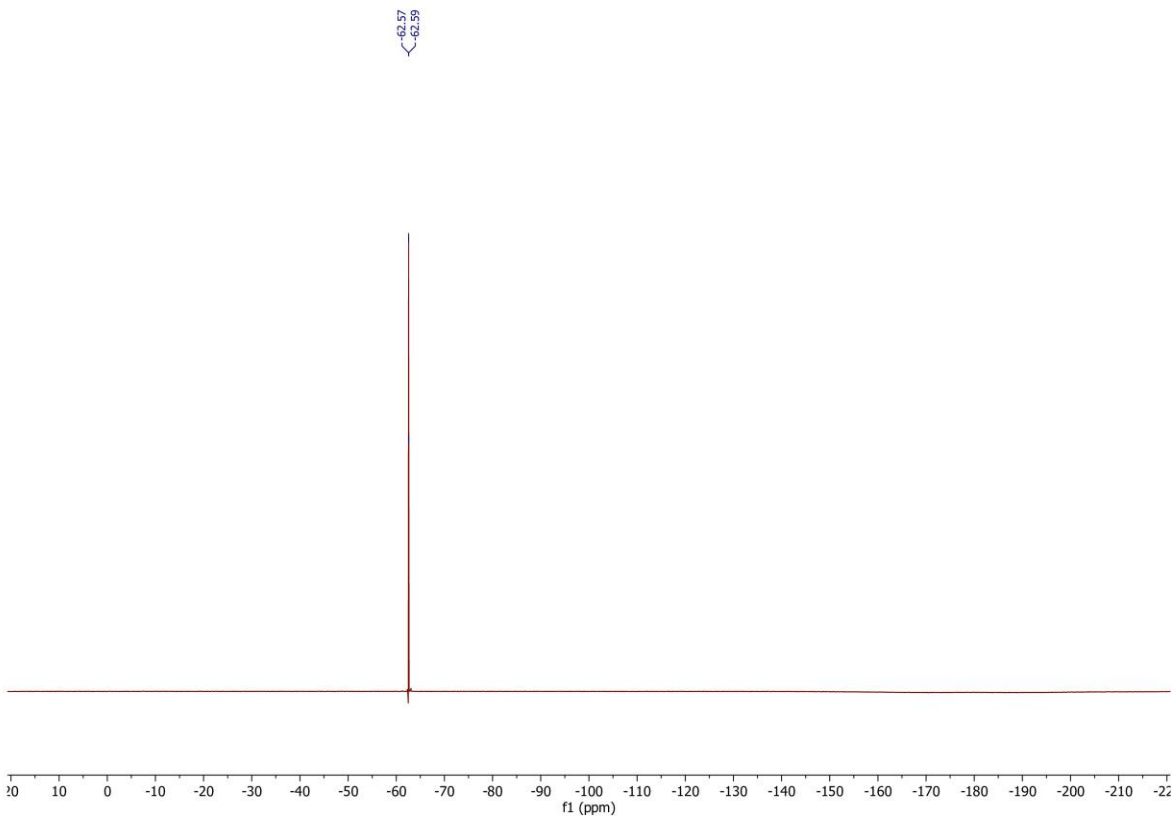
-62.57
-62.59
20 10 0 -10 -20 -30 -40 -50 -60 -70 -80 -90 -100 -110 -120 -130 -140 -150 -160 -170 -180 -190 -200 -210 -22
f1 (ppm)

***N*-(3,4-Dimethoxyphenethyl)-N-methyl-7-(trimethylsilyl)hept-6-ynamide (9g)**

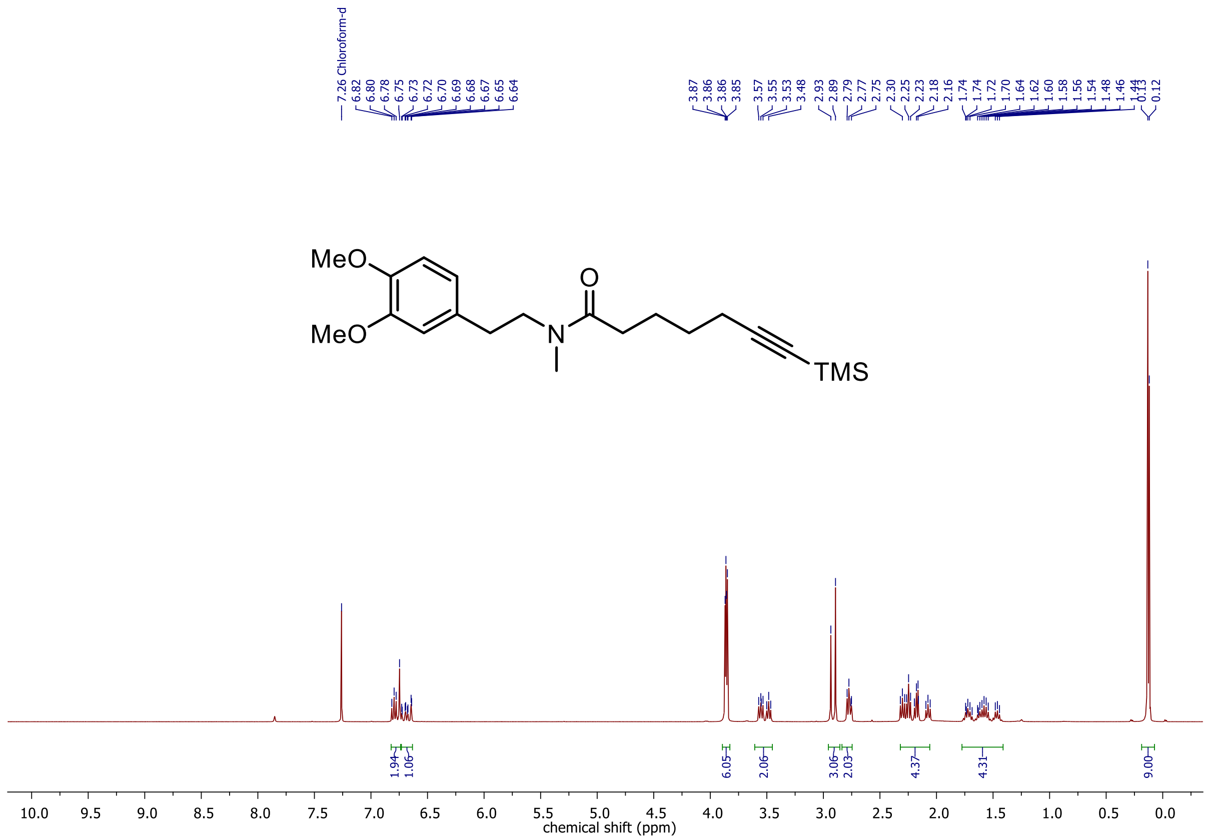

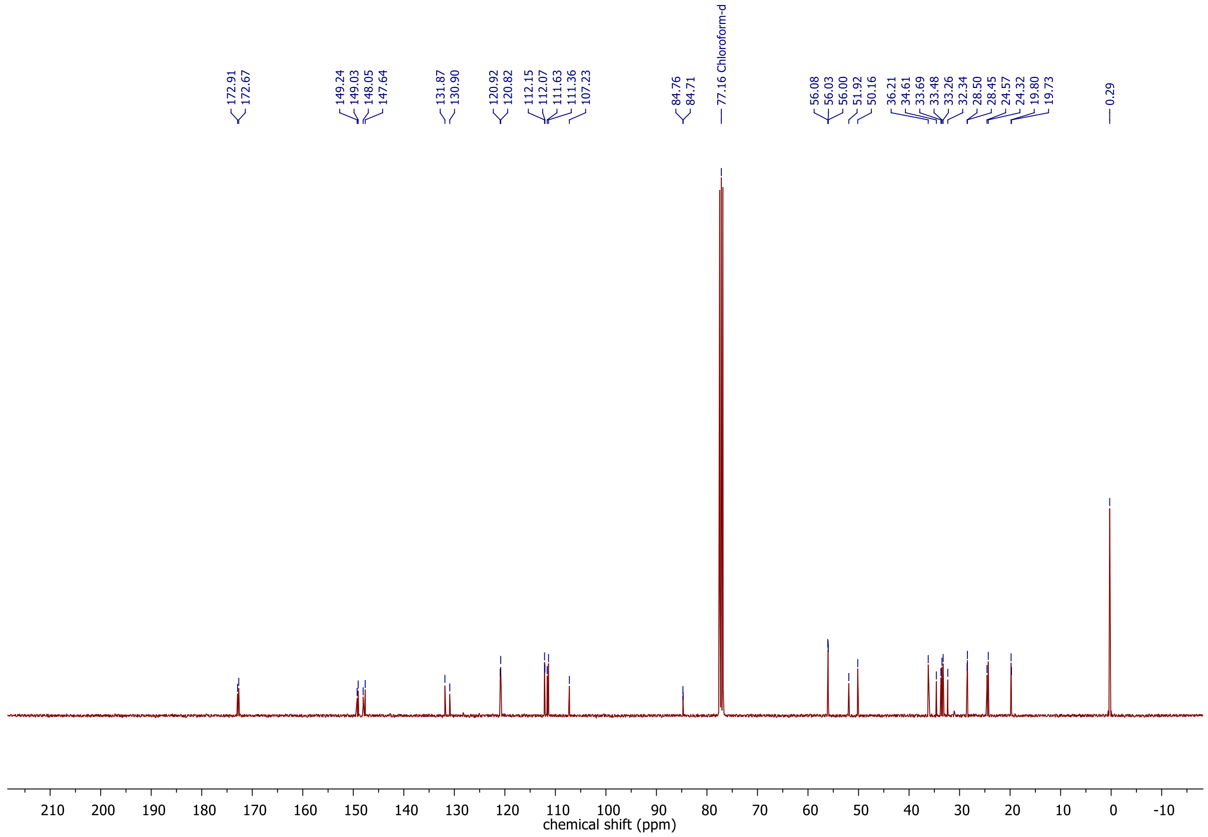

***N*-(3,4-Dimethoxyphenethyl)-*N*-methylpyrazolo[1,5-*a*]pyridine-3-carboxamide (9h)**

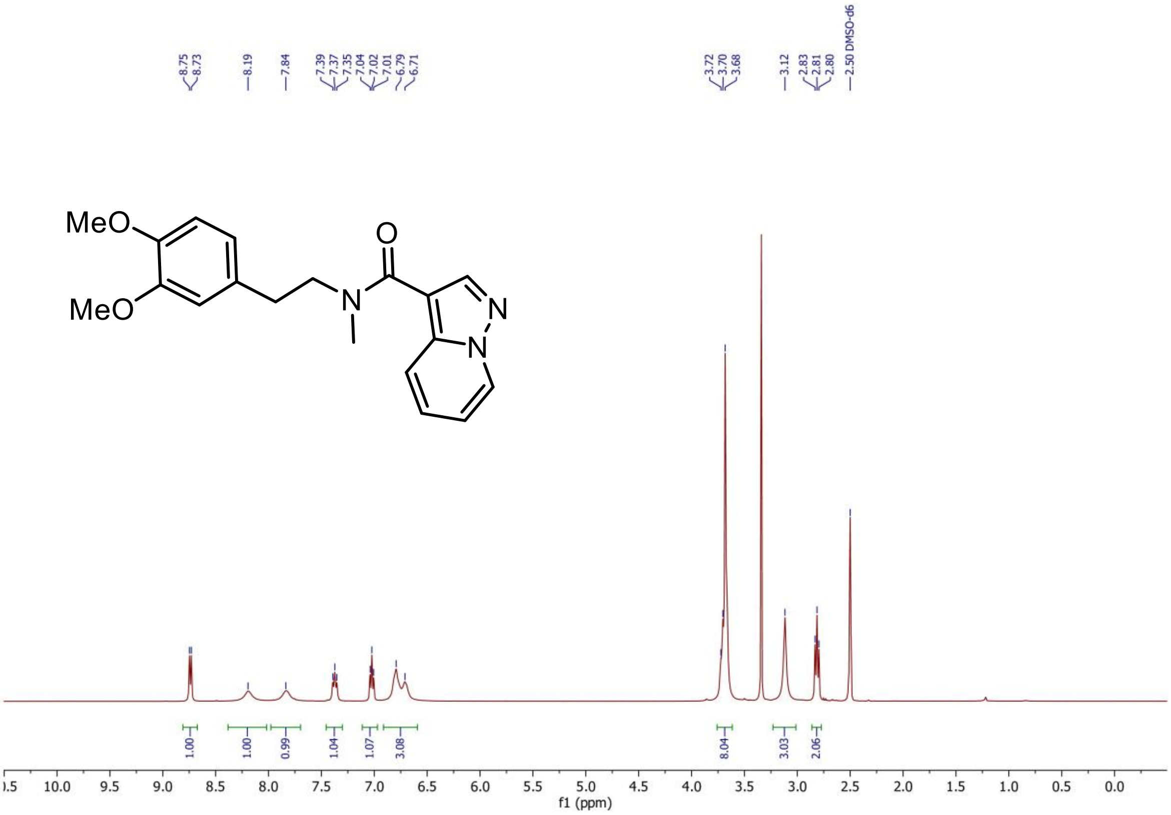

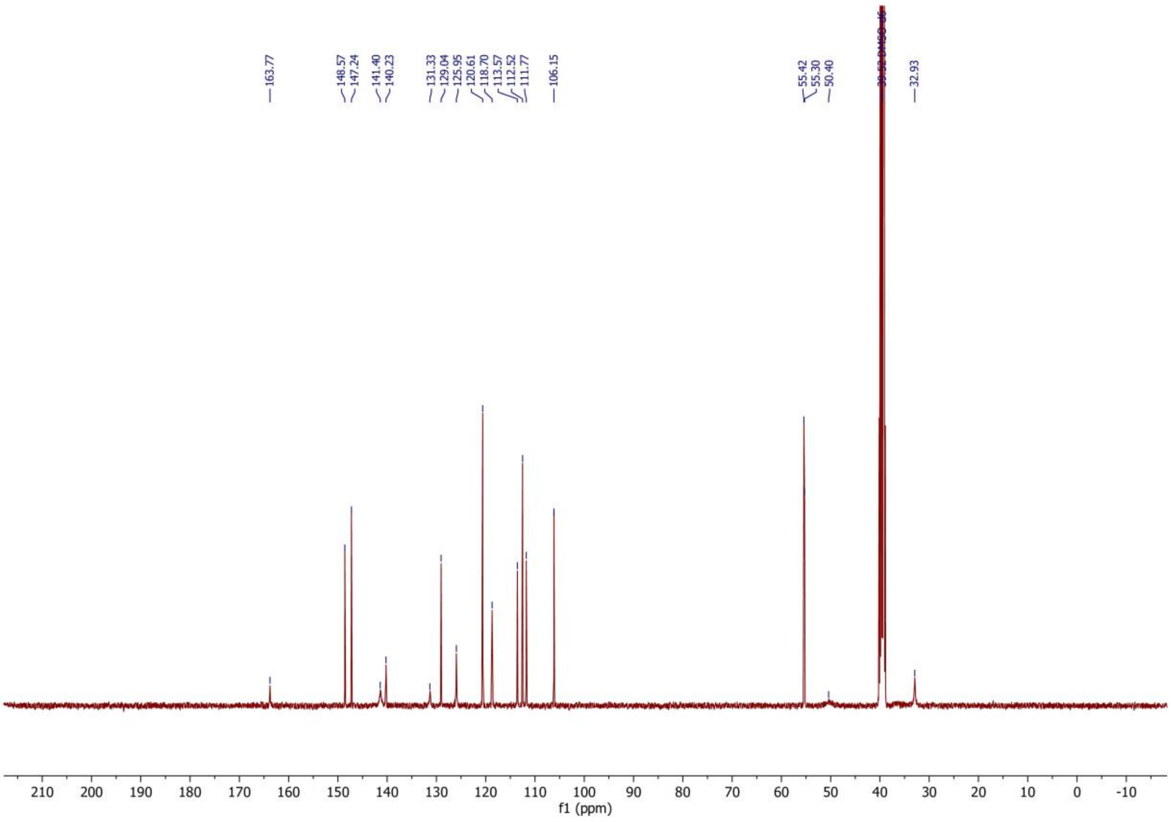

***tert*-Butyl (2*S*,4*R*)-2-((3,4-dimethoxyphenethyl)(methyl)carbamoyl)-4-hydroxypyrrolidine-1-carboxylate (9i)**

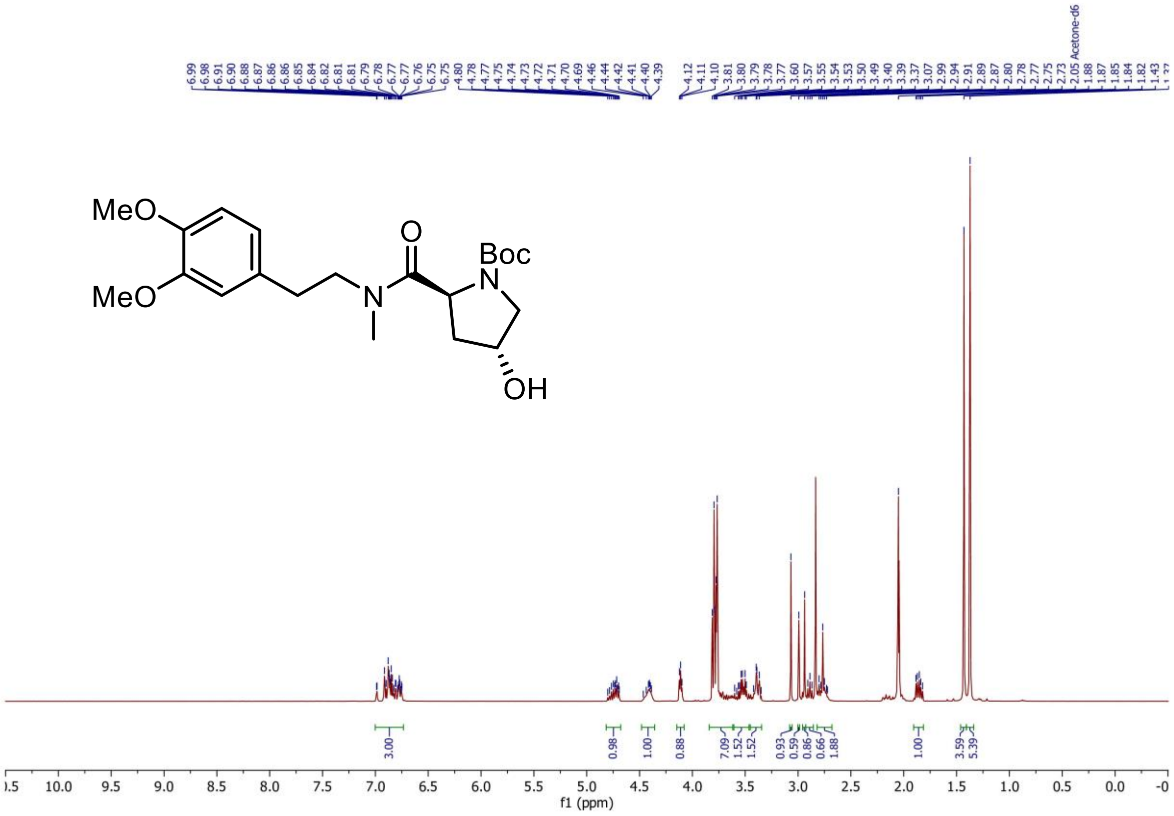

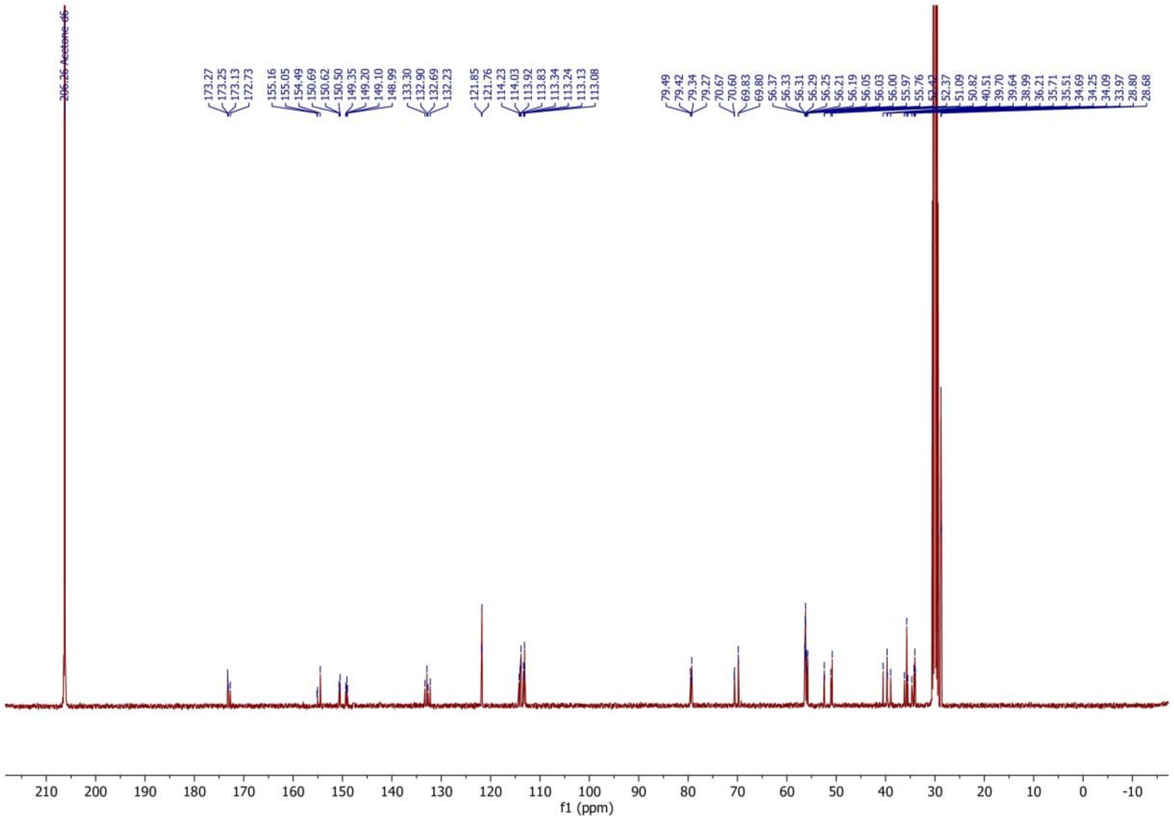

***tert*-Butyl (2*S*,4*R*)-2-((3,4-dimethoxyphenethyl)(methyl)carbamoyl)-4-hydroxypyrrolidine-1-carboxylate (9i)**

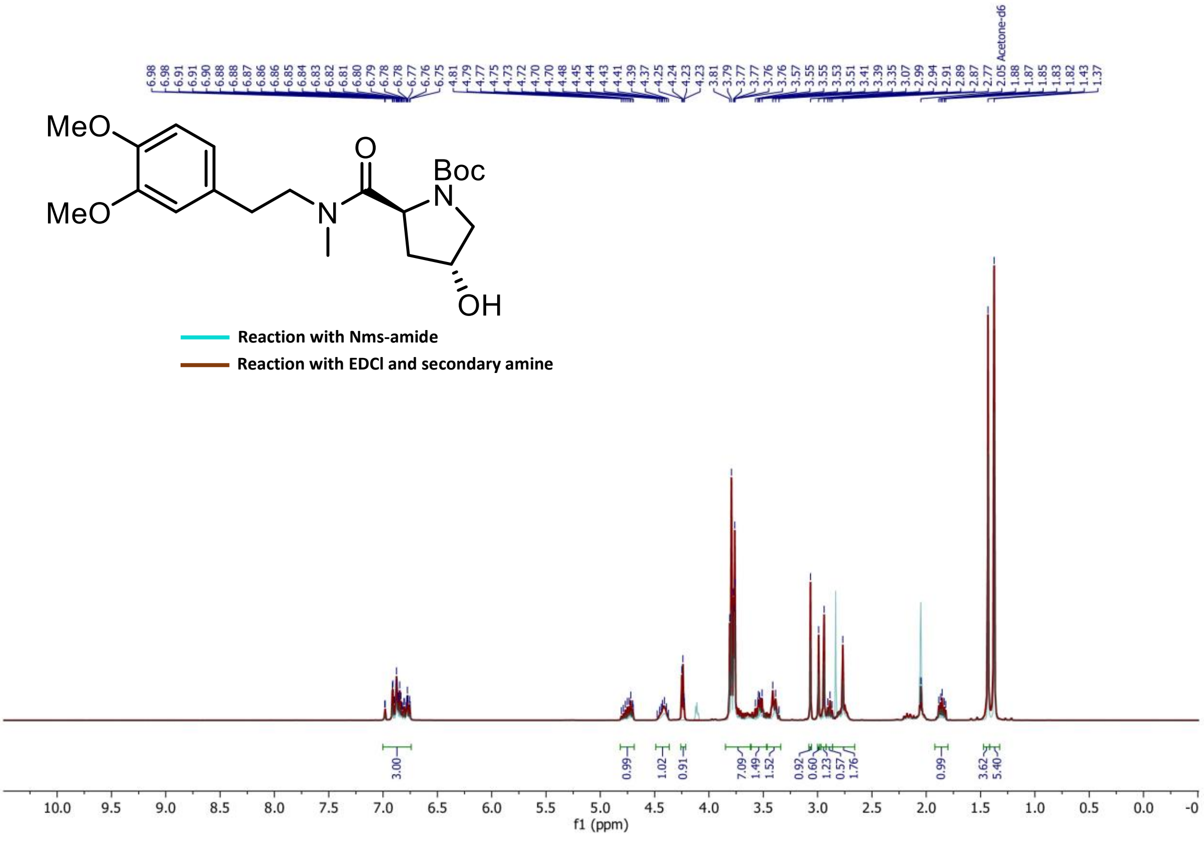

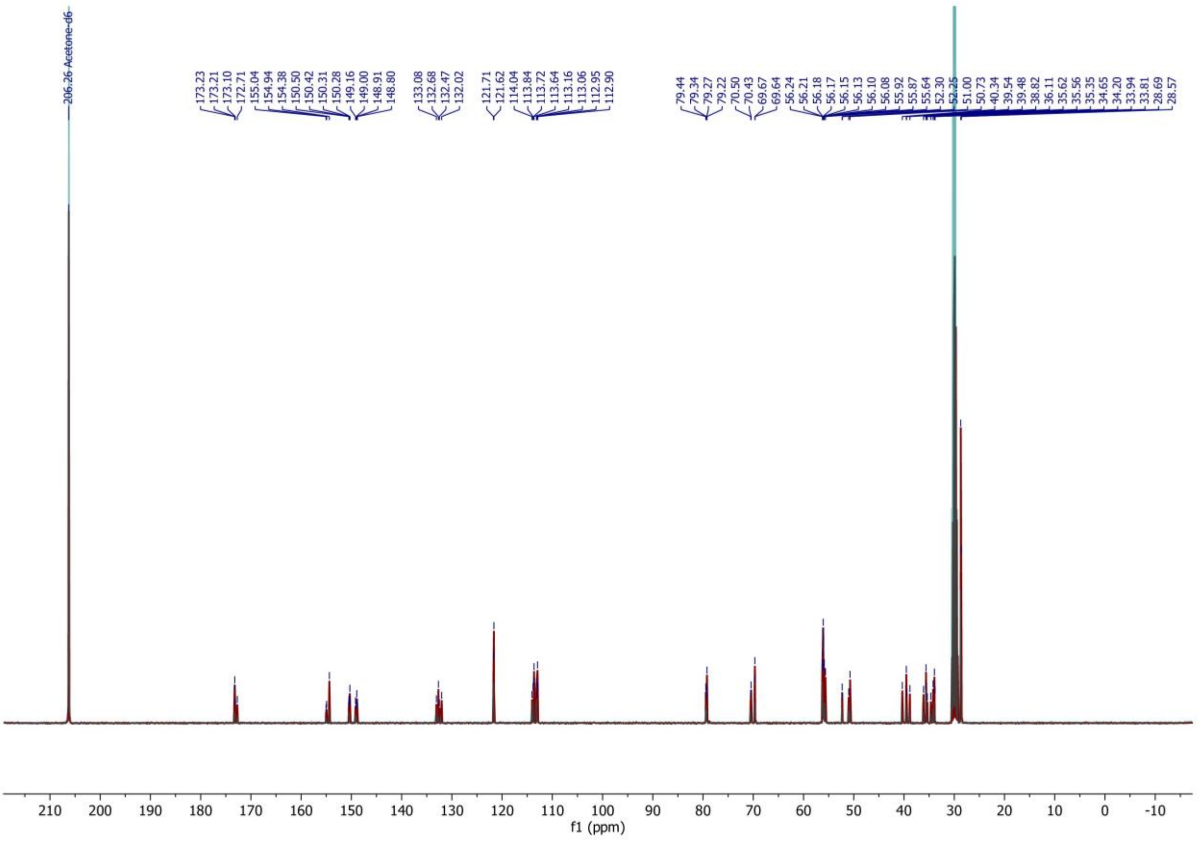

Tert-butyl (*S*)-2-((3,4-dimethoxyphenethyl)(methyl)carbamoyl)pyrrolidine-1-carboxylate (9j)

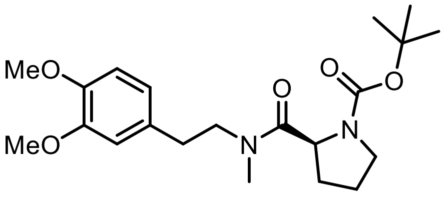

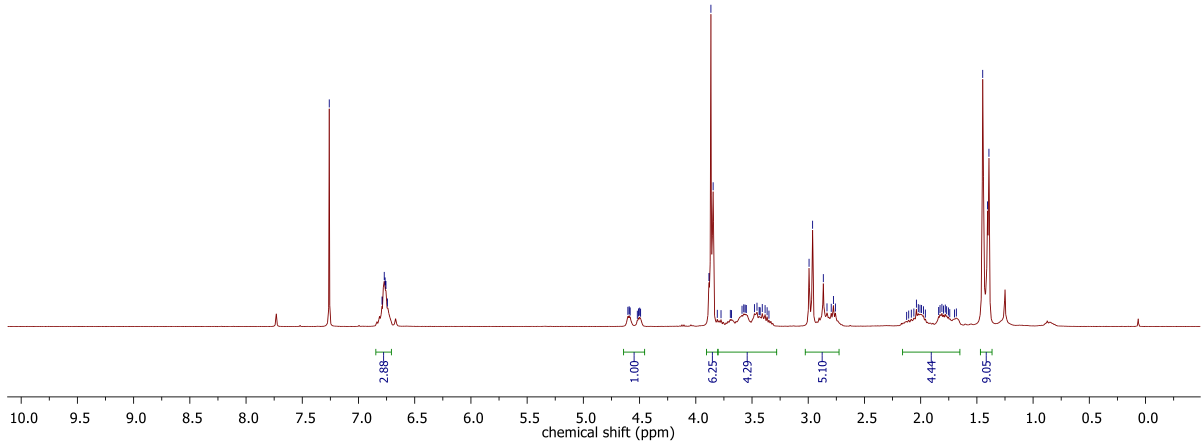

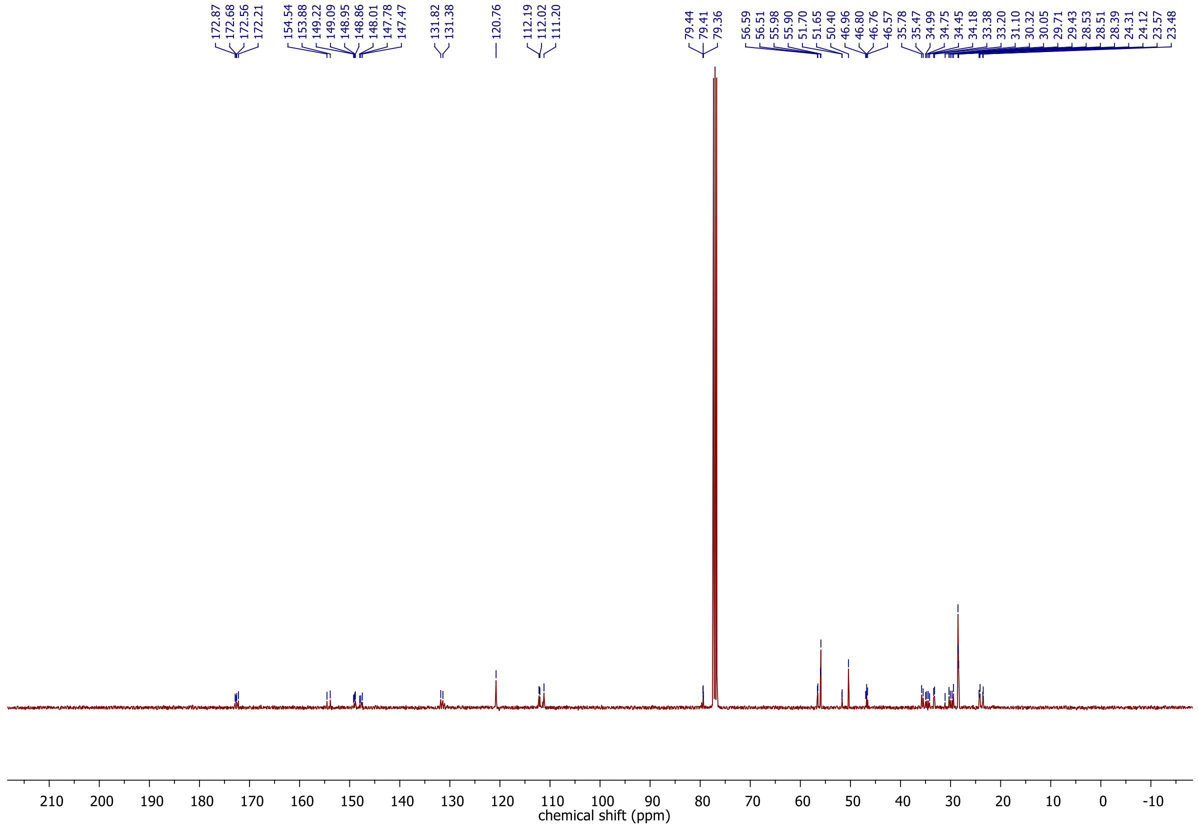

2-(3-Cyano-4-isobutoxyphenyl)-*N*-(3,4-dimethoxyphenethyl)-*N*,4-dimethylthiazole-5-carboxamide (9k)

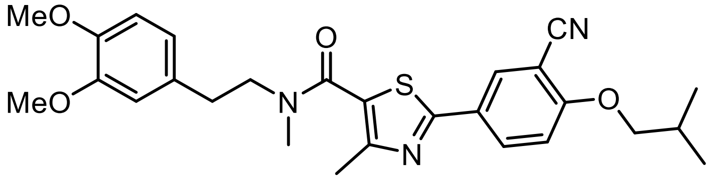

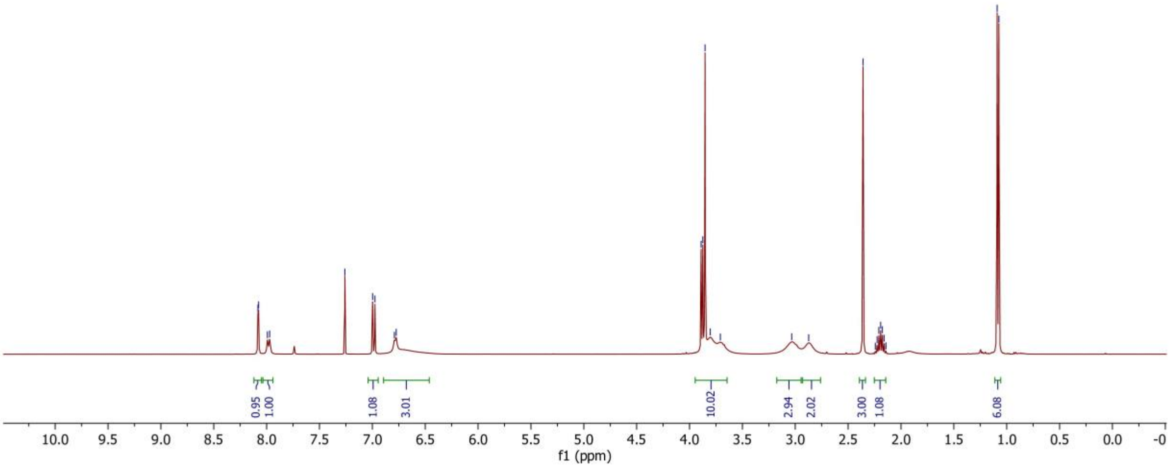

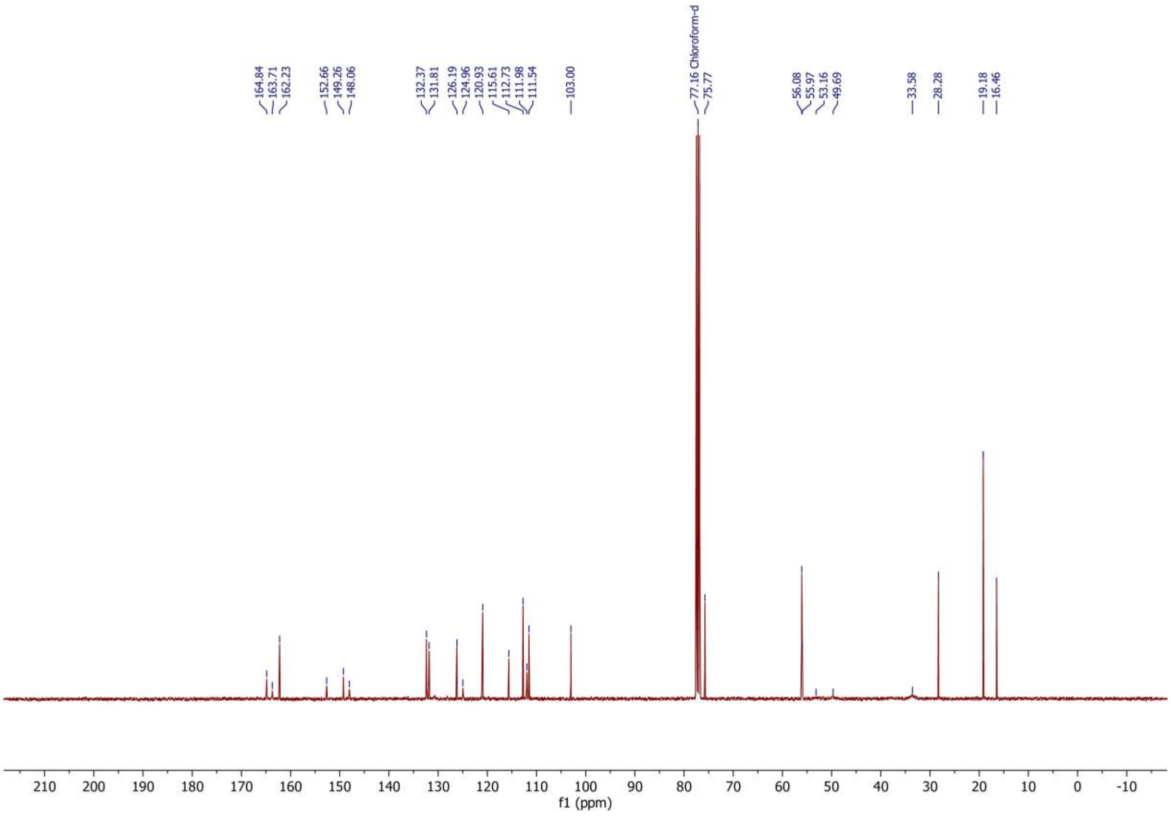

N-(3,4-Dimethoxyphenethyl)-4-(N,N-dipropylsulfamoyl)-N-methylbenzamide (9l)

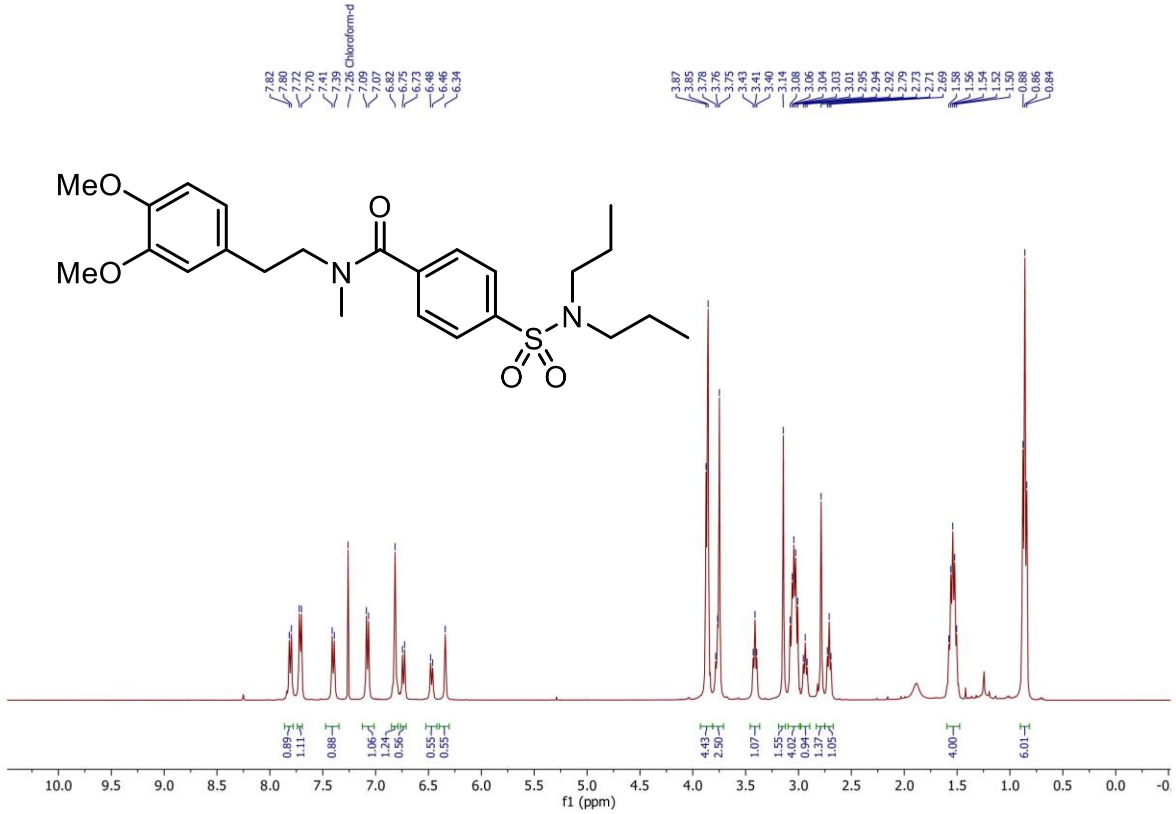

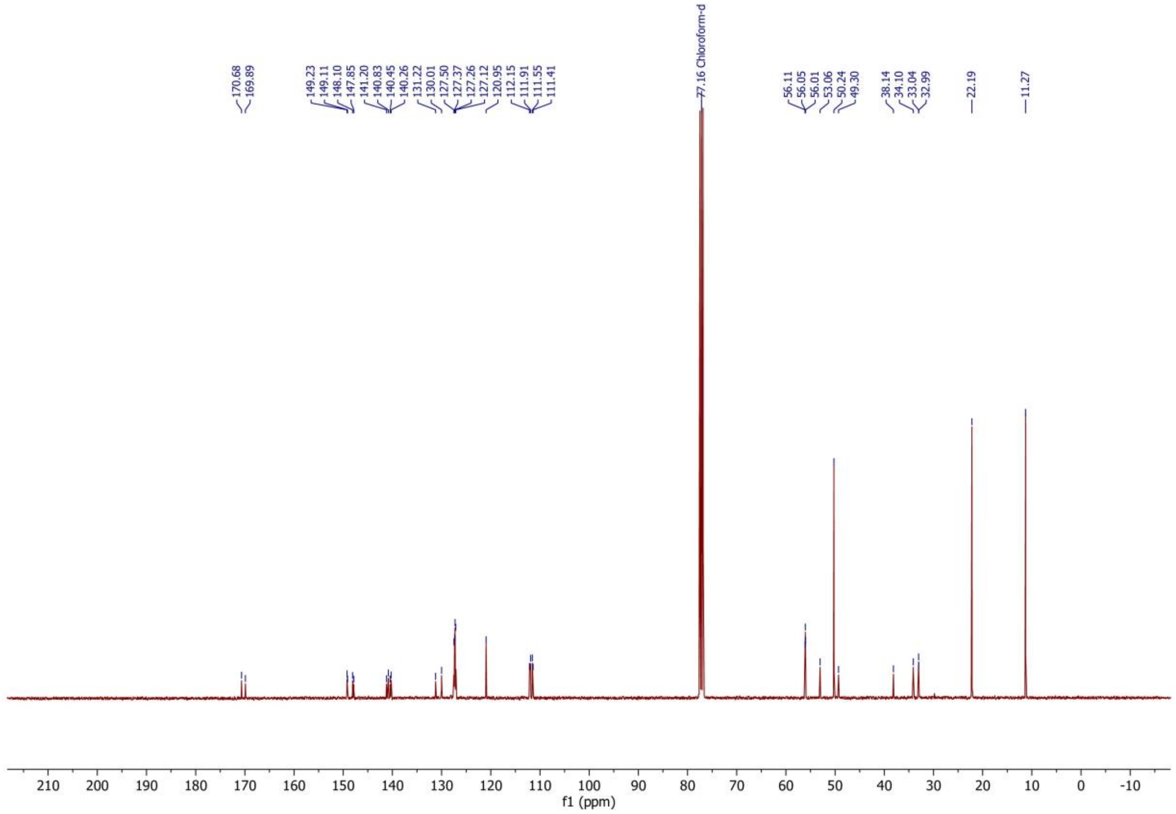

***tert*-Butyl 4-(1-cyclopropyl-3-((3,4-dimethoxyphenethyl)(methyl)carbamoyl)-6-fluoro-4-oxo-1,4-dihydroquinolin-7-yl)piperazine-1-carboxylate (9m)**

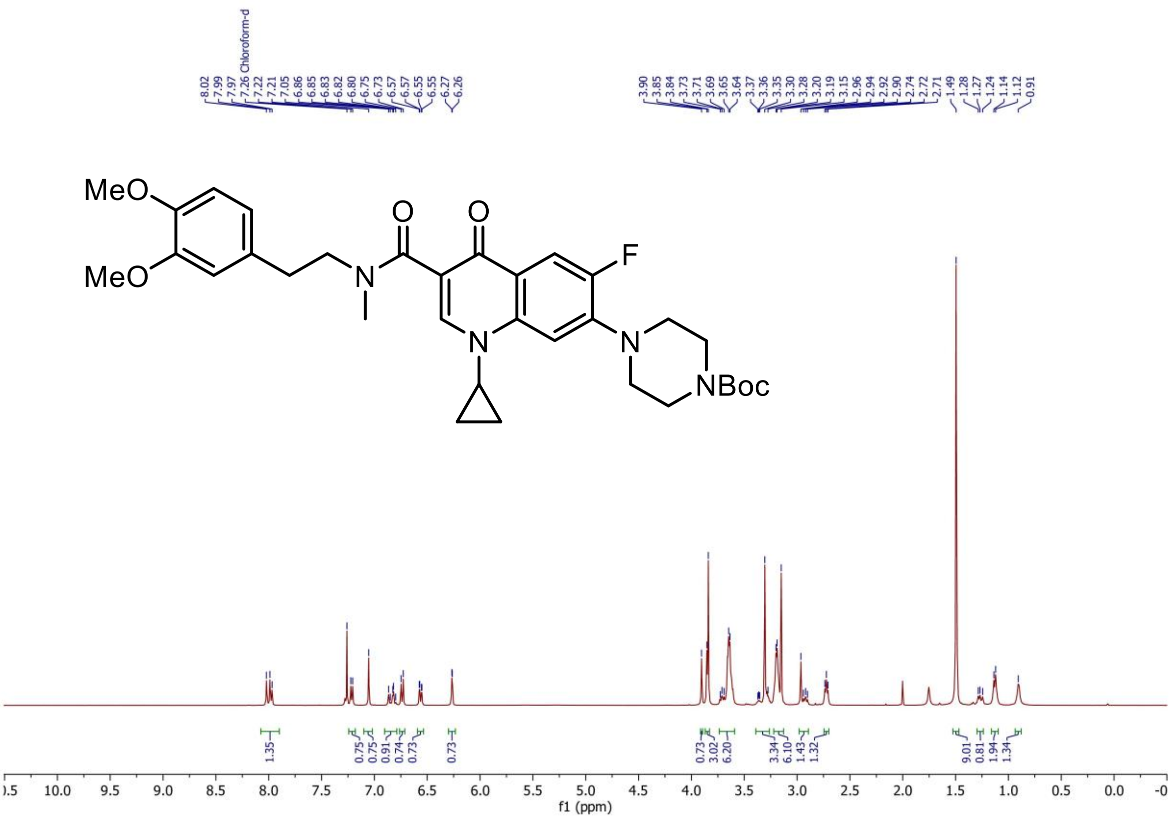

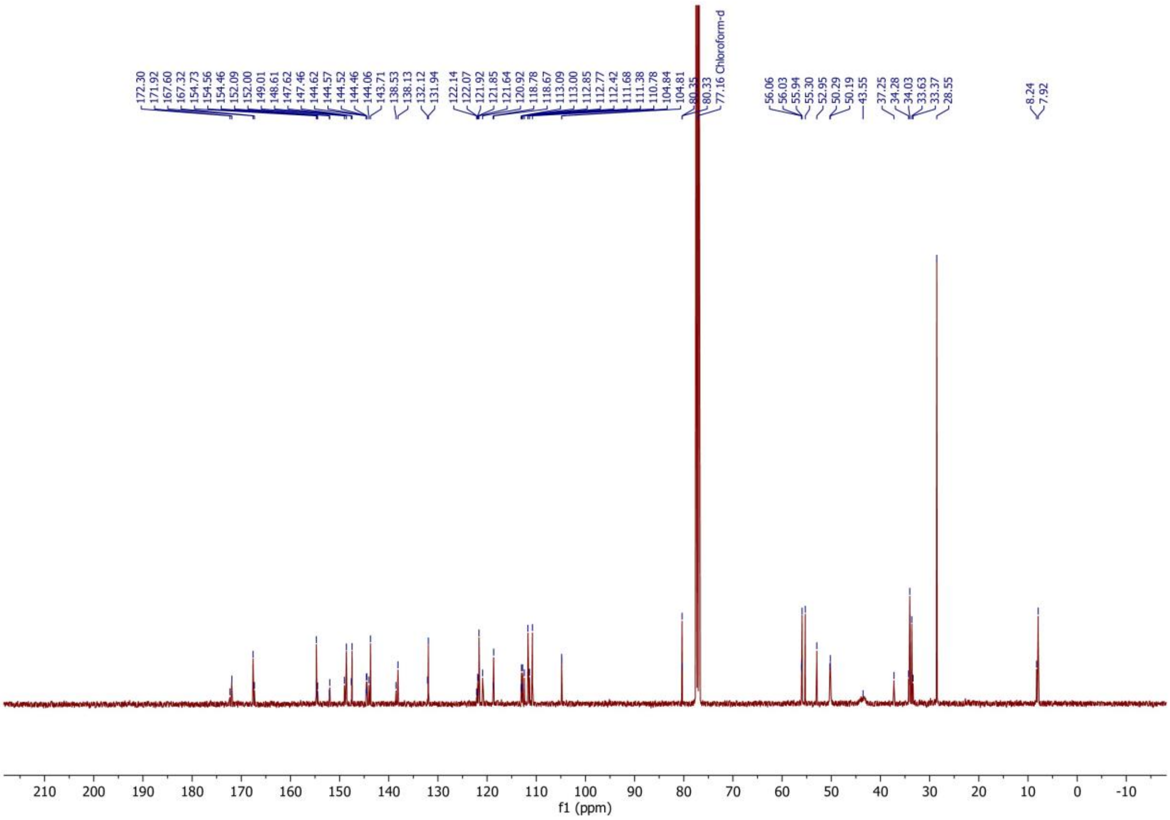

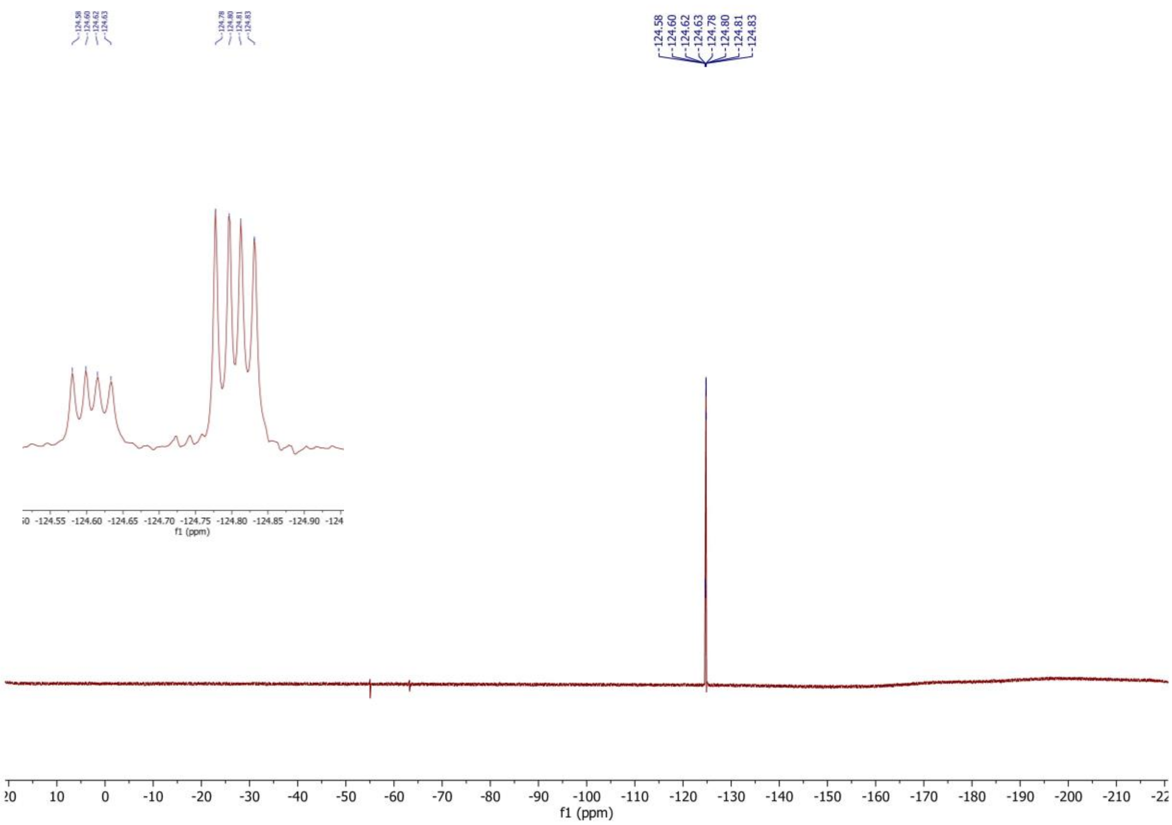

-124.58
-124.60
-124.62
-124.63
-124.78
-124.80
-124.81
-124.83
f1 (ppm)

(*Z*)-*N*-(3,4-Dimethoxyphenethyl)-2-(5-fluoro-2-methyl-1-(4-(methylsulfinyl)benzylidene)-1*H*-inden-3-yl)-*N*-methylacetamide (9n)

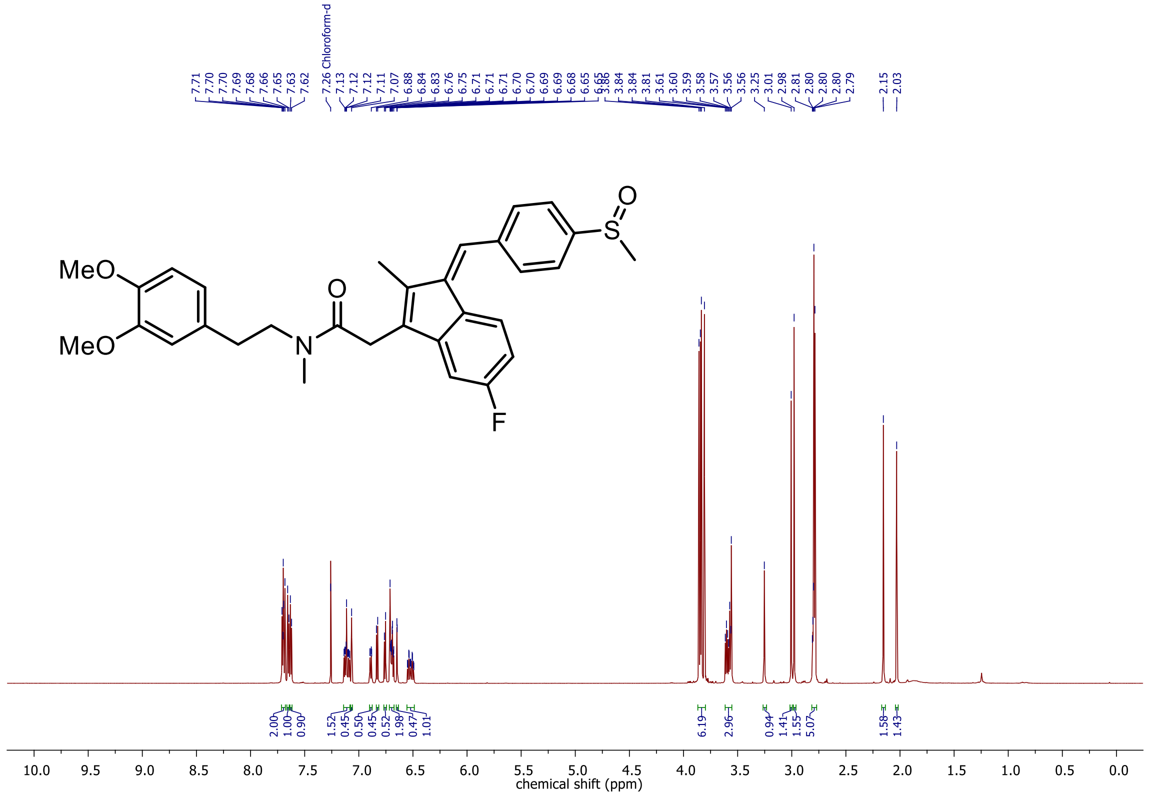

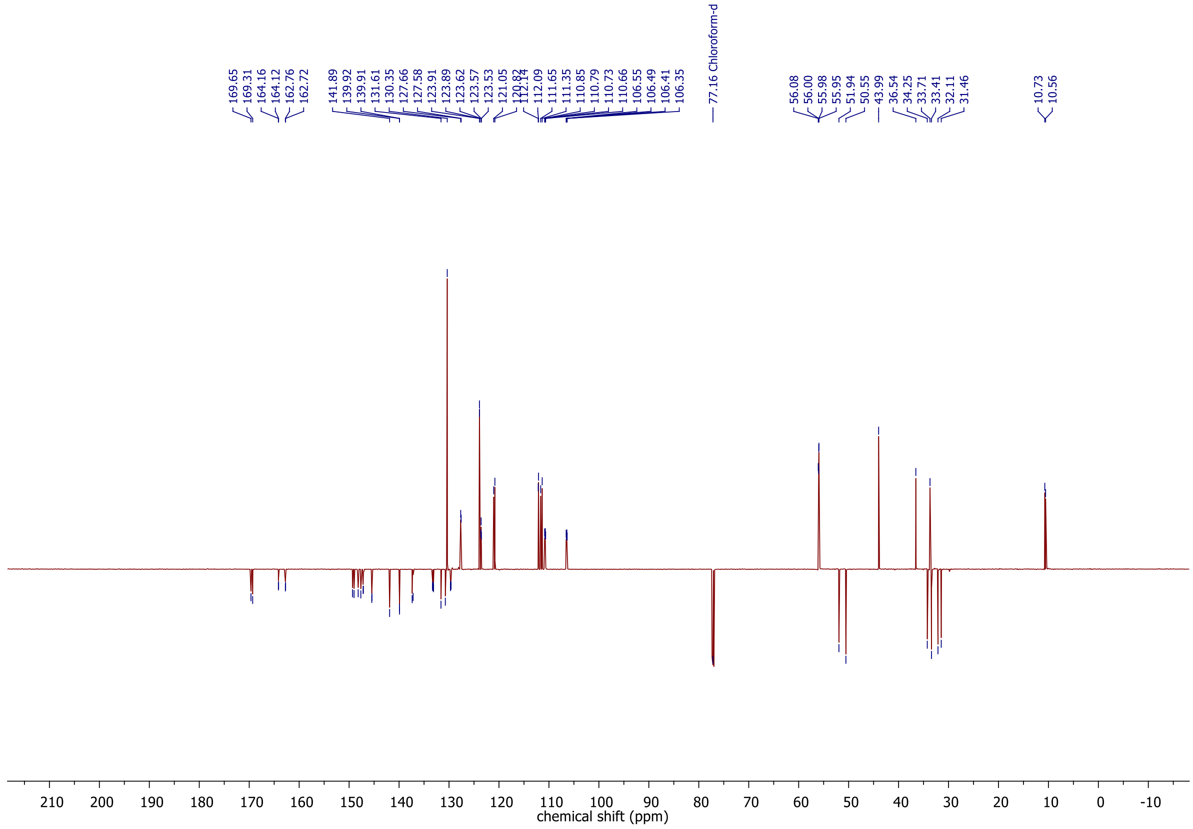

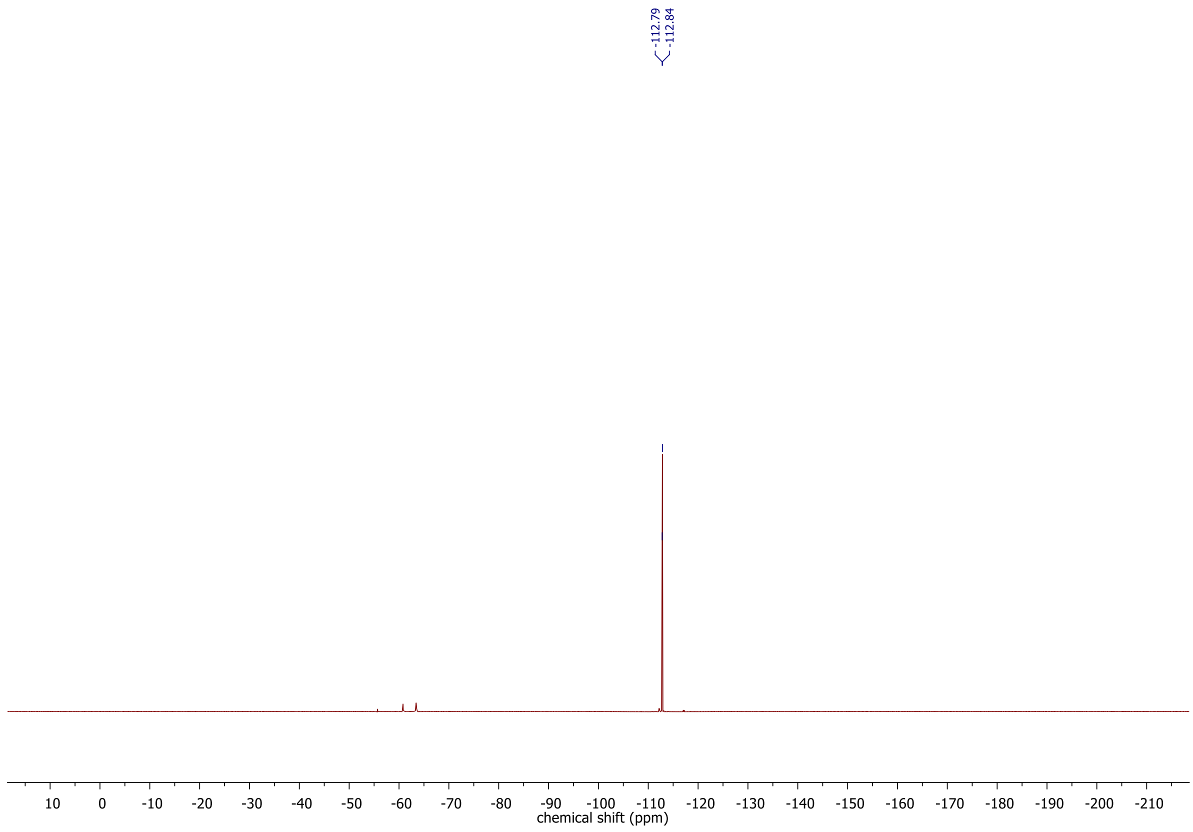
-112.79
-112.84
10 0 -10 -20 -30 -40 -50 -60 -70 -80 -90 -100 -110 -120 -130 -140 -150 -160 -170 -180 -190 -200 -210
chemical shift (ppm)

tert-Butyl (_S_)-(2-((1-((3,4-dimethoxyphenethyl)(methyl)amino)-4-methyl-1-oxopentan-2-yl)amino)-2-oxoethyl)carbamate (9o)

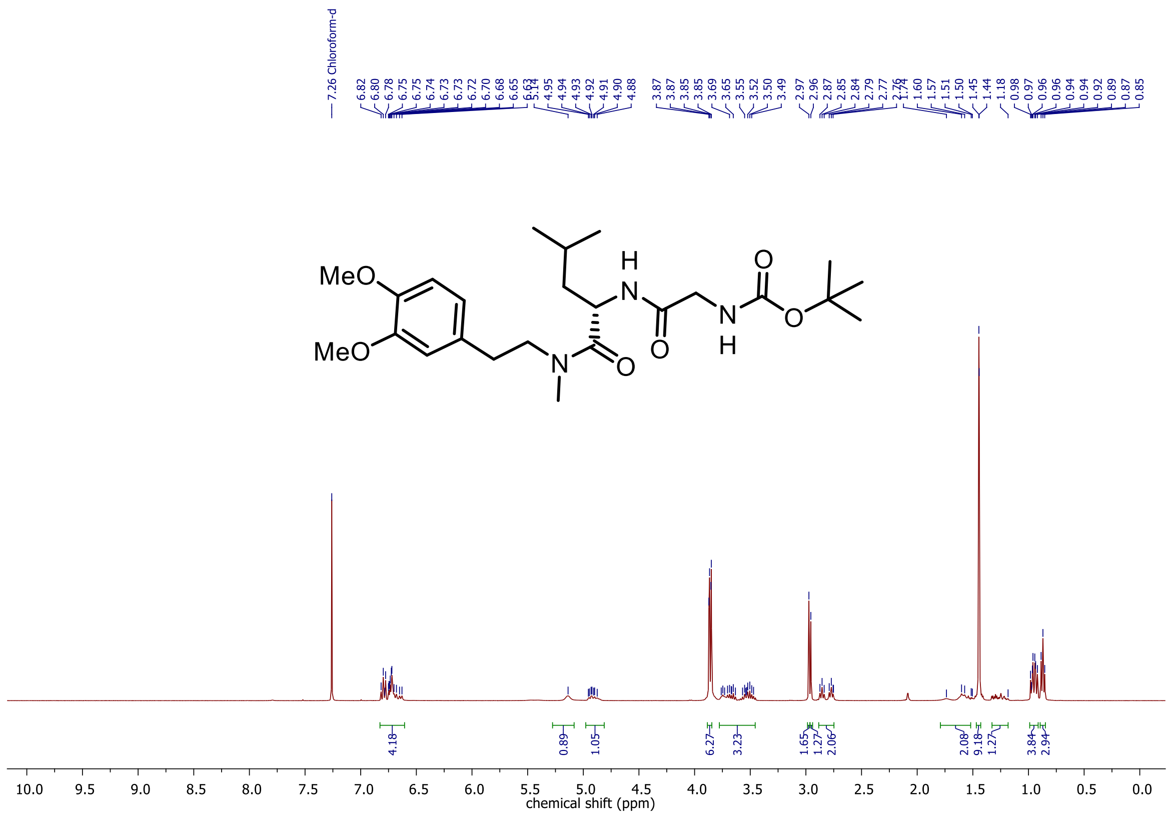

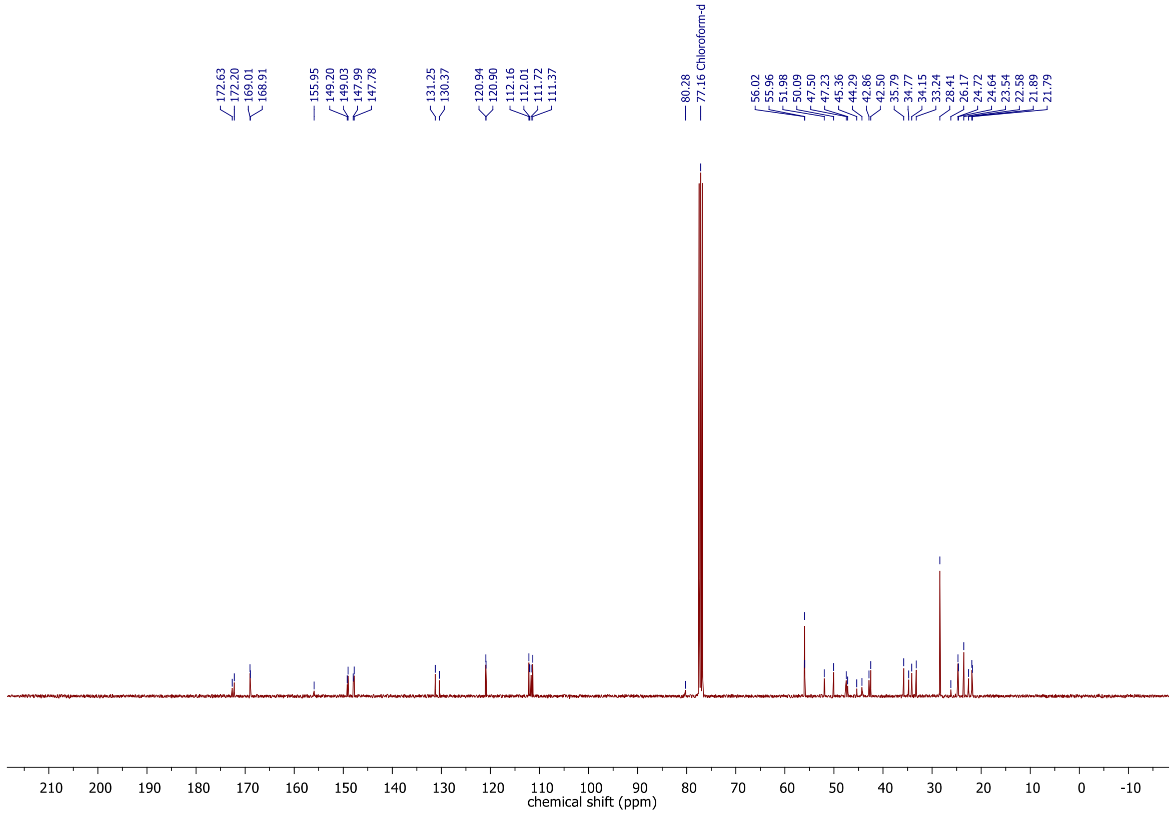

tert-Butyl ((_S_)-1-((_S_)-2-((3,4-dimethoxyphenethyl)(methyl)carbamoyl)pyrrolidin-1-yl)-3-methyl-1-oxobutan-2-yl)carbamate (9p)

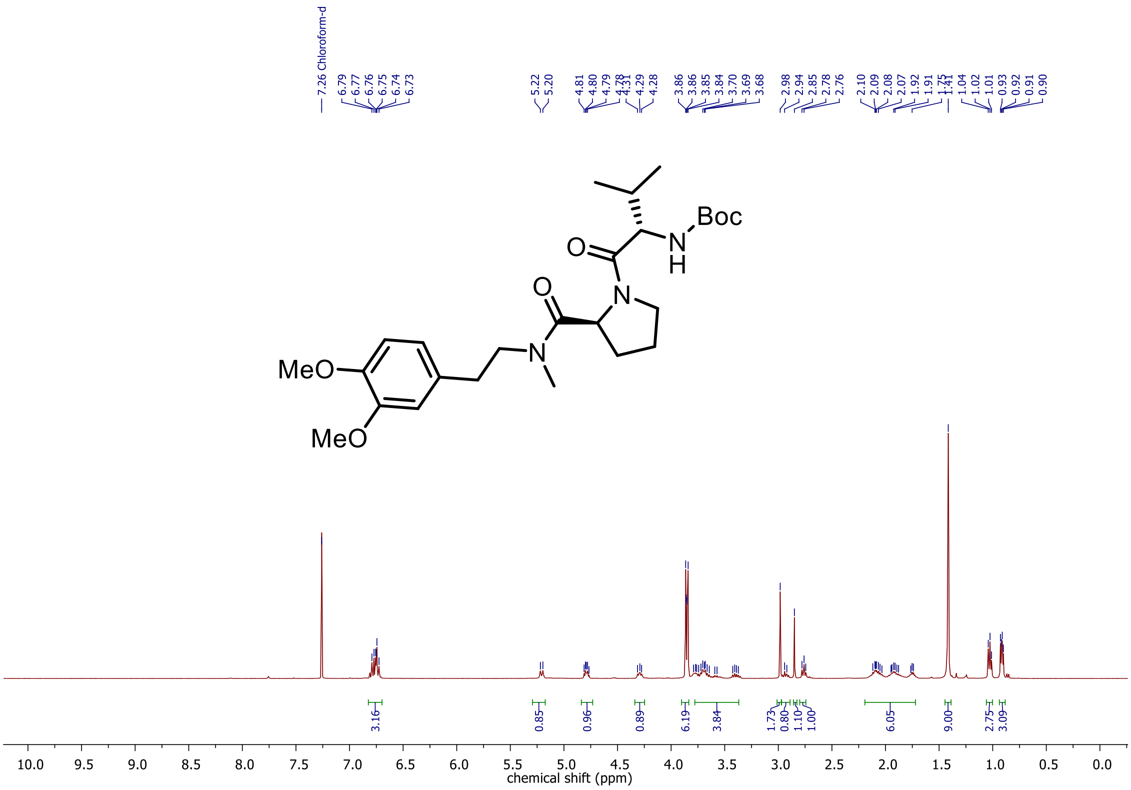

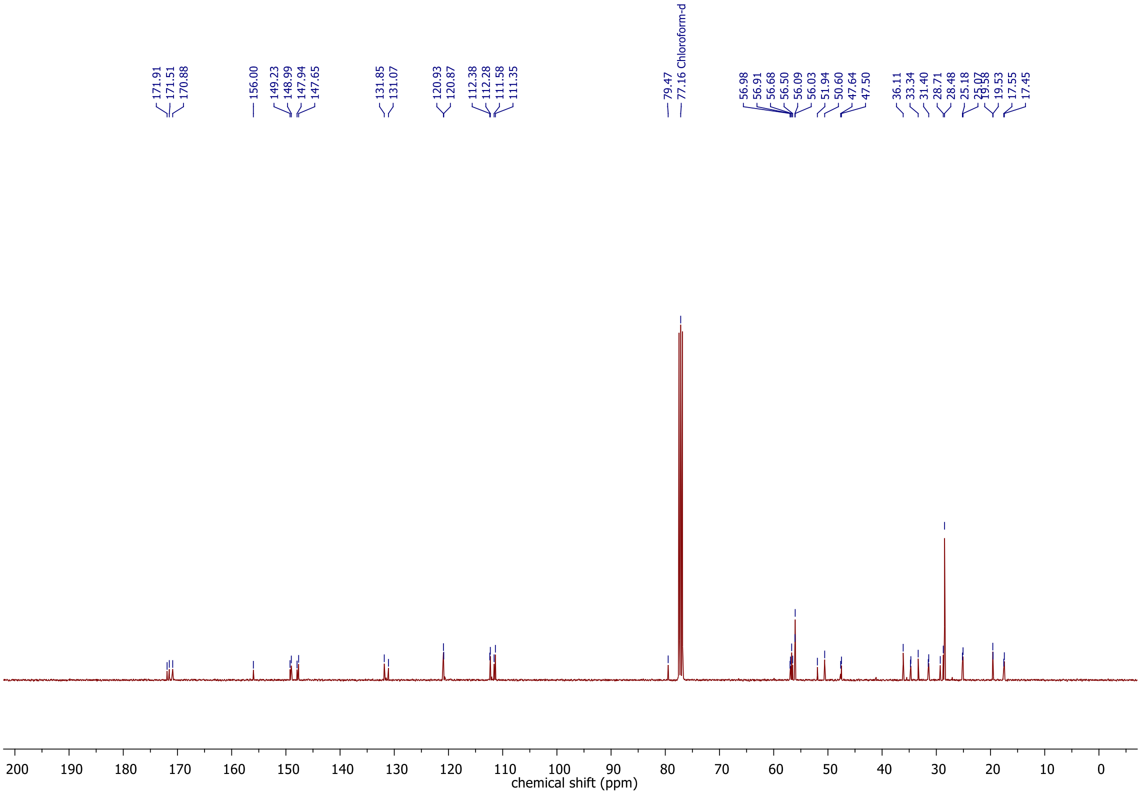

(4*R*)-*N*-(3,4-Dimethoxyphenethyl)-4-((8*R*,9*S*,10*S*,13*R*,14*S*,17*R*)-10,13-dimethyl-3,7,12-trioxohexadecahydro-1*H*-cyclopenta[*a*]phenanthren-17-yl)-*N*-methylpentanamide (9q)

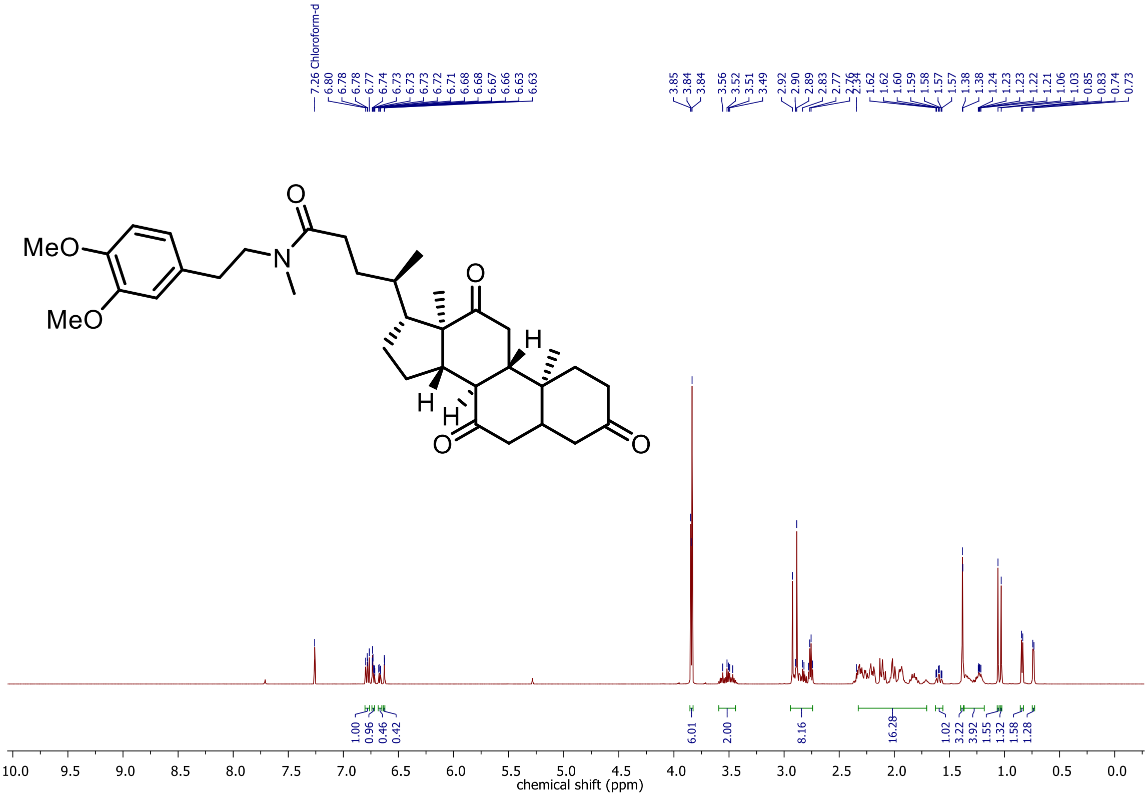

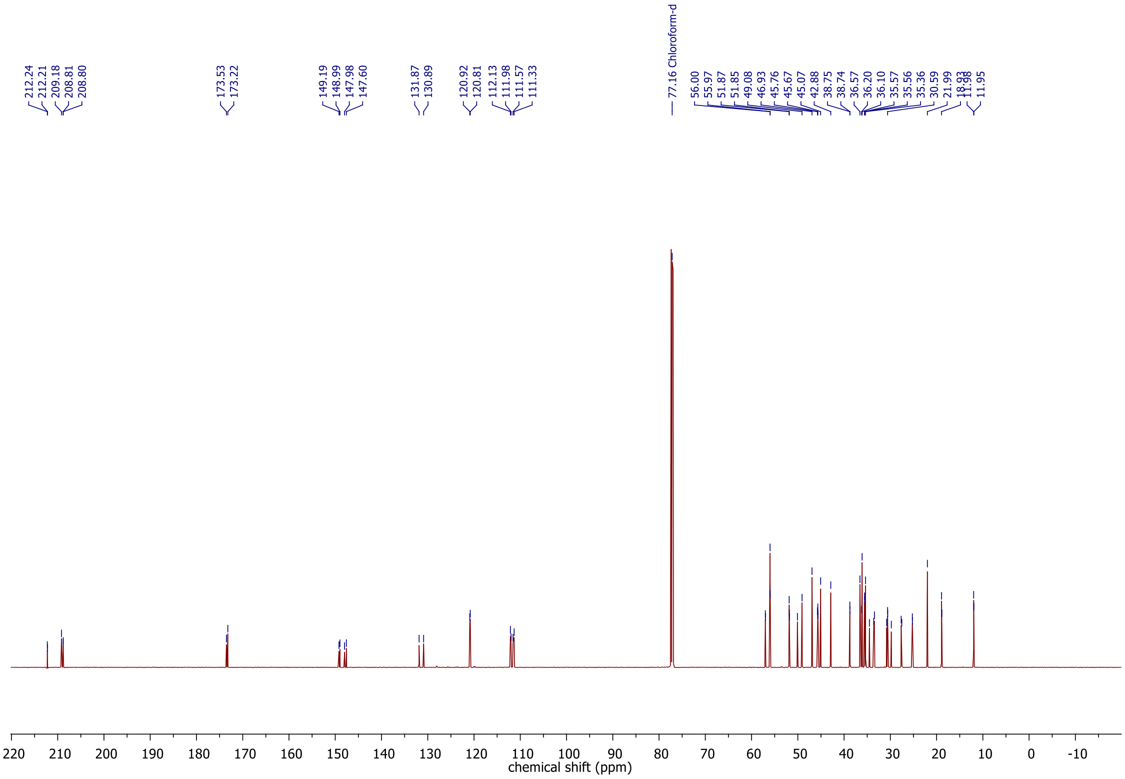

2-(1-(4-Chlorobenzoyl)-5-methoxy-2-methyl-1*H*-indol-3-yl)-*N*-(3,4-dimethoxyphenethyl)-*N*-methylacetamide (9r)

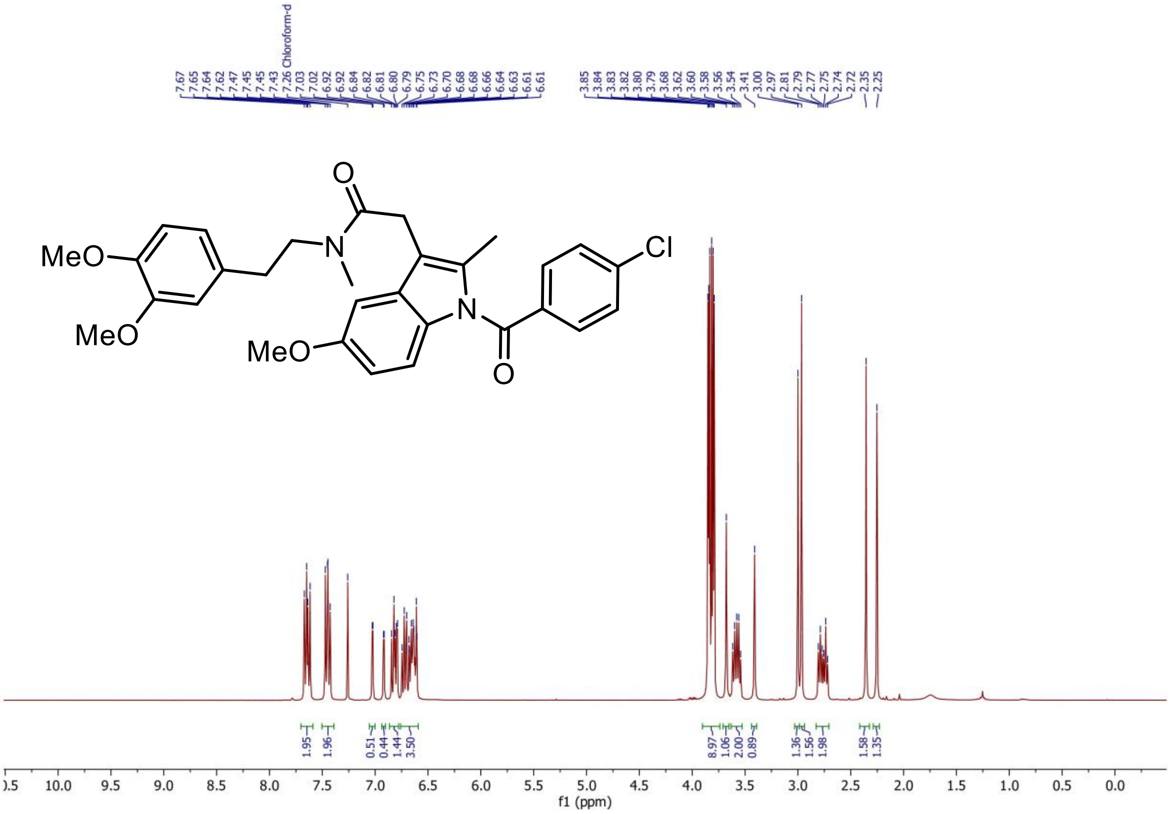

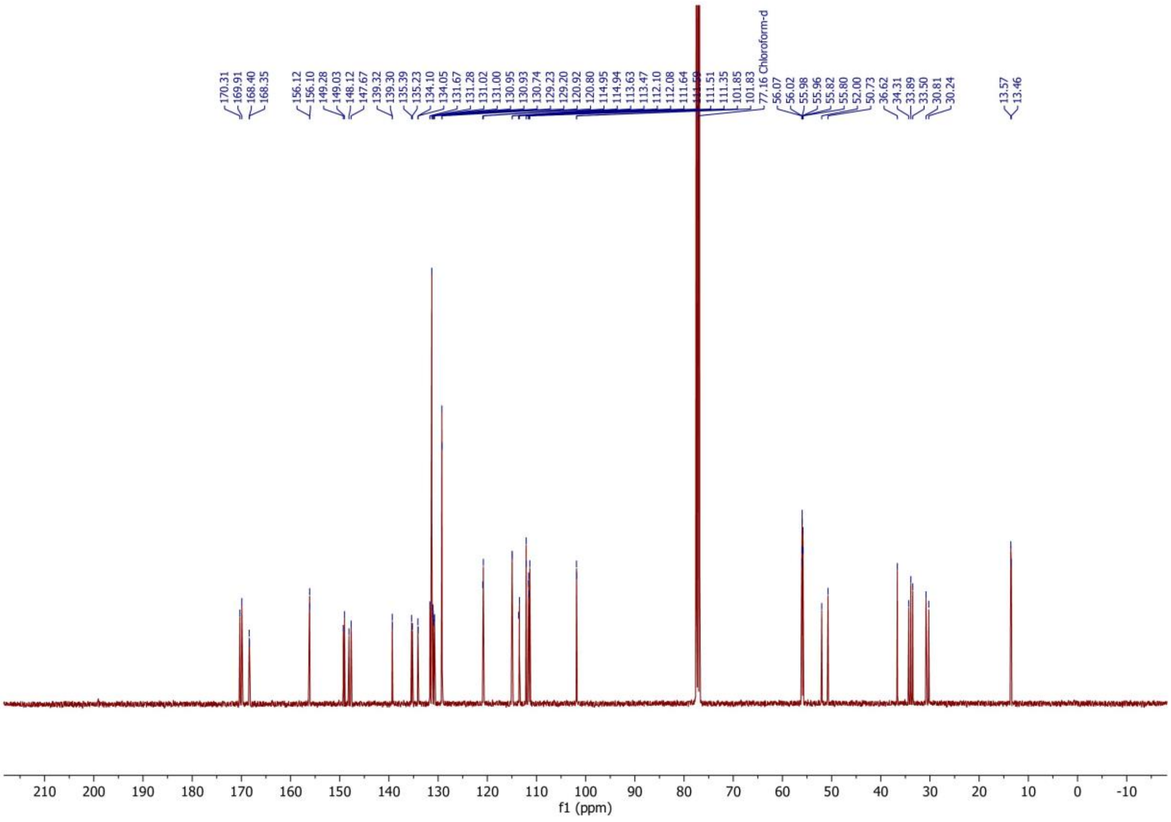

4-Chloro-*N*-phenethylbenzamide (12a)

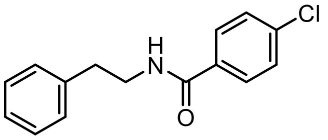

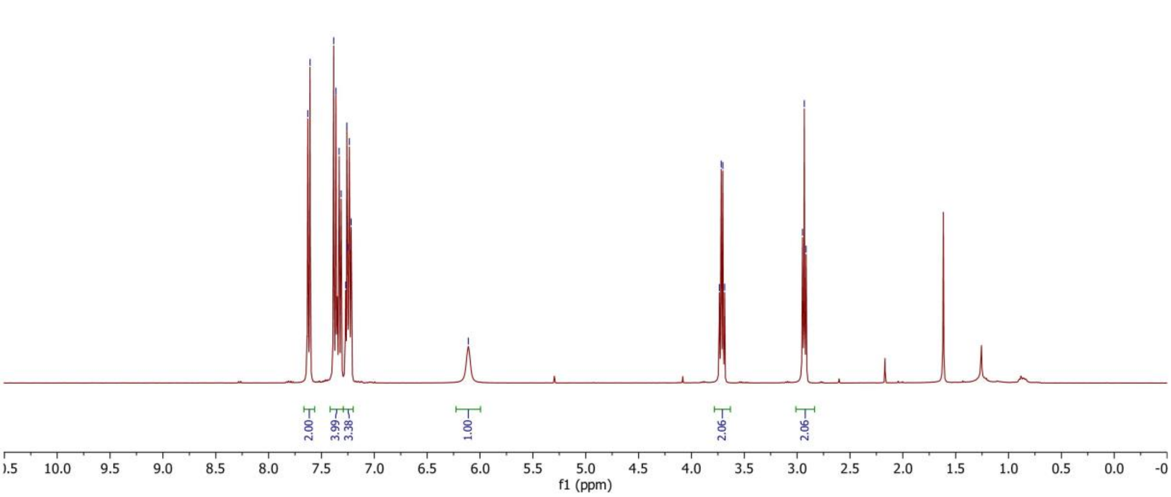

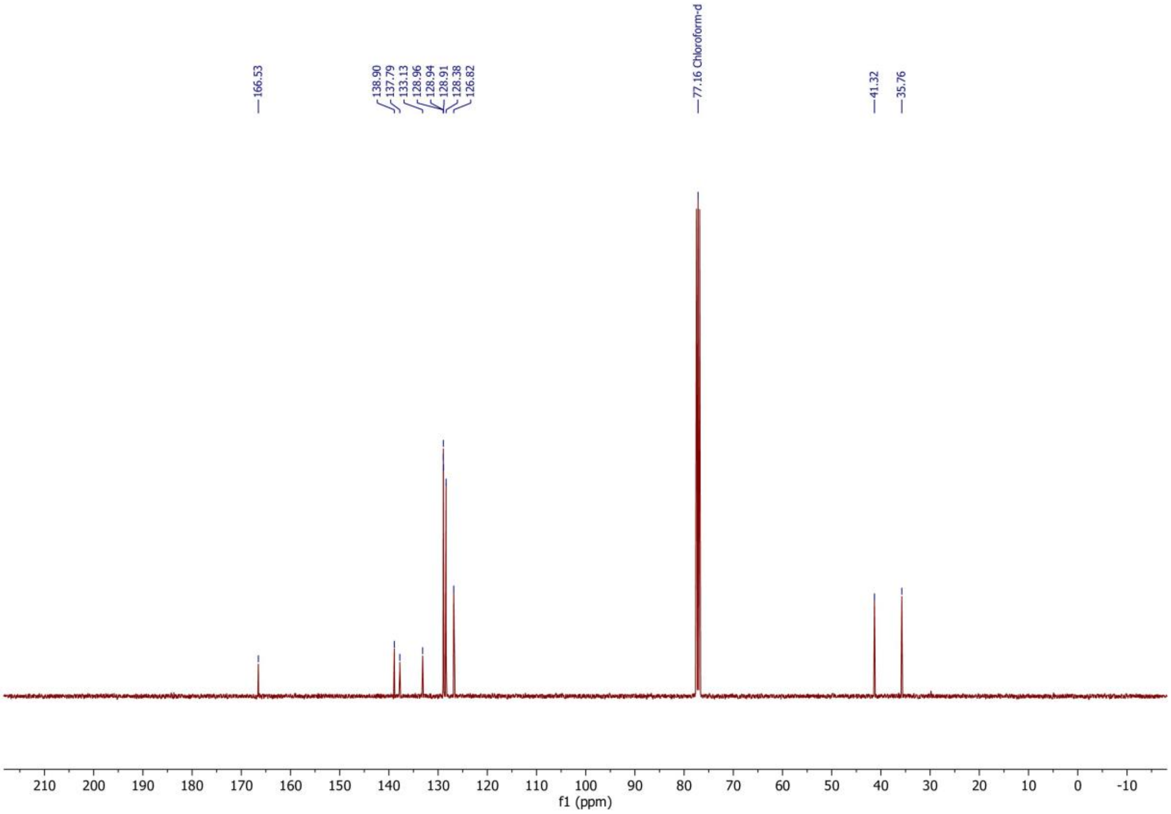

***N*-(2-(1*H*-Indol-3-yl)ethyl)-4-chlorobenzamide (12b)**

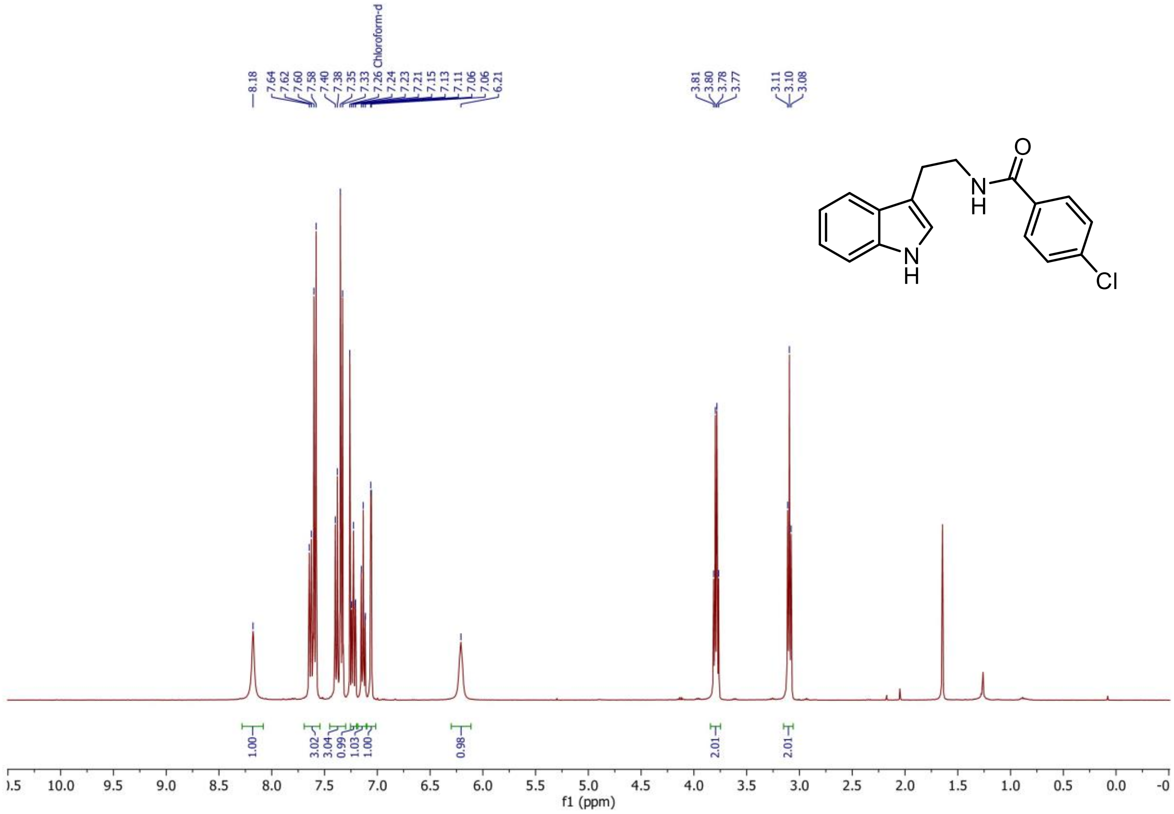

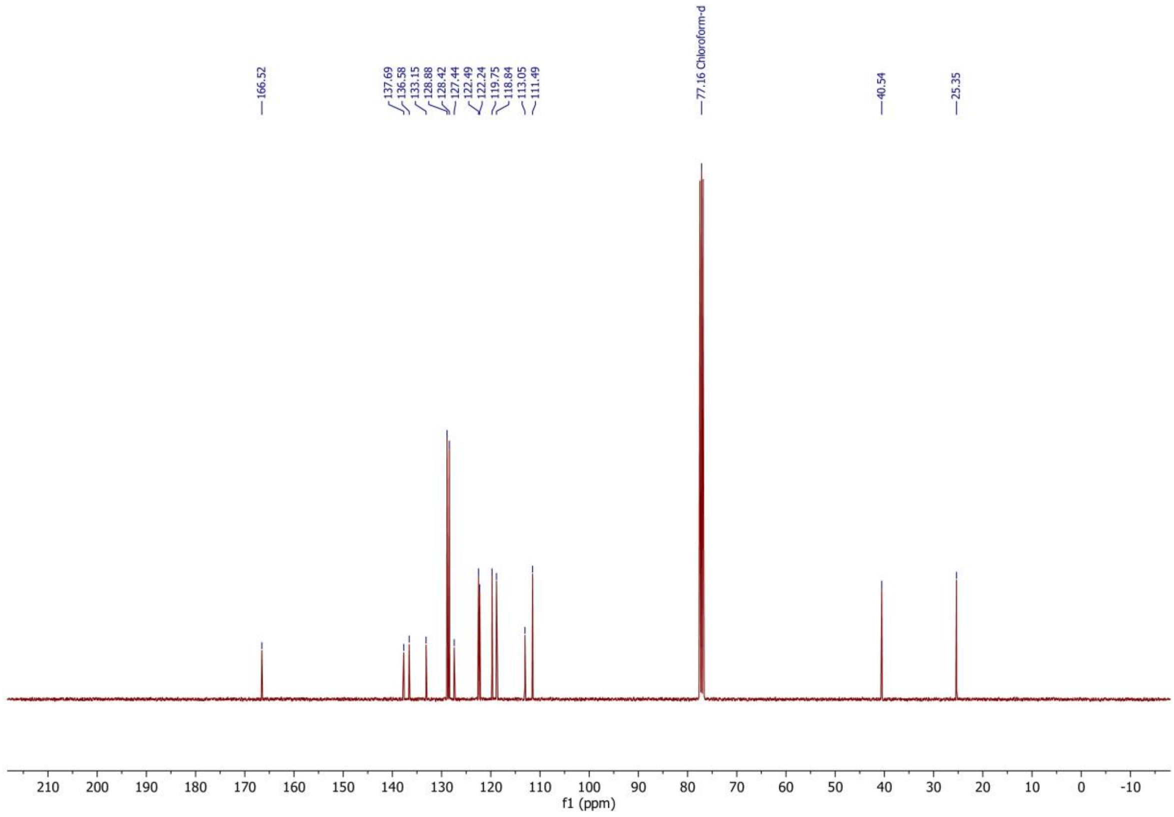

N-(tert-Butyl)-4-chlorobenzamide (12c)

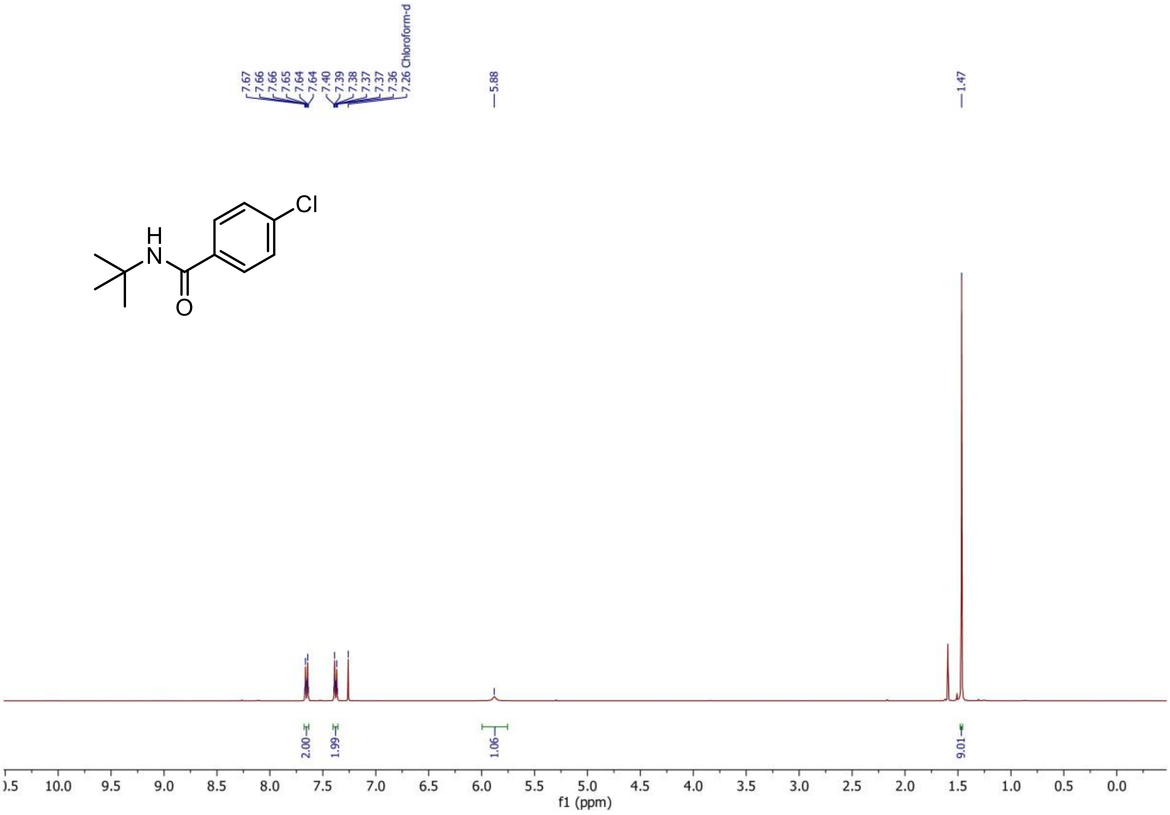

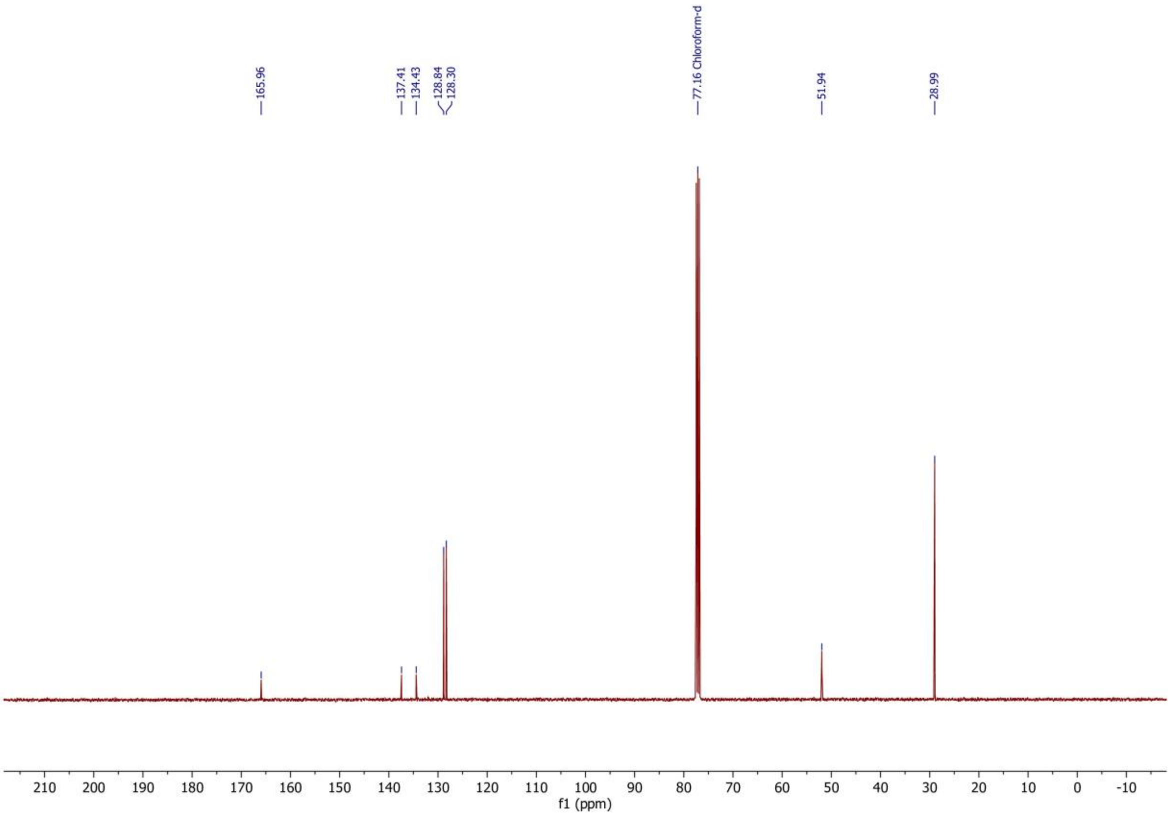

4-Chloro-*N*-(4-methoxybenzyl)benzamide (12d)

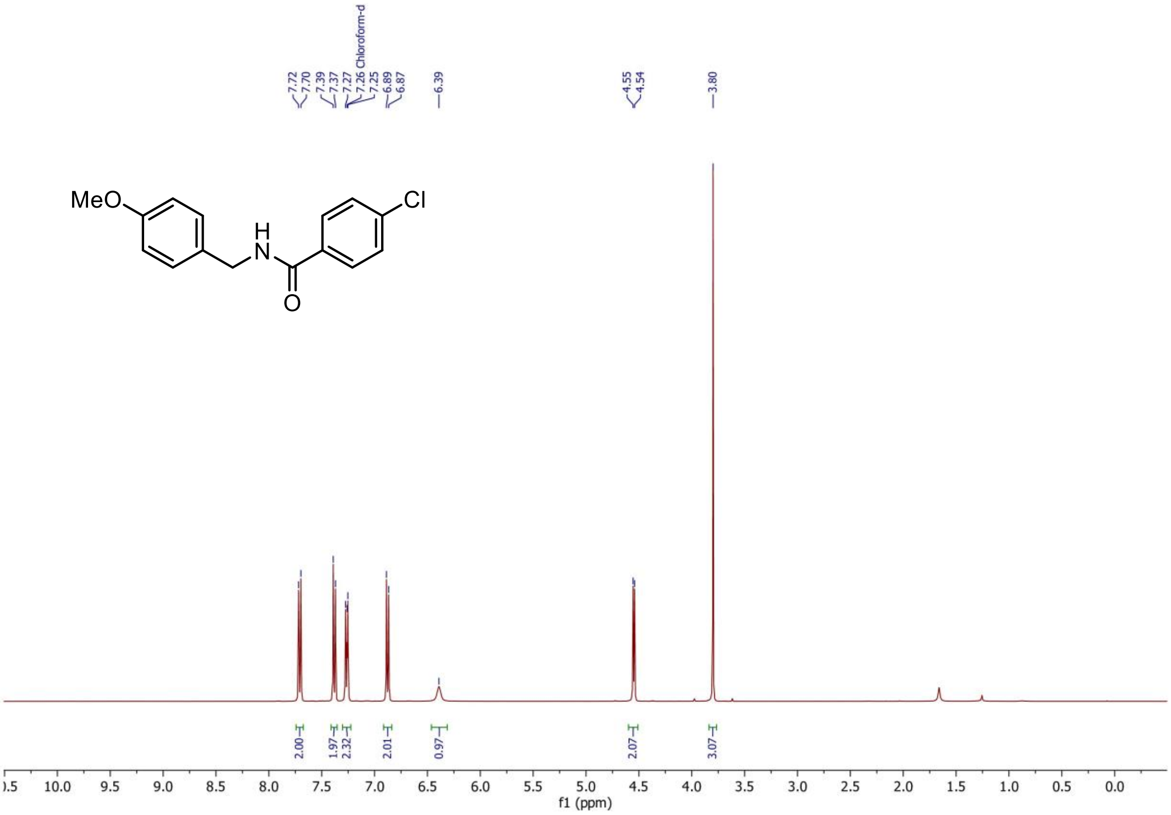

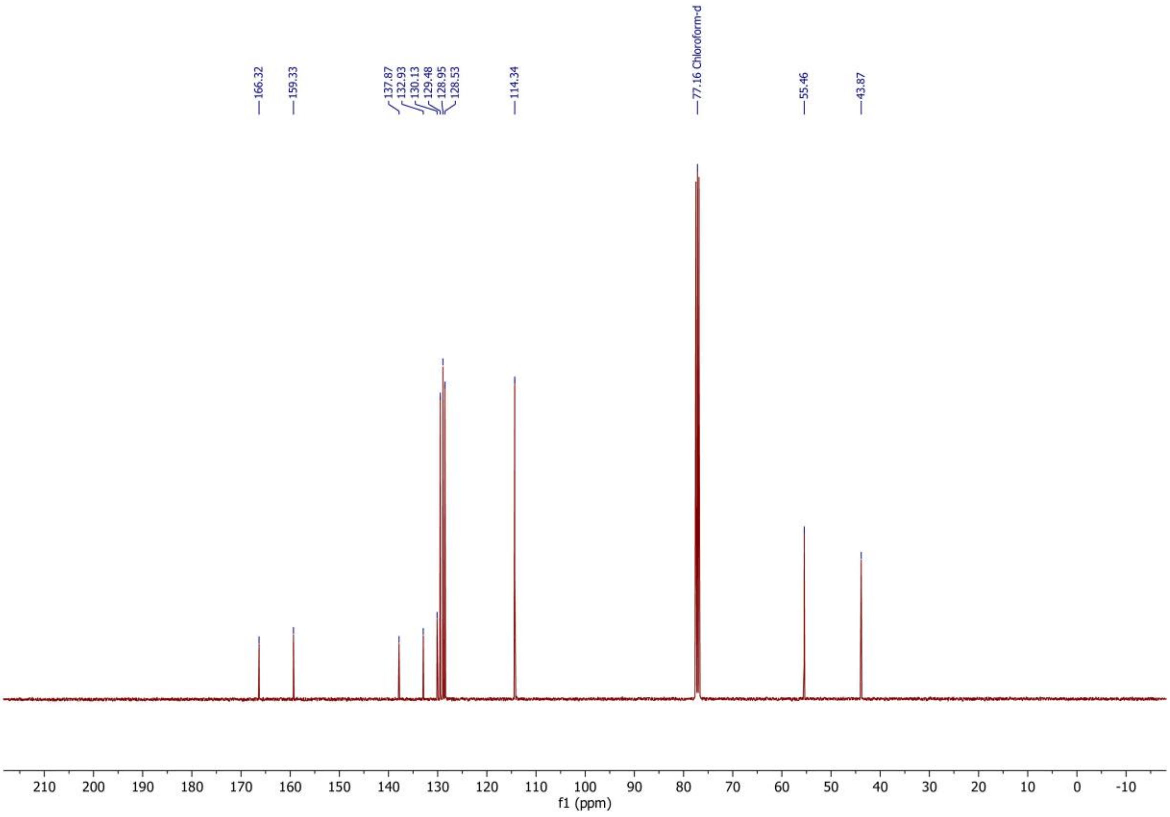

Methyl (4-chlorobenzoyl)-*L*-isoleucinate (12e)

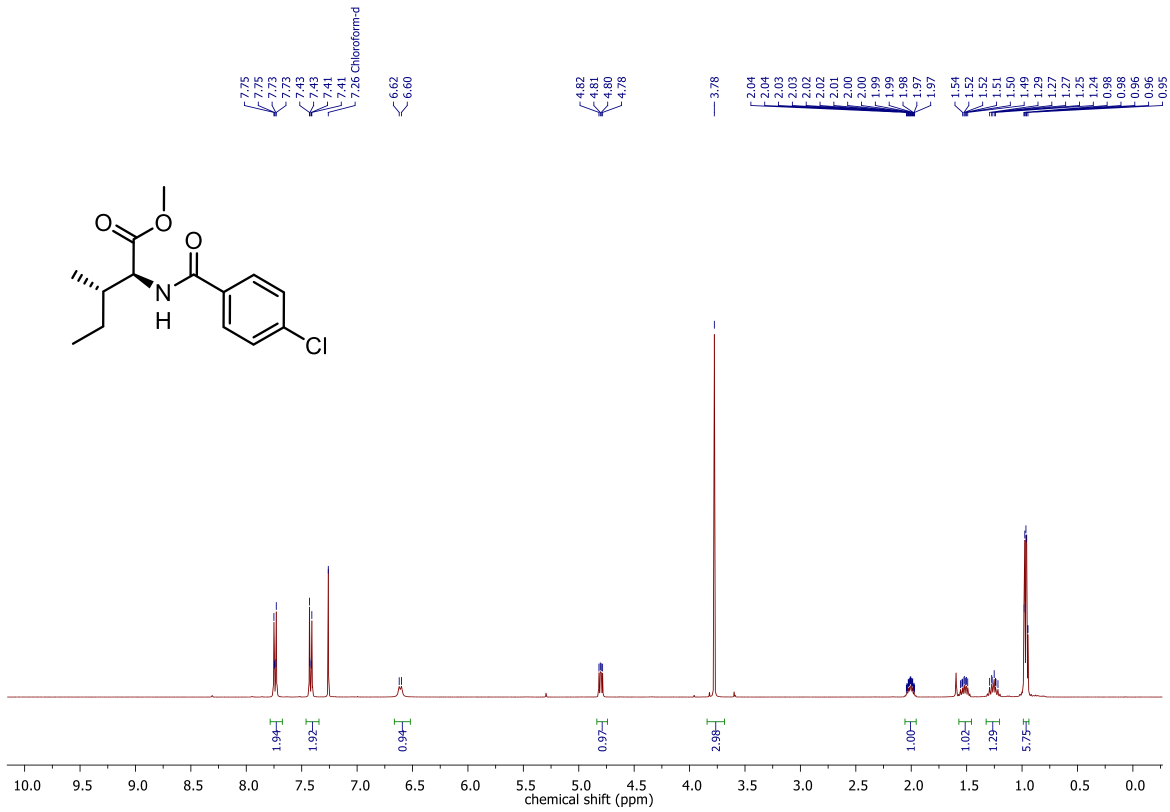

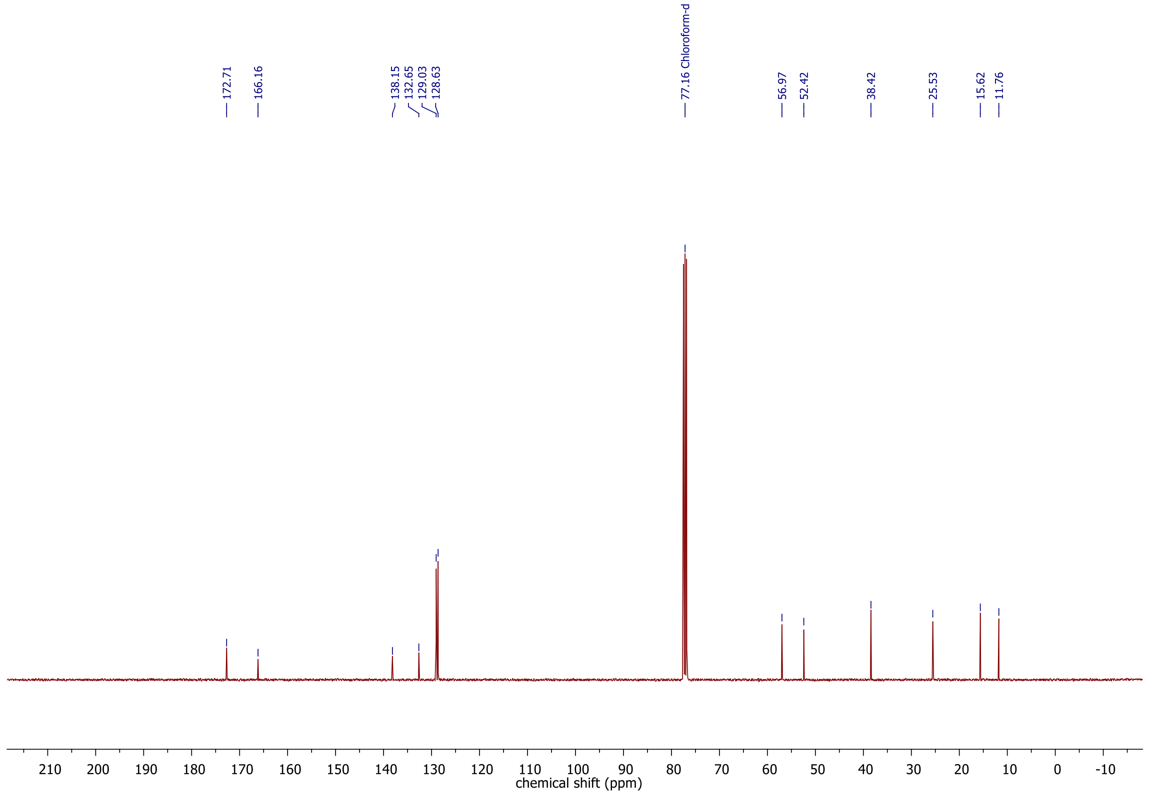

(4-Chlorophenyl)(4-(4,4,5,5-tetramethyl-1,3,2-dioxaborolan-2-yl)piperidin-1-yl)methanone (12f)

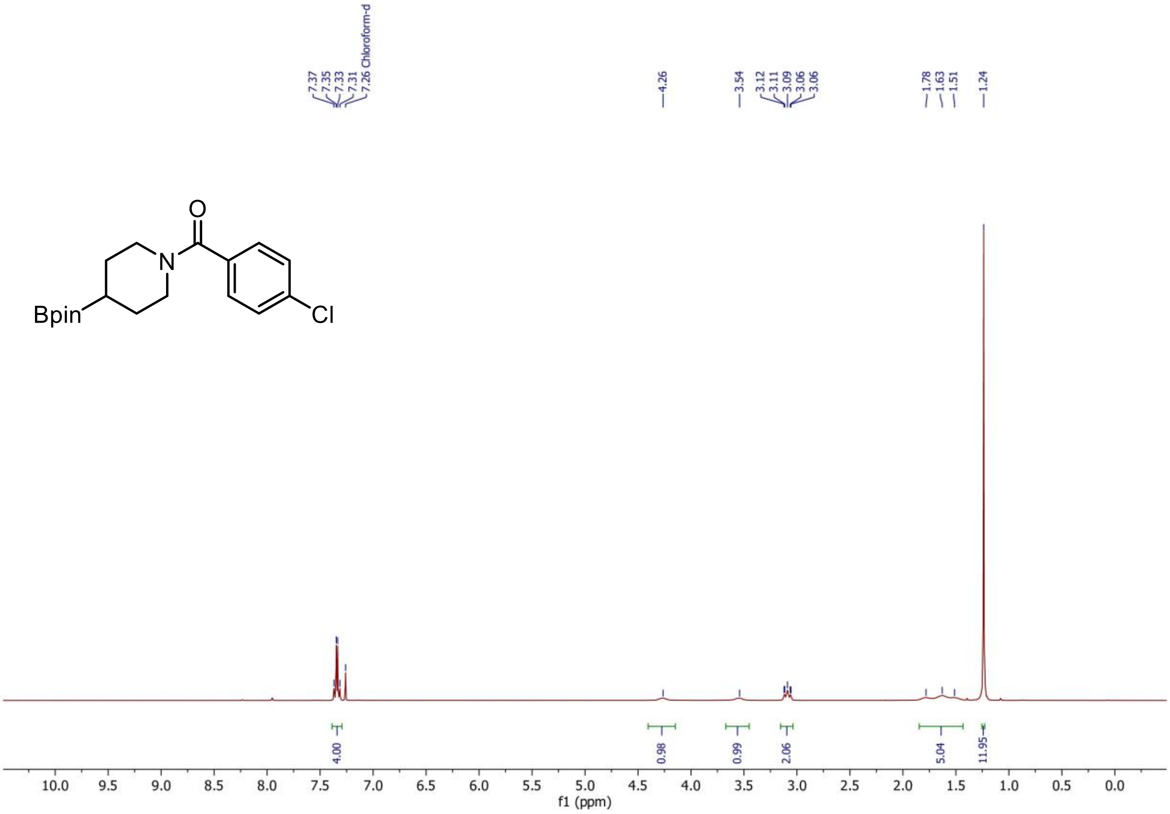

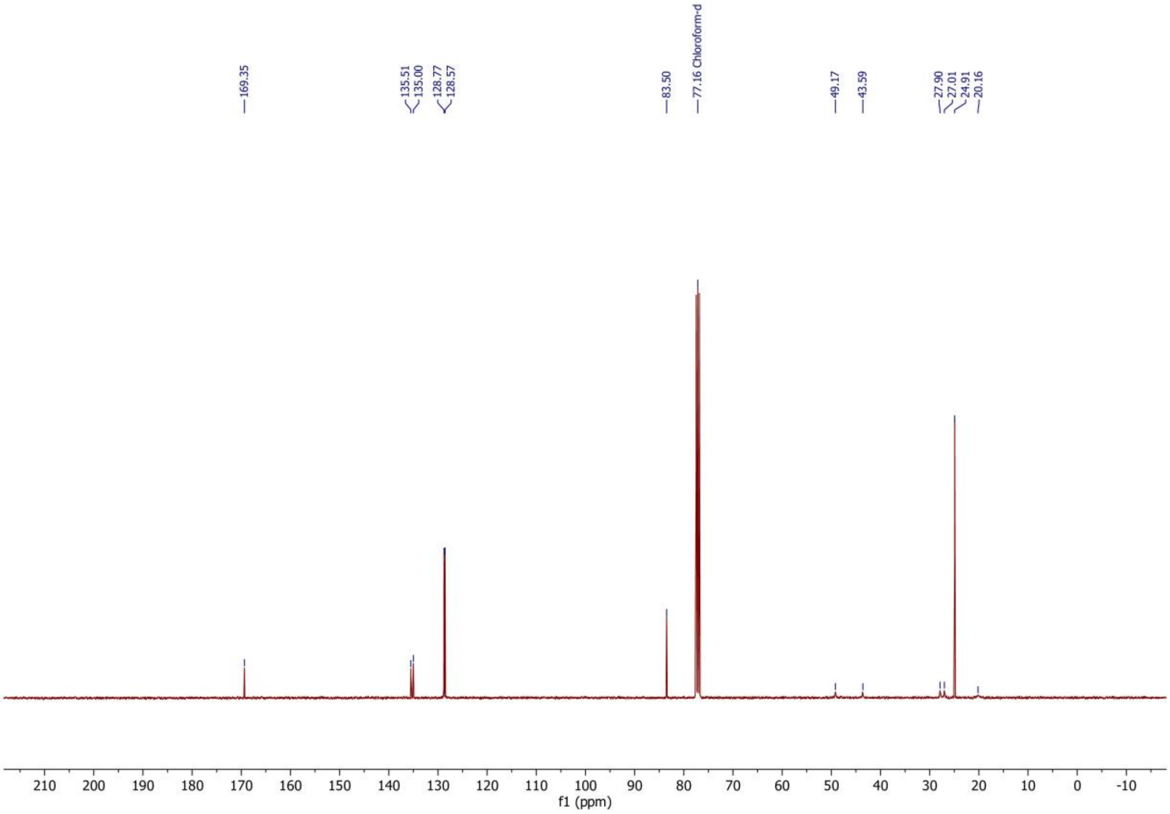

8-(4-Chlorobenzoyl)-8-azabicyclo[3.2.1]octan-3-one (12g)

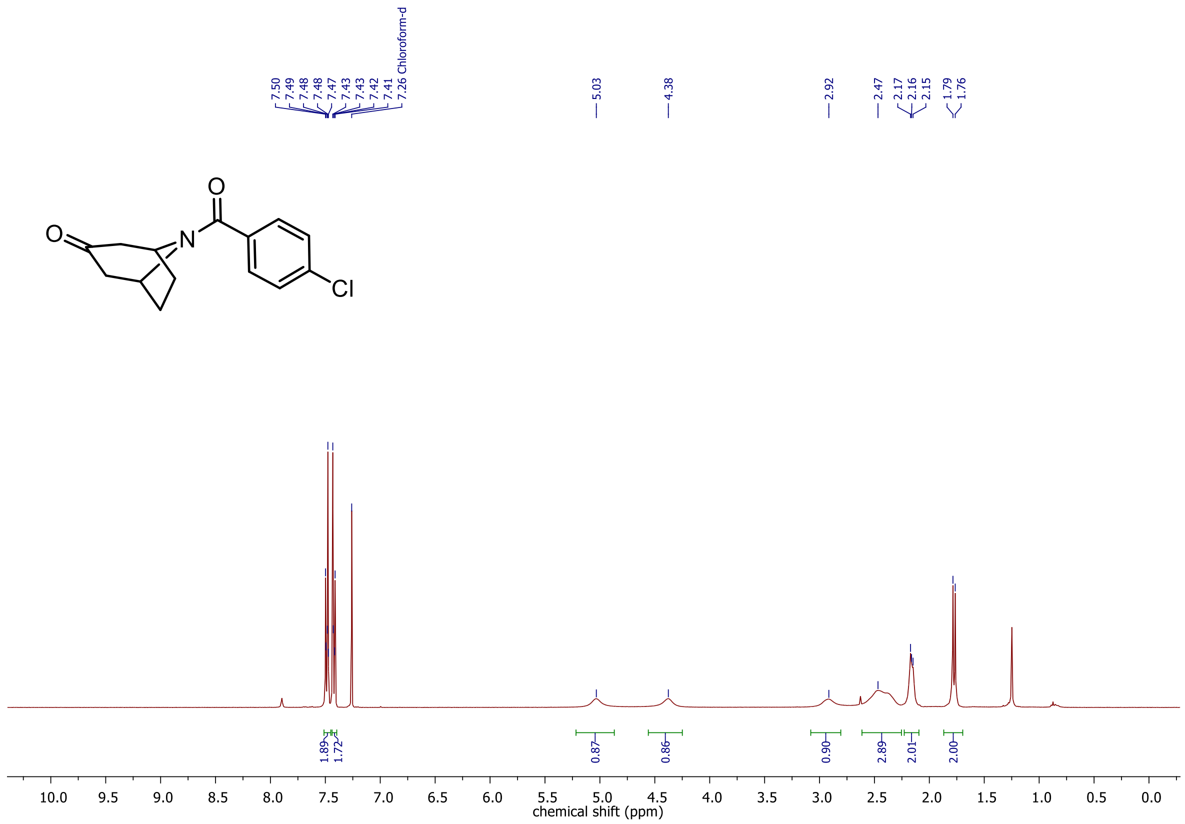

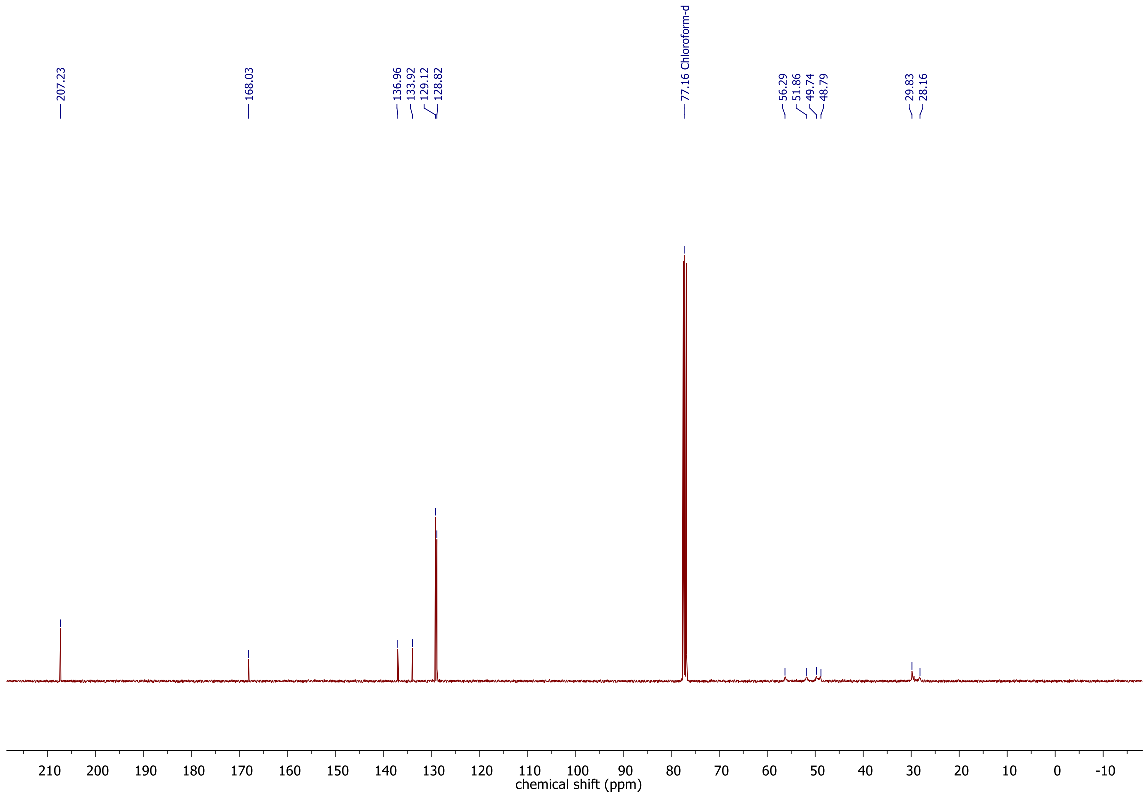

HSQC

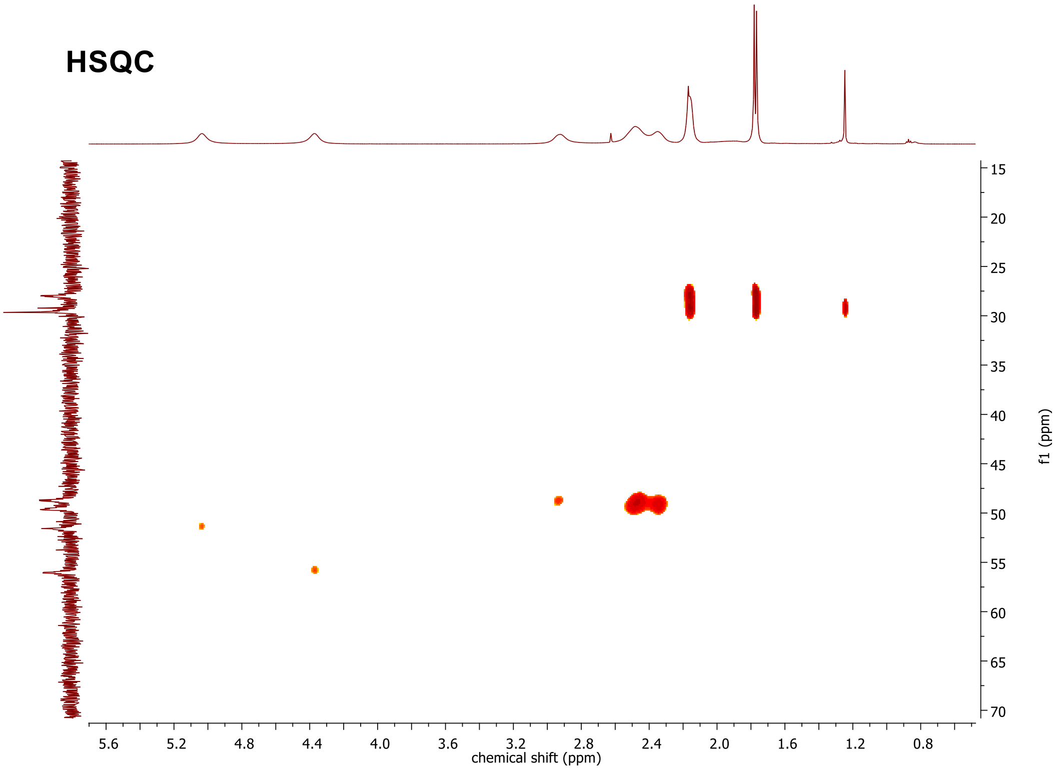

5.6
5.2
4.8
4.4
4.0
3.6
3.2
2.8
2.4
2.0
1.6
1.2
0.8
chemical shift (ppm)
15
20
25
30
35
40
45
50
55
60
65
70
f1 (ppm)

((3*S*,4*R*)-3-((Benzo[*d*][1,3]dioxol-5-yloxy)methyl)-4-(4-fluorophenyl)piperidin-1-yl)(4-chlorophenyl)methanone (12h)

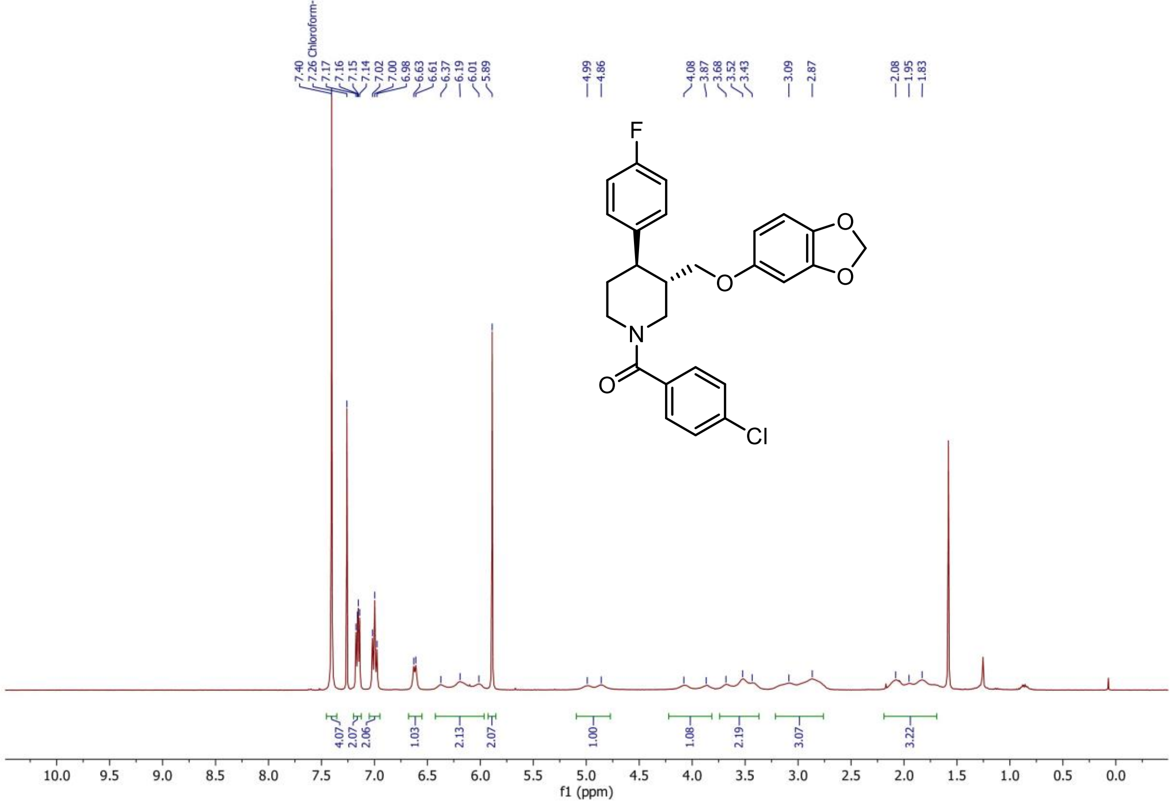

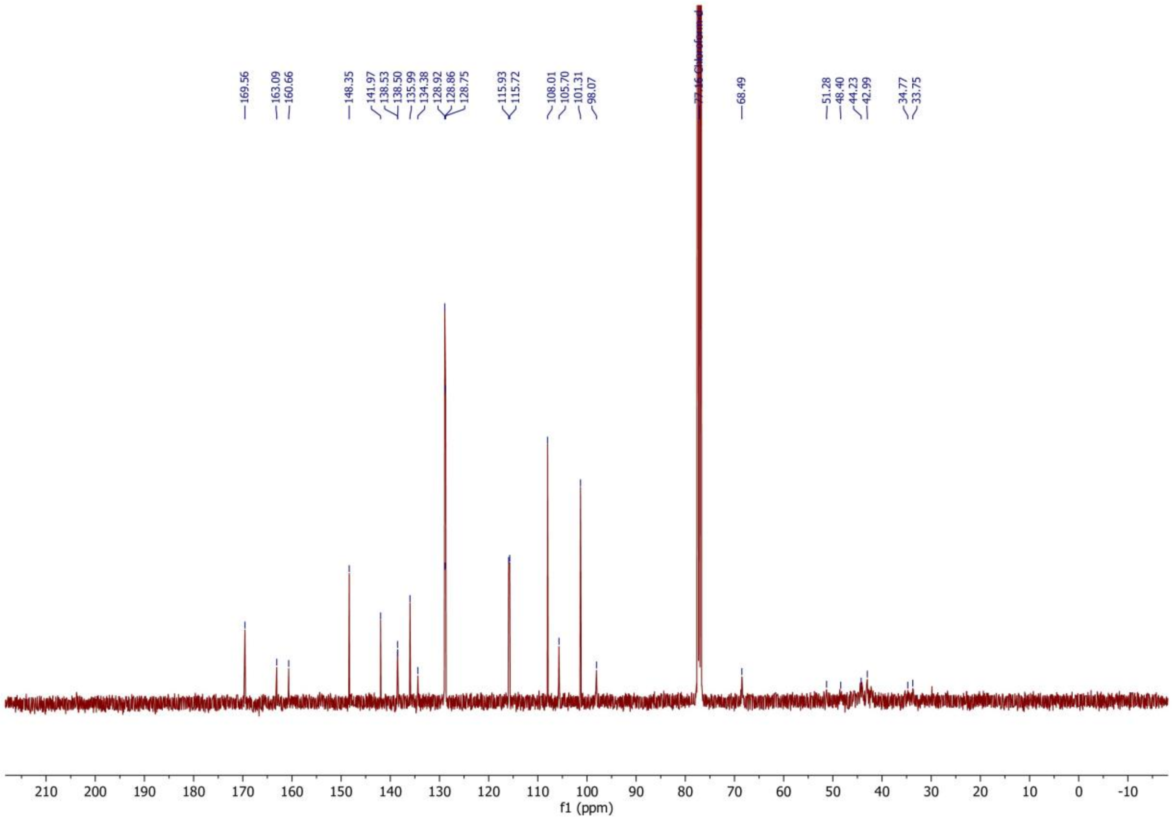

HSQC

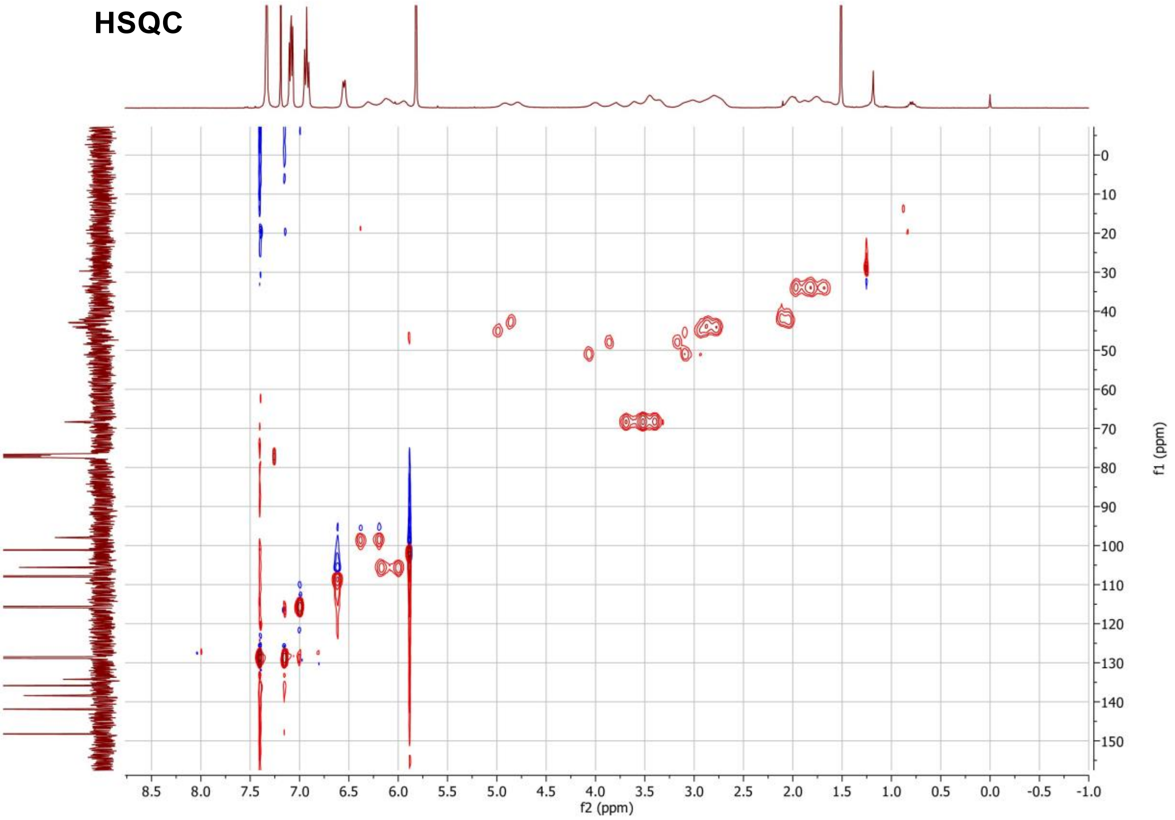

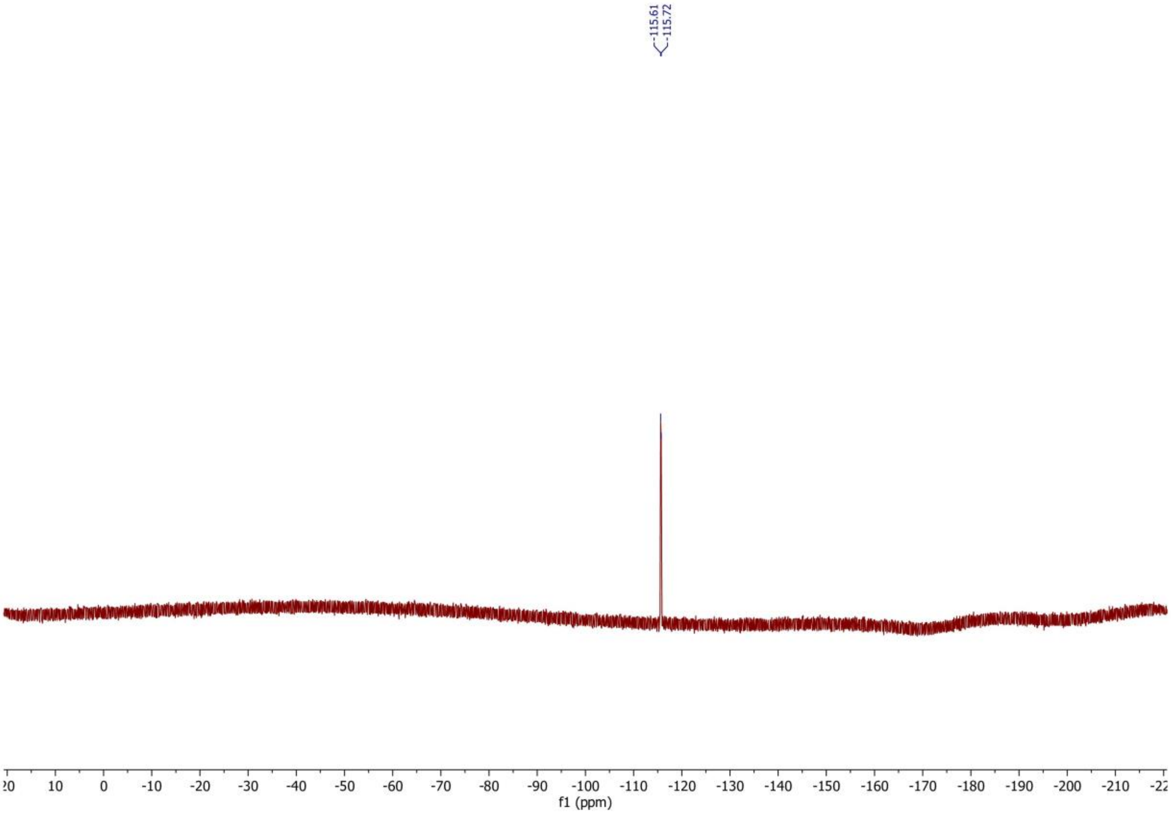

(4-(8-Chloro-5,6-dihydro-11*H*-benzo[5,6]cyclohepta[1,2-*b*]pyridin-11-ylidene)piperidin-1-yl)(4-chlorophenyl)methanone (12i)

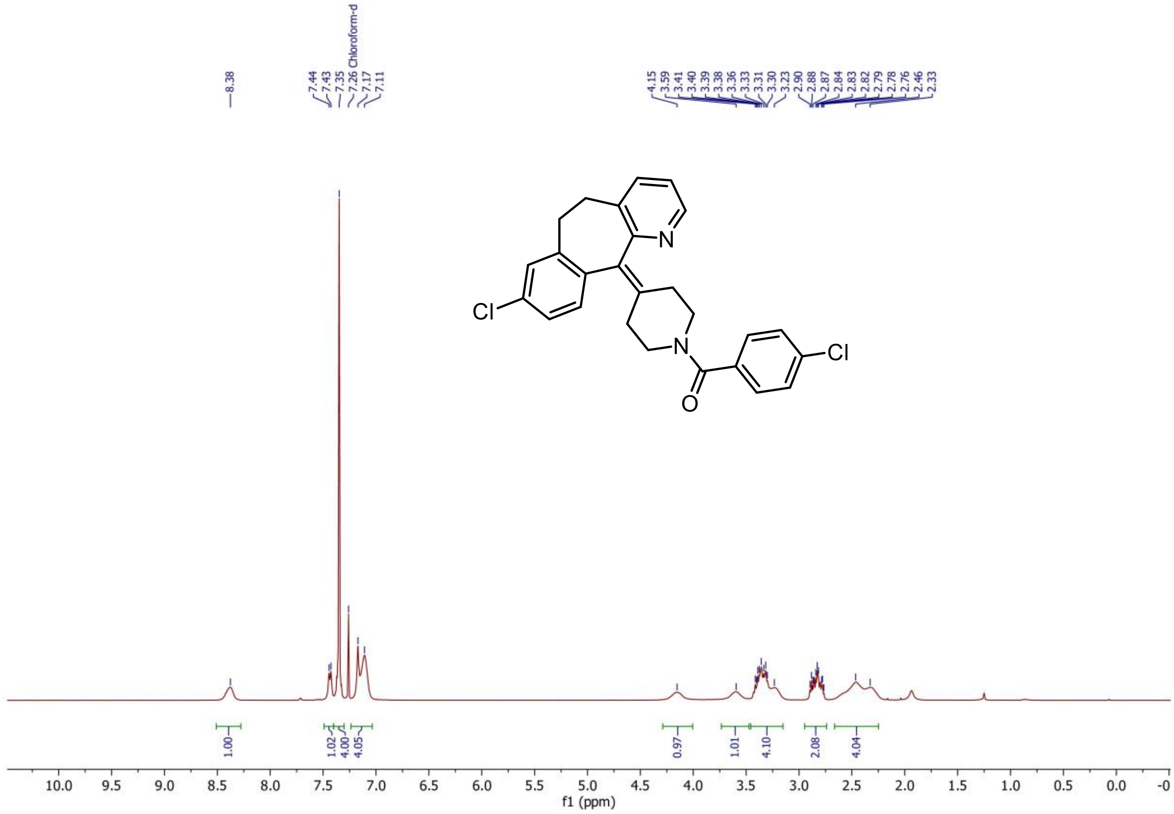

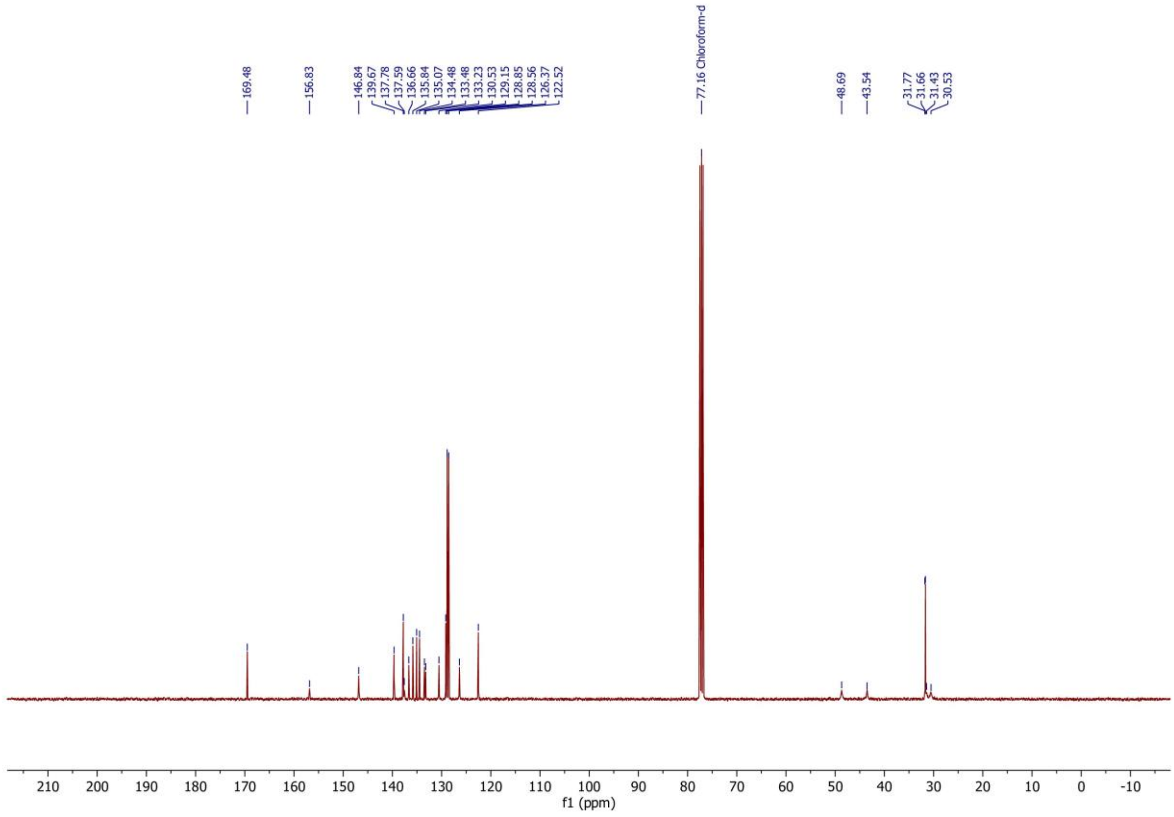

(*S*)-4-Chloro-*N*-methyl-*N*-(3-(naphthalen-1-yloxy)-3-(thiophen-2-yl)propyl)benzamide (12j)

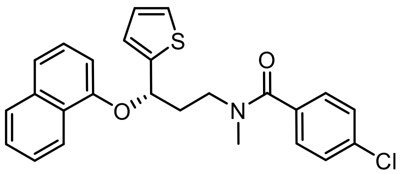

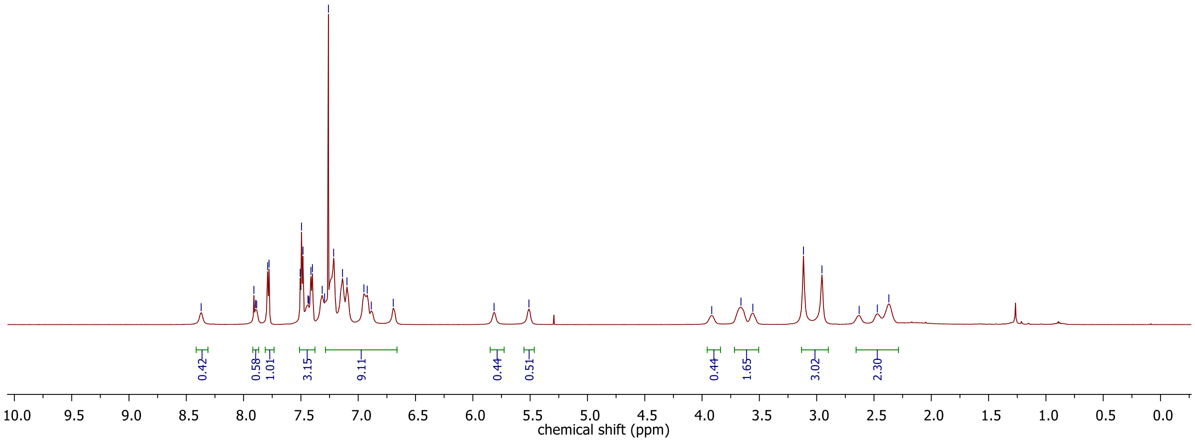

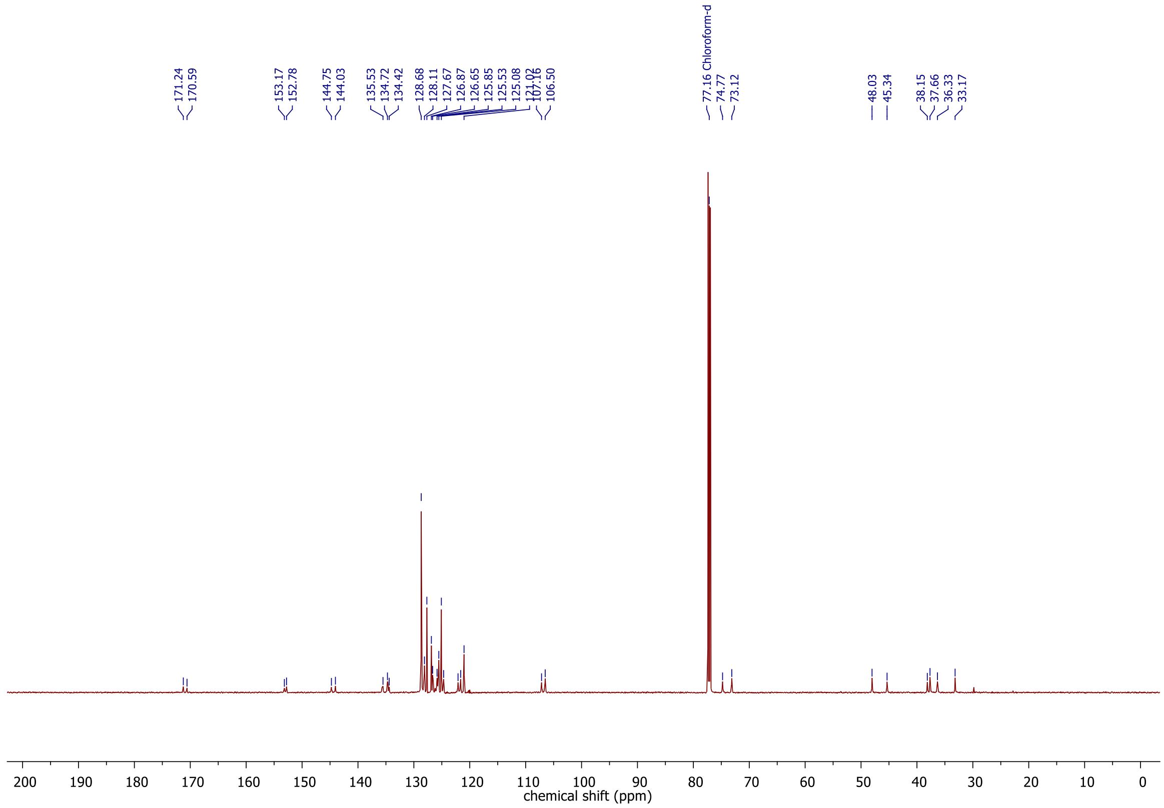

S8

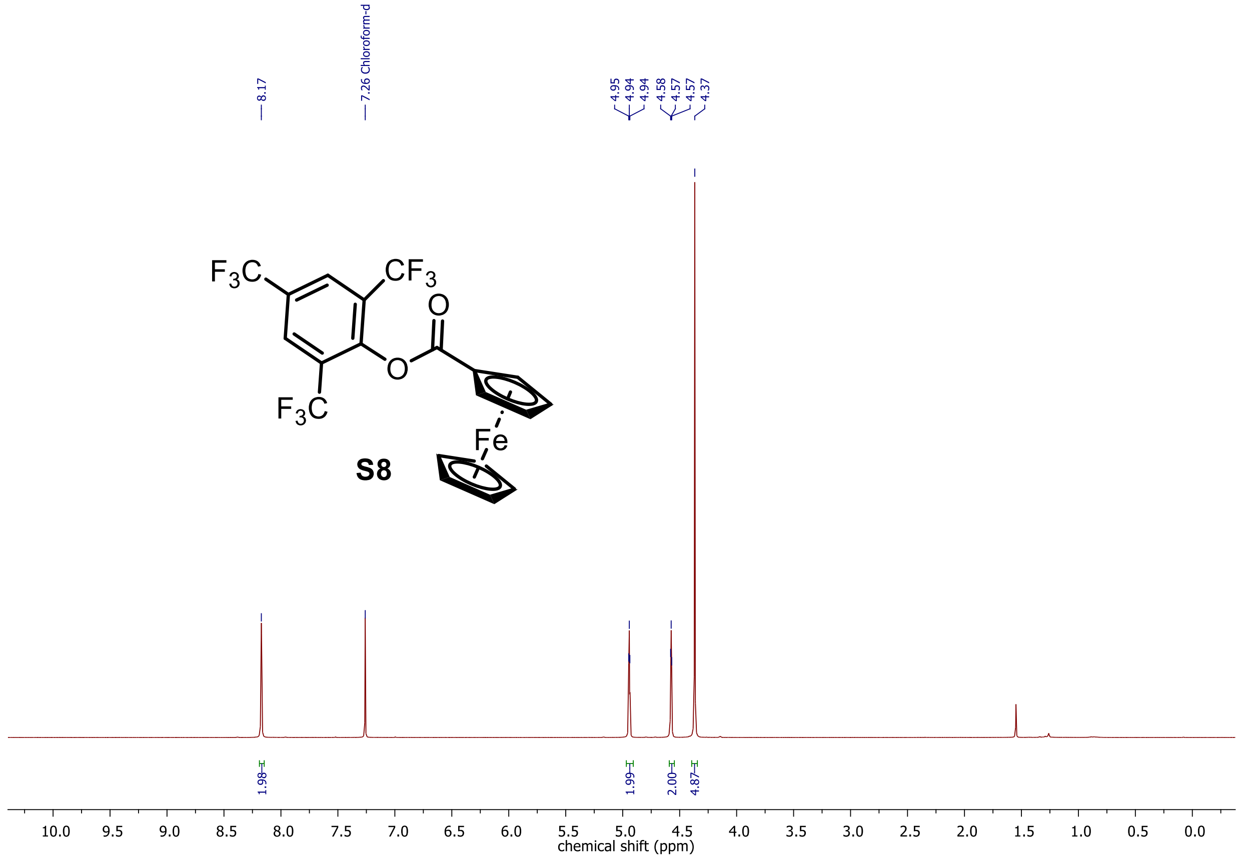
8.17
7.26 Chloroform-d
4.95
4.94
4.94
4.58
4.57
4.57
4.37
F_3C
CF_3
O
O
F_3C
Fe
S8
1.98
1.99
2.00
4.87
10.0 9.5 9.0 8.5 8.0 7.5 7.0 6.5 6.0 5.5 5.0 4.5 4.0 3.5 3.0 2.5 2.0 1.5 1.0 0.5 0.0
chemical shift (ppm)

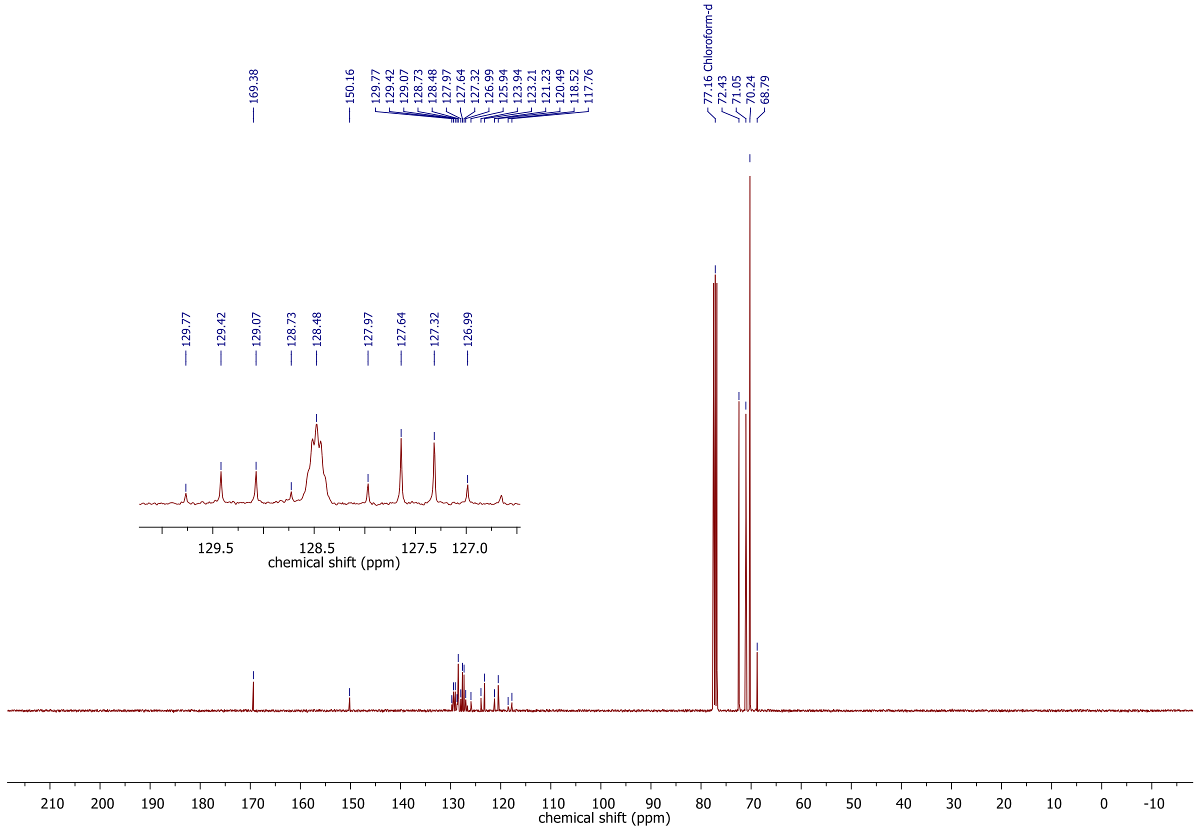
169.38
150.16
129.77
129.42
129.07
128.73
128.48
127.97
127.64
127.32
126.99
125.94
123.94
123.21
121.23
120.49
118.52
117.76
77.16 Chloroform-d
72.43
71.05
70.24
68.79
129.77
129.42
129.07
128.73
128.48
127.97
127.64
127.32
126.99
129.5 128.5 127.5 127.0
chemical shift (ppm)
210 200 190 180 170 160 150 140 130 120 110 100 90 80 70 60 50 40 30 20 10 0 -10
chemical shift (ppm)

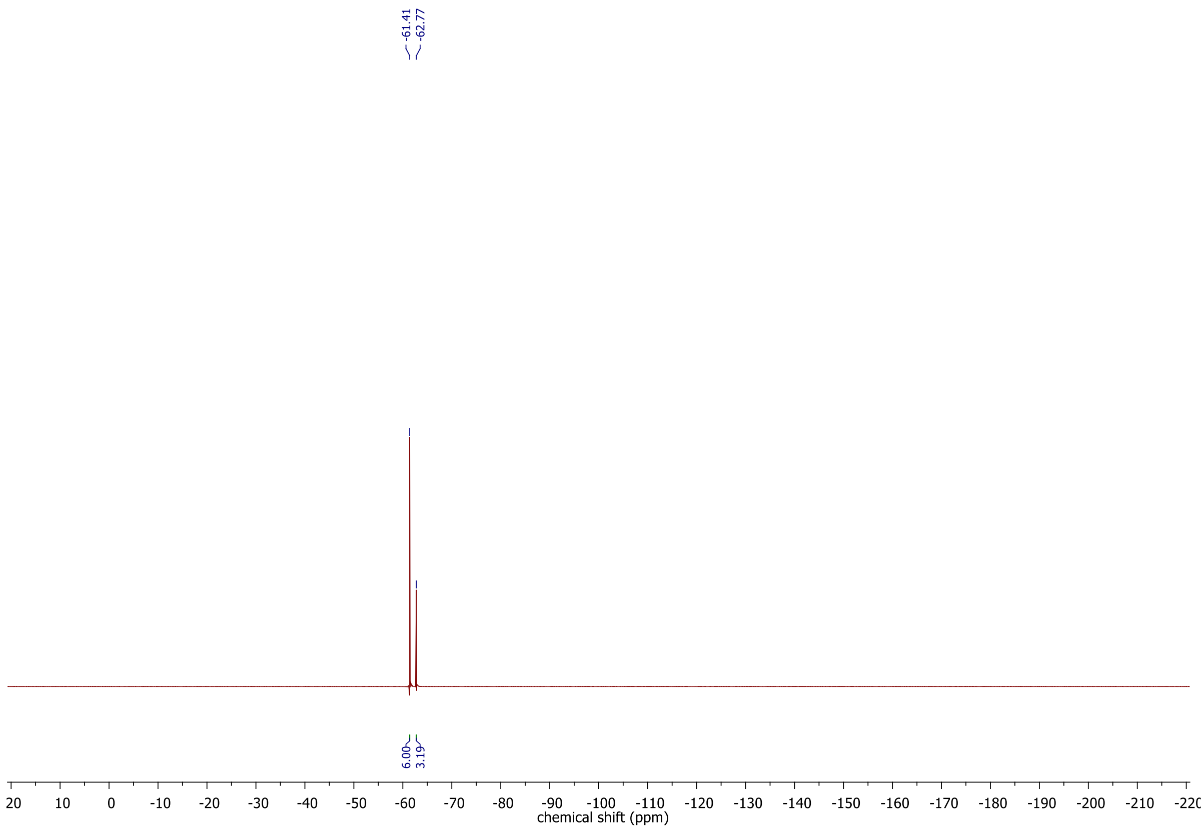

-61.41
-62.77
6.00
3.19
20 10 0 -10 -20 -30 -40 -50 -60 -70 -80 -90 -100 -110 -120 -130 -140 -150 -160 -170 -180 -190 -200 -210 -220
chemical shift (ppm)

1-((2,4,6-Tris(trifluoromethyl)phenyl)sulfonyl)piperazine (S9)

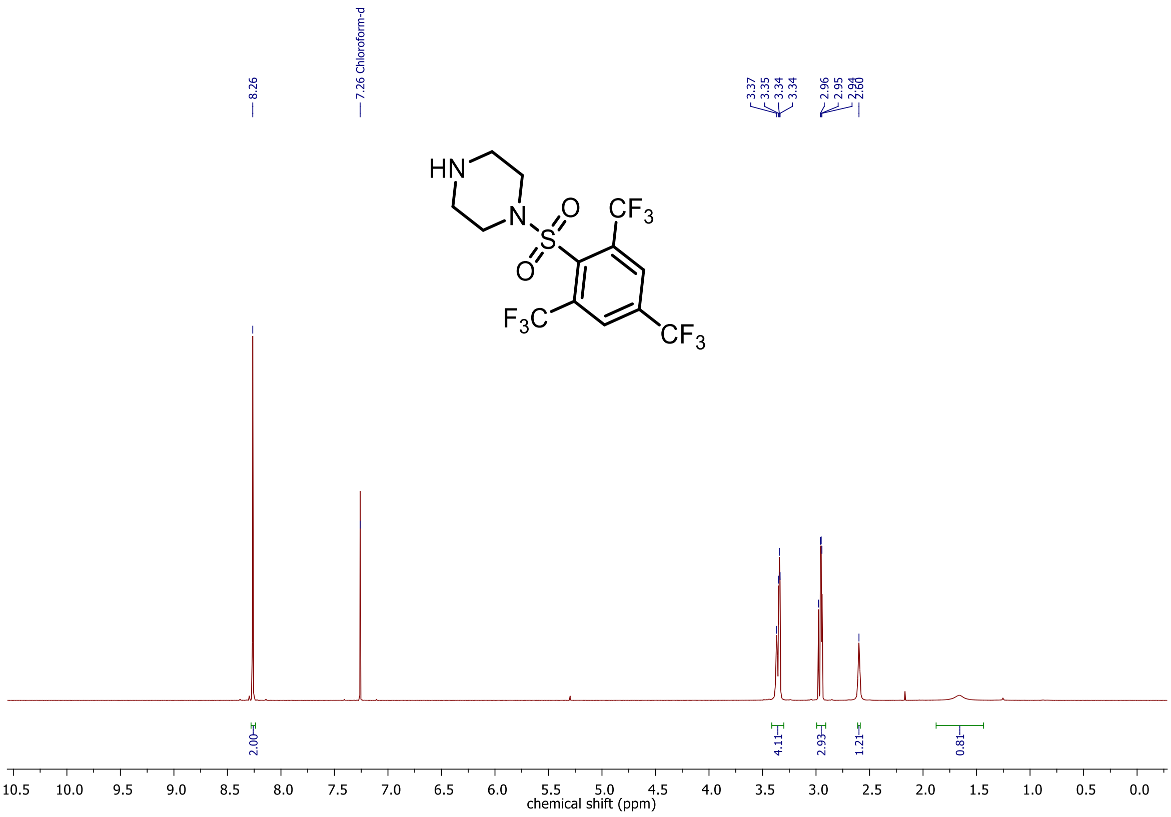

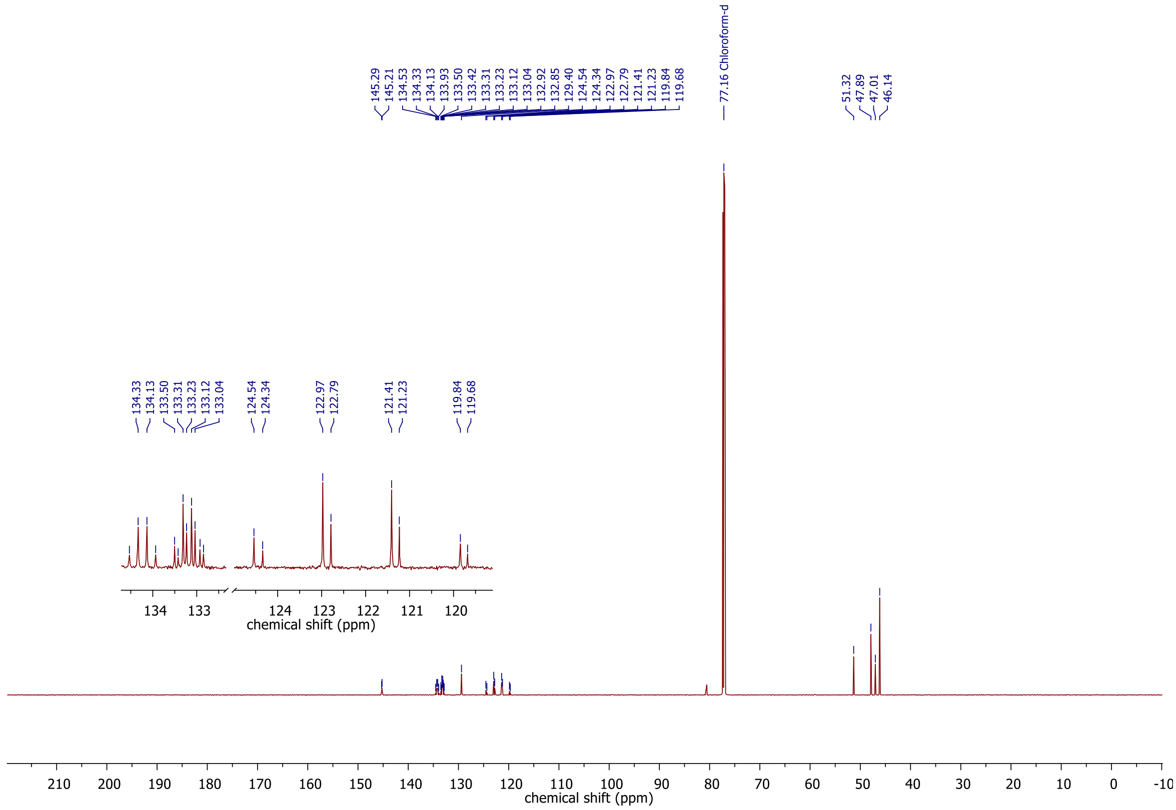

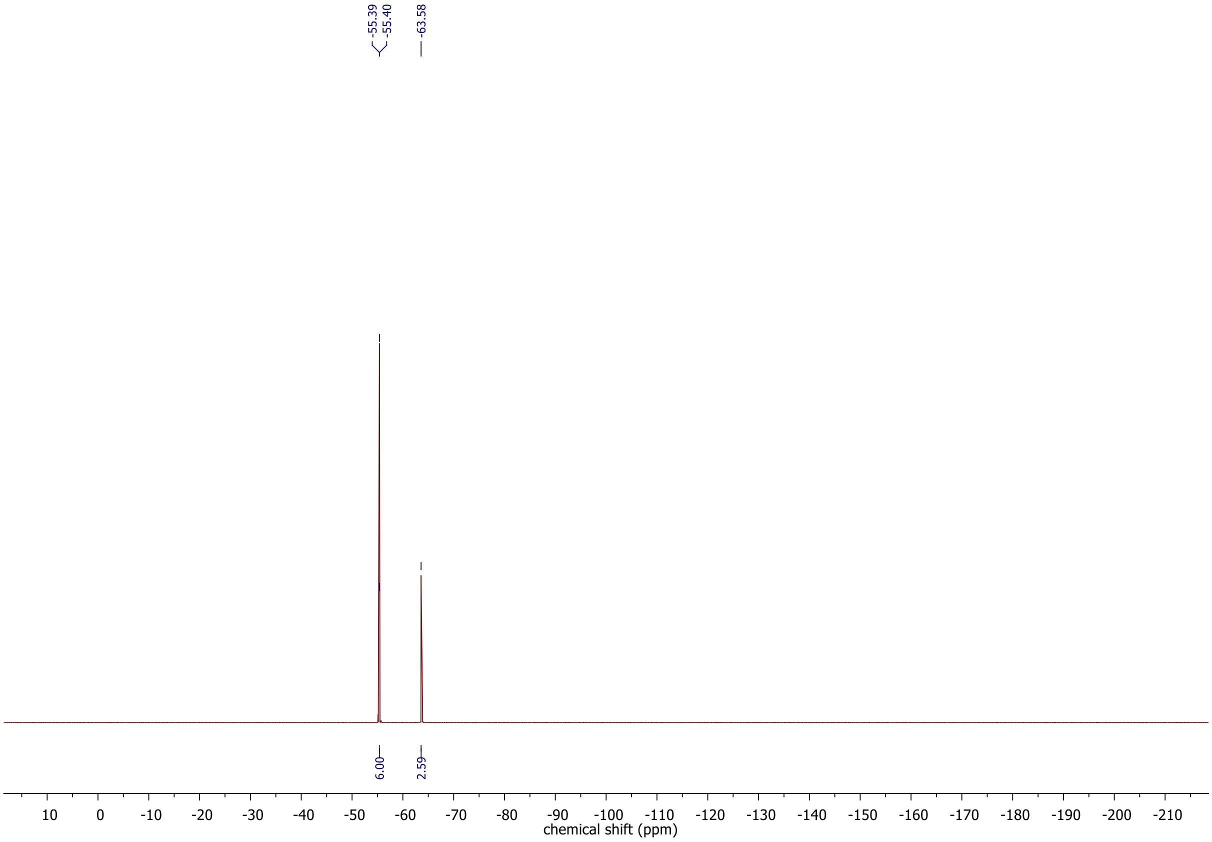
-55.39
-55.40
-63.58
6.00
2.59
10 0 -10 -20 -30 -40 -50 -60 -70 -80 -90 -100 -110 -120 -130 -140 -150 -160 -170 -180 -190 -200 -210
chemical shift (ppm)

(2*E*,4*E*)-5-(Benzo[d][1,3]dioxol-5-yl)-1-(4-((2,4,6-tris(trifluoromethyl)phenyl)sulfonyl)piperazin-1-yl)penta-2,4-dien-1-one (14)

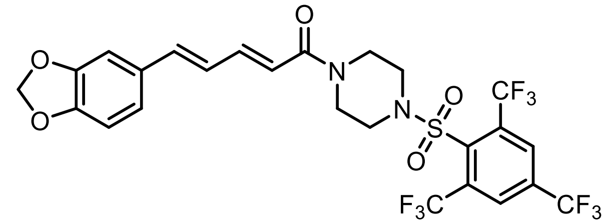

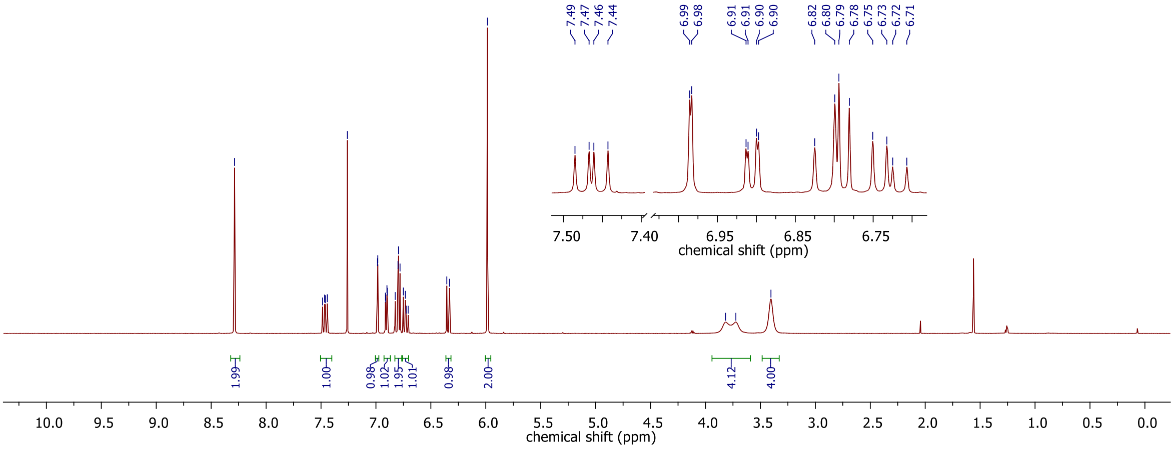

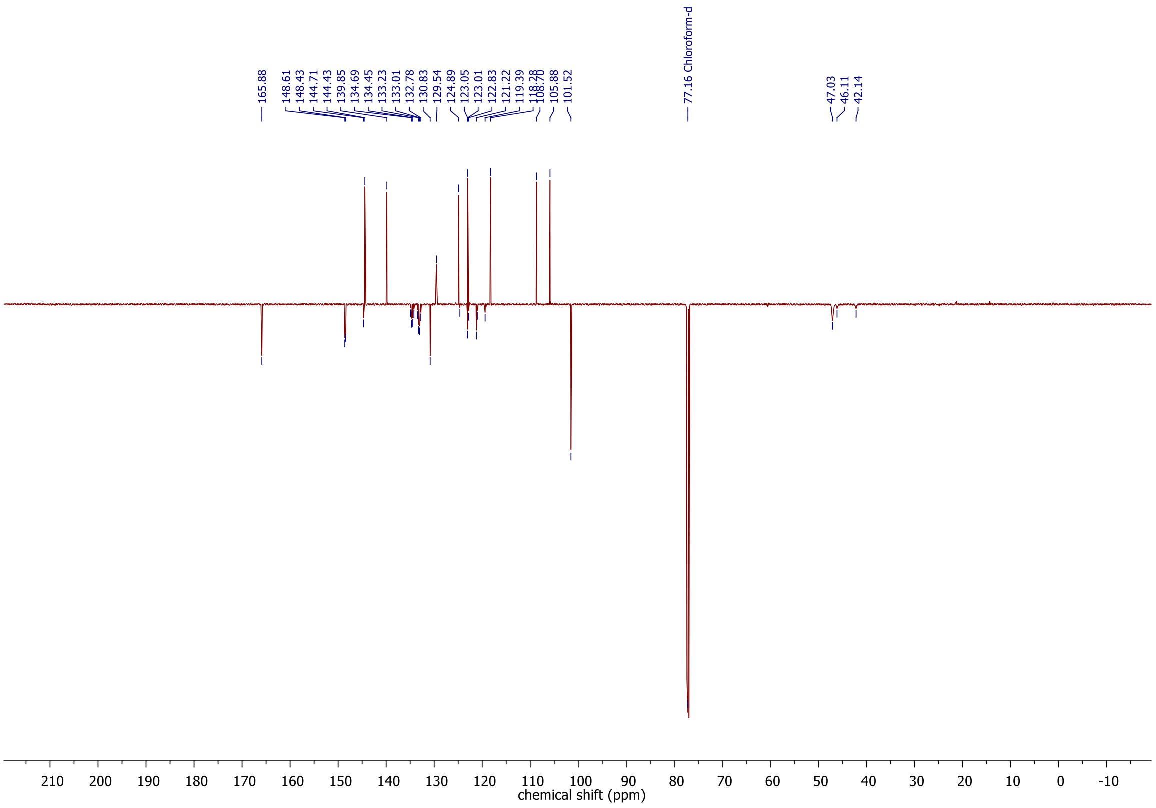

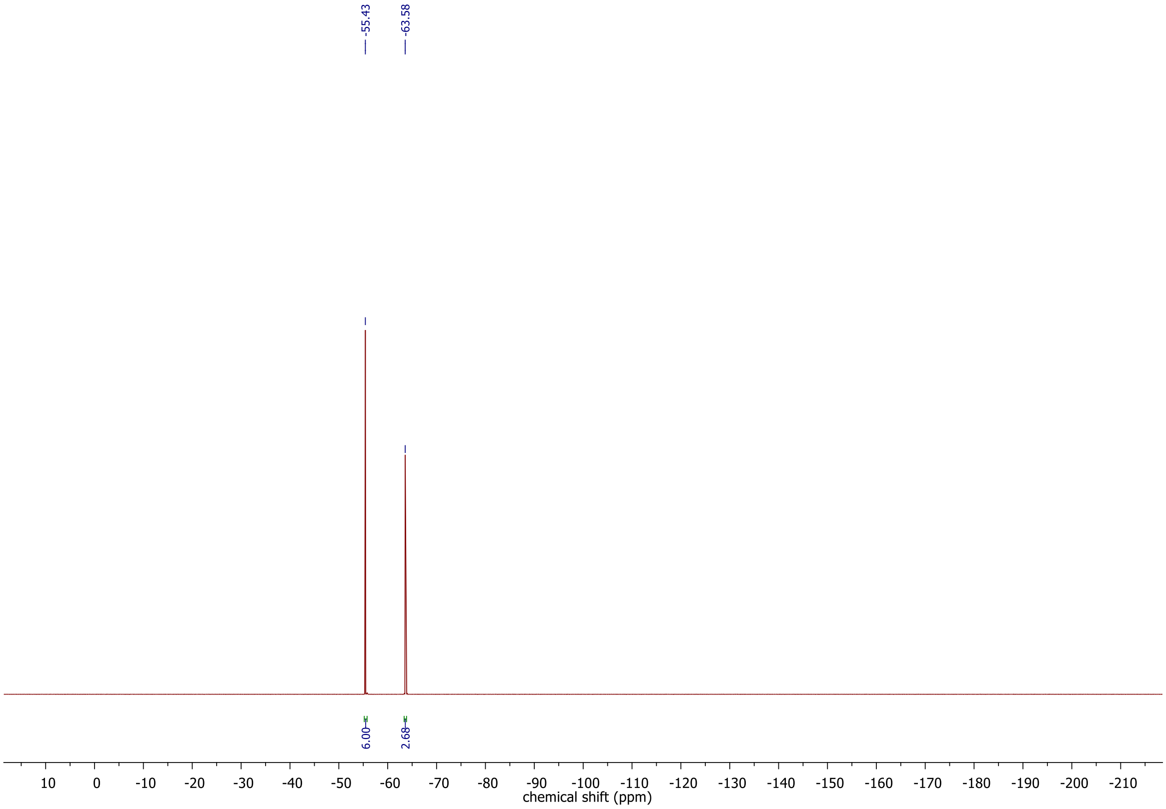
-55.43
-63.58
6.00
2.68
10 0 -10 -20 -30 -40 -50 -60 -70 -80 -90 -100 -110 -120 -130 -140 -150 -160 -170 -180 -190 -200 -210
chemical shift (ppm)

8. References

[1] P. Spieß, A. Sirvent, I. Tiefenbrunner, J. Sargueil, A. J. Fernandes, A. Arroyo-Bondía, R. Meyrelles, D. Just, A. Prado-Roller, S. Shaaban, D. Kaiser, N. Maulide, *Chem. Eur. J.* **2023**, *29*, e202301312.

[2] S. H. Kim, S. H. Hong, *Org. Lett.* **2016**, *18*, 212–215.

[3] Y. Kuwahara, A. Zhang, H. Soma, A. Tsuda, *Org. Lett.* **2012**, *14*, 3376–3379.

[4] Y. Katayama, K. Watanabe, Y. Nishiyama, S. Yokoshima, *Org. Lett.* **2023**, *25*, 1326–1330.

[5] L. E. Evans, A. Krishna, Y. Ma, T. E. Webb, D. C. Marshall, C. L. Tooke, J. Spencer, T. B. Clarke, A. Armstrong, A. M. Edwards, *J. Med. Chem.* **2019**, *62*, 4411–4425.

[6] W. König, R. Geiger, *Chem. Ber.* **1970**, *103*, 788–798.

[7] L. Zhou, J. Qiu, C. Wang, F. Zhang, K. Yang, Q. Song, *Org. Lett.* ***2022**, 24, 3249–3253*

[8] F. G. Njoroge, R. J. Doll, B. Vibulbhan, C. S. Alvarez, W. R. Bishop, J. Petrin, P. Kirschmeier, N. I. Carruthers, J. K. Wong, M. M. Albanese, J. J. Piwinski, J. Catino, V. Girijavallabhan, A. K. Ganguly, *Bioorg. Med. Chem.* **1997**, *5*, 101–113.

[9] J. Su, J.-N. Mo, X. Chen, A. Umanzor, Z. Zhang, K. N. Houk, J. Zhao, *Angew. Chem. Int. Ed.* **2022**, *61*, e202112668.

[10] R. D. Slack, A. M. Abramyan, H. Tang, S. Meena, B. A. Davis, A. Bonifazi, J. B. Giancola, J. R. Deschamps, S. Naing, H. Yano, S. K. Singh, A. H. Newman, L. Shi, *ACS Chem. Neurosci.* **2019**, *10*, 3946–3952.

[11] K. Bhanukiran, S. K. Singh, R. Singh, A. Kumar, S. Hemalatha, *ACS Chem. Neurosci.* **2023**, *14*, 2743–2760.

[12] P. Pracht, F. Bohle, S. Grimme, *Phys. Chem. Chem. Phys.* **2020**, *22*, 7169–7192.

[13] S. Grimme, *J. Chem. Theory Comput.* **2019**, *15*, 2847–2862.

[14] J.-D. Chai, M. Head-Gordon, *Phys. Chem. Chem. Phys.* **2008**, *10*, 6615–6620.

[15] F. Weigend, *Phys. Chem. Chem. Phys.* **2006**, *8*, 1057–1065.

[16] F. Weigend, R. Ahlrichs, *Phys. Chem. Chem. Phys.* **2005**, *7*, 3297–3305.

[17] Gaussian 16, Revision C. 01, M. J. Frisch, G. W. Trucks, H. B. Schlegel, G. E. Scuseria, M. A. Robb, J. R. Cheeseman, G. Scalmani, V. Barone, G. A. Petersson, H. Nakatsuji, X. Li, M. Caricato, A. V. Marenich, J. Bloino, B. G. Janesko, R. Gomperts, B. Mennucci, H. P. Hratchian, J. V. Ortiz, A. F. Izmaylov, J. L. Sonnenberg, D. Williams-Young, F. Ding, F. Lipparini, F. Egidi, J. Goings, B. Peng, A. Petrone, T. Henderson, D. Ranasinghe, V. G. Zakrzewski, J. Gao, N. Rega, G. Zheng, W. Liang, M. Hada, M. Ehara, K. Toyota, R. Fukuda, J. Hasegawa, M. Ishida, T. Nakajima, Y. Honda, O. Kitao, H. Nakai, T. Vreven, K. Throssell, J. A. Montgomery, J. E. Peralta, F. Ogliaro, M. J. Bearpark, J. J. Heyd, E. N. Brothers, K. N. Kudin, V. N. Staroverov, T. A. Keith, R. Kobayashi, J. Normand, K. Raghavachari, A. P. Rendell, J. C. Burant, S. S. Iyengar, J. Tomasi, M. Cossi, J. M. Millam, M. Klene, C. Adamo, R. Cammi, J. W. Ochterski, R. L. Martin, K. Morokuma, O. Farkas, J. B. Foresman, and D. J. Fox, Inc., Wallingford CT, **2016**.

[18] E. Cancès, B. Mennucci, J. Tomasi, *J. Chem. Phys.* **1997**, *107*, 3032–3041.

[19] A. V. Marenich, C. J. Cramer, D. G. Truhlar, *J. Phys. Chem. B* **2009**, *113*, 6378–6396.
